# Epidemiological analysis of porcine reproductive and respiratory syndrome viruses in 2020–2023 in China and the impact of serum acclimatization on production performance of sows farm

**DOI:** 10.3389/fvets.2025.1614039

**Published:** 2025-06-23

**Authors:** Shuangxi Li, Jinlian Hua

**Affiliations:** ^1^College of Veterinary Medicine, Northwest A&F University, Yangling, China; ^2^Shaanxi Centre of Stem Cells Engineering & Technology, Yangling, China

**Keywords:** PRRSV, prevalence, time of infection, serum acclimatization, reproductive performance, production performance

## Abstract

Porcine reproductive and respiratory syndrome (PRRS), caused by PRRS virus (PRRSV), leads to severe economic losses in the pig farming industry. Currently, there is no specific treatment for PRRSV. This study investigated the epidemiological characteristics of PRRSV in large-scale pig farms across 24 provinces and municipalities in China from 2020 to 2023. Subsequently, serum acclimatization of gilts was implemented in large-scale pig farms to evaluate its impact on the reproductive performance of sows and the growth performance of piglets. The results showed that 14 provinces had PRRSV-positive rates exceeding 10%, with Yunnan Province reporting the highest rate at 23.5%, whereas Zhejiang had the lowest at 3.5%. The PRRSV-positive rate exhibited clear seasonality, with markedly higher rates in winter and spring compared to summer and autumn. The introduction of gilts was identified as the most important cause of PRRS outbreaks, with an incidence rate as high as 49%. Following serum acclimatization, the average total number of piglets per litter, the average number of live piglets per litter, the number of piglets weaned per litter, and the total weaning weight per litter were all significantly higher than those before acclimatization and in unacclimated sows (*p* < 0.05). The results indicated that serum acclimatization could effectively improve the reproductive performance of sows and the growth performance of piglets. This study provides valuable data for understanding the epidemiology of PRRSV and supports serum acclimatization as a potential strategy for PRRSV prevention and control in China.

## 1 Introduction

Porcine reproductive and respiratory syndrome (PRRS), commonly referred to as blue ear disease in pigs, is a highly contagious and lethal disease caused by the PRRS virus (PRRSV), posing a serious threat to the swine industry (1, 2). It is also a typical immunosuppressive disease, often leading to severe immune suppression, including the inhibition of antibody production (3, 4). Additionally, PRRSV frequently co-infects with bacteria, parasites, and other viruses, further complicating prevention and control efforts. Currently, effective control of this disease remains elusive. PRRS causes reproductive disorders in pregnant sows and respiratory symptoms in pigs of all ages (5, 6). Infected pregnant sows may experience decreased farrowing rates, with abortion, stillbirth, mummification, and weak piglets affecting 30 to 100% of cases. Infected fattening pigs often exhibit poor appetite, dyspnea, multiple secondary infections, slow growth, and reduced feed efficiency (7).

PRRSV, a member of the genus Betaarterivirus, has spherical or ovoid, enveloped virions. These virions measure 45–80 nm in diameter and contain an icosahedral nucleocapsid structure. The PRRSV genome is approximately 15.4 kb in length (8). PRRSV is divided into two gene types: the European type (PRRSV-1), represented by the Lelystad virus (LV strain), and the North American type (PRRSV-2), represented by the VR-2332 strain (9, 10).

The primary strategy for preventing PRRS currently relies on vaccination, with a vaccination rate of 70% in pig farms across China. The effectiveness and safety of vaccines are crucial criteria in their development and deployment. Presently, PRRS attenuated live vaccines are highly effective (11); however, they carry a potential risk of pathogenicity. In contrast, PRRS inactivated vaccines offer sufficient safety but exhibit poor immunogenicity. They are effective primarily against homologous strains, offering inadequate protection against heterologous strains and lacking cross-protection capabilities. Consequently, their overall effectiveness remains unsatisfactory (4, 7, 12–14).

Serum acclimatization is a technique used to protect pigs by deliberately controlling the timing of herd infections and administering serum or tissues containing low-dose viruses. In recent years, PRRS serum acclimatization has become a widely adopted and effective method for preventing and controlling PRRS in large-scale pig farms. This approach has gained increasing support from pig farm managers and veterinarians (15). The process involves collecting positive serum from specific serotypes of PRRSV strains, diluting it in appropriate proportions, and inoculating the entire pig herd with the serum. This induces a high level of uniform antibodies, thereby blocking virus excretion and protecting uninfected pigs, ultimately controlling PRRSV within the farm (16). The advantages of serum acclimatization include the ability to manage the infection timing, achieve uniform antibody levels, maintain herd consistency, and potentially enhance the reproductive and production performance of sows in affected farms.

During this experiment, 28,054 samples were collected from pig farms across 24 provinces and municipalities in China, spanning January 2020 to June 2023. The objective was to investigate the epidemiological characteristics of PRRSV in China. Subsequently, in large-scale pig farms located in Xuwen County, Guangdong Province, serum acclimatization was applied to gilts. The reproductive performance of sows and the production performance of piglets were then measured. The aim was to determine whether serum acclimatization could enhance the reproductive performance of sows and the production performance of piglets. This study provides new data and experiential references for the prevention and control of PRRSV in large-scale pig farms, offering new directions and ideas for PRRSV management.

## 2 Materials and methods

### 2.1 Main reagents and instruments

The magnetic bead method virus DNA/RNA extraction kit was obtained from Hangzhou Bioer Technology Co., Ltd. (Hangzhou, China). The PRRSV RT-qPCR detection kit was acquired from Thermo Fisher Scientific Co., Ltd. (Shanghai, China). Compound amoxicillin powder (no. 110802092) was sourced from Zhejiang Hisun Animal Healthcare Products Co., Ltd. (Hangzhou, China).

The fully automated nucleic acid extraction and purification instrument, NPA-32P, was obtained from Hangzhou Bioer Technology Co., Ltd. (Hangzhou, China). The nucleic acid concentration was determined using the NanoDrop One instrument from Thermo Fisher Scientific Co., Ltd. (Shanghai, China). For real-time fluorescent quantitative PCR, the LightCycler 96 instrument, acquired from Roche Co., Ltd. (Basel, Switzerland), was used.

### 2.2 Analysis of the epidemiological characteristics of PRRSV in large-scale pig farms across different regions

#### 2.2.1 Analysis of the detection rate of PRRSV

Between January 2020 and June 2023, samples were collected from large-scale pig farms across 24 provinces and municipalities. On average, 13,454 blood samples were taken per province, amounting to a total of 322,896 samples for nucleic acid detection. The detailed information of pig farms was listed in Supplementary Table S1.

#### 2.2.2 Analysis of the time of PRRSV infection

From July 2021 to June 2023, blood and throat swab samples were collected monthly from 961 individual pig farms, representing 192 companies across 24 provinces and municipalities. Nucleic acid tests were then conducted on these samples. The detailed information of pig farms and the positive number of samples were listed in Supplementary Tables S2, S3.

#### 2.2.3 Analysis of the number of pig farms with PRRS

From January 2021 to December 2022, the large-scale pig farms across the country was surveyed and analyzed each month. The number of farms surveyed is detailed in Table 1. The study statistically analyzed the number of pig farms affected by PRRS and the incidence rate of this disease. The detailed information of pig farms was listed in Supplementary Table S4.

**Table 1:** The number of pig farms surveyed each month.

Years	Jan	Feb	Mar	Apr	May	Jun	Jul	Aug	Sep	Oct	Nov	Dec
2021	552	563	593	618	632	638	637	609	585	534	488	481
2022	486	471	460	441	409	391	386	388	395	405	422	430

### 2.3 Serum acclimatization on large-scale pig farm

#### 2.3.1 The sample source of serum acclimatization

A positive serum sample containing a strain of the NADC30-like gene type of PRRSV was obtained from a large-scale pig farm in Guangdong, where this strain is prevalent. Subsequently, piglets testing positive for PRRSV were selected and euthanized. Serum samples were collected and confirmed to be positive only for the PRRSV antigen using RT-qPCR. These serum samples were sent to Shanghai Sangon Biotech Co., Ltd. for sequencing of the viral nucleic acids. The positive serum, containing PRRSV, was then quantified and stored at −80°C for future serum acclimatization.

#### 2.3.2 The procedure of serum acclimatization

Based on quantitative results, the serum was diluted to ensure each milliliter contained 500 virions. Subsequently, 90-day-old gilts received an injection of 2 mL of serum containing 1,000 virions administered into the neck muscle. Compound amoxicillin was provided via water for three consecutive days, beginning on the day of injection. The serum injection was repeated on the third day. Within 10 to 14 days following the initial serum injection, PRRSV antibodies began developing in the gilts. One month post-acclimatization, the PRRSV antibody positive rate in the gilts reached 100%, confirming the success of the serum acclimatization process.

#### 2.3.3 The time and number of gilts for serum acclimatization

The serum acclimatization site was located on a pig farm in Xuwen County, Guangdong Province. From February 2021 and July 2022, the farm did not engage in serum acclimatization, and during this period, there were 3,817 gilts. From August 2022 to December 2023, a total of 11,363 gilts underwent acclimatization. Concurrently, the number of non-acclimatization gilts totaled 18,251. Statistical analyses were conducted on the reproductive performance of sows and the growth performance of piglets.

### 2.4 RT-qPCR detection

Viral RNA was extracted using the magnetic bead method, followed by RT-qPCR to detect PRRSV nucleic acids. The RT-qPCR program included reverse transcription at 45°C for 10 min and pre-denaturation at 95°C for 10 min. This was followed by 40 cycles of denaturation at 95°C for 15 s and annealing/extension at 60°C for 45 s. The judgment criteria were as follows, based on the amplification curve and cycle threshold (Ct) value: a Ct value of ≤40 indicated a PRRSV-positive result. If the Ct value was between 40 and 45, the experiment was repeated. Should the Ct value remain in this range or fall below 40 upon repetition, the sample was deemed PRRSV-positive. A Ct value >45 or the absence of an amplification curve indicated a negative result for PRRSV. The threshold of Ct value is set 40 based on commercialized reagent kit (purchased from Thermo Fisher Scientific Co., Ltd). The detection method used in this study was also in accordance with the instructions provided in the kit.

### 2.5 Statistical analysis

Statistical analysis was performed using SPSS 27.0 software. Independent-samples T test was used for comparison between groups. *p*-value of less than 0.05 was considered statistically significant, P* < 0.05, P** < 0.01, P*** < 0.001.

Statistical analysis was conducted using SPSS 27.0 software. An independent-samples t-test was employed to compare the groups. A *p*-value of less than 0.05 was considered statistically significant, with * indicating *p* < 0.05, ** indicating *p* < 0.01, and *** indicating *p* < 0.001.

## 3 Results

### 3.1 Sequencing analysis of PRRSV strain

Positive samples with Ct values below 30 were selected for sequencing of the ORF5 region. To obtain the sequences, PCR was performed using the specific primer pair PRRSV-ORF5-F and PRRSV-ORF5-R. PCR products were then analyzed via electrophoresis on 1% agarose gels. The resulting fragments were cloned into pEASY-blunt vectors (TransGen Biotech, Beijing), and the positive clones were sequenced at Sangon Biotech (Qingdao, China). A phylogenetic tree was constructed using the neighbor-joining method in MEGA v.7.1.0 software with default settings, including 1,000 bootstrap replicates, based on the gene sequences obtained in this study and reference sequences from GenBank. The initial tree was drawn to scale, with branch lengths representing the number of substitutions per site. The resulting tree was visualized using iTOL v.6 (Interactive Tree of Life, http://itol.embl.de/).

**Figure 1:**
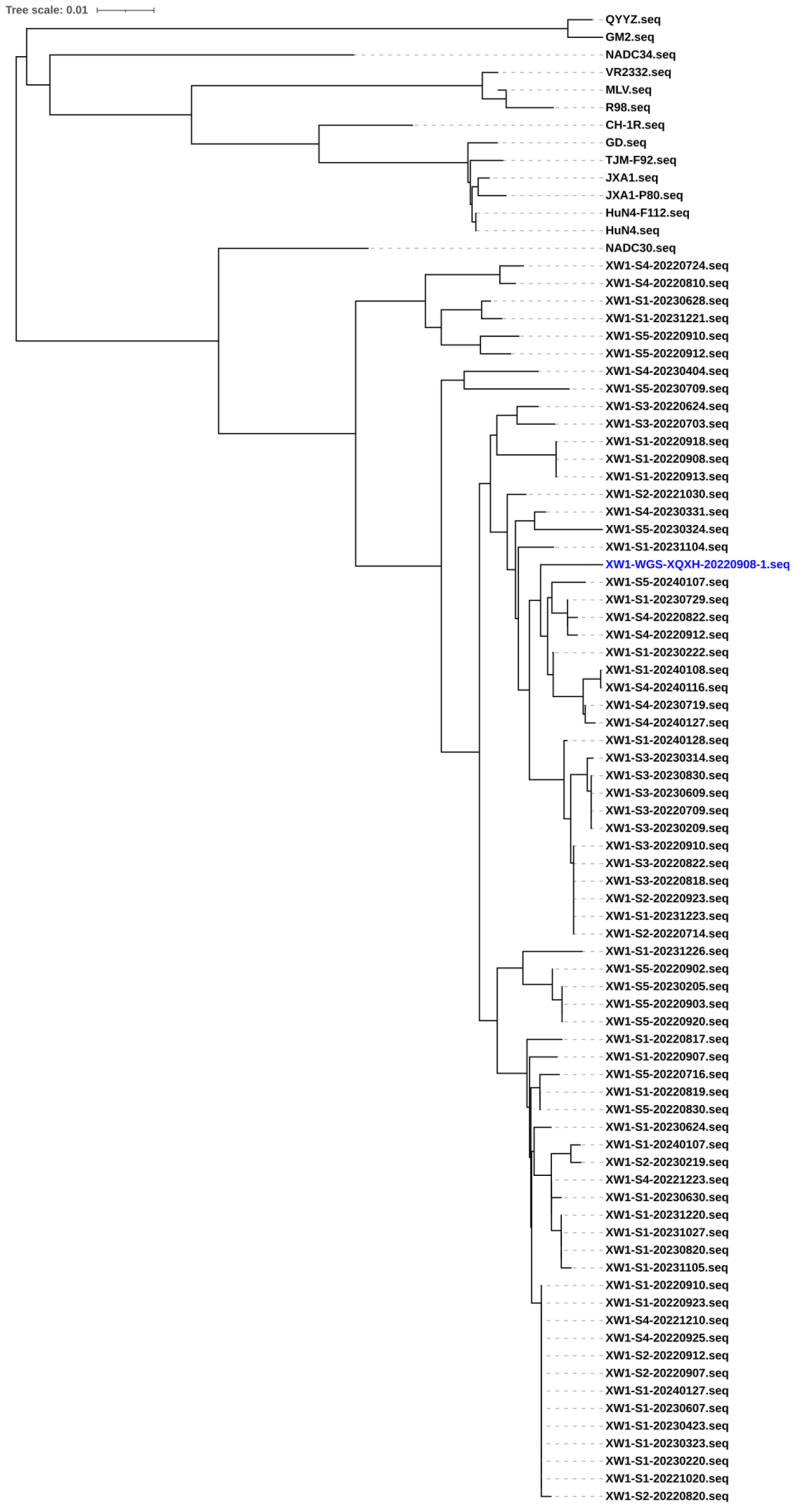
Phylogenetic trees of reference strains of PRRSV and the representative strains of PRRSV were constructed based on the ORF5 gene sequences. Positive samples with Ct values below 30 were selected for sequencing of the ORF5 region. PCR was performed using the specific primer pair PRRSV-ORF5-F and PRRSV-ORF5-R. PCR products were analyzed.

All the sequenced strains cluster into a single large branch (Figure 1). Compared to the reference strain, these sequenced strains exhibit the highest homology with the NADC30 strain. Additionally, the homology among the sequenced virus strains themselves is relatively high. Consequently, a representative strain, named XW1-WGS-XQXH-20220908-1, was selected for the experiment.

### 3.2 Epidemiological analysis of PRRSV

#### 3.2.1 Analyses of the detection rate of PRRSV in different regions

**Figure 2:**
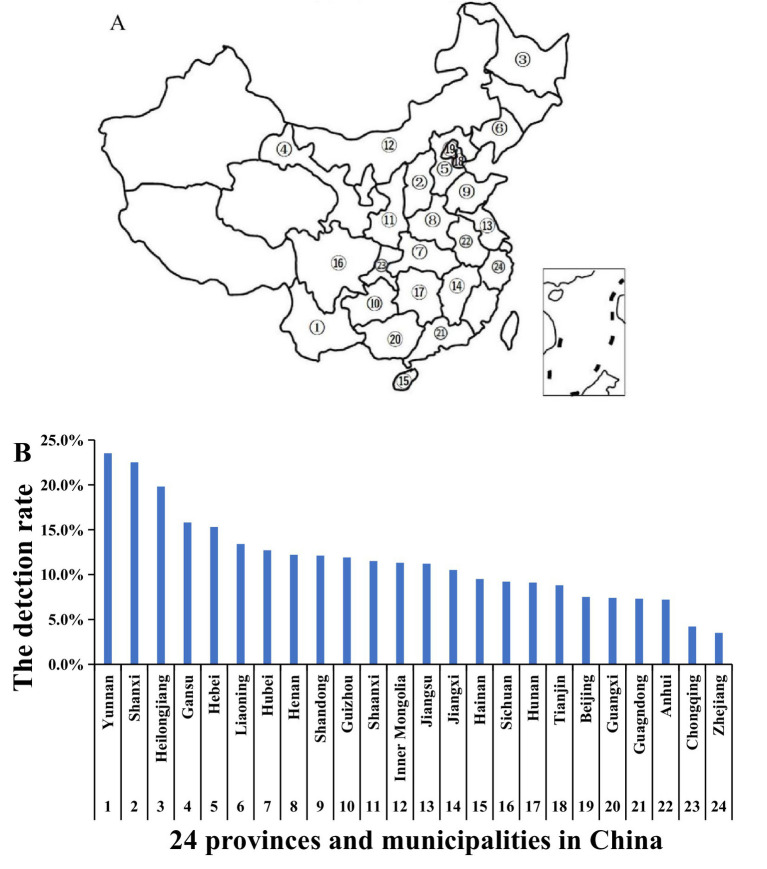
The PRRSV positive rate in 24 provinces and municipalities. **(A)** The samples from 24 provinces and municipalities in China. **(B)** The PRRSV positive detection rate. Between January 2020 and June 2023, samples were collected from large-scale pig farms across 24 provinces and municipalities. ①–㉔ represent different provinces and municipalities in China.

The analysis results revealed that, among the 24 provinces and municipalities (Figure 2A), Yunnan Province had the highest positive detection rate at 23.5%. In contrast, Zhejiang had the lowest rate at 3.5%. Two provinces, Yunnan and Shanxi, exhibited a positive rate exceeding 20%, while 14 provinces had a positive rate exceeding 10% (Figure 2B).

#### 3.2.2 Analysis of the time of PRRSV infection

**Figure 3:**
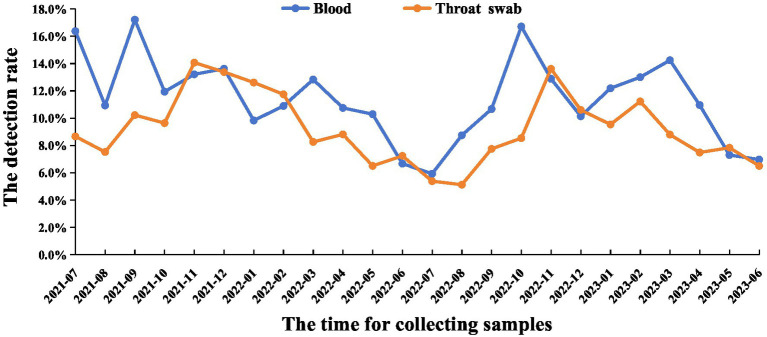
The time of PRRSV infection. From July 2021 to June 2023, blood and throat swab samples were collected monthly from 961 individual pig farms across 24 province sand municipalities. Nucleic acid in a total of 495,711 blood samples and 418,788 throat swab samples was measured by RT-qPCR.

The results demonstrated that the positive rate of PRRSV displayed clear seasonality. Specifically, the positive rate was significantly higher during winter and spring (October to March) compared to summer and autumn (April to September). Additionally, no significant difference was observed between the detection rates from abnormal pig blood samples and throat swab samples, with both exhibiting similar detection patterns. The average positive rate of blood samples was 11.4%, the average positive rate of throat swab samples was 9.2% (Figure 3).

#### 3.2.3 Analysis of the number of pig farms with PRRSV infection

**Figure 4:**
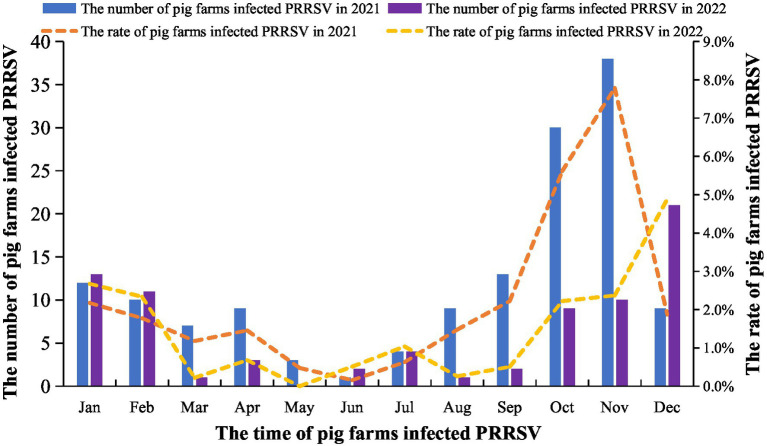
The number of pig farms and incidence rate with PRRSV infection. From January 2021 to December 2022, the large-scale pig farms across the country was surveyed and analyzed each month. The pig farms with obvious clinical symptoms of PRRS and positive nucleic acid results are identified as PRRS-positive farms.

The results indicated that the number of infected pig farms and the incidence rate were higher in 2021 than in 2022. The lowest number of infections occurred from May to July, while the number of infections and the incidence rate peaked from October to February. Additionally, the 2021 infection numbers surpassed those of 2022 (Figure 4).

### 3.3 The impact of serum acclimatization on the reproductive performance of gilts and the growth performance of piglets

#### 3.3.1 The impact of serum acclimatization on the reproductive performance of gilts

**Figure 5:**
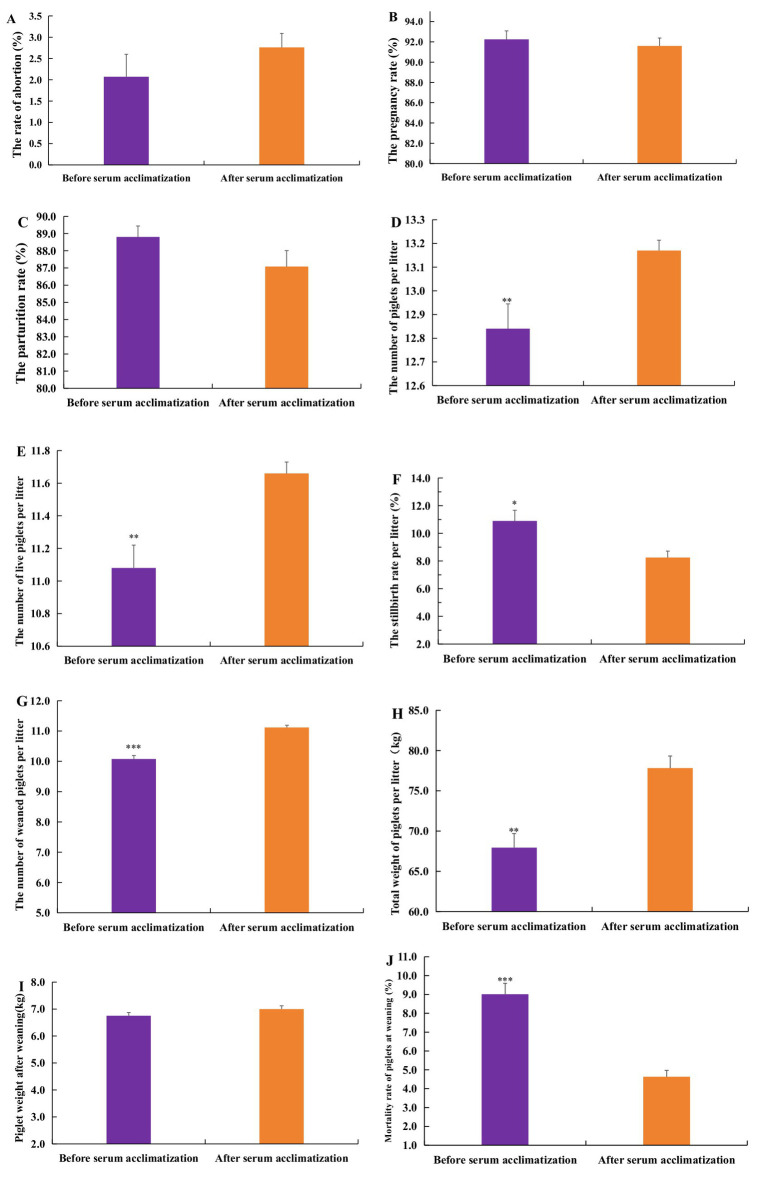
The impact of serum acclimatization on the reproductive performance of gilts and the growth performance of piglets. **(A)** The effects of serum acclimatization on the abortion rate. **(B)** The effects of serum acclimatization on the pregnancy rate. **(C)** The effects of serum acclimatization on the parturition rate. **(D)** The effects of serum acclimatization on the average total number of piglets per litter. **(E)** The effects of serum acclimatization on the average number of live piglets per litter. **(F)** The effects of serum acclimatization on the average stillbirth rate per litter. **(G)** The effects of serum acclimatization on the average number of weaned piglets per litter. **(H)** The effects of serum acclimatization on the average total weight of piglets per litter. **(I)** The effects of serum acclimatization on the weight of weaned piglets. **(J)** The effects of serum acclimatization on the mortality rate of weaned piglets. From February 2021 and July 2022, there were 3,817 gilts which did not engage in serum acclimatization. From August 2022 to December 2023, the gilts were acclimated monthly, the total number of acclimatization gilts was 11,363. A *p*-value of less than 0.05 was considered statistically significant, with * indicating *p* < 0.05, ** indicating *p* < 0.01, and *** indicating *p* < 0.001.

Figure 5 illustrated the effects of serum acclimatization on the reproductive performance of gilts in pig farms. The findings indicate no significant differences in the abortion rate, pregnancy rate, or parturition rate of gilts before and after acclimatization (Figures 5A–C). However, the average total number of piglets per litter post-acclimatization is 13.17, significantly greater than the pre-acclimatization number (*p* < 0.01) (Figure 5D). Similarly, the average number of live piglets per litter post-acclimatization was 11.66, a significant increase compared to pre-acclimatization levels (*p* < 0.01) (Figure 5E). Furthermore, the average stillbirth rate per litter post-acclimatization decreased significantly to 8.25%, compared to pre-acclimatization figures (*p* < 0.05) (Figure 5F).

#### 3.3.2 The impact of serum acclimatization on the growth performance of piglets

The results indicated that the average number of weaned piglets per litter after serum acclimatization was 11.12, which is significantly higher than the number before acclimatization (*p* < 0.05) (Figure 5G). The average total weight of piglets per litter post-serum acclimation was 77.82 kg, also significantly greater than the weight before acclimatization (*p* < 0.01) (Figure 5H). However, there was no significant difference in the individual weight of weaned piglets before and after serum acclimatization (*p* > 0.05) (Figure 5I). Furthermore, the mortality rate of weaned piglets after serum acclimatization was 4.63%, notably lower than the rate before acclimation (*p* < 0.01) (Figure 5J).

### 3.4 The impact of serum acclimatization and non-acclimatization on the reproductive performance of gilts and growth performance of piglets

#### 3.4.1 The effect of acclimatization and non-acclimatization on the abortion rate of gilts

**Figure 6:**
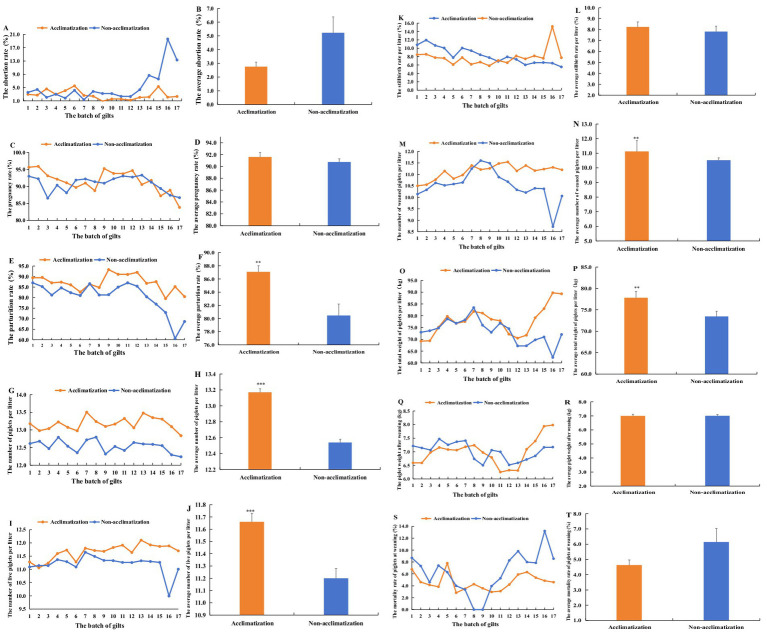
The effect of serum acclimatization and non-acclimatization on the reproductive performance of gilts and the growth performance of piglets from the 1st batch of gilts to 17th batch. **(A)** The comparison of the abortion rate in different batch gilts between serum acclimatization and non-acclimatization. **(B)** The comparison of average abortion rates between acclimatization and non-acclimatization. **(C)** The comparison of pregnancy rate between acclimatization and non-acclimatization. **(D)** The comparison of average pregnancy rates between acclimatization and non-acclimatization. **(E)** The comparison of parturition rate between acclimatization and non-acclimatization. **(F)** The comparison of average parturition rates between acclimatization and non-acclimatization. **(G)** The comparison of the number of piglets per litter between acclimatization and non-acclimatization. **(H)** The comparison of the average number of piglets per litter between acclimatization and non-acclimatization. **(I)** The comparison of the number of live piglets per litter between acclimatization and non-acclimatization. **(J)** The comparison of the average number of live piglets per litter between acclimatization and non-acclimatization. **(K)** The comparison of the stillbirth rate per litter between acclimatization and non-acclimatization. **(L)** The comparison of the average stillbirth rate per litter between acclimatization and non-acclimatization. **(M)** The comparison of the number of weaned piglets per litter between acclimatization and non-acclimatization. **(N)** The comparison of the average number of weaned piglets per litter between acclimatization and non-acclimatization. **(O)** The comparison of the total weight of piglets per litter between acclimatization and non-acclimatization. **(P)** The comparison of the average total weight of piglets per litter between acclimatization and non-acclimatization. **(Q)** The comparison of the weight of weaned piglets between acclimatization and non-acclimatization. **(R)** The comparison of the average weight of weaned piglets between acclimatization and non-acclimatization. **(S)** The comparison of the mortality rate of weaned piglets between acclimatization and non-acclimatization. **(T)** The comparison of the average mortality rate of weaned piglets between acclimatization and non-acclimatization. From August 2022 to December 2023, a total of 11,363 gilts underwent acclimatization. Concurrently, the number of non-acclimatization gilts totaled 18,251. From April 2023, the gilts acclimatized began to give birth every month until August 2024. The reproductive performance and the growth performance were analyzed and compared each month. These pigs were divided into 17 batches according to the duration of parturition (one batch/month).

The results showed that from the 1st batch of gilts to the 7th batch, the abortion rates between acclimatization and non-acclimatization gilts were similar and did not differ significantly (*p* > 0.05). However, from the 8th batch of gilts to the 17th batch, the non-acclimatization gilts exhibited consistently higher abortion rates compared to the acclimatization gilts. Notably, the highest abortion rate for non-acclimatization gilts reaching 19.63% in the 16th batch of gilts (Figure 6A). Throughout the entire period, the average abortion rate for acclimatization gilts was 2.76%, which was lower than the 5.22% observed for non-acclimatization gilts. This difference, however, was not statistically significant (*p* > 0.05) (Figure 6B).

#### 3.4.2 The effect of acclimatization and non-acclimatization on the pregnancy rate of gilts

The results indicated that from the 1st batch of gilts to the 5th batch, the pregnancy rate of the acclimatization gilts consistently exceeded that of non-acclimatization gilts. However, from the 6th batch of gilts to the 17th batch, the pregnancy rates of acclimatization and non-acclimatization gilts were closely similar, with no significant difference observed (Figure 6C). Over the entire period, the average pregnancy rate of the acclimatization gilts was 91.59%, which was slightly higher than the 90.75% for non-acclimatization gilts, though this difference was not statistically significant (*p* > 0.05) (Figure 6D).

#### 3.4.3 The effect of acclimatization and non-acclimatization on the parturition rate of gilts

The results indicated that, from the 1st batch of gilts to the 17th batch, the parturition rate of the acclimatization gilts consistently exceeded that of the non-acclimatization except the 7th batch. The most pronounced difference in parturition rates occurred in 16th batch (Figure 6E). Over the entire period, the average parturition rate for acclimatization gilts was significantly higher than for non-acclimatization gilts (*p* < 0.01) (Figure 6F).

#### 3.4.4 The effect of acclimatization and non-acclimatization on the total number of piglets per litter

The results indicated that from 1st batch of gilts to the 17th batch, the total number of piglets per litter was consistently higher in the serum acclimatization gilts compared to non-acclimatization (Figure 6G). Over the entire period, the average total number of piglets per litter in serum acclimatization gilts was 13.17, which was significantly higher than that in non-acclimatization gilts (*p* < 0.001) (Figure 6H).

#### 3.4.5 The effect of acclimatization and non-acclimatization on the number of live piglets per litter

The results showed that from 3rd batch of gilts to the 17th batch, the acclimatization gilts consistently produced a higher number of live piglets per litter compared to the non-acclimatization gilts (Figure 6I). Over the entire period, the average number of live piglets per litter for the serum acclimatization gilts was 11.66, significantly surpassing that of the non-acclimatization gilts (*p* < 0.001) (Figure 6J).

#### 3.4.6 The effect of acclimatization and non-acclimatization on the stillbirth rate per litter

The results indicated that, in the 16th batch, the stillbirth rate per litter for acclimatization gilts was significantly lower than that of non-acclimatization gilts. During other periods, the stillbirth rates per litter for the acclimatization and non-acclimatization gilts were similar (Figure 6K). Over the entire study period, no significant difference was observed in the stillbirth rate per litter between the two groups of gilts (*p* > 0.05) (Figure 6L).

#### 3.4.7 The effect of acclimatization and non-acclimatization on the number of weaned piglets per litter

The results indicated that, in the 8th and 9th batch, the number of weaned piglets per litter in the serum acclimatization group was slightly lower than in the non-acclimatization group. However, during other periods, the acclimatization group exhibited a higher number of weaned piglets per litter compared to the non-acclimatization group (Figure 6M). Overall, the average number of weaned piglets per litter throughout the entire period was significantly higher in the acclimatization group than in the non-acclimatization group (*p* < 0.01) (Figure 6N).

#### 3.4.8 The effect of acclimatization and non-acclimatization on the total weight of piglets per litter

The results indicated that from 1st batch of gilts to 11th batch, the total weight of piglets per litter in the acclimatization group followed a trend similar to that of the non-acclimatization group. However, from 12th batch to 17th batch, the total weight per litter in the acclimatization group exceeded that of the non-acclimatization group, with the difference increasing progressively (Figure 6O). Over the entire period, the average total weight of piglets per litter in the acclimatization group was significantly higher than that of the non-acclimatization group (*p* < 0.01) (Figure 6P).

#### 3.4.9 The effect of acclimatization and non-acclimatization on the weight of weaned piglets

The results indicated that, the weight trend of weaned piglets in acclimatization closely resembled that of the non-acclimatization group (Figure 6Q). No significant difference was observed in the weight of weaned piglets between the acclimatization and non-acclimatization groups throughout the entire period (*p* > 0.05) (Figure 6R).

#### 3.4.10 The effect of acclimatization and non-acclimatization on the mortality rate of weaned piglets

The results indicated that from 1st batch of gilts to 9th batch, the mortality rate of weaned piglets in the acclimatization group was similar to that of the non-acclimatization group. However, from 10th batch of gilts to 17th batch, the mortality rate of weaned piglets in the acclimatization group was lower than that of the non-acclimatization group (Figure 6S). Across the entire period, the mortality rate of weaned piglets in the acclimatization group remained lower than that in the non-acclimatization group, although the difference was not statistically significant (*p* > 0.05) (Figure 6T).

## 4 Discussion

PRRS is caused by PRRSV and is considered one of the most serious swine diseases affecting the pig industry in China and globally (5, 17, 18, 32). PRRSV is a highly variable RNA virus, and traditional vaccination methods increasingly fail to offer effective cross-protection, particularly against NADC30-like PRRSV strains. Consequently, conducting an epidemiological investigation of large-scale pig farms is crucial for understanding the characteristics of PRRS. Recent studies have addressed the genetic diversity and prevalence of PRRSV in China, yet many have been restricted by regional scope, farm size, or small sample sizes (19–24). In response to these limitations, this study examines the incidence rate, timing of infection, number of affected pig farms, and causes of PRRSV across 244 large-scale pig farms in 24 provinces and municipalities in China.

The results revealed variability in the detection rate of PRRSV across 24 provinces and municipalities. Yunnan Province recorded the highest positive rate at 23.5%, while Zhejiang exhibited the lowest at 3.5%. Notably, 14 provinces reported a positive rate exceeding 10%. This indicates regional differences influenced by varying terrain, altitude, and climate. Arruda et al. highlighted the impact of these factors on the transmission of airborne diseases, such as PRRS (25, 26). Additionally, the results demonstrated clear seasonality in the positive rate of PRRSV, with significantly higher rates in winter and spring compared to summer and autumn. The introduction of gilts is identified as the primary catalyst for PRRS outbreaks, with an incidence as high as 49%. Other studies have confirmed the pronounced seasonality of PRRSV incidence (27), aligning with our survey results. In summary, by analyzing samples from 24 provinces and municipalities, we have investigated the incidence and diversity of PRRSV in China. These findings provide valuable epidemiological data and insights that enhance the understanding of PRRS from an epidemiological standpoint and may contribute to the prevention and control of PRRSV in China.

Serum acclimatization of PRRS has emerged as an effective method for the clinical prevention and control of the disease in large-scale pig farms in recent years. This approach has gained support from an increasing number of pig farm managers and veterinarians. Serum acclimatization offers procedural advantages. First, the acclimatization timing of the pigs can be artificially controlled, allowing for greater consistency. Second, the purity of the acclimatization PRRSV serum type can enhance the reproductive performance of sows and the productive performance of piglets to some extent (15, 33). In this study, the pathogen responsible for a PRRS outbreak at a large-scale pig farm in Xuwen County, Guangdong Province, was isolated and identified. An analysis of its genomic characteristics confirmed that it belongs to the NADC30-PRRSV strain. Since this isolated strain represents the current prevalent strains and was obtained from this particular farm, it holds a certain level of representativeness. Consequently, the positive serum from this isolated strain was used for serum acclimatization.

Regarding serum acclimatization methods, some studies recommend two injections within a short timeframe (28, 29), while others advocate for a single injection (15, 16). Results consistently demonstrate that single and double injection protocols yield significantly better outcomes than no serum acclimatization. However, no comparative studies between these two methods have been published. Thus, in this experiment, the protocol of administering two injections over three days was selected. Although this approach increases labor costs, it addresses the large number of gilts involved and mitigates concerns about improper injections potentially affecting acclimatization outcomes. The experiment confirmed that serum acclimatization is effective for PRRS prevention and control. Future research will focus on comparing these methods to identify the most effective and cost-efficient strategy.

The results demonstrated no significant differences in abortion rate, pregnancy rate, parturition rate, and the average weight of weaned piglets before and after acclimatization. However, significant differences were found in the average total number of piglets per litter, average number of live piglets per litter, average stillbirth rate per litter, the number of piglets weaned per litter, total weight of piglets weaned per litter, and the mortality rate of weaned piglets before and after acclimatization. When compared to non-acclimatization, serum acclimatization resulted in significant improvements in parturition rate, average total number of piglets per litter, average number of live piglets per litter, number of piglets weaned per litter, and total weaning weight per litter. The above results indicate that serum acclimatization can improve the reproductive performance of sows and the production performance of piglets to a certain extent. Similar findings were reported by Wu et al. (15), who observed a significant increase in the average number of live piglets per litter following serum acclimatization. However, in this study, there were no significant differences in abortion rate or pregnancy rate of gilts after acclimatization when compared with either pre-acclimatization or un-acclimatization sows. This suggests that serum acclimatization has no significant effect on abortion rate and pregnancy rate of sows.

Serum acclimatization is an effective method for preventing and controlling PRRSV, but it must be performed following strict operational standards to ensure safety and efficacy. Combining our experimental results with data from previous studies, we find that the success of serum acclimatization primarily involves the following aspects: First, it is essential to rigorously test the serum for pathogens before acclimatization. This testing ensures that all pathogens are negative except for the PRRSV antigen, which confirms the disease infection status of the pigs and prevents the spread of other diseases during subsequent adaptation phases. Second, sequencing of the PRRSV strain is necessary to determine whether the strain infecting the pig farm is the dominant epidemic strain in the farm or region in recent years. Third, determining the optimal dilution ratio of the acclimatization serum is crucial. This step ensures that pigs produce high levels of antibodies quickly post-acclimatization while maintaining relatively mild clinical symptoms of PRRS, avoiding severe stress, abortion, and other adverse effects (30).

Serum acclimatization is recognized as an effective strategy for preventing and controlling PRRS, but it faces certain challenges in practice. Notably, the purified serum used in acclimatization often fails to provide cross-protection against various PRRSV strains, particularly those with significant genetic divergence. Moreover, as an RNA virus, PRRSV is prone to mutations, posing a risk of virulence reversion after several generations of transmission in pig farms. Consequently, there exists a potential for virulence reversion during the serum acclimatization process, which could exacerbate PRRS-related damage (31). Therefore, it is crucial to continuously monitor the clinical symptoms of pigs after acclimatization. A key issue in clinical production is how to reduce the virulence of the domesticated PRRSV strain while enhancing its infectivity, enabling a lower viral dose to convert the pig herd to a positive status without causing severe symptoms. Additionally, serum acclimatization requires stringent disease management, efficient production operations, and strong team confidence. In large-scale pig farms, where multiple diseases are prevalent and management is suboptimal, inadequate technical infrastructure can hinder the success of serum acclimatization (30).

Despite the ongoing mutation of PRRSV, PRRS continues to pose a significant threat as a major infectious disease within the global pig industry. It remains a critical target for prevention and control efforts on pig farms. In China, the pressure to manage PRRS in pig farms is particularly substantial. However, with ongoing, in-depth research into the epidemiology and fundamental aspects of PRRS, alongside the enhancement of prevention and control measures, comprehensive prevention and control technologies for PRRS are expected to improve significantly. Consequently, PRRS will increasingly become a controllable, preventable, and treatable disease.

## 5 Conclusion

This study investigated the incidence of PRRSV, the timing of infection, the number of infected farms, and the causes of the disease in large-scale pig farms across China. Following this, serum acclimatization was applied to gilts in these farms to assess its impact on the reproductive performance of sows and the production performance of piglets. The findings showed that the PRRSV-positive rate was highest in Yunnan Province and lowest in Zhejiang Province. The prevalence of PRRSV exhibited significant seasonality, with higher incidence during winter and spring. The introduction of gilts was identified as the primary cause of PRRS outbreaks. Post-serum acclimatization, improvements were observed in the reproductive performance of sows and production performance of piglets. These results suggest that serum acclimatization is an effective strategy for the prevention and control of PRRS in large-scale pig farms. These results provide national-level epidemiological data, advancing the understanding of PRRS from an epidemiological perspective, and may aid in the prevention and control of PRRSV in China. They also offer data support and experiential references for large-scale pig farms to prevent and control PRRS through serum acclimatization.

## Data Availability

The original contributions presented in the study are included in the article/supplementary material, further inquiries can be directed to the corresponding author.
