# Supplementary material for: Epidemiological analysis of porcine reproductive and respiratory syndrome viruses in 2020–2023 in China and the impact of serum acclimatization on production performance of sows farm
Source: Front Vet Sci. 2025 Jun 23;12:1614039. doi: 10.3389/fvets.2025.1614039 (PMC12229867; doi:10.3389/fvets.2025.1614039)
Supplement: Supplementary file 1 [file Data_Sheet_1.docx]

**Supplementary materials**

1. *The proportion of different Ct of positive results in different samples when analyzing the time of PRRSV infection*

The results showed 54790 blood samples were positive, the positive rate was the highest (40.49%) when the Ct value was 30≤Ct<35. When Ct values were 25≤Ct<30 and 35≤Ct<40, the positive rates were 25.79% and 19.65% respectively. When Ct value was Ct<20, the positive rates was the lowest (2.44%) (Fig. S1A). The results showed 57931 throat swab samples were positive, the positive rate was the highest (49.54%) when the Ct value was 30≤Ct<35. When Ct values were 35≤Ct<40 and 25≤Ct<30, the positive rates were 30.41% and 17.29% respectively. When Ct value was Ct<20, the positive rates was the lowest (0.25%) (Fig. S1B). The positive rates in blood samples and throat swab samples were compared. The results showed that the positive rates in throat swab samples were higher than that in blood samples when Ct values were 30≤Ct<35 and 35≤Ct<40. The positive rates in blood samples were higher than that in throat swab samples when Ct values were Ct<20, 20≤Ct<25 and 25≤Ct<30 (Fig. S1C).”

**Fig. S1. The proportion of different Ct of positive results in different samples**

A, The proportion of different Ct of positive results in blood samples. B, the proportion of different Ct of positive results in throat swap samples. C, the comparison of different Ct of positive results between blood sample and throat swap samples.

1. *The standard curve for ensuring the number of virions in serum acclimatization*

The standard curve was Y=-3.6864X+39.954, R^2^ was 0.9998 (Fig. S2).

**Fig. S2. The standard curve for ensuring the number of virions**

1. *The antibody levels after acclimatization at 14d and 30d in serum acclimatization*

The antibodies were measured on the 14 and 30 days after acclimatization respectively. The results showed that at 14 days, half of the gilts had positive antibodies, and at 30 days, all of them were positive (Fig. S3A). In addition, S/P values showed that at 14 days, S/P values of the gilts all were all larger than 1.0, at 30 days, S/P values were between 1.5 and 2.5 (Fig. S3B). Therefore, it could be inferred that after 30 days of acclimatization, the antibodies appeared in all gilts.

**Fig. S3. The antibody levels after acclimatization at 14d and 30d**

A, the antibody levels after acclimatization. B, the S/P value after acclimatization at 14d and 30d.
