# Supplementary material for: Epidemiological analysis of porcine reproductive and respiratory syndrome viruses in 2020–2023 in China and the impact of serum acclimatization on production performance of sows farm
Source: Front Vet Sci. 2025 Jun 23;12:1614039. doi: 10.3389/fvets.2025.1614039 (PMC12229867; doi:10.3389/fvets.2025.1614039)
Supplement: Supplementary file 2 [file Table_1.docx]

The information of pig farms for collecting blood samples from January 2020 to June 2023 in 24 provinces and municipalities was listed in Table S1. All sow farms are the standard farm with a scale of 3,000. The scale of fattening farms are 1,500. PRRS vaccine for sow farms is administered three times a year. The fattening farms are not immunized with PRRS vaccine.

**Table S1.** **The information of pig farms for collecting blood samples from January 2020 to June 2023 in 24 provinces and municipalities**

| **Province or municipality** | **Company** | **The pig farm** | **Type of pig farm** | **The number of blood samples/month** | **The total number of blood samples** | **The numbers of positive samples** |
| --- | --- | --- | --- | --- | --- | --- |
| Anhui Province | Anhui Xinliu Breeding Co., Ltd. | Fuyang Qingfeng pig farm | Sow farm | 8 | 336 | 30 |
| Anhui Province | Anhui Xinliu Breeding Co., Ltd. | Fuyang Xinmei pig farm | Sow farm | 8 | 336 | 22 |
| Anhui Province | Tongcheng City Xinliu Agriculture and Animal Husbandry Technology Co., Ltd. | Xiangshan No.4 farm | Sow farm | 8 | 336 | 37 |
| Anhui Province | Tongcheng City Xinliu Agriculture and Animal Husbandry Technology Co., Ltd. | Xiangshan No.4 farm | Sow farm | 8 | 336 | 42 |
| Anhui Province | Tongcheng City Xinliu Agriculture and Animal Husbandry Technology Co., Ltd. | Xiangshan No.2 farm | Sow farm | 8 | 336 | 21 |
| Anhui Province | Tongcheng City Xinliu Agriculture and Animal Husbandry Technology Co., Ltd. | Xiangshan No.1 farm | Sow farm | 8 | 336 | 35 |
| Anhui Province | Wuhe New Hope Liuhe Animal Husbandry Co., Ltd. | Xiaoxi No.1 farm | Sow farm | 8 | 336 | 17 |
| Anhui Province | Wuhe New Hope Liuhe Animal Husbandry Co., Ltd. | Xiaoxi No.5 farm | Sow farm | 8 | 336 | 22 |
| Anhui Province | Wuhe New Hope Liuhe Animal Husbandry Co., Ltd. | Daxi No.2 farm | Sow farm | 8 | 336 | 23 |
| Anhui Province | Wuhe New Hope Liuhe Animal Husbandry Co., Ltd. | Daxi No.3 farm | Sow farm | 8 | 336 | 18 |
| Anhui Province | Wuhe New Hope Liuhe Animal Husbandry Co., Ltd. | Xiaoxi No.2 farm | Sow farm | 8 | 336 | 14 |
| Anhui Province | Wuhe New Hope Liuhe Animal Husbandry Co., Ltd. | Xiaoxi No.3 farm | Sow farm | 8 | 336 | 14 |
| Anhui Province | Wuhe New Hope Liuhe Animal Husbandry Co., Ltd. | Xiaoxi No.4 farm | Sow farm | 8 | 336 | 17 |
| Anhui Province | Xiaoxian Liuhe Buchang Breeding Co., Ltd. | Xiao county pig farm | Sow farm | 8 | 336 | 36 |
| Anhui Province | Suixi County Xinhao Agriculture and Animal Husbandry Co., Ltd. | Daliangzhuang No.3 farm | Fattening farm | 8 | 336 | 44 |
| Anhui Province | Suixi County Xinhao Agriculture and Animal Husbandry Co., Ltd. | Daliangzhuang No.2 farm | Fattening farm | 8 | 336 | 23 |
| Anhui Province | Tongcheng City Xinliu Agriculture and Animal Husbandry Technology Co., Ltd. | Qipanling No.2 farm | Fattening farm | 8 | 336 | 30 |
| Anhui Province | Tongcheng City Xinliu Agriculture and Animal Husbandry Technology Co., Ltd. | Qipanling No.6 farm | Fattening farm | 8 | 336 | 19 |
| Anhui Province | Tongcheng City Xinliu Agriculture and Animal Husbandry Technology Co., Ltd. | Qipanling No.4 farm | Fattening farm | 8 | 336 | 28 |
| Anhui Province | Tongcheng City Xinliu Agriculture and Animal Husbandry Technology Co., Ltd. | Qipanling No.5 farm | Fattening farm | 8 | 336 | 21 |
| Anhui Province | Wuhe New Hope Liuhe Animal Husbandry Co., Ltd. | Dengyu No.1 farm | Fattening farm | 8 | 336 | 19 |
| Anhui Province | Wuhe New Hope Liuhe Animal Husbandry Co., Ltd. | Qiaozhang No.1 farm | Fattening farm | 8 | 336 | 14 |
| Anhui Province | Wuhe New Hope Liuhe Animal Husbandry Co., Ltd. | Changhuai No.14 farm | Fattening farm | 8 | 336 | 19 |
| Anhui Province | Wuhe New Hope Liuhe Animal Husbandry Co., Ltd. | Changhuai No.15 farm | Fattening farm | 8 | 336 | 14 |
| Anhui Province | Wuhe New Hope Liuhe Animal Husbandry Co., Ltd. | Changhuai No.11 farm | Fattening farm | 8 | 336 | 27 |
| Anhui Province | Wuhe New Hope Liuhe Animal Husbandry Co., Ltd. | Zhuyu No.1 farm | Fattening farm | 8 | 336 | 19 |
| Anhui Province | Xiaoxian Liuhe Buchang Breeding Co., Ltd. | Xiao county fattening farm | Fattening farm | 8 | 336 | 28 |
| Beijing | Beijing Xinliu Agriculture and Animal Husbandry Co., Ltd. | Xiling No.1 farm | Sow farm | 8 | 336 | 24 |
| Beijing | Beijing Xinliu Agriculture and Animal Husbandry Co., Ltd. | Xiling No.2 farm | Sow farm | 8 | 336 | 16 |
| Beijing | Beijing Xinliu Agriculture and Animal Husbandry Co., Ltd. | Xiling Fattening No.1 farm | Fattening farm | 8 | 336 | 22 |
| Beijing | Beijing Xinliu Agriculture and Animal Husbandry Co., Ltd. | Xiling Fattening No.6 farm | Fattening farm | 8 | 336 | 29 |
| Beijing | Beijing Xinliu Agriculture and Animal Husbandry Co., Ltd. | Xiling Fattening No.4 farm | Fattening farm | 8 | 336 | 40 |
| Beijing | Beijing Xinliu Agriculture and Animal Husbandry Co., Ltd. | Xiling Fattening No.4 farm | Fattening farm | 8 | 336 | 17 |
| Beijing | Beijing Xinliu Agriculture and Animal Husbandry Co., Ltd. | Xiling Fattening No.5 farm | Fattening farm | 8 | 336 | 28 |
| Gansu Province | Baiyin New Hope Agriculture and Animal Husbandry Technology Co., Ltd. | Jingyuan Ancestral Generation | Sow farm | 8 | 336 | 77 |
| Gansu Province | Baiyin New Hope Agriculture and Animal Husbandry Technology Co., Ltd. | Jingyuan Second farm | Sow farm | 8 | 336 | 62 |
| Gansu Province | Baiyin New Hope Agriculture and Animal Husbandry Technology Co., Ltd. | Jingyuan Sixth farm | Sow farm | 8 | 336 | 45 |
| Gansu Province | Baiyin New Hope Agriculture and Animal Husbandry Technology Co., Ltd. | Jingyuan Third farm | Sow farm | 8 | 336 | 83 |
| Gansu Province | Baiyin New Hope Agriculture and Animal Husbandry Technology Co., Ltd. | Jingyuan Fourth farm | Sow farm | 8 | 336 | 51 |
| Gansu Province | Baiyin New Hope Agriculture and Animal Husbandry Technology Co., Ltd. | Jingyuan Fifth farm | Sow farm | 8 | 336 | 62 |
| Gansu Province | Baiyin New Hope Agriculture and Animal Husbandry Technology Co., Ltd. | Jingyuan First farm | Sow farm | 8 | 336 | 58 |
| Gansu Province | Gansu Xinliu Agriculture and Animal Husbandry Technology Co., Ltd. | Yumen Ancestral Generation | Sow farm | 8 | 336 | 79 |
| Gansu Province | Gansu Xinliu Agriculture and Animal Husbandry Technology Co., Ltd. | Yumen Second farm | Sow farm | 8 | 336 | 56 |
| Gansu Province | Gansu Xinliu Agriculture and Animal Husbandry Technology Co., Ltd. | Yumen First farm | Sow farm | 8 | 336 | 47 |
| Gansu Province | Gansu New Hope Liuhe Agriculture and Animal Husbandry Co., Ltd. | Tuanzhuang Ancestral Generation | Sow farm | 8 | 336 | 46 |
| Gansu Province | Gansu New Hope Liuhe Agriculture and Animal Husbandry Co., Ltd. | Zhongchuan First farm | Sow farm | 8 | 336 | 75 |
| Gansu Province | Gansu New Hope Liuhe Agriculture and Animal Husbandry Co., Ltd. | Tuanzhuang Eighth farm | Sow farm | 8 | 336 | 30 |
| Gansu Province | Gansu New Hope Liuhe Agriculture and Animal Husbandry Co., Ltd. | Tuanzhuang Second farm | Sow farm | 8 | 336 | 24 |
| Gansu Province | Gansu New Hope Liuhe Agriculture and Animal Husbandry Co., Ltd. | Tuanzhuang Sixth farm | Sow farm | 8 | 336 | 52 |
| Gansu Province | Gansu New Hope Liuhe Agriculture and Animal Husbandry Co., Ltd. | Tuanzhuang Seventh farm | Sow farm | 8 | 336 | 73 |
| Gansu Province | Gansu New Hope Liuhe Agriculture and Animal Husbandry Co., Ltd. | Tuanzhuang Third farm | Sow farm | 8 | 336 | 68 |
| Gansu Province | Gansu New Hope Liuhe Agriculture and Animal Husbandry Co., Ltd. | Tuanzhuang Fourth farm | Sow farm | 8 | 336 | 37 |
| Gansu Province | Gansu New Hope Liuhe Agriculture and Animal Husbandry Co., Ltd. | Tuanzhuang Fifth farm | Sow farm | 8 | 336 | 55 |
| Gansu Province | Gansu New Hope Liuhe Agriculture and Animal Husbandry Co., Ltd. | Tuanzhuang First farm | Sow farm | 8 | 336 | 61 |
| Gansu Province | Gansu New Hope Liuhe Agriculture and Animal Husbandry Co., Ltd. | Zhongchuan Second farm | Sow farm | 8 | 336 | 30 |
| Gansu Province | Gansu New Hope Liuhe Agriculture and Animal Husbandry Co., Ltd. | Zhongchuan Sixth farm | Sow farm | 8 | 336 | 69 |
| Gansu Province | Gansu New Hope Liuhe Agriculture and Animal Husbandry Co., Ltd. | Zhongchuan Third farm | Sow farm | 8 | 336 | 53 |
| Gansu Province | Gansu New Hope Liuhe Agriculture and Animal Husbandry Co., Ltd. | Zhongchuan Fourth farm | Sow farm | 8 | 336 | 29 |
| Gansu Province | Gansu New Hope Liuhe Agriculture and Animal Husbandry Co., Ltd. | Zhongchuan Fifth farm | Sow farm | 8 | 336 | 47 |
| Gansu Province | Gansu New Hope Liuhe Agriculture and Animal Husbandry Co., Ltd. | Tongda Pig farm | Sow farm | 8 | 336 | 62 |
| Gansu Province | Gansu New Hope Liuhe Agriculture and Animal Husbandry Co., Ltd. | Zhongchuan Twenty-Third farm | Sow farm | 8 | 336 | 37 |
| Gansu Province | Baiyin New Hope Agriculture and Animal Husbandry Technology Co., Ltd. | Tianzhu Wanbo Reserve Breeding farm | Fattening farm | 8 | 336 | 45 |
| Gansu Province | Gansu Xinliu Agriculture and Animal Husbandry Technology Co., Ltd. | Yumen Sixth farm | Fattening farm | 8 | 336 | 73 |
| Gansu Province | Gansu Xinliu Agriculture and Animal Husbandry Technology Co., Ltd. | Yumen Third farm | Fattening farm | 8 | 336 | 49 |
| Gansu Province | Gansu Xinliu Agriculture and Animal Husbandry Technology Co., Ltd. | Yumen Fourth farm | Fattening farm | 8 | 336 | 57 |
| Gansu Province | Gansu Xinliu Agriculture and Animal Husbandry Technology Co., Ltd. | Yumen Fifth farm | Fattening farm | 8 | 336 | 32 |
| Gansu Province | Lanzhou New Hope Feed Co., Ltd. | Lanzhou New Hope Free-Range Service Department | Fattening farm | 8 | 336 | 28 |
| Guangdong Province | Guangdong Xinhao Zhenghe Agriculture and Animal Husbandry Co., Ltd. | Longgui Sixth farm | Sow farm | 8 | 336 | 21 |
| Guangdong Province | Guangdong Xinhao Zhenghe Agriculture and Animal Husbandry Co., Ltd. | Longgui Fifth farm | Sow farm | 8 | 336 | 57 |
| Guangdong Province | Guangdong Xinhao Zhenghe Agriculture and Animal Husbandry Co., Ltd. | Longgui Second farm | Fattening farm | 8 | 336 | 22 |
| Guangdong Province | Guangdong Xinhao Zhenghe Agriculture and Animal Husbandry Co., Ltd. | Longgui Third farm | Fattening farm | 8 | 336 | 47 |
| Guangdong Province | Guangdong Xinhao Zhenghe Agriculture and Animal Husbandry Co., Ltd. | Longgui Fourth farm | Fattening farm | 8 | 336 | 39 |
| Guangdong Province | Guangdong Xinhao Zhenghe Agriculture and Animal Husbandry Co., Ltd. | Longgui First farm | Fattening farm | 8 | 336 | 31 |
| Guangdong Province | Qingyuan Xinhao Agriculture and Animal Husbandry Co., Ltd. | Qingyuan Xiaosanjian Pig farm | Fattening farm | 8 | 336 | 28 |
| Guangdong Province | Qingyuan Xinhao Agriculture and Animal Husbandry Co., Ltd. | Qingyuan Lianxing Pig farm | Sow farm | 8 | 336 | 9 |
| Guangdong Province | Ruyuan Yao Autonomous County Xinhao Agriculture and Animal Husbandry Co., Ltd. | Ruyuan Ddong Pig farm | Sow farm | 8 | 336 | 17 |
| Guangdong Province | Ruyuan Yao Autonomous County Xinhao Agriculture and Animal Husbandry Co., Ltd. | Ddong First farm | Sow farm | 8 | 336 | 22 |
| Guangdong Province | Ruyuan Yao Autonomous County Xinhao Agriculture and Animal Husbandry Co., Ltd. | Ddong Second farm | Sow farm | 8 | 336 | 41 |
| Guangdong Province | Ruyuan Yao Autonomous County Xinhao Agriculture and Animal Husbandry Co., Ltd. | Ddong Third farm | Sow farm | 8 | 336 | 32 |
| Guangdong Province | Taian City Xincheng Agriculture and Animal Husbandry Co., Ltd. Wengyuan Xinmu | Wengyuan Xiangxing Second District | Sow farm | 8 | 336 | 28 |
| Guangdong Province | Taian City Xincheng Agriculture and Animal Husbandry Co., Ltd. Wengyuan Xincheng | Wengyuan Xiangxing Third District | Sow farm | 8 | 336 | 22 |
| Guangdong Province | Taian City Xincheng Agriculture and Animal Husbandry Co., Ltd. Wengyuan Xincheng | Wengyuan Xiangxing First District | Sow farm | 8 | 336 | 10 |
| Guangdong Province | Zhaoqing Xinhao Agriculture and Animal Husbandry Co., Ltd. | Shidong Third farm | Sow farm | 8 | 336 | 17 |
| Guangdong Province | Zhaoqing Xinhao Agriculture and Animal Husbandry Co., Ltd. | Shidong Fourth farm | Sow farm | 8 | 336 | 15 |
| Guangdong Province | Zhaoqing Xinhao Agriculture and Animal Husbandry Co., Ltd. | Shidong Second farm | Sow farm | 8 | 336 | 9 |
| Guangdong Province | Zhaoqing Xinhao Agriculture and Animal Husbandry Co., Ltd. | Shidong First farm | Sow farm | 8 | 336 | 11 |
| Guangdong Province | Zhaoqing Xinhao Agriculture and Animal Husbandry Co., Ltd. | Shidong Sixth farm | Sow farm | 8 | 336 | 13 |
| Guangxi Zhuang Autonomous Region | Guangxi Luocheng Xinhao Agriculture and Animal Husbandry Co., Ltd. | Shuangmeng Fifth farm | Sow farm | 8 | 336 | 23 |
| Guangxi Zhuang Autonomous Region | Guangxi Luocheng Xinhao Agriculture and Animal Husbandry Co., Ltd. | Shuangmeng Fattening Second farm | Sow farm | 8 | 336 | 44 |
| Guangxi Zhuang Autonomous Region | Guangxi Luocheng Xinhao Agriculture and Animal Husbandry Co., Ltd. | Shuangmeng Fattening First farm | Sow farm | 8 | 336 | 69 |
| Guangxi Zhuang Autonomous Region | Guigang Xinliu Agriculture and Animal Husbandry Technology Co., Ltd. | Mapi Sixth farm | Sow farm | 8 | 336 | 11 |
| Guangxi Zhuang Autonomous Region | Guigang Xinliu Agriculture and Animal Husbandry Technology Co., Ltd. | Mapi Second farm | Sow farm | 8 | 336 | 29 |
| Guangxi Zhuang Autonomous Region | Guigang Xinliu Agriculture and Animal Husbandry Technology Co., Ltd. | Mapi Third farm | Sow farm | 8 | 336 | 21 |
| Guangxi Zhuang Autonomous Region | Guigang Xinliu Agriculture and Animal Husbandry Technology Co., Ltd. | Mapi Fourth farm | Sow farm | 8 | 336 | 40 |
| Guangxi Zhuang Autonomous Region | Guigang Xinliu Agriculture and Animal Husbandry Technology Co., Ltd. | Mapi Fifth farm | Sow farm | 8 | 336 | 28 |
| Guangxi Zhuang Autonomous Region | Guigang Xinliu Agriculture and Animal Husbandry Technology Co., Ltd. | Mapi First farm | Sow farm | 8 | 336 | 29 |
| Guangxi Zhuang Autonomous Region | Hezhou Xinliu Co., Ltd. | Wanggao Sixth farm | Sow farm | 8 | 336 | 31 |
| Guangxi Zhuang Autonomous Region | Hezhou Xinhao Agriculture and Animal Husbandry Co., Ltd. | Wanggao Second farm | Sow farm | 8 | 336 | 17 |
| Guangxi Zhuang Autonomous Region | Hezhou Xinhao Agriculture and Animal Husbandry Co., Ltd. | Wanggao First farm | Sow farm | 8 | 336 | 9 |
| Guangxi Zhuang Autonomous Region | Hezhou Xinhao Agriculture and Animal Husbandry Co., Ltd. | Wanggao Third farm | Sow farm | 8 | 336 | 27 |
| Guangxi Zhuang Autonomous Region | Hezhou Xinhao Agriculture and Animal Husbandry Co., Ltd. | Wanggao Fourth farm | Sow farm | 8 | 336 | 21 |
| Guangxi Zhuang Autonomous Region | Hezhou Xinhao Agriculture and Animal Husbandry Co., Ltd. | Wanggao Fifth farm | Sow farm | 8 | 336 | 19 |
| Guangxi Zhuang Autonomous Region | Laibin Xinhao Agriculture and Animal Husbandry Co., Ltd. | Liaoping North District Second farm | Sow farm | 8 | 336 | 23 |
| Guangxi Zhuang Autonomous Region | Laibin Xinhao Agriculture and Animal Husbandry Co., Ltd. | Liaoping North District Sixth farm | Sow farm | 8 | 336 | 27 |
| Guangxi Zhuang Autonomous Region | Laibin Xinhao Agriculture and Animal Husbandry Co., Ltd. | Liaoping North District Third farm | Sow farm | 8 | 336 | 31 |
| Guangxi Zhuang Autonomous Region | Laibin Xinhao Agriculture and Animal Husbandry Co., Ltd. | Liaoping North District Fourth farm | Sow farm | 8 | 336 | 9 |
| Guangxi Zhuang Autonomous Region | Laibin Xinhao Agriculture and Animal Husbandry Co., Ltd. | Liaoping North District Fifth farm | Sow farm | 8 | 336 | 13 |
| Guangxi Zhuang Autonomous Region | Laibin Xinhao Agriculture and Animal Husbandry Co., Ltd. | Liaoping North District First farm | Sow farm | 8 | 336 | 11 |
| Guangxi Zhuang Autonomous Region | Laibin Xinhao Agriculture and Animal Husbandry Co., Ltd. | Liaoping South District Second farm | Sow farm | 8 | 336 | 29 |
| Guangxi Zhuang Autonomous Region | Laibin Xinhao Agriculture and Animal Husbandry Co., Ltd. | Liaoping South District First farm | Sow farm | 8 | 336 | 14 |
| Guangxi Zhuang Autonomous Region | Laibin Xinhao Agriculture and Animal Husbandry Co., Ltd. | Liaoping Breeding Pig farm North District Sow farm | Sow farm | 8 | 336 | 15 |
| Guangxi Zhuang Autonomous Region | Laibin Xinhao Agriculture and Animal Husbandry Co., Ltd. | Liaoping Breeding Pig farm South District Sow farm | Sow farm | 8 | 336 | 17 |
| Guangxi Zhuang Autonomous Region | Laibin Xinhao Agriculture and Animal Husbandry Co., Ltd. Breeding farm | Liaoping Ancestral farm | Sow farm | 8 | 336 | 32 |
| Guangxi Zhuang Autonomous Region | Laibin New Hope Liuhe Agriculture and Animal Husbandry Technology Co., Ltd. | Fenghuang Second farm | Sow farm | 8 | 336 | 12 |
| Guangxi Zhuang Autonomous Region | Laibin New Hope Liuhe Agriculture and Animal Husbandry Technology Co., Ltd. | Fenghuang Third farm | Sow farm | 8 | 336 | 19 |
| Guangxi Zhuang Autonomous Region | Laibin New Hope Liuhe Agriculture and Animal Husbandry Technology Co., Ltd. | Fenghuang First farm | Sow farm | 8 | 336 | 28 |
| Guangxi Zhuang Autonomous Region | Liuzhou Xinliu Agriculture and Animal Husbandry Technology Co., Ltd. | Liuzhou Liushan Second farm | Sow farm | 8 | 336 | 22 |
| Guangxi Zhuang Autonomous Region | Liuzhou Xinliu Agriculture and Animal Husbandry Technology Co., Ltd. | Liuzhou Liushan Third farm | Sow farm | 8 | 336 | 24 |
| Guangxi Zhuang Autonomous Region | Liuzhou Xinliu Agriculture and Animal Husbandry Technology Co., Ltd. | Liuzhou Liushan First farm | Sow farm | 8 | 336 | 27 |
| Guangxi Zhuang Autonomous Region | Longzhou Xinhao Agriculture and Animal Husbandry Co., Ltd. | Xiangshui First farm | Sow farm | 8 | 336 | 42 |
| Guangxi Zhuang Autonomous Region | Longzhou Xinhao Agriculture and Animal Husbandry Co., Ltd. | Xiangshui Second farm | Sow farm | 8 | 336 | 23 |
| Guangxi Zhuang Autonomous Region | Longzhou Xinhao Agriculture and Animal Husbandry Co., Ltd. | Xiangshui Sixth farm | Sow farm | 8 | 336 | 20 |
| Guangxi Zhuang Autonomous Region | Longzhou Xinhao Agriculture and Animal Husbandry Co., Ltd. | Xiangshui Seventh farm | Sow farm | 8 | 336 | 7 |
| Guangxi Zhuang Autonomous Region | Longzhou Xinhao Agriculture and Animal Husbandry Co., Ltd. | Xiangshui Third farm | Sow farm | 8 | 336 | 27 |
| Guangxi Zhuang Autonomous Region | Longzhou Xinhao Agriculture and Animal Husbandry Co., Ltd. | Xiangshui Fourth farm | Sow farm | 8 | 336 | 29 |
| Guangxi Zhuang Autonomous Region | Longzhou Xinhao Agriculture and Animal Husbandry Co., Ltd. | Xiangshui Fifth farm | Sow farm | 8 | 336 | 14 |
| Guangxi Zhuang Autonomous Region | Longzhou Xinhao Agriculture and Animal Husbandry Co., Ltd. | Xiangshui Boar Station | Sow farm | 8 | 336 | 31 |
| Guangxi Zhuang Autonomous Region | Nanning Wuming District Xinliu Agriculture and Animal Husbandry Technology Co., Ltd. | Chuanqian Pig farm | Sow farm | 8 | 336 | 29 |
| Guangxi Zhuang Autonomous Region | Nanning Xinhao Agriculture and Animal Husbandry Co., Ltd. | Tongliang Fifth farm | Sow farm | 8 | 336 | 16 |
| Guangxi Zhuang Autonomous Region | Nanning Xinhao Agriculture and Animal Husbandry Co., Ltd. | Tongliang Second farm | Sow farm | 8 | 336 | 15 |
| Guangxi Zhuang Autonomous Region | Nanning Xinhao Agriculture and Animal Husbandry Co., Ltd. | Tongliang Third farm | Sow farm | 8 | 336 | 33 |
| Guangxi Zhuang Autonomous Region | Nanning Xinhao Agriculture and Animal Husbandry Co., Ltd. | Tongliang Fourth farm | Sow farm | 8 | 336 | 31 |
| Guangxi Zhuang Autonomous Region | Nanning Xinhao Agriculture and Animal Husbandry Co., Ltd. | Tongliang First farm | Sow farm | 8 | 336 | 54 |
| Guangxi Zhuang Autonomous Region | Nanning Xinliu Agriculture and Animal Husbandry Technology Co., Ltd. | Xisheng Ancestral farm | Sow farm | 8 | 336 | 29 |
| Guangxi Zhuang Autonomous Region | Nanning Xinliu Agriculture and Animal Husbandry Technology Co., Ltd. | Xisheng Second farm | Sow farm | 8 | 336 | 11 |
| Guangxi Zhuang Autonomous Region | Nanning Xinliu Agriculture and Animal Husbandry Technology Co., Ltd. | Xisheng First farm | Sow farm | 8 | 336 | 17 |
| Guangxi Zhuang Autonomous Region | Ningming Xinhao Agriculture and Animal Husbandry Co., Ltd. | Tingliang Seventh farm | Sow farm | 8 | 336 | 11 |
| Guangxi Zhuang Autonomous Region | Ningming Xinhao Agriculture and Animal Husbandry Co., Ltd. | Tingliang Second farm | Sow farm | 8 | 336 | 37 |
| Guangxi Zhuang Autonomous Region | Ningming Xinhao Agriculture and Animal Husbandry Co., Ltd. | Tingliang Sixth farm | Sow farm | 8 | 336 | 18 |
| Guangxi Zhuang Autonomous Region | Ningming Xinhao Agriculture and Animal Husbandry Co., Ltd. | Tingliang Third farm | Sow farm | 8 | 336 | 10 |
| Guangxi Zhuang Autonomous Region | Ningming Xinhao Agriculture and Animal Husbandry Co., Ltd. | Tingliang Fourth farm | Sow farm | 8 | 336 | 21 |
| Guangxi Zhuang Autonomous Region | Ningming Xinhao Agriculture and Animal Husbandry Co., Ltd. | Tingliang Fifth farm | Sow farm | 8 | 336 | 29 |
| Guangxi Zhuang Autonomous Region | Ningming Xinhao Agriculture and Animal Husbandry Co., Ltd. | Tingliang First farm | Sow farm | 8 | 336 | 12 |
| Guangxi Zhuang Autonomous Region | Ningming Xinhao Agriculture and Animal Husbandry Co., Ltd. | Tingliang Boar Station | Sow farm | 8 | 336 | 17 |
| Guangxi Zhuang Autonomous Region | Xiangzhou Xinhao Agriculture and Animal Husbandry Co., Ltd. | Damon Fifth farm | Sow farm | 8 | 336 | 31 |
| Guangxi Zhuang Autonomous Region | Xiangzhou Xinhao Agriculture and Animal Husbandry Co., Ltd. | Damon Second farm | Sow farm | 8 | 336 | 19 |
| Guangxi Zhuang Autonomous Region | Xiangzhou Xinhao Agriculture and Animal Husbandry Co., Ltd. | Damon Third farm | Sow farm | 8 | 336 | 10 |
| Guangxi Zhuang Autonomous Region | Xiangzhou Xinhao Agriculture and Animal Husbandry Co., Ltd. | Damon Fourth farm | Sow farm | 8 | 336 | 21 |
| Guangxi Zhuang Autonomous Region | Xiangzhou Xinhao Agriculture and Animal Husbandry Co., Ltd. | Damon First farm | Sow farm | 8 | 336 | 31 |
| Guangxi Zhuang Autonomous Region | Guigang Xinliu Agriculture and Animal Husbandry Technology Co., Ltd. | Jiahe Pig farm | Fattening farm | 8 | 336 | 38 |
| Guangxi Zhuang Autonomous Region | Guilin Xinhao Agriculture and Animal Husbandry Technology Co., Ltd. | Lingui Service Department (Guilin Agriculture and Animal Husbandry) | Fattening farm | 8 | 336 | 16 |
| Guangxi Zhuang Autonomous Region | Jiexi New Hope Liuhe Breeding Co., Ltd. | Jieyang Xiyang Pig farm | Fattening farm | 8 | 336 | 11 |
| Guangxi Zhuang Autonomous Region | Laibin Xinhao Agriculture and Animal Husbandry Co., Ltd. | Chenliu Second farm | Fattening farm | 8 | 336 | 33 |
| Guangxi Zhuang Autonomous Region | Laibin Xinhao Agriculture and Animal Husbandry Co., Ltd. | Chenliu Third farm | Fattening farm | 8 | 336 | 39 |
| Guangxi Zhuang Autonomous Region | Laibin Xinhao Agriculture and Animal Husbandry Co., Ltd. | Chenliu Fourth farm | Fattening farm | 8 | 336 | 42 |
| Guangxi Zhuang Autonomous Region | Laibin Xinhao Agriculture and Animal Husbandry Co., Ltd. | Chenliu Fifth farm | Fattening farm | 8 | 336 | 17 |
| Guangxi Zhuang Autonomous Region | Laibin Xinhao Agriculture and Animal Husbandry Co., Ltd. | Chenliu First farm | Fattening farm | 8 | 336 | 21 |
| Guangxi Zhuang Autonomous Region | Laibin Xinhao Agriculture and Animal Husbandry Co., Ltd. | Laibin Fanghe Pig farm | Fattening farm | 8 | 336 | 39 |
| Guangxi Zhuang Autonomous Region | Laibin Xinhao Agriculture and Animal Husbandry Co., Ltd. | Chenliu Sixth farm | Fattening farm | 8 | 336 | 31 |
| Guangxi Zhuang Autonomous Region | Laibin Xinhao Agriculture and Animal Husbandry Co., Ltd. | Laibin Liancai Pig farm | Fattening farm | 8 | 336 | 21 |
| Guangxi Zhuang Autonomous Region | Laibin Xinhao Agriculture and Animal Husbandry Co., Ltd. | Laibin Paiku Pig farm | Fattening farm | 8 | 336 | 21 |
| Guangxi Zhuang Autonomous Region | Laibin Xinliu Agriculture and Animal Husbandry Co., Ltd. | Pingguo Service Department | Fattening farm | 8 | 336 | 13 |
| Guangxi Zhuang Autonomous Region | Lipu Xinhao Agriculture and Animal Husbandry Technology Co., Ltd. | Lipu Qingshuitang Second farm | Fattening farm | 8 | 336 | 18 |
| Guangxi Zhuang Autonomous Region | Lipu Xinhao Agriculture and Animal Husbandry Technology Co., Ltd. | Lipu Qingshuitang Third farm | Fattening farm | 8 | 336 | 31 |
| Guangxi Zhuang Autonomous Region | Lipu Xinhao Agriculture and Animal Husbandry Technology Co., Ltd. | Lipu Qingshuitang Fourth farm | Fattening farm | 8 | 336 | 27 |
| Guangxi Zhuang Autonomous Region | Lipu Xinhao Agriculture and Animal Husbandry Technology Co., Ltd. | Lipu Qingshuitang Fifth farm | Fattening farm | 8 | 336 | 30 |
| Guangxi Zhuang Autonomous Region | Lipu Xinhao Agriculture and Animal Husbandry Technology Co., Ltd. | Lipu Qingshuitang First farm | Fattening farm | 8 | 336 | 33 |
| Guangxi Zhuang Autonomous Region | Lipu Xinhao Agriculture and Animal Husbandry Technology Co., Ltd. | Lipu Qingshuitang Pig farm | Fattening farm | 8 | 336 | 19 |
| Guangxi Zhuang Autonomous Region | Longzhou Xinhao Agriculture and Animal Husbandry Co., Ltd. | Xiangshui Fattening Second farm | Fattening farm | 8 | 336 | 52 |
| Guangxi Zhuang Autonomous Region | Longzhou Xinhao Agriculture and Animal Husbandry Co., Ltd. | Xiangshui Fattening Sixth farm | Fattening farm | 8 | 336 | 34 |
| Guangxi Zhuang Autonomous Region | Longzhou Xinhao Agriculture and Animal Husbandry Co., Ltd. | Xiangshui Fattening Third farm | Fattening farm | 8 | 336 | 31 |
| Guangxi Zhuang Autonomous Region | Longzhou Xinhao Agriculture and Animal Husbandry Co., Ltd. | Xiangshui Fattening First farm | Fattening farm | 8 | 336 | 17 |
| Guangxi Zhuang Autonomous Region | Nanning Wuming District Xinliu Agriculture and Animal Husbandry Technology Co., Ltd. | Xinliu Liuzhou Service Department (Wuming Agriculture and Animal Husbandry) | Fattening farm | 8 | 336 | 10 |
| Guangxi Zhuang Autonomous Region | Xiangzhou Xinhao Agriculture and Animal Husbandry Co., Ltd. | Heren Fattening First farm | Fattening farm | 8 | 336 | 22 |
| Guangxi Zhuang Autonomous Region | Xiangzhou Xinhao Agriculture and Animal Husbandry Co., Ltd. | Xiangzhou Mumen Pig farm | Fattening farm | 8 | 336 | 42 |
| Guangxi Zhuang Autonomous Region | Yulin Xinhao Agriculture and Animal Husbandry Technology Co., Ltd. | Lingshan Service Department (Yulin Agriculture and Animal Husbandry) | Fattening farm | 8 | 336 | 39 |
| Guizhou Province | Bijie Xinliu Agriculture and Animal Husbandry Co., Ltd. | Bijie Xinliu First farm | Sow farm | 8 | 336 | 32 |
| Guizhou Province | Bijie Xinliu Agriculture and Animal Husbandry Co., Ltd. | Bijie Xinliu Second farm | Sow farm | 8 | 336 | 36 |
| Guizhou Province | Guanling Xinmu Breeding Co., Ltd. | Kangzhai Parent Generation First farm | Sow farm | 8 | 336 | 31 |
| Guizhou Province | Guanling Xinmu Breeding Co., Ltd. | Kangzhai First farm Second farm | Sow farm | 8 | 336 | 39 |
| Guizhou Province | Guanling Xinmu Breeding Co., Ltd. | Kangzhai First farm First farm | Sow farm | 8 | 336 | 58 |
| Guizhou Province | Guanling Xinmu Breeding Co., Ltd. | Kangzhai Sow farm First farm First farm | Sow farm | 8 | 336 | 46 |
| Guizhou Province | Shibing County New Hope Liuhe Breeding Co., Ltd. | Shibing Phase II Parent Generation farm | Sow farm | 8 | 336 | 49 |
| Guizhou Province | Shibing County New Hope Liuhe Breeding Co., Ltd. | Shibing First farm | Sow farm | 8 | 336 | 20 |
| Guizhou Province | Shibing County New Hope Liuhe Breeding Co., Ltd. | Shibing Phase II Second farm | Sow farm | 8 | 336 | 33 |
| Guizhou Province | Shibing County New Hope Liuhe Breeding Co., Ltd. | Shibing Phase II Third farm | Sow farm | 8 | 336 | 42 |
| Guizhou Province | Shibing County New Hope Liuhe Breeding Co., Ltd. | Shibing Phase II First farm | Sow farm | 8 | 336 | 57 |
| Guizhou Province | Shibing County New Hope Liuhe Breeding Co., Ltd. | Shibing Second farm | Sow farm | 8 | 336 | 30 |
| Guizhou Province | Xingren Xinliu Agricultural and Animal Husbandry Technology Co., Ltd. | Xingren Panjiazhuang Parent Generation farm | Sow farm | 8 | 336 | 49 |
| Guizhou Province | Xingren Xinliu Agricultural and Animal Husbandry Technology Co., Ltd. | Xingren Panjiazhuang Parent Stock Second farm | Sow farm | 8 | 336 | 33 |
| Guizhou Province | Xingren Xinliu Agricultural and Animal Husbandry Technology Co., Ltd. | Xingren Panjiazhuang Parent Stock First farm | Sow farm | 8 | 336 | 50 |
| Guizhou Province | Xingren Xinliu Agricultural and Animal Husbandry Technology Co., Ltd. | Xingren Panjiazhuang Second farm | Sow farm | 8 | 336 | 49 |
| Guizhou Province | Xingren Xinliu Agricultural and Animal Husbandry Technology Co., Ltd. | Xingren Panjiazhuang First farm | Sow farm | 8 | 336 | 57 |
| Guizhou Province | Guangling New Pasture Breeding Co., Ltd. | Guanling Xinzhai Pig farm | Fattening farm | 8 | 336 | 62 |
| Guizhou Province | Guangling New Pasture Breeding Co., Ltd. | Yongmu Pig farm | Fattening farm | 8 | 336 | 35 |
| Guizhou Province | Guangling New Pasture Breeding Co., Ltd. | Yongyao Pig farm | Fattening farm | 8 | 336 | 29 |
| Guizhou Province | Guizhou New Hope Liuhe Breeding Co., Ltd. | Guji Pig farm | Fattening farm | 8 | 336 | 42 |
| Guizhou Province | Panzhou Xinliu Agricultural and Animal Husbandry Development Co., Ltd. | Panzhou Jiuying First farm | Fattening farm | 8 | 336 | 11 |
| Guizhou Province | Shibing County New Hope Liuhe Breeding Co., Ltd. | Shibing Guxi First farm | Fattening farm | 8 | 336 | 49 |
| Guizhou Province | Shibing County New Hope Liuhe Breeding Co., Ltd. | Phase I Third farm First farm | Fattening farm | 8 | 336 | 53 |
| Guizhou Province | Shibing County New Hope Liuhe Breeding Co., Ltd. | Shibing Baiyanlong | Fattening farm | 8 | 336 | 28 |
| Guizhou Province | Zhenyuan New Pasture Agricultural Development Co., Ltd. | Shuangba Second farm | Fattening farm | 8 | 336 | 19 |
| Guizhou Province | Zhenyuan New Pasture Agricultural Development Co., Ltd. | Shuangba Sixth farm | Fattening farm | 8 | 336 | 30 |
| Guizhou Province | Zhenyuan New Pasture Agricultural Development Co., Ltd. | Shuangba Fifth farm | Fattening farm | 8 | 336 | 44 |
| Guizhou Province | Zhenyuan New Pasture Agricultural Development Co., Ltd. | Shuangba First farm | Fattening farm | 8 | 336 | 47 |
| Hainan Province | Hainan Changjiang Xinliu Breeding Co., Ltd. | Changjiang Parent Generation Pig farm | Sow farm | 8 | 336 | 22 |
| Hainan Province | Hainan Changjiang Xinliu Breeding Co., Ltd. | Changjiang Baoping Second farm | Sow farm | 8 | 336 | 10 |
| Hainan Province | Hainan Changjiang Xinliu Breeding Co., Ltd. | Changjiang Baoping First farm | Sow farm | 8 | 336 | 32 |
| Hainan Province | Hainan Lingao Xinliu Breeding Co., Ltd. | Lingao Binglian Second farm | Sow farm | 8 | 336 | 55 |
| Hainan Province | Hainan Lingao Xinliu Breeding Co., Ltd. | Lingao Binglian First farm | Sow farm | 8 | 336 | 49 |
| Hainan Province | Hainan Lingao Xinliu Breeding Co., Ltd. | Lingao Binglian Third farm | Sow farm | 8 | 336 | 21 |
| Hainan Province | Hainan Xinliu Agricultural and Animal Husbandry Technology Co., Ltd. | Haikou Hongming Parent Generation farm | Sow farm | 8 | 336 | 69 |
| Hainan Province | Hainan Xinliu Agricultural and Animal Husbandry Technology Co., Ltd. | Haikou Hongming Second farm | Sow farm | 8 | 336 | 53 |
| Hainan Province | Hainan Xinliu Agricultural and Animal Husbandry Technology Co., Ltd. | Haikou Hongming First farm | Sow farm | 8 | 336 | 37 |
| Hainan Province | Hainan Lingao Xinliu Breeding Co., Ltd. | Binglian Quick farm | Fattening farm | 8 | 336 | 33 |
| Hainan Province | Hainan Lingao Xinliu Breeding Co., Ltd. | Qiancai Fattening Second farm | Fattening farm | 8 | 336 | 11 |
| Hainan Province | Hainan Xinliu Agricultural and Animal Husbandry Technology Co., Ltd. | Hongming Fattening Second farm | Fattening farm | 8 | 336 | 17 |
| Hainan Province | Hainan Xinliu Agricultural and Animal Husbandry Technology Co., Ltd. | Hongming Fattening Third farm | Fattening farm | 8 | 336 | 29 |
| Hainan Province | Hainan Xinliu Agricultural and Animal Husbandry Technology Co., Ltd. | Hongming Fattening Fourth farm | Fattening farm | 8 | 336 | 32 |
| Hainan Province | Hainan Xinliu Agricultural and Animal Husbandry Technology Co., Ltd. | Hongming Fattening Fifth farm | Fattening farm | 8 | 336 | 10 |
| Hainan Province | Hainan Xinliu Agricultural and Animal Husbandry Technology Co., Ltd. | Hongming Fattening First farm | Fattening farm | 8 | 336 | 19 |
| Hainan Province | Hainan New Hope Agriculture Co., Ltd. | West farm Free Range Service Department | Fattening farm | 8 | 336 | 44 |
| Hebei Province | Dingzhou Xinhao Agricultural and Animal Husbandry Co., Ltd. | Xizhangqian First farm | Sow farm | 8 | 336 | 33 |
| Hebei Province | Dingzhou Xinhao Agricultural and Animal Husbandry Co., Ltd. | Xizhangqian Fourth farm | Sow farm | 8 | 336 | 79 |
| Hebei Province | Dingzhou Xinhao Agricultural and Animal Husbandry Co., Ltd. | Xizhangqian Fifth farm | Sow farm | 8 | 336 | 53 |
| Hebei Province | Hebei Xinhao Fucheng Agricultural Technology Co., Ltd. | Qianhuayuan First farm | Sow farm | 8 | 336 | 44 |
| Hebei Province | Hebei Xinhao Fucheng Agricultural Technology Co., Ltd. | Yangwutou First farm | Sow farm | 8 | 336 | 69 |
| Hebei Province | Hebei Xinhao Fucheng Agricultural Technology Co., Ltd. | Houhuayuan Second farm | Sow farm | 8 | 336 | 66 |
| Hebei Province | Hebei Xinhao Fucheng Agricultural Technology Co., Ltd. | Houhuayuan First farm | Sow farm | 8 | 336 | 21 |
| Hebei Province | Hebei Xinhao Fucheng Agricultural Technology Co., Ltd. | Yangwutou Second farm | Sow farm | 8 | 336 | 47 |
| Hebei Province | Huanghua Xinhao Technology Co., Ltd. | Liguanzhuang Fifth farm | Sow farm | 8 | 336 | 49 |
| Hebei Province | Huanghua Xinhao Technology Co., Ltd. | Liguanzhuang Second farm | Sow farm | 8 | 336 | 74 |
| Hebei Province | Huanghua Xinhao Technology Co., Ltd. | Liguanzhuang Third farm | Sow farm | 8 | 336 | 93 |
| Hebei Province | Huanghua Xinhao Technology Co., Ltd. | Liguanzhuang Fourth farm | Sow farm | 8 | 336 | 67 |
| Hebei Province | Huanghua Xinhao Technology Co., Ltd. | Liguanzhuang First farm | Sow farm | 8 | 336 | 43 |
| Hebei Province | Jingxian Xinhao Agricultural and Animal Husbandry Co., Ltd. | Xizhiyao Third farm | Sow farm | 8 | 336 | 19 |
| Hebei Province | Jingxian Xinhao Agricultural and Animal Husbandry Co., Ltd. | Xizhiyao Second farm | Sow farm | 8 | 336 | 67 |
| Hebei Province | Jingxian Xinhao Agricultural and Animal Husbandry Co., Ltd. | Xizhiyao First farm | Sow farm | 8 | 336 | 33 |
| Hebei Province | Raoyang Xinhao Agricultural and Animal Husbandry Co., Ltd. | Dongliuzhuang Third farm | Sow farm | 8 | 336 | 69 |
| Hebei Province | Raoyang Xinhao Agricultural and Animal Husbandry Co., Ltd. | Dongliuzhuang Second farm | Sow farm | 8 | 336 | 29 |
| Hebei Province | Raoyang Xinhao Agricultural and Animal Husbandry Co., Ltd. | Dongliuzhuang First farm | Sow farm | 8 | 336 | 28 |
| Hebei Province | Shenze County New Hope Liuhe Breeding Co., Ltd. | Daxing Second farm | Sow farm | 8 | 336 | 53 |
| Hebei Province | Shenze County New Hope Liuhe Breeding Co., Ltd. | Daxing Sixth farm | Sow farm | 8 | 336 | 89 |
| Hebei Province | Shenze County New Hope Liuhe Breeding Co., Ltd. | Daxing Third farm | Sow farm | 8 | 336 | 44 |
| Hebei Province | Shenze County New Hope Liuhe Breeding Co., Ltd. | Daxing Fourth farm | Sow farm | 8 | 336 | 41 |
| Hebei Province | Shenze County New Hope Liuhe Breeding Co., Ltd. | Daxing Fifth farm | Sow farm | 8 | 336 | 64 |
| Hebei Province | Shenze County New Hope Liuhe Breeding and Breeding Company | Daxing Breeding farm | Sow farm | 8 | 336 | 61 |
| Hebei Province | Tangshan Xinhao Agricultural and Animal Husbandry Co., Ltd. | Jiangjunzhuang Eighth farm | Sow farm | 8 | 336 | 53 |
| Hebei Province | Tangshan Xinhao Agricultural and Animal Husbandry Co., Ltd. | Jiangjunzhuang Seventh farm | Sow farm | 8 | 336 | 57 |
| Hebei Province | Tangshan Xinhao Agricultural and Animal Husbandry Co., Ltd. | Jiangjunzhuang Third farm | Sow farm | 8 | 336 | 11 |
| Hebei Province | Tangshan Xinhao Agricultural and Animal Husbandry Co., Ltd. | Jiangjunzhuang First farm | Sow farm | 8 | 336 | 63 |
| Hebei Province | Tianjin New Hope Liuhe Agricultural and Animal Husbandry Technology Co., Ltd. | Tianjin Red Star | Sow farm | 8 | 336 | 91 |
| Hebei Province | Zhangjiakou Xinwang Agricultural and Animal Husbandry Co., Ltd. | Futujiang Fifth farm | Sow farm | 8 | 336 | 39 |
| Hebei Province | Zhangjiakou Xinwang Agricultural and Animal Husbandry Co., Ltd. | Futujiang Second farm | Sow farm | 8 | 336 | 33 |
| Hebei Province | Zhangjiakou Xinwang Agricultural and Animal Husbandry Co., Ltd. | Futujiang Third farm | Sow farm | 8 | 336 | 81 |
| Hebei Province | Zhangjiakou Xinwang Agricultural and Animal Husbandry Co., Ltd. | Futujiang Fourth farm | Sow farm | 8 | 336 | 48 |
| Hebei Province | Zhangjiakou Xinwang Agricultural and Animal Husbandry Co., Ltd. | Futujiang First farm | Sow farm | 8 | 336 | 50 |
| Hebei Province | Dingzhou Xinhao Agricultural and Animal Husbandry Co., Ltd. | Xizhangqian Sixth farm | Fattening farm | 8 | 336 | 67 |
| Hebei Province | Fuping Xinliu Agricultural and Animal Husbandry Technology Co., Ltd. | Baijiayu Second farm | Fattening farm | 8 | 336 | 11 |
| Hebei Province | Fuping Xinliu Agricultural and Animal Husbandry Technology Co., Ltd. | Baijiayu Sixth farm | Fattening farm | 8 | 336 | 39 |
| Hebei Province | Fuping Xinliu Agricultural and Animal Husbandry Technology Co., Ltd. | Baijiayu Third farm | Fattening farm | 8 | 336 | 59 |
| Hebei Province | Fuping Xinliu Agricultural and Animal Husbandry Technology Co., Ltd. | Baijiayu Fourth farm | Fattening farm | 8 | 336 | 65 |
| Hebei Province | Fuping Xinliu Agricultural and Animal Husbandry Technology Co., Ltd. | Baijiayu Fifth farm | Fattening farm | 8 | 336 | 39 |
| Hebei Province | Fuping Xinliu Agricultural and Animal Husbandry Technology Co., Ltd. | Baijiayu First farm | Fattening farm | 8 | 336 | 23 |
| Hebei Province | Hebei Xinhao Fucheng Agricultural Technology Co., Ltd. | Pibotun Second farm | Fattening farm | 8 | 336 | 77 |
| Hebei Province | Hebei Xinhao Fucheng Agricultural Technology Co., Ltd. | Xindi Pig farm | Fattening farm | 8 | 336 | 47 |
| Hebei Province | Hengshui Jizhou Xinhao Agricultural and Animal Husbandry Co., Ltd. | Fujiazhuang First farm | Fattening farm | 8 | 336 | 39 |
| Hebei Province | Hengshui New Hope Liuhe Agricultural and Animal Husbandry Co., Ltd. | Gucheng Free Range Service Department | Fattening farm | 8 | 336 | 75 |
| Hebei Province | Huanghua Xinhao Technology Co., Ltd. | Liguanzhuang Ninth farm | Fattening farm | 8 | 336 | 43 |
| Hebei Province | Huanghua Xinhao Technology Co., Ltd. | Liguanzhuang Thirteenth farm | Fattening farm | 8 | 336 | 29 |
| Hebei Province | Nangong Xinhao Qianxihe Agricultural and Animal Husbandry Co., Ltd. | Gongjiawa Pig farm | Fattening farm | 8 | 336 | 63 |
| Hebei Province | Neiqiu Xinliu Agricultural and Animal Husbandry Technology Co., Ltd. | Neiqiu Wangjun Second farm | Fattening farm | 8 | 336 | 70 |
| Hebei Province | Raoyang Xinhao Agricultural and Animal Husbandry Co., Ltd. | Raoyang Dayuezhuang Pig farm | Fattening farm | 8 | 336 | 36 |
| Hebei Province | Raoyang Xinhao Agricultural and Animal Husbandry Co., Ltd. | Dongmazhong Pig farm Second farm | Fattening farm | 8 | 336 | 40 |
| Hebei Province | Raoyang Xinhao Agricultural and Animal Husbandry Co., Ltd. | Dongmazhong Pig farm First farm | Fattening farm | 8 | 336 | 48 |
| Hebei Province | Shenze County New Hope Liuhe Breeding Co., Ltd. | Shenze Lijiayuan Pig farm | Fattening farm | 8 | 336 | 52 |
| Hebei Province | Shenze County New Hope Liuhe Breeding Co., Ltd. | Shenze Nanxinzhuang Pig farm | Fattening farm | 8 | 336 | 51 |
| Hebei Province | Shenze County New Hope Liuhe Breeding Co., Ltd. | Shenze Xiaoli Pig farm | Fattening farm | 8 | 336 | 57 |
| Hebei Province | Xinji Xinliu Agricultural and Animal Husbandry Technology Co., Ltd. | Xinji Free-Ranging Service Department (Xinji Agriculture and Animal Husbandry) | Fattening farm | 8 | 336 | 39 |
| Henan Province | Anyang Xinliu Technology Co., Ltd. | Tangyin Parent Stock farm | Sow farm | 8 | 336 | 30 |
| Henan Province | Anyang Xinliu Technology Co., Ltd. | Tangyin Second farm | Sow farm | 8 | 336 | 25 |
| Henan Province | Anyang Xinliu Technology Co., Ltd. | Tangyin Third farm | Sow farm | 8 | 336 | 33 |
| Henan Province | Anyang Xinliu Technology Co., Ltd. | Tangyin First farm | Sow farm | 8 | 336 | 55 |
| Henan Province | Lingbao Xinliu Agriculture and Animal Husbandry Co., Ltd. | Lingbao Parent Stock farm | Sow farm | 8 | 336 | 62 |
| Henan Province | Lingbao Xinliu Agriculture and Animal Husbandry Co., Ltd. | Lingbao Parent Stock Second farm | Sow farm | 8 | 336 | 37 |
| Henan Province | Lingbao Xinliu Agriculture and Animal Husbandry Co., Ltd. | Lingbao Parent Stock First farm | Sow farm | 8 | 336 | 40 |
| Henan Province | Lingbao Xinliu Agriculture and Animal Husbandry Co., Ltd. | Lingbao Xinliu Sow farm | Sow farm | 8 | 336 | 19 |
| Henan Province | Qingfeng Xinliu Agriculture and Animal Husbandry Technology Co., Ltd. | Qingfeng Second farm | Sow farm | 8 | 336 | 46 |
| Henan Province | Qingfeng Xinliu Agriculture and Animal Husbandry Technology Co., Ltd. | Weishi County Reserve Breeding Third farm | Sow farm | 8 | 336 | 19 |
| Henan Province | Qingfeng Xinliu Agriculture and Animal Husbandry Technology Co., Ltd. | Qingfeng Third farm | Sow farm | 8 | 336 | 19 |
| Henan Province | Qingfeng Xinliu Agriculture and Animal Husbandry Technology Co., Ltd. | Qingfeng First farm | Sow farm | 8 | 336 | 50 |
| Henan Province | Ruzhou Quansheng Agriculture and Animal Husbandry Technology Co., Ltd. | Dongying Parent Stock Third farm | Sow farm | 8 | 336 | 37 |
| Henan Province | Ruzhou Quansheng Agriculture and Animal Husbandry Technology Co., Ltd. | Dongying Parent Stock First farm | Sow farm | 8 | 336 | 31 |
| Henan Province | Taian Xinliu Agriculture and Animal Husbandry Technology Co., Ltd. | Taian Parent Stock farm | Sow farm | 8 | 336 | 39 |
| Henan Province | Taian Xinliu Agriculture and Animal Husbandry Technology Co., Ltd. | Taian Second farm | Sow farm | 8 | 336 | 69 |
| Henan Province | Taian Xinliu Agriculture and Animal Husbandry Technology Co., Ltd. | Taian Third farm | Sow farm | 8 | 336 | 44 |
| Henan Province | Taian Xinliu Agriculture and Animal Husbandry Technology Co., Ltd. | Taian Fourth farm | Sow farm | 8 | 336 | 46 |
| Henan Province | Taian Xinliu Agriculture and Animal Husbandry Technology Co., Ltd. | Taian First farm | Sow farm | 8 | 336 | 82 |
| Henan Province | Taian City Xinchih Agriculture and Animal Husbandry Co., Ltd. | Weihui Dongsheng Pig farm | Sow farm | 8 | 336 | 37 |
| Henan Province | Taian City Xinchih Agriculture and Animal Husbandry Co., Ltd. | Changge Liangying Pig farm | Sow farm | 8 | 336 | 30 |
| Henan Province | Anyang Xinliu Technology Co., Ltd. | Bairui Reserve Sow farm | Fattening farm | 8 | 336 | 37 |
| Henan Province | Anyang Xinliu Technology Co., Ltd. | Qixian Bairui Animal Husbandry | Fattening farm | 8 | 336 | 37 |
| Henan Province | Anyang New Hope Liuhe Feed Co., Ltd. | Anyang Free-Ranging Service Department (Liuheli Feed) | Fattening farm | 8 | 336 | 78 |
| Henan Province | Henan New Hope Liuhe Agriculture and Animal Husbandry Technology Co., Ltd. | Huojia Pig farm | Fattening farm | 8 | 336 | 71 |
| Henan Province | Henan New Hope Liuhe Agriculture and Animal Husbandry Technology Co., Ltd. | Yufengyuan Pig farm | Fattening farm | 8 | 336 | 63 |
| Henan Province | Puyang Xinliu Agriculture and Animal Husbandry Technology Co., Ltd. | Huoying Second farm Second farm | Fattening farm | 8 | 336 | 47 |
| Henan Province | Puyang Xinliu Agriculture and Animal Husbandry Technology Co., Ltd. | Huoying Second farm Third farm | Fattening farm | 8 | 336 | 40 |
| Henan Province | Puyang Xinliu Agriculture and Animal Husbandry Technology Co., Ltd. | Huoying Second farm Fourth farm | Fattening farm | 8 | 336 | 54 |
| Henan Province | Puyang Xinliu Agriculture and Animal Husbandry Technology Co., Ltd. | Huoying Second farm First farm | Fattening farm | 8 | 336 | 37 |
| Henan Province | Puyang Xinliu Agriculture and Animal Husbandry Technology Co., Ltd. | Huoying First farm Third farm | Fattening farm | 8 | 336 | 32 |
| Henan Province | Puyang Xinliu Agriculture and Animal Husbandry Technology Co., Ltd. | Huoying First farm Fourth farm | Fattening farm | 8 | 336 | 13 |
| Henan Province | Puyang Xinliu Agriculture and Animal Husbandry Technology Co., Ltd. | Huoying First farm First farm | Fattening farm | 8 | 336 | 19 |
| Henan Province | Puyang Xinliu Agriculture and Animal Husbandry Technology Co., Ltd. | Qianfanzhai Third farm | Fattening farm | 8 | 336 | 20 |
| Henan Province | Puyang Xinliu Agriculture and Animal Husbandry Technology Co., Ltd. | Qianfanzhai Fourth farm | Fattening farm | 8 | 336 | 50 |
| Henan Province | Puyang Xinliu Agriculture and Animal Husbandry Technology Co., Ltd. | Qianfanzhai First farm | Fattening farm | 8 | 336 | 57 |
| Henan Province | Ruzhou Quansheng Agriculture and Animal Husbandry Technology Co., Ltd. | Beizhifang Second farm | Fattening farm | 8 | 336 | 42 |
| Henan Province | Ruzhou Quansheng Agriculture and Animal Husbandry Technology Co., Ltd. | Gucheng Fattening farm First farm | Fattening farm | 8 | 336 | 84 |
| Henan Province | Ruzhou Quansheng Agriculture and Animal Husbandry Technology Co., Ltd. | Gucheng Fattening farm Second farm | Fattening farm | 8 | 336 | 29 |
| Henan Province | Ruzhou Quansheng Agriculture and Animal Husbandry Technology Co., Ltd. | Gucheng Fattening farm Sixth farm | Fattening farm | 8 | 336 | 40 |
| Henan Province | Ruzhou Quansheng Agriculture and Animal Husbandry Technology Co., Ltd. | Gucheng Fattening farm Third farm | Fattening farm | 8 | 336 | 19 |
| Henan Province | Ruzhou Quansheng Agriculture and Animal Husbandry Technology Co., Ltd. | Gucheng Fattening farm Fourth farm | Fattening farm | 8 | 336 | 17 |
| Henan Province | Ruzhou Quansheng Agriculture and Animal Husbandry Technology Co., Ltd. | Gucheng Fattening farm Fifth farm | Fattening farm | 8 | 336 | 53 |
| Henan Province | Ruzhou Quansheng Agriculture and Animal Husbandry Technology Co., Ltd. | Dongying Parent Stock | Fattening farm | 8 | 336 | 63 |
| Henan Province | Taian City Xinchih Agriculture and Animal Husbandry Co., Ltd. | Weishi Reserve Breeding Second farm | Fattening farm | 8 | 336 | 12 |
| Henan Province | Taian City Xinchih Agriculture and Animal Husbandry Co., Ltd. | Weishi Reserve Breeding First farm | Fattening farm | 8 | 336 | 20 |
| Henan Province | Zhengzhou Quansheng Agriculture and Animal Husbandry Technology Co., Ltd. | Dengfeng Pig farm | Fattening farm | 8 | 336 | 43 |
| Heilongjiang Province | Yichun Xinwang Agriculture and Animal Husbandry Co., Ltd. | Yitiewang First farm | Sow farm | 8 | 336 | 53 |
| Heilongjiang Province | Yichun Xinwang Agriculture and Animal Husbandry Co., Ltd. | Yitiewang Parent Stock farm | Sow farm | 8 | 336 | 84 |
| Heilongjiang Province | Yichun Xinwang Agriculture and Animal Husbandry Co., Ltd. | Yitiewang First farm | Sow farm | 8 | 336 | 71 |
| Heilongjiang Province | Yichun Xinwang Agriculture and Animal Husbandry Co., Ltd. | Yitiewang Second farm | Sow farm | 8 | 336 | 90 |
| Heilongjiang Province | Yichun Xinwang Agriculture and Animal Husbandry Co., Ltd. | Yitiewang Second farm | Fattening farm | 8 | 336 | 51 |
| Heilongjiang Province | Yichun Xinwang Agriculture and Animal Husbandry Co., Ltd. | Yitiewang Third farm | Fattening farm | 8 | 336 | 54 |
| Heilongjiang Province | Yichun Xinwang Agriculture and Animal Husbandry Co., Ltd. | Yitiewang Fattening Second farm | Fattening farm | 8 | 336 | 47 |
| Heilongjiang Province | Yichun Xinwang Agriculture and Animal Husbandry Co., Ltd. | Yitiewang Fattening Third farm | Fattening farm | 8 | 336 | 89 |
| Heilongjiang Province | Yichun Xinwang Agriculture and Animal Husbandry Co., Ltd. | Yitiewang Fattening First farm | Fattening farm | 8 | 336 | 60 |
| Hubei Province | Hubei Xinhao Agriculture and Animal Husbandry Co., Ltd. | Guling Parent Stock farm | Sow farm | 8 | 336 | 70 |
| Hubei Province | Hubei Xinhao Agriculture and Animal Husbandry Co., Ltd. | Luohan Ninth farm | Sow farm | 8 | 336 | 67 |
| Hubei Province | Hubei Xinhao Agriculture and Animal Husbandry Co., Ltd. | Guling Second farm | Sow farm | 8 | 336 | 42 |
| Hubei Province | Hubei Xinhao Agriculture and Animal Husbandry Co., Ltd. | Guling Third farm | Sow farm | 8 | 336 | 49 |
| Hubei Province | Hubei Xinhao Agriculture and Animal Husbandry Co., Ltd. | Guling Fourth farm | Sow farm | 8 | 336 | 29 |
| Hubei Province | Hubei Xinhao Agriculture and Animal Husbandry Co., Ltd. | Guling First farm | Sow farm | 8 | 336 | 21 |
| Hubei Province | Hubei Xinhao Agriculture and Animal Husbandry Co., Ltd. | Luohan Eighth farm | Sow farm | 8 | 336 | 53 |
| Hubei Province | Hubei Xinhao Agriculture and Animal Husbandry Co., Ltd. | Luohan Second farm | Sow farm | 8 | 336 | 47 |
| Hubei Province | Hubei Xinhao Agriculture and Animal Husbandry Co., Ltd. | Luohan Sixth farm | Sow farm | 8 | 336 | 43 |
| Hubei Province | Hubei Xinhao Agriculture and Animal Husbandry Co., Ltd. | Luohan Fourth farm | Sow farm | 8 | 336 | 9 |
| Hubei Province | Hubei Xinhao Agriculture and Animal Husbandry Co., Ltd. | Luohan Fifth farm | Sow farm | 8 | 336 | 38 |
| Hubei Province | Hubei Xinhao Agriculture and Animal Husbandry Co., Ltd. | Luohan First farm | Sow farm | 8 | 336 | 65 |
| Hubei Province | Hubei Xinhao Agriculture and Animal Husbandry Co., Ltd. | Luohan Seventh farm | Sow farm | 8 | 336 | 37 |
| Hubei Province | Xiangyang Xinchih Agriculture and Animal Husbandry Co., Ltd. | Taiping Parent Stock | Sow farm | 8 | 336 | 49 |
| Hubei Province | Xiangyang Xinhao Agriculture and Animal Husbandry Co., Ltd. | Yanwan Third farm | Sow farm | 8 | 336 | 23 |
| Hubei Province | Xiangyang Xinhao Agriculture and Animal Husbandry Co., Ltd. | Xiangfan Cuiwan Pig farm | Sow farm | 8 | 336 | 62 |
| Hubei Province | Xiangyang Xinhao Agriculture and Animal Husbandry Co., Ltd. | Yanwan Second farm | Sow farm | 8 | 336 | 17 |
| Hubei Province | Xiangyang Xinhao Agriculture and Animal Husbandry Co., Ltd. | Yanwan Third farm | Fattening farm | 8 | 336 | 75 |
| Hubei Province | Xiangyang Xinhao Agriculture and Animal Husbandry Co., Ltd. | Yanwan Parent Stock | Fattening farm | 8 | 336 | 90 |
| Hubei Province | Xiangyang Xinhao Agriculture and Animal Husbandry Co., Ltd. | Yanwan Fourth farm | Fattening farm | 8 | 336 | 57 |
| Hubei Province | Yingcheng Xinhao Agriculture and Animal Husbandry Co., Ltd. | Xuzhou Seventh farm | Fattening farm | 8 | 336 | 30 |
| Hubei Province | Yingcheng Xinhao Agriculture and Animal Husbandry Co., Ltd. | Xuzhou Fourth farm | Fattening farm | 8 | 336 | 68 |
| Hubei Province | Yingcheng Xinhao Agriculture and Animal Husbandry Co., Ltd. | Xuzhou Second farm | Fattening farm | 8 | 336 | 56 |
| Hubei Province | Yingcheng Xinhao Agriculture and Animal Husbandry Co., Ltd. | Xuzhou Sixth farm | Fattening farm | 8 | 336 | 44 |
| Hubei Province | Yingcheng Xinhao Agriculture and Animal Husbandry Co., Ltd. | Xuzhou Third farm | Fattening farm | 8 | 336 | 29 |
| Hubei Province | Yingcheng Xinhao Agriculture and Animal Husbandry Co., Ltd. | Xuzhou Fifth farm | Fattening farm | 8 | 336 | 28 |
| Hubei Province | Yingcheng Xinhao Agriculture and Animal Husbandry Co., Ltd. | Xuzhou First farm | Fattening farm | 8 | 336 | 73 |
| Hubei Province | Hubei Xinhao Agriculture and Animal Husbandry Co., Ltd. | Huying Pig farm | Fattening farm | 8 | 336 | 10 |
| Hubei Province | Hubei Xinhao Agriculture and Animal Husbandry Co., Ltd. | Zaoyang Denglin Pig farm | Fattening farm | 8 | 336 | 27 |
| Hubei Province | Hubei Xinhao Agriculture and Animal Husbandry Co., Ltd. | Zaoyang Liangjia Pig farm | Fattening farm | 8 | 336 | 29 |
| Hubei Province | Xiangyang Xinhao Agriculture and Animal Husbandry Co., Ltd. | First Factory Pig farm | Fattening farm | 8 | 336 | 59 |
| Hubei Province | Xiangyang New Hope Agriculture and Animal Husbandry Co., Ltd. | Xiangfan Fuda Pig farm | Fattening farm | 8 | 336 | 42 |
| Hubei Province | Xiangyang New Hope Agriculture and Animal Husbandry Co., Ltd. | Xiangfan Heyuan Pig farm | Fattening farm | 8 | 336 | 14 |
| Hubei Province | Xiangyang New Hope Agriculture and Animal Husbandry Co., Ltd. | Xiangfan Tengyuan Pig farm | Fattening farm | 8 | 336 | 37 |
| Hubei Province | Yingcheng New Hope Agriculture and Animal Husbandry Co., Ltd. | Xuzhou Parent Stock Fattening | Fattening farm | 8 | 336 | 28 |
| Hubei Province | Yingcheng New Hope Agriculture and Animal Husbandry Co., Ltd. | Yingcheng Huilong Pig farm | Fattening farm | 8 | 336 | 19 |
| Hunan Province | Chenzhou New Hope Agriculture and Animal Husbandry Co., Ltd. | Cengtang First farm | Sow farm | 8 | 336 | 20 |
| Hunan Province | Chenzhou New Hope Agriculture and Animal Husbandry Co., Ltd. | Tonghe First farm | Sow farm | 8 | 336 | 19 |
| Hunan Province | Chenzhou New Hope Agriculture and Animal Husbandry Co., Ltd. | Cengtang Second farm | Sow farm | 8 | 336 | 27 |
| Hunan Province | Chenzhou New Hope Agriculture and Animal Husbandry Co., Ltd. | Cengtang Third farm | Sow farm | 8 | 336 | 6 |
| Hunan Province | Chenzhou New Hope Agriculture and Animal Husbandry Co., Ltd. | Cengtang Fourth farm | Sow farm | 8 | 336 | 15 |
| Hunan Province | Chenzhou New Hope Agriculture and Animal Husbandry Co., Ltd. | Tonghe Second farm | Sow farm | 8 | 336 | 54 |
| Hunan Province | Chenzhou New Hope Agriculture and Animal Husbandry Co., Ltd. | Tonghe Third farm | Sow farm | 8 | 336 | 27 |
| Hunan Province | Chenzhou New Hope Agriculture and Animal Husbandry Co., Ltd. | Tonghe Fourth farm | Sow farm | 8 | 336 | 19 |
| Hunan Province | Chenzhou New Hope Agriculture and Animal Husbandry Co., Ltd. | Tonghe Fifth farm | Sow farm | 8 | 336 | 30 |
| Hunan Province | Hengnan County Muyun Ecological Agriculture Co., Ltd. | Muyun Parent Stock First farm | Sow farm | 8 | 336 | 50 |
| Hunan Province | Hengnan County Muyun Ecological Agriculture Co., Ltd. | Muyun Parent Stock Second farm | Sow farm | 8 | 336 | 28 |
| Hunan Province | Hengnan County Muyun Ecological Agriculture Co., Ltd. | Muyun Parent Stock Third farm | Sow farm | 8 | 336 | 39 |
| Hunan Province | Longhui New Hope Liuhe Agriculture and Animal Husbandry Co., Ltd. | Biyunquan Parent Stock farm | Sow farm | 8 | 336 | 30 |
| Hunan Province | Chenzhou New Hope Agriculture and Animal Husbandry Co., Ltd. | Yongfeng Breeding farm | Fattening farm | 8 | 336 | 47 |
| Hunan Province | Guangdong New Hope Zhenghe Agriculture and Animal Husbandry Co., Ltd. | Lanshan Service Department (Guangdong Zhenghe) | Fattening farm | 8 | 336 | 45 |
| Hunan Province | Hengnan County Muyun Ecological Agriculture Co., Ltd. | Muyun Reserve farm | Fattening farm | 8 | 336 | 26 |
| Hunan Province | Qingyuan Xinhao Agriculture and Animal Husbandry Co., Ltd. | Qingyuan Daoxian Pig farm | Fattening farm | 8 | 336 | 27 |
| Hunan Province | Shaoyang County Newmu Agriculture and Animal Husbandry Co., Ltd. | Qiaotou Second farm | Fattening farm | 8 | 336 | 49 |
| Hunan Province | Shaoyang County Newmu Agriculture and Animal Husbandry Co., Ltd. | Qiaotou Third farm | Fattening farm | 8 | 336 | 38 |
| Hunan Province | Shaoyang County Newmu Agriculture and Animal Husbandry Co., Ltd. | Qiaotou First farm | Fattening farm | 8 | 336 | 38 |
| Hunan Province | Yongzhou New Hope Liuhe Feed Co., Ltd. | Yueyang Free-Ranging Service Department | Fattening farm | 8 | 336 | 37 |
| Hunan Province | Zhaoqing New Hope Agriculture and Animal Husbandry Co., Ltd. | Daoxian First farm | Fattening farm | 8 | 336 | 9 |
| Hunan Province | Zhaoqing New Hope Agriculture and Animal Husbandry Co., Ltd. | Daoxian Second farm | Fattening farm | 8 | 336 | 23 |
| Jiangsu Province | Donghai County New Hope Agriculture and Animal Husbandry Co., Ltd. | Donghai Fourth farm | Sow farm | 8 | 336 | 57 |
| Jiangsu Province | Donghai County New Hope Agriculture and Animal Husbandry Co., Ltd. | Donghai Second farm | Sow farm | 8 | 336 | 49 |
| Jiangsu Province | Donghai County New Hope Agriculture and Animal Husbandry Co., Ltd. | Donghai Third farm | Sow farm | 8 | 336 | 23 |
| Jiangsu Province | Donghai County New Hope Agriculture and Animal Husbandry Co., Ltd. | Donghai First farm | Sow farm | 8 | 336 | 43 |
| Jiangsu Province | Rugao Xinhao Agriculture and Animal Husbandry Co., Ltd. | Rugao First farm | Sow farm | 8 | 336 | 62 |
| Jiangsu Province | Rugao Xinhao Agriculture and Animal Husbandry Co., Ltd. | Zhenlong Pig farm | Sow farm | 8 | 336 | 35 |
| Jiangsu Province | Suining Xinliu Agriculture and Animal Husbandry Technology Co., Ltd. | Luwu Third farm | Sow farm | 8 | 336 | 69 |
| Jiangsu Province | Suining Xinliu Agriculture and Animal Husbandry Technology Co., Ltd. | Luwu Second farm | Sow farm | 8 | 336 | 52 |
| Jiangsu Province | Suining Xinliu Agriculture and Animal Husbandry Technology Co., Ltd. | Luwu First farm | Sow farm | 8 | 336 | 60 |
| Jiangsu Province | Taian City Xincheng Agriculture and Animal Husbandry Co., Ltd. | Huaiyin Baishui Pig farm | Sow farm | 8 | 336 | 40 |
| Jiangsu Province | Taian City Xincheng Agriculture and Animal Husbandry Co., Ltd. | Huaiyin Matou Pig farm | Sow farm | 8 | 336 | 27 |
| Jiangsu Province | Xuzhou Liuhe Longda Feed Co., Ltd. | Xuzhou Minggang Pig farm | Sow farm | 8 | 336 | 40 |
| Jiangsu Province | Yancheng Zhongtai Animal Husbandry Co., Ltd. | Dafeng Pig farm | Sow farm | 8 | 336 | 19 |
| Jiangsu Province | Zhenjiang New Hope Liuhe Agriculture and Animal Husbandry Co., Ltd. | Jurong Third farm | Sow farm | 8 | 336 | 72 |
| Jiangsu Province | Zhenjiang New Hope Liuhe Agriculture and Animal Husbandry Co., Ltd. | Jurong Second farm | Sow farm | 8 | 336 | 35 |
| Jiangsu Province | Zhenjiang New Hope Liuhe Agriculture and Animal Husbandry Co., Ltd. | Jurong First farm | Sow farm | 8 | 336 | 28 |
| Jiangsu Province | Donghai County New Hope Agriculture and Animal Husbandry Co., Ltd. | Donghai Breeding and Fattening | Fattening farm | 8 | 336 | 20 |
| Jiangsu Province | Nanjing Xinmu Agriculture and Animal Husbandry Co., Ltd. | Gaochun First farm | Fattening farm | 8 | 336 | 26 |
| Jiangsu Province | Suining Xinliu Agriculture and Animal Husbandry Technology Co., Ltd. | Lu Xu Sixth farm | Fattening farm | 8 | 336 | 25 |
| Jiangsu Province | Suining Xinliu Agriculture and Animal Husbandry Technology Co., Ltd. | Lu Xu Fourth farm | Fattening farm | 8 | 336 | 13 |
| Jiangsu Province | Suining Xinliu Agriculture and Animal Husbandry Technology Co., Ltd. | Lu Xu Fifth farm | Fattening farm | 8 | 336 | 24 |
| Jiangsu Province | Yancheng Zhongtai Animal Husbandry Co., Ltd. | Dafeng Free-Range Service Department | Fattening farm | 8 | 336 | 19 |
| Jiangsu Province | Zhenjiang New Hope Liuhe Agriculture and Animal Husbandry Co., Ltd. | Jurong Fattening | Fattening farm | 8 | 336 | 28 |
| Jiangxi Province | Ji'an City Xincheng Agriculture and Animal Husbandry Co., Ltd. | Nancheng Ririwang Pig farm | Sow farm | 8 | 336 | 60 |
| Jiangxi Province | Ji'an City Xincheng Agriculture and Animal Husbandry Co., Ltd. | Shunyuan Pig farm 01 | Sow farm | 8 | 336 | 15 |
| Jiangxi Province | Ji'an City Xincheng Agriculture and Animal Husbandry Co., Ltd. | Dayu Xiangfeng First farm | Sow farm | 8 | 336 | 17 |
| Jiangxi Province | Ji'an City Xincheng Agriculture and Animal Husbandry Co., Ltd. | Fenyi Qinzhou Pig farm | Sow farm | 8 | 336 | 43 |
| Jiangxi Province | Jiangxi New Hope Liuhe Agriculture and Animal Husbandry Technology Co., Ltd. | Jianshan Parent Stock farm | Sow farm | 8 | 336 | 40 |
| Jiangxi Province | Jiangxi New Hope Liuhe Agriculture and Animal Husbandry Technology Co., Ltd. | Jianshan Third farm | Sow farm | 8 | 336 | 29 |
| Jiangxi Province | Jiangxi New Hope Liuhe Agriculture and Animal Husbandry Technology Co., Ltd. | Jiangxi Breeding and Training Base | Sow farm | 8 | 336 | 26 |
| Jiangxi Province | Jiangxi New Hope Liuhe Agriculture and Animal Husbandry Technology Co., Ltd. | Jiangxi Breeding and Training Base Second farm | Sow farm | 8 | 336 | 21 |
| Jiangxi Province | Jiangxi New Hope Liuhe Agriculture and Animal Husbandry Technology Co., Ltd. | Jiangxi Breeding and Training Base Third farm | Sow farm | 8 | 336 | 47 |
| Jiangxi Province | Jiangxi New Hope Liuhe Agriculture and Animal Husbandry Technology Co., Ltd. | Jiangxi Breeding and Training Base Fourth farm | Sow farm | 8 | 336 | 49 |
| Jiangxi Province | Jiangxi New Hope Liuhe Agriculture and Animal Husbandry Technology Co., Ltd. | Jiangxi Breeding and Training Base First farm | Sow farm | 8 | 336 | 24 |
| Jiangxi Province | Taian City Xincheng Agriculture and Animal Husbandry Co., Ltd. | Xiangren Pig farm | Sow farm | 8 | 336 | 32 |
| Jiangxi Province | Taian City Xincheng Agriculture and Animal Husbandry Co., Ltd. | Jiangxi Heizhibao First farm | Sow farm | 8 | 336 | 38 |
| Jiangxi Province | Wannian County New Hope Liuhe Agriculture and Animal Husbandry Co., Ltd. | Shizhen Parent Stock farm | Sow farm | 8 | 336 | 33 |
| Jiangxi Province | Wannian County New Hope Liuhe Agriculture and Animal Husbandry Co., Ltd. | Shizhen Fattening Second farm | Sow farm | 8 | 336 | 29 |
| Jiangxi Province | Wannian County New Hope Liuhe Agriculture and Animal Husbandry Co., Ltd. | Shizhen First farm | Sow farm | 8 | 336 | 13 |
| Jiangxi Province | Xiajiang Xinliu Agriculture and Animal Husbandry Co., Ltd. | Xiajiang Pig farm | Sow farm | 8 | 336 | 28 |
| Jiangxi Province | Yingtan Xinliu Technology Co., Ltd. | Huangzhuang Parent Stock First farm First farm | Sow farm | 8 | 336 | 41 |
| Jiangxi Province | Yingtan Xinliu Technology Co., Ltd. | Huangzhuang Parent Stock First farm Second farm | Sow farm | 8 | 336 | 43 |
| Jiangxi Province | Yingtan Xinliu Technology Co., Ltd. | Huangzhuang Parent Stock First farm First farm | Sow farm | 8 | 336 | 29 |
| Jiangxi Province | Yingtan Xinliu Technology Co., Ltd. | Maquan Pig farm | Sow farm | 8 | 336 | 21 |
| Jiangxi Province | Yingtan Xinliu Technology Co., Ltd. | Yujiang Sow farm | Sow farm | 8 | 336 | 40 |
| Jiangxi Province | Jiangxi New Hope Liuhe Agriculture and Animal Husbandry Technology Co., Ltd. | Jianshan Fattening Second farm | Fattening farm | 8 | 336 | 73 |
| Jiangxi Province | Jiangxi New Hope Liuhe Agriculture and Animal Husbandry Technology Co., Ltd. | Jianshan Fattening Sixth farm | Fattening farm | 8 | 336 | 44 |
| Jiangxi Province | Jiangxi New Hope Liuhe Agriculture and Animal Husbandry Technology Co., Ltd. | Jianshan Fattening Fourth farm | Fattening farm | 8 | 336 | 48 |
| Jiangxi Province | Jiangxi New Hope Liuhe Agriculture and Animal Husbandry Technology Co., Ltd. | Jianshan Fattening Fifth farm | Fattening farm | 8 | 336 | 64 |
| Jiangxi Province | Jiangxi New Hope Liuhe Agriculture and Animal Husbandry Technology Co., Ltd. | Jianshan Fattening First farm | Fattening farm | 8 | 336 | 53 |
| Jiangxi Province | Jiangxi New Hope Liuhe Agriculture and Animal Husbandry Technology Co., Ltd. | Jianshan Fattening Third farm | Fattening farm | 8 | 336 | 39 |
| Jiangxi Province | Taian City Xincheng Agriculture and Animal Husbandry Co., Ltd. | Xinyu Tianhong Pig farm | Fattening farm | 8 | 336 | 47 |
| Jiangxi Province | Wannian County New Hope Liuhe Agriculture and Animal Husbandry Co., Ltd. | Lvong Breeding Base | Fattening farm | 8 | 336 | 19 |
| Jiangxi Province | Yingtan Xinliu Technology Co., Ltd. | Maquan Fattening Fourth farm | Fattening farm | 8 | 336 | 35 |
| Jiangxi Province | Yingtan Xinliu Technology Co., Ltd. | Maquan Fattening Fifth farm | Fattening farm | 8 | 336 | 32 |
| Jiangxi Province | Yingtan Xinliu Technology Co., Ltd. | Maquan Fattening Second farm | Fattening farm | 8 | 336 | 8 |
| Jiangxi Province | Yingtan Xinliu Technology Co., Ltd. | Maquan Fattening Third farm | Fattening farm | 8 | 336 | 20 |
| Jiangxi Province | Yingtan Xinliu Technology Co., Ltd. | Maquan Fattening First farm | Fattening farm | 8 | 336 | 35 |
| Liaoning Province | Fuxin Xinwang Animal Husbandry Co., Ltd. | Fulanwang Parent Stock farm | Sow farm | 8 | 336 | 55 |
| Liaoning Province | Fuxin Xinwang Animal Husbandry Co., Ltd. | Fulanwang Second farm | Sow farm | 8 | 336 | 54 |
| Liaoning Province | Fuxin Xinwang Animal Husbandry Co., Ltd. | Fulanwang Third farm | Sow farm | 8 | 336 | 29 |
| Liaoning Province | Fuxin Xinwang Animal Husbandry Co., Ltd. | Fulanwang Fourth farm | Sow farm | 8 | 336 | 67 |
| Liaoning Province | Fuxin Xinwang Animal Husbandry Co., Ltd. | Fulanwang First farm | Sow farm | 8 | 336 | 46 |
| Liaoning Province | Heishan Xinliu Agriculture and Animal Husbandry Technology Co., Ltd. | Ciyu Pig farm Fourth farm | Sow farm | 8 | 336 | 36 |
| Liaoning Province | Heishan Xinliu Agriculture and Animal Husbandry Technology Co., Ltd. | Woniu Pig farm Second farm | Sow farm | 8 | 336 | 38 |
| Liaoning Province | Heishan Xinliu Agriculture and Animal Husbandry Technology Co., Ltd. | Woniu Pig farm First farm | Sow farm | 8 | 336 | 29 |
| Liaoning Province | Heishan Xinliu Agriculture and Animal Husbandry Technology Co., Ltd. | Woniu Pig farm Parent Stock farm | Sow farm | 8 | 336 | 49 |
| Liaoning Province | Heishan Xinliu Agriculture and Animal Husbandry Technology Co., Ltd. | Gaojia Third farm | Sow farm | 8 | 336 | 47 |
| Liaoning Province | Heishan Xinliu Agriculture and Animal Husbandry Technology Co., Ltd. | Gaojia Fourth farm | Sow farm | 8 | 336 | 54 |
| Liaoning Province | Heishan Xinliu Agriculture and Animal Husbandry Technology Co., Ltd. | Gaojia First farm | Sow farm | 8 | 336 | 53 |
| Liaoning Province | Heishan Xinliu Agriculture and Animal Husbandry Technology Co., Ltd. | Gaojia Isolation farm | Sow farm | 8 | 336 | 66 |
| Liaoning Province | Heishan Xinliu Agriculture and Animal Husbandry Technology Co., Ltd. | Gaojia Parent Stock | Sow farm | 8 | 336 | 47 |
| Liaoning Province | Heishan Xinliu Agriculture and Animal Husbandry Technology Co., Ltd. | Woniu Parent Stock farm | Sow farm | 8 | 336 | 94 |
| Liaoning Province | Heishan Xinliu Agriculture and Animal Husbandry Technology Co., Ltd. | Woniu Second farm | Sow farm | 8 | 336 | 44 |
| Liaoning Province | Heishan Xinliu Agriculture and Animal Husbandry Technology Co., Ltd. | Woniu First farm | Sow farm | 8 | 336 | 51 |
| Liaoning Province | Kangping Xinwang Agriculture and Animal Husbandry Co., Ltd. | Woniu Isolation farm 1 | Sow farm | 8 | 336 | 44 |
| Liaoning Province | Kangping Xinwang Agriculture and Animal Husbandry Co., Ltd. | Kangbeiwang Parent Stock farm | Sow farm | 8 | 336 | 45 |
| Liaoning Province | Kangping Xinwang Agriculture and Animal Husbandry Co., Ltd. | Kangjiawang Parent Stock farm | Sow farm | 8 | 336 | 50 |
| Liaoning Province | Kangping Xinwang Agriculture and Animal Husbandry Co., Ltd. | Kangjiawang First farm | Sow farm | 8 | 336 | 30 |
| Liaoning Province | Kangping Xinwang Agriculture and Animal Husbandry Co., Ltd. | Kangbeiwang Second farm | Sow farm | 8 | 336 | 28 |
| Liaoning Province | Kangping Xinwang Agriculture and Animal Husbandry Co., Ltd. | Kangbeiwang Third farm | Sow farm | 8 | 336 | 60 |
| Liaoning Province | Kangping Xinwang Agriculture and Animal Husbandry Co., Ltd. | Kangbeiwang Fourth farm | Sow farm | 8 | 336 | 54 |
| Liaoning Province | Kangping Xinwang Agriculture and Animal Husbandry Co., Ltd. | Kangbeiwang First farm | Sow farm | 8 | 336 | 59 |
| Liaoning Province | Kangping Xinwang Agriculture and Animal Husbandry Co., Ltd. | Kangjiawang Second farm | Sow farm | 8 | 336 | 55 |
| Liaoning Province | Kangping Xinwang Agriculture and Animal Husbandry Co., Ltd. | Kangjiawang Third farm | Sow farm | 8 | 336 | 53 |
| Liaoning Province | Kangping Xinwang Agriculture and Animal Husbandry Co., Ltd. | Kangjiawang Fourth farm | Sow farm | 8 | 336 | 59 |
| Liaoning Province | Liaoning Xinwang Technology Co., Ltd. | Liaobei Diaobingshan Leased Sow farm | Sow farm | 8 | 336 | 39 |
| Liaoning Province | Liaoning Xinwang Technology Co., Ltd. | Mintun Parent Stock farm | Sow farm | 8 | 336 | 60 |
| Liaoning Province | Liaoning Xinwang Technology Co., Ltd. | Mintun Second farm | Sow farm | 8 | 336 | 44 |
| Liaoning Province | Liaoning Xinwang Technology Co., Ltd. | Mintun Third farm | Sow farm | 8 | 336 | 60 |
| Liaoning Province | Liaoning Xinwang Technology Co., Ltd. | Mintun Fourth farm | Sow farm | 8 | 336 | 69 |
| Liaoning Province | Liaoning Xinwang Technology Co., Ltd. | Mintun First farm | Sow farm | 8 | 336 | 44 |
| Liaoning Province | Liaoning Xinwang Technology Co., Ltd. | Mintun Boar Station | Sow farm | 8 | 336 | 17 |
| Liaoning Province | Liaoning Xinwang Technology Co., Ltd. | Taotun Eighth farm | Sow farm | 8 | 336 | 38 |
| Liaoning Province | Liaoning Xinwang Technology Co., Ltd. | Taotun Second farm | Sow farm | 8 | 336 | 50 |
| Liaoning Province | Liaoning Xinwang Technology Co., Ltd. | Taotun Sixth farm | Sow farm | 8 | 336 | 31 |
| Liaoning Province | Liaoning Xinwang Technology Co., Ltd. | Taotun Seventh farm | Sow farm | 8 | 336 | 29 |
| Liaoning Province | Liaoning Xinwang Technology Co., Ltd. | Taotun Third farm | Sow farm | 8 | 336 | 69 |
| Liaoning Province | Liaoning Xinwang Technology Co., Ltd. | Taotun Fourth farm | Sow farm | 8 | 336 | 54 |
| Liaoning Province | Liaoning Xinwang Technology Co., Ltd. | Taotun Fifth farm | Sow farm | 8 | 336 | 73 |
| Liaoning Province | Liaoning Xinwang Technology Co., Ltd. | Taotun First farm | Sow farm | 8 | 336 | 34 |
| Liaoning Province | Liaoning Xinwang Technology Co., Ltd. | Taotun Parent Generation farm | Sow farm | 8 | 336 | 63 |
| Liaoning Province | Liaoning Xinwang Technology Co., Ltd. | Taotun Parent Generation farm | Sow farm | 8 | 336 | 79 |
| Liaoning Province | Liaoning Xinwang Technology Co., Ltd. | Taotun Eighth farm | Sow farm | 8 | 336 | 26 |
| Liaoning Province | Liaoning Xinwang Technology Co., Ltd. | Taotun Seventh farm | Sow farm | 8 | 336 | 30 |
| Liaoning Province | Liaoning Xinwang Technology Co., Ltd. | Taotun Fifth farm | Sow farm | 8 | 336 | 53 |
| Liaoning Province | Liaoning Xinwang Technology Co., Ltd. | Taotun First farm | Sow farm | 8 | 336 | 58 |
| Liaoning Province | Liaoning Xinwang Technology Co., Ltd. | Taotun Second farm | Sow farm | 8 | 336 | 39 |
| Liaoning Province | Liaoning Xinwang Technology Co., Ltd. | Taotun Third farm | Sow farm | 8 | 336 | 44 |
| Liaoning Province | Liaoning Xinwang Food Co., Ltd. | Taotun Fourth farm | Sow farm | 8 | 336 | 27 |
| Liaoning Province | Zhangwu Xinwang Agriculture and Animal Husbandry Co., Ltd. | Fushun Hongye Lease farm | Sow farm | 8 | 336 | 12 |
| Liaoning Province | Zhangwu Xinwang Agriculture and Animal Husbandry Co., Ltd. | Zhangwei Parent Generation farm | Sow farm | 8 | 336 | 29 |
| Liaoning Province | Zhangwu Xinwang Agriculture and Animal Husbandry Co., Ltd. | Zhangxinwang Third farm | Sow farm | 8 | 336 | 20 |
| Liaoning Province | Zhangwu Xinwang Agriculture and Animal Husbandry Co., Ltd. | Zhangxinwang Parent Generation farm | Sow farm | 8 | 336 | 45 |
| Liaoning Province | Zhangwu Xinwang Agriculture and Animal Husbandry Co., Ltd. | Zhangxinwang Second farm | Sow farm | 8 | 336 | 49 |
| Liaoning Province | Zhangwu Xinwang Agriculture and Animal Husbandry Co., Ltd. | Zhangxinwang Fourth farm | Sow farm | 8 | 336 | 37 |
| Liaoning Province | Fuxin Xinwang Animal Husbandry Co., Ltd. | Zhangxinwang First farm | Sow farm | 8 | 336 | 34 |
| Liaoning Province | Fuxin Xinwang Animal Husbandry Co., Ltd. | Fulanwang Fourth farm | Fattening farm | 8 | 336 | 27 |
| Liaoning Province | Fuxin Xinwang Animal Husbandry Co., Ltd. | Fulanwang Fattening farm | Fattening farm | 8 | 336 | 30 |
| Liaoning Province | Heishan Xinliu Agriculture and Animal Husbandry Technology Co., Ltd. | Fulanwang Fattening farm Second | Fattening farm | 8 | 336 | 28 |
| Liaoning Province | Heishan Xinliu Agriculture and Animal Husbandry Technology Co., Ltd. | Chengbo Livestock farm | Fattening farm | 8 | 336 | 20 |
| Liaoning Province | Heishan Xinliu Agriculture and Animal Husbandry Technology Co., Ltd. | Ciyu Pig farm Third | Fattening farm | 8 | 336 | 55 |
| Liaoning Province | Heishan Xinliu Agriculture and Animal Husbandry Technology Co., Ltd. | Liuce Village Fattening farm | Fattening farm | 8 | 336 | 68 |
| Liaoning Province | Heishan Xinliu Agriculture and Animal Husbandry Technology Co., Ltd. | Woniupig Fattening farm | Fattening farm | 8 | 336 | 49 |
| Liaoning Province | Kangping Xinwang Agriculture and Animal Husbandry Co., Ltd. | Woni First farm | Fattening farm | 8 | 336 | 54 |
| Liaoning Province | Kangping Xinwang Agriculture and Animal Husbandry Co., Ltd. | Jiutun First farm | Fattening farm | 8 | 336 | 57 |
| Liaoning Province | Kangping Xinwang Agriculture and Animal Husbandry Co., Ltd. | Kangbawang First farm | Fattening farm | 8 | 336 | 72 |
| Liaoning Province | Kangping Xinwang Agriculture and Animal Husbandry Co., Ltd. | Kangerwang Second farm | Fattening farm | 8 | 336 | 31 |
| Liaoning Province | Kangping Xinwang Agriculture and Animal Husbandry Co., Ltd. | Kangerwang First farm | Fattening farm | 8 | 336 | 30 |
| Liaoning Province | Kangping Xinwang Agriculture and Animal Husbandry Co., Ltd. | Kangliuwang First farm | Fattening farm | 8 | 336 | 40 |
| Liaoning Province | Kangping Xinwang Agriculture and Animal Husbandry Co., Ltd. | Kangsanwang Fourth farm | Fattening farm | 8 | 336 | 45 |
| Liaoning Province | Kangping Xinwang Agriculture and Animal Husbandry Co., Ltd. | Kangsiwang First farm | Fattening farm | 8 | 336 | 34 |
| Liaoning Province | Kangping Xinwang Agriculture and Animal Husbandry Co., Ltd. | Yaolingangzi First farm | Fattening farm | 8 | 336 | 67 |
| Liaoning Province | Kangping Xinwang Agriculture and Animal Husbandry Co., Ltd. | Kangqiwang Fattening farm | Fattening farm | 8 | 336 | 21 |
| Liaoning Province | Kangping Xinwang Agriculture and Animal Husbandry Co., Ltd. | Kangsanwang Fattening farm | Fattening farm | 8 | 336 | 23 |
| Liaoning Province | Kangping Xinwang Agriculture and Animal Husbandry Co., Ltd. | Kangwuwang Fattening farm | Fattening farm | 8 | 336 | 31 |
| Liaoning Province | Liaoning Xinwang Technology Co., Ltd. | Kangyiwang Fattening farm | Fattening farm | 8 | 336 | 38 |
| Liaoning Province | Liaoning Xinwang Technology Co., Ltd. | Gongliu Second farm | Fattening farm | 8 | 336 | 54 |
| Liaoning Province | Liaoning Xinwang Technology Co., Ltd. | Gongliu First farm | Fattening farm | 8 | 336 | 30 |
| Liaoning Province | Liaoning Xinwang Technology Co., Ltd. | Liuji Fattening farm | Sow farm | 8 | 336 | 29 |
| Liaoning Province | Liaoning Xinwang Technology Co., Ltd. | Liuwei Fattening farm | Sow farm | 8 | 336 | 53 |
| Liaoning Province | Liaoning Xinwang Technology Co., Ltd. | Fangshen Second farm | Sow farm | 8 | 336 | 66 |
| Liaoning Province | Liaoning Xinwang Technology Co., Ltd. | Fangshen First farm | Sow farm | 8 | 336 | 72 |
| Liaoning Province | Liaoning Xinwang Technology Co., Ltd. | Dongwei Fattening farm | Sow farm | 8 | 336 | 40 |
| Liaoning Province | Liaoning Xinwang Technology Co., Ltd. | Fangshen Pig farm | Sow farm | 8 | 336 | 41 |
| Liaoning Province | Liaoning Xinwang Technology Co., Ltd. | Gongliu Fattening farm | Sow farm | 8 | 336 | 45 |
| Liaoning Province | Liaoning Xinwang Technology Co., Ltd. | Tao Hua Pig farm | Sow farm | 8 | 336 | 84 |
| Liaoning Province | Liaoning Xinwang Technology Co., Ltd. | Xinshuo Fattening farm | Sow farm | 8 | 336 | 47 |
| Liaoning Province | Liaoning Xinwang Technology Co., Ltd. | Zhou'an Fattening farm | Sow farm | 8 | 336 | 48 |
| Liaoning Province | Liaoning Xinwang Technology Co., Ltd. | Dongta Third farm | Sow farm | 8 | 336 | 20 |
| Liaoning Province | Liaoning Xinwang Technology Co., Ltd. | Dongta First farm | Sow farm | 8 | 336 | 33 |
| Liaoning Province | Liaoning Xinwang Food Co., Ltd. | Jinzhou Service Department | Sow farm | 8 | 336 | 19 |
| Liaoning Province | Zhangwu Xinwang Agriculture and Animal Husbandry Co., Ltd. | Zhangyiwang Fattening farm | Sow farm | 8 | 336 | 55 |
| Liaoning Province | Zhangwu Xinwang Agriculture and Animal Husbandry Co., Ltd. | Zhangerwang Fattening farm | Sow farm | 8 | 336 | 46 |
| Liaoning Province | Zhangwu Xinwang Agriculture and Animal Husbandry Co., Ltd. | Zhangqiwang Production Area | Fattening farm | 8 | 336 | 21 |
| Liaoning Province | Zhangwu Xinwang Agriculture and Animal Husbandry Co., Ltd. | Zhangsanwang Fattening farm | Fattening farm | 8 | 336 | 28 |
| Liaoning Province | Zhangwu Xinwang Agriculture and Animal Husbandry Co., Ltd. | Zhangshiwang Fattening farm | Fattening farm | 8 | 336 | 53 |
| Liaoning Province | Zhangwu Xinwang Agriculture and Animal Husbandry Co., Ltd. | Zhangsiwang Fattening farm | Fattening farm | 8 | 336 | 56 |
| Liaoning Province | Zhangwu Xinwang Agriculture and Animal Husbandry Co., Ltd. | Zhangweiwang Parent Generation farm | Fattening farm | 8 | 336 | 35 |
| Inner Mongolia Autonomous Region | Tongliao Xinhao Agriculture and Animal Husbandry Co., Ltd. | Tongwang Parent Generation Sixth farm | Sow farm | 8 | 336 | 45 |
| Inner Mongolia Autonomous Region | Tongliao Xinhao Agriculture and Animal Husbandry Co., Ltd. | Tongwang Parent Generation Eleventh farm | Sow farm | 8 | 336 | 59 |
| Inner Mongolia Autonomous Region | Tongliao Xinhao Agriculture and Animal Husbandry Co., Ltd. | Tongwang Ninth farm | Sow farm | 8 | 336 | 43 |
| Inner Mongolia Autonomous Region | Tongliao Xinhao Agriculture and Animal Husbandry Co., Ltd. | Tongwang Seventh farm | Sow farm | 8 | 336 | 31 |
| Inner Mongolia Autonomous Region | Tongliao Xinhao Agriculture and Animal Husbandry Co., Ltd. | Tongwang Twelfth farm | Sow farm | 8 | 336 | 20 |
| Inner Mongolia Autonomous Region | Tongliao Xinhao Agriculture and Animal Husbandry Co., Ltd. | Tongwang Thirteenth farm | Sow farm | 8 | 336 | 37 |
| Inner Mongolia Autonomous Region | Tongliao Xinhao Agriculture and Animal Husbandry Co., Ltd. | Tongwang Fourteenth farm | Sow farm | 8 | 336 | 52 |
| Inner Mongolia Autonomous Region | Tongliao Xinhao Agriculture and Animal Husbandry Co., Ltd. | Sanyitang Eighth farm | Sow farm | 8 | 336 | 20 |
| Inner Mongolia Autonomous Region | Tongliao Xinhao Agriculture and Animal Husbandry Co., Ltd. | Sanyitang Seventh farm | Sow farm | 8 | 336 | 74 |
| Inner Mongolia Autonomous Region | Tongliao Xinhao Agriculture and Animal Husbandry Co., Ltd. | Sanyitang Tenth farm | Sow farm | 8 | 336 | 51 |
| Inner Mongolia Autonomous Region | Tongliao Xinhao Agriculture and Animal Husbandry Co., Ltd. | Sanyitang First farm | Sow farm | 8 | 336 | 54 |
| Inner Mongolia Autonomous Region | Tongliao Xinhao Agriculture and Animal Husbandry Co., Ltd. | Tongwang Xinhao Agriculture and Animal Husbandry | Sow farm | 8 | 336 | 40 |
| Inner Mongolia Autonomous Region | Tongliao Xinhao Agriculture and Animal Husbandry Co., Ltd. | Tongwang Tenth farm | Sow farm | 8 | 336 | 29 |
| Inner Mongolia Autonomous Region | Tongliao Xinhao Agriculture and Animal Husbandry Co., Ltd. | Sanyitang Second farm | Sow farm | 8 | 336 | 18 |
| Inner Mongolia Autonomous Region | Tongliao Xinhao Agriculture and Animal Husbandry Co., Ltd. | Sanyitang Ninth farm | Sow farm | 8 | 336 | 25 |
| Inner Mongolia Autonomous Region | Tongliao Xinhao Agriculture and Animal Husbandry Co., Ltd. | Sanyitang Sixth farm | Sow farm | 8 | 336 | 62 |
| Inner Mongolia Autonomous Region | Tongliao Xinhao Agriculture and Animal Husbandry Co., Ltd. | Sanyitang Third farm | Sow farm | 8 | 336 | 19 |
| Inner Mongolia Autonomous Region | Tongliao Xinhao Agriculture and Animal Husbandry Co., Ltd. | Sanyitang Fourth farm | Sow farm | 8 | 336 | 39 |
| Inner Mongolia Autonomous Region | Tongliao Xinhao Agriculture and Animal Husbandry Co., Ltd. | Sanyitang Fifth farm | Sow farm | 8 | 336 | 14 |
| Inner Mongolia Autonomous Region | Tongliao Xinhao Agriculture and Animal Husbandry Co., Ltd. | Qianjuliuhe First farm | Fattening farm | 8 | 336 | 48 |
| Inner Mongolia Autonomous Region | Tongliao Xinhao Agriculture and Animal Husbandry Co., Ltd. | Tongliao Dalin Pig farm | Fattening farm | 8 | 336 | 37 |
| Inner Mongolia Autonomous Region | Tongliao Xinhao Agriculture and Animal Husbandry Co., Ltd. | Tongliao Toyota Pig farm | Fattening farm | 8 | 336 | 21 |
| Inner Mongolia Autonomous Region | Tongliao Xinhao Agriculture and Animal Husbandry Co., Ltd. | Tongliao Nanyuanxing Pig farm | Fattening farm | 8 | 336 | 39 |
| Inner Mongolia Autonomous Region | Tongliao Xinhao Agriculture and Animal Husbandry Co., Ltd. | Tongliao Su Family Pig farm | Fattening farm | 8 | 336 | 53 |
| Inner Mongolia Autonomous Region | Tongliao Xinhao Agriculture and Animal Husbandry Co., Ltd. | Hanbai Pig farm | Fattening farm | 8 | 336 | 55 |
| Inner Mongolia Autonomous Region | Tongliao Xinhao Agriculture and Animal Husbandry Co., Ltd. | Mayongjin Pig farm | Fattening farm | 8 | 336 | 24 |
| Inner Mongolia Autonomous Region | Tongliao Xinhao Agriculture and Animal Husbandry Co., Ltd. | Tongliao Yaohailisitai Pig farm | Fattening farm | 8 | 336 | 32 |
| Inner Mongolia Autonomous Region | Tongliao Xinhao Agriculture and Animal Husbandry Co., Ltd. | Xue Zhenhai Pig farm | Fattening farm | 8 | 336 | 39 |
| Inner Mongolia Autonomous Region | Liaoning Xinwang Food Co., Ltd. | Gongzhuling Xinhang Pig farm | Fattening farm | 8 | 336 | 40 |
| Inner Mongolia Autonomous Region | Tongliao Xinhao Agriculture and Animal Husbandry Co., Ltd. | Dehui Service Department | Fattening farm | 8 | 336 | 19 |
| Shandong Province | Caoxian Xinhao Agriculture and Animal Husbandry Co., Ltd. | Caoxian First farm | Sow farm | 8 | 336 | 54 |
| Shandong Province | Caoxian Xinhao Agriculture and Animal Husbandry Co., Ltd. | Caoxian Second farm | Sow farm | 8 | 336 | 34 |
| Shandong Province | Caoxian Xinhao Agriculture and Animal Husbandry Co., Ltd. | Caoxian Third farm | Sow farm | 8 | 336 | 39 |
| Shandong Province | Caoxian Xinhao Agriculture and Animal Husbandry Co., Ltd. | Caoxian Fourth farm | Sow farm | 8 | 336 | 20 |
| Shandong Province | Caoxian Xinhao Agriculture and Animal Husbandry Co., Ltd. | Caoxian Fifth farm | Sow farm | 8 | 336 | 39 |
| Shandong Province | Changyi Xinhao Animal Husbandry Co., Ltd. | Changyi Third farm | Sow farm | 8 | 336 | 17 |
| Shandong Province | Changyi Xinhao Animal Husbandry Co., Ltd. | Changyi Second farm | Sow farm | 8 | 336 | 47 |
| Shandong Province | Changyi Xinhao Animal Husbandry Co., Ltd. | Changyi First farm | Sow farm | 8 | 336 | 54 |
| Shandong Province | Shanxian Xinhao Agriculture and Animal Husbandry Co., Ltd. | Guotang Second farm | Sow farm | 8 | 336 | 57 |
| Shandong Province | Shanxian Xinhao Agriculture and Animal Husbandry Co., Ltd. | Guotang Third farm | Sow farm | 8 | 336 | 29 |
| Shandong Province | Shanxian Xinhao Agriculture and Animal Husbandry Co., Ltd. | Guotang Fourth farm | Sow farm | 8 | 336 | 20 |
| Shandong Province | Shanxian Xinhao Agriculture and Animal Husbandry Co., Ltd. | Guotang Fifth farm | Sow farm | 8 | 336 | 44 |
| Shandong Province | Shanxian Xinhao Agriculture and Animal Husbandry Co., Ltd. | Guotang First farm | Sow farm | 8 | 336 | 47 |
| Shandong Province | Shanxian Xinliu Agriculture and Animal Husbandry Technology Co., Ltd. | Anzhuang Second farm | Sow farm | 8 | 336 | 57 |
| Shandong Province | Dezhou Xinhao Agriculture and Animal Husbandry Co., Ltd. | Qianliu Second farm | Sow farm | 8 | 336 | 49 |
| Shandong Province | Dezhou Xinhao Agriculture and Animal Husbandry Co., Ltd. | Qianliu Fourth farm | Sow farm | 8 | 336 | 32 |
| Shandong Province | Dezhou Xinhao Agriculture and Animal Husbandry Co., Ltd. | Qianliu First farm | Sow farm | 8 | 336 | 49 |
| Shandong Province | Dong'e County Xinwang Liuhe Breeding Co., Ltd. | Dong'e Seventh farm | Sow farm | 8 | 336 | 30 |
| Shandong Province | Dong'e County Xinwang Liuhe Breeding Co., Ltd. | Dong'e Second farm | Sow farm | 8 | 336 | 29 |
| Shandong Province | Dong'e County Xinwang Liuhe Breeding Co., Ltd. | Dong'e Sixth farm | Sow farm | 8 | 336 | 13 |
| Shandong Province | Dong'e County Xinwang Liuhe Breeding Co., Ltd. | Dong'e Third farm | Sow farm | 8 | 336 | 43 |
| Shandong Province | Dong'e County Xinwang Liuhe Breeding Co., Ltd. | Dong'e Fourth farm | Sow farm | 8 | 336 | 30 |
| Shandong Province | Dong'e County Xinwang Liuhe Breeding Co., Ltd. | Dong'e Fifth farm | Sow farm | 8 | 336 | 41 |
| Shandong Province | Dong'e County Xinwang Liuhe Breeding Co., Ltd. | Dong'e First farm | Sow farm | 8 | 336 | 65 |
| Shandong Province | Dong'e County Xinwang Liuhe Breeding Co., Ltd. | Dong'e Fourth farm | Sow farm | 8 | 336 | 52 |
| Shandong Province | Dongying Xinhao Modern Agriculture and Animal Husbandry Co., Ltd. | Guanzhuang Seventh farm | Sow farm | 8 | 336 | 34 |
| Shandong Province | Dongying Xinhao Modern Agriculture and Animal Husbandry Co., Ltd. | Fangjia Seventh farm | Sow farm | 8 | 336 | 44 |
| Shandong Province | Dongying Xinhao Modern Agriculture and Animal Husbandry Co., Ltd. | Guanzhuang First farm | Sow farm | 8 | 336 | 48 |
| Shandong Province | Dongying Xinhao Modern Agriculture and Animal Husbandry Co., Ltd. | Fangjia Eighth farm | Sow farm | 8 | 336 | 34 |
| Shandong Province | Dongying Xinhao Modern Agriculture and Animal Husbandry Co., Ltd. | Beixue Second farm | Sow farm | 8 | 336 | 44 |
| Shandong Province | Dongying Xinhao Modern Agriculture and Animal Husbandry Co., Ltd. | Beixue Third farm | Sow farm | 8 | 336 | 39 |
| Shandong Province | Dongying Xinhao Modern Agriculture and Animal Husbandry Co., Ltd. | Beixue Fourth farm | Sow farm | 8 | 336 | 40 |
| Shandong Province | Dongying Xinhao Modern Agriculture and Animal Husbandry Co., Ltd. | Beixue Fifth farm | Sow farm | 8 | 336 | 23 |
| Shandong Province | Dongying Xinhao Modern Agriculture and Animal Husbandry Co., Ltd. | Guanzhuang Second farm | Sow farm | 8 | 336 | 19 |
| Shandong Province | Dongying Xinhao Modern Agriculture and Animal Husbandry Co., Ltd. | Guanzhuang Third farm | Sow farm | 8 | 336 | 65 |
| Shandong Province | Dongying Xinhao Modern Agriculture and Animal Husbandry Co., Ltd. | Guanzhuang Fourth farm | Sow farm | 8 | 336 | 47 |
| Shandong Province | Dongying Xinhao Modern Agriculture and Animal Husbandry Co., Ltd. | Guanzhuang Fifth farm | Sow farm | 8 | 336 | 24 |
| Shandong Province | Dongying Xinhao Modern Agriculture and Animal Husbandry Co., Ltd. | Fangjia Third farm | Sow farm | 8 | 336 | 35 |
| Shandong Province | Gaomi Liuhe Pig Raising Co., Ltd. | Gaomi Pig farm | Sow farm | 8 | 336 | 55 |
| Shandong Province | Gaomi Xinliu Agriculture and Animal Husbandry Technology Co., Ltd. | Zhaoli Fifth farm | Sow farm | 8 | 336 | 73 |
| Shandong Province | Gaomi Xinliu Agriculture and Animal Husbandry Technology Co., Ltd. | Zhaoli Second farm | Sow farm | 8 | 336 | 77 |
| Shandong Province | Gaomi Xinliu Agriculture and Animal Husbandry Technology Co., Ltd. | Zhaoli Third farm | Sow farm | 8 | 336 | 43 |
| Shandong Province | Gaomi Xinliu Agriculture and Animal Husbandry Technology Co., Ltd. | Zhaoli Fourth farm | Sow farm | 8 | 336 | 47 |
| Shandong Province | Gaomi Xinliu Agriculture and Animal Husbandry Technology Co., Ltd. | Zhaoli First farm | Sow farm | 8 | 336 | 51 |
| Shandong Province | Gaotang County Xinhao Agriculture and Animal Husbandry Co., Ltd. | Gaotang Second farm | Sow farm | 8 | 336 | 30 |
| Shandong Province | Gaotang County Xinhao Agriculture and Animal Husbandry Co., Ltd. | Gaotang Third farm | Sow farm | 8 | 336 | 36 |
| Shandong Province | Gaotang County Xinhao Agriculture and Animal Husbandry Co., Ltd. | Gaotang Fourth farm | Sow farm | 8 | 336 | 67 |
| Shandong Province | Jiaxiang County Xinwang Liuhe Breeding Co., Ltd. | Jiaxiang Third farm | Sow farm | 8 | 336 | 54 |
| Shandong Province | Jiaxiang County Xinwang Liuhe Breeding Co., Ltd. | Jiaxiang Second farm | Sow farm | 8 | 336 | 40 |
| Shandong Province | Jiaxiang County Xinwang Liuhe Breeding Co., Ltd. | Jiaxiang First farm | Sow farm | 8 | 336 | 43 |
| Shandong Province | Juye Xinhao Agriculture and Animal Husbandry Co., Ltd. | Dongguantun First farm | Sow farm | 8 | 336 | 39 |
| Shandong Province | Juye Xinhao Agriculture and Animal Husbandry Co., Ltd. | Dongguantun Sixth farm | Sow farm | 8 | 336 | 29 |
| Shandong Province | Juye Xinhao Agriculture and Animal Husbandry Co., Ltd. | Dongguantun Fifth farm | Sow farm | 8 | 336 | 50 |
| Shandong Province | Juye Xinhao Agriculture and Animal Husbandry Co., Ltd. | Dongguantun Fourth farm | Sow farm | 8 | 336 | 64 |
| Shandong Province | Juye Xinhao Agriculture and Animal Husbandry Co., Ltd. | Dongguantun Second farm | Sow farm | 8 | 336 | 31 |
| Shandong Province | Juye Xinhao Agriculture and Animal Husbandry Co., Ltd. | Dongguantun Seventh farm | Sow farm | 8 | 336 | 34 |
| Shandong Province | Juye Xinhao Agriculture and Animal Husbandry Co., Ltd. | Dongguantun Third farm | Sow farm | 8 | 336 | 14 |
| Shandong Province | Juye Xinhao Agriculture and Animal Husbandry Co., Ltd. | Juye Dongguantun Pig farm | Sow farm | 8 | 336 | 43 |
| Shandong Province | Laixi New Hope Liuhe Agriculture and Animal Husbandry Co., Ltd. | Xixiagzhuang Seventh farm | Sow farm | 8 | 336 | 49 |
| Shandong Province | Laixi New Hope Liuhe Agriculture and Animal Husbandry Co., Ltd. | Xixiagzhuang First farm | Sow farm | 8 | 336 | 55 |
| Shandong Province | Laixi New Hope Liuhe Agriculture and Animal Husbandry Co., Ltd. | Cuijiazhuang Second farm | Sow farm | 8 | 336 | 57 |
| Shandong Province | Laixi New Hope Liuhe Agriculture and Animal Husbandry Co., Ltd. | Songwangzhuang Third farm | Sow farm | 8 | 336 | 29 |
| Shandong Province | Laixi New Hope Liuhe Agriculture and Animal Husbandry Co., Ltd. | Songwangzhuang Fourth farm | Sow farm | 8 | 336 | 36 |
| Shandong Province | Laixi New Hope Liuhe Agriculture and Animal Husbandry Co., Ltd. | Songwangzhuang Fifth farm | Sow farm | 8 | 336 | 54 |
| Shandong Province | Laixi New Hope Liuhe Agriculture and Animal Husbandry Co., Ltd. | Xixiagzhuang Second farm | Sow farm | 8 | 336 | 38 |
| Shandong Province | Laixi New Hope Liuhe Agriculture and Animal Husbandry Co., Ltd. | Xixiagzhuang Third farm | Sow farm | 8 | 336 | 30 |
| Shandong Province | Laixi New Hope Liuhe Agriculture and Animal Husbandry Co., Ltd. | Songwangzhuang First farm | Sow farm | 8 | 336 | 85 |
| Shandong Province | Laixi New Hope Liuhe Agriculture and Animal Husbandry Co., Ltd. | Songwangzhuang Second farm | Sow farm | 8 | 336 | 48 |
| Shandong Province | Laiyang Xinmu Breeding Co., Ltd. | Tanggezhuang First farm | Sow farm | 8 | 336 | 49 |
| Shandong Province | Laiyang Xinmu Breeding Co., Ltd. | Tanggezhuang Second farm | Sow farm | 8 | 336 | 24 |
| Shandong Province | Laiyang Xinmu Breeding Co., Ltd. | Tanggezhuang Third farm | Sow farm | 8 | 336 | 23 |
| Shandong Province | Laiyang Xinhao Animal Husbandry Co., Ltd. | Laiyang Fourth farm | Sow farm | 8 | 336 | 49 |
| Shandong Province | Laiyang Xinhao Animal Husbandry Co., Ltd. | Laiyang First farm | Sow farm | 8 | 336 | 34 |
| Shandong Province | Laiyang Xinhao Animal Husbandry Co., Ltd. | Laiyang Second farm | Sow farm | 8 | 336 | 59 |
| Shandong Province | Laiyang Xinhao Animal Husbandry Co., Ltd. | Laiyang Third farm | Sow farm | 8 | 336 | 55 |
| Shandong Province | Lai Zhou City New Hope Liuhe Agriculture and Animal Husbandry Co., Ltd. | Nanxiang Third farm | Sow farm | 8 | 336 | 30 |
| Shandong Province | Lai Zhou City New Hope Liuhe Agriculture and Animal Husbandry Co., Ltd. | Nanxiang First farm | Sow farm | 8 | 336 | 67 |
| Shandong Province | Lai Zhou City New Hope Liuhe Agriculture and Animal Husbandry Co., Ltd. | Nanxiang Eighth farm | Sow farm | 8 | 336 | 30 |
| Shandong Province | Lai Zhou City New Hope Liuhe Agriculture and Animal Husbandry Co., Ltd. | Nanxiang Second farm | Sow farm | 8 | 336 | 32 |
| Shandong Province | Lai Zhou City New Hope Liuhe Agriculture and Animal Husbandry Co., Ltd. | Nanxiang Ninth farm | Sow farm | 8 | 336 | 40 |
| Shandong Province | Lai Zhou City New Hope Liuhe Agriculture and Animal Husbandry Co., Ltd. | Nanxiang Sixth farm | Sow farm | 8 | 336 | 39 |
| Shandong Province | Lai Zhou City New Hope Liuhe Agriculture and Animal Husbandry Co., Ltd. | Nanxiang Seventh farm | Sow farm | 8 | 336 | 44 |
| Shandong Province | Lai Zhou City New Hope Liuhe Agriculture and Animal Husbandry Co., Ltd. | Nanxiang Tenth farm | Sow farm | 8 | 336 | 63 |
| Shandong Province | Lai Zhou City New Hope Liuhe Agriculture and Animal Husbandry Co., Ltd. | Nanxiang Fourth farm | Sow farm | 8 | 336 | 23 |
| Shandong Province | Lai Zhou City New Hope Liuhe Agriculture and Animal Husbandry Co., Ltd. | Nanxiang Fifth farm | Sow farm | 8 | 336 | 24 |
| Shandong Province | Liaocheng Xinhao Agriculture and Animal Husbandry Co., Ltd. | Yanggu Fifth farm | Sow farm | 8 | 336 | 56 |
| Shandong Province | Liaocheng Xinhao Agriculture and Animal Husbandry Co., Ltd. | Yanggu Second farm | Sow farm | 8 | 336 | 52 |
| Shandong Province | Liaocheng Xinhao Agriculture and Animal Husbandry Co., Ltd. | Yanggu Third farm | Sow farm | 8 | 336 | 29 |
| Shandong Province | Liaocheng Xinhao Agriculture and Animal Husbandry Co., Ltd. | Yanggu Fourth farm | Sow farm | 8 | 336 | 30 |
| Shandong Province | Liaocheng Xinhao Agriculture and Animal Husbandry Co., Ltd. | Yanggu First farm | Sow farm | 8 | 336 | 32 |
| Shandong Province | Linshu Liuhe Breeding Pig Co., Ltd. | Linshu Second farm | Sow farm | 8 | 336 | 49 |
| Shandong Province | Linyi Xinhao Breeding Co., Ltd. | Yanma First farm | Sow farm | 8 | 336 | 48 |
| Shandong Province | Linyi Liuhe Breeding Pig Co., Ltd. | Linyi Pig farm | Sow farm | 8 | 336 | 50 |
| Shandong Province | Tai'an City Xincheng Agriculture and Animal Husbandry Co., Ltd. | Haizhen Breeding farm | Sow farm | 8 | 336 | 39 |
| Shandong Province | Wulian Xinhao Agriculture and Animal Husbandry Co., Ltd. | Dongjiaying Eighth farm | Sow farm | 8 | 336 | 32 |
| Shandong Province | Wulian Xinhao Agriculture and Animal Husbandry Co., Ltd. | Dongjiaying Sixth farm | Sow farm | 8 | 336 | 30 |
| Shandong Province | Wulian Xinhao Agriculture and Animal Husbandry Co., Ltd. | Dongjiaying Fifth farm | Sow farm | 8 | 336 | 39 |
| Shandong Province | Wulian Xinhao Agriculture and Animal Husbandry Co., Ltd. | Dongjiaying First farm | Sow farm | 8 | 336 | 50 |
| Shandong Province | Wulian Xinhao Agriculture and Animal Husbandry Co., Ltd. | Dongjiaying Fourth farm | Sow farm | 8 | 336 | 31 |
| Shandong Province | Wulian Xinhao Agriculture and Animal Husbandry Co., Ltd. | Dongjiaying Second farm | Sow farm | 8 | 336 | 34 |
| Shandong Province | Xiajin New Hope Liuhe Agriculture and Animal Husbandry Co., Ltd. | Xiajin Seventh farm | Sow farm | 8 | 336 | 49 |
| Shandong Province | Xiajin New Hope Liuhe Agriculture and Animal Husbandry Co., Ltd. | Xiazhuang Third farm | Sow farm | 8 | 336 | 43 |
| Shandong Province | Xiajin New Hope Liuhe Agriculture and Animal Husbandry Co., Ltd. | Xiajin Second farm | Sow farm | 8 | 336 | 29 |
| Shandong Province | Xiajin New Hope Liuhe Agriculture and Animal Husbandry Co., Ltd. | Xiajin Sixth farm | Sow farm | 8 | 336 | 27 |
| Shandong Province | Xiajin New Hope Liuhe Agriculture and Animal Husbandry Co., Ltd. | Xiajin Third farm | Sow farm | 8 | 336 | 83 |
| Shandong Province | Xiajin New Hope Liuhe Agriculture and Animal Husbandry Co., Ltd. | Xiajin Fourth farm | Sow farm | 8 | 336 | 50 |
| Shandong Province | Xiajin New Hope Liuhe Agriculture and Animal Husbandry Co., Ltd. | Xiajin Fifth farm | Sow farm | 8 | 336 | 47 |
| Shandong Province | Xiajin New Hope Liuhe Agriculture and Animal Husbandry Co., Ltd. | Xiajin First farm | Sow farm | 8 | 336 | 28 |
| Shandong Province | Xiajin New Hope Liuhe Agriculture and Animal Husbandry Co., Ltd. | Xiazhuang Second farm | Sow farm | 8 | 336 | 29 |
| Shandong Province | Xiajin New Hope Liuhe Agriculture and Animal Husbandry Co., Ltd. | Xiazhuang Fourth farm | Sow farm | 8 | 336 | 65 |
| Shandong Province | Xiajin New Hope Liuhe Agriculture and Animal Husbandry Co., Ltd. | Xiazhuang First farm | Sow farm | 8 | 336 | 30 |
| Shandong Province | New Hope Liuhe (Zibo) Agricultural Science and Technology Development Co., Ltd. | Kunlun Third farm | Sow farm | 8 | 336 | 56 |
| Shandong Province | New Hope Liuhe (Zibo) Agricultural Science and Technology Development Co., Ltd. | Kunlun Second farm | Sow farm | 8 | 336 | 49 |
| Shandong Province | Yantai Xinhao Agriculture and Animal Husbandry Co., Ltd. | Yadi First farm | Sow farm | 8 | 336 | 53 |
| Shandong Province | Yantai Xinhao Agriculture and Animal Husbandry Co., Ltd. | Yadi Eighth farm | Sow farm | 8 | 336 | 49 |
| Shandong Province | Yantai Xinhao Agriculture and Animal Husbandry Co., Ltd. | Yadi Second farm | Sow farm | 8 | 336 | 74 |
| Shandong Province | Yantai Xinhao Agriculture and Animal Husbandry Co., Ltd. | Yadi Ninth farm | Sow farm | 8 | 336 | 33 |
| Shandong Province | Yantai Xinhao Agriculture and Animal Husbandry Co., Ltd. | Yadi Sixth farm | Sow farm | 8 | 336 | 39 |
| Shandong Province | Yantai Xinhao Agriculture and Animal Husbandry Co., Ltd. | Yadi Seventh farm | Sow farm | 8 | 336 | 37 |
| Shandong Province | Yantai Xinhao Agriculture and Animal Husbandry Co., Ltd. | Yadi Third farm | Sow farm | 8 | 336 | 69 |
| Shandong Province | Yantai Xinhao Agriculture and Animal Husbandry Co., Ltd. | Yadi Fourth farm | Sow farm | 8 | 336 | 46 |
| Shandong Province | Yantai Xinhao Agriculture and Animal Husbandry Co., Ltd. | Yadi Fifth farm | Sow farm | 8 | 336 | 45 |
| Shandong Province | Yucheng City New Hope Liuhe Breeding Pig Co., Ltd. | Yucheng Second farm | Sow farm | 8 | 336 | 39 |
| Shandong Province | Yucheng City New Hope Liuhe Breeding Pig Co., Ltd. | Yucheng First farm | Sow farm | 8 | 336 | 50 |
| Shandong Province | Yucheng City New Hope Liuhe Breeding Pig Co., Ltd. | Yucheng Xinliu | Sow farm | 8 | 336 | 53 |
| Shandong Province | Zhoucheng Xinliu Agriculture and Animal Husbandry Technology Co., Ltd. | Qujiazhuang Fifth farm | Sow farm | 8 | 336 | 56 |
| Shandong Province | Zhoucheng Xinliu Agriculture and Animal Husbandry Technology Co., Ltd. | Qujiazhuang Second farm | Sow farm | 8 | 336 | 47 |
| Shandong Province | Zhoucheng Xinliu Agriculture and Animal Husbandry Technology Co., Ltd. | Qujiazhuang Third farm | Sow farm | 8 | 336 | 29 |
| Shandong Province | Zhoucheng Xinliu Agriculture and Animal Husbandry Technology Co., Ltd. | Qujiazhuang Fourth farm | Sow farm | 8 | 336 | 27 |
| Shandong Province | Zhoucheng Xinliu Agriculture and Animal Husbandry Technology Co., Ltd. | Qujiazhuang First farm | Sow farm | 8 | 336 | 63 |
| Shandong Province | Binzhou New Hope Liuhe Agriculture and Animal Husbandry Co., Ltd. | Wudi Liuhe Factory | Fattening farm | 8 | 336 | 40 |
| Shandong Province | Cao County Xinhao Agriculture and Animal Husbandry Co., Ltd. | Caoxian Guobinzhai Pig farm | Fattening farm | 8 | 336 | 65 |
| Shandong Province | Cao County Xinhao Agriculture and Animal Husbandry Co., Ltd. | Cai Zengyu Pig farm | Fattening farm | 8 | 336 | 39 |
| Shandong Province | Cao County Xinhao Agriculture and Animal Husbandry Co., Ltd. | Dai Fuguo Pig farm | Fattening farm | 8 | 336 | 31 |
| Shandong Province | Cao County Xinhao Agriculture and Animal Husbandry Co., Ltd. | Fu Lihong Pig farm | Fattening farm | 8 | 336 | 22 |
| Shandong Province | Changle New Hope Liuhe Agriculture and Animal Husbandry Co., Ltd. | Shiren Third farm | Fattening farm | 8 | 336 | 26 |
| Shandong Province | Changle New Hope Liuhe Agriculture and Animal Husbandry Co., Ltd. | Shiren Fourth farm | Fattening farm | 8 | 336 | 27 |
| Shandong Province | Changle New Hope Liuhe Agriculture and Animal Husbandry Co., Ltd. | Shiren First farm | Fattening farm | 8 | 336 | 19 |
| Shandong Province | Dong'e County New Hope Liuhe Breeding Pig Co., Ltd. | Dong'e Yaozhai Pig farm | Fattening farm | 8 | 336 | 23 |
| Shandong Province | Dong'e County New Hope Liuhe Breeding Pig Co., Ltd. | Zhaozhuang Second farm | Fattening farm | 8 | 336 | 21 |
| Shandong Province | Dong'e County New Hope Liuhe Breeding Pig Co., Ltd. | Zhao Zhuang Third farm | Fattening farm | 8 | 336 | 20 |
| Shandong Province | Dong'e County New Hope Liuhe Breeding Pig Co., Ltd. | Zhao Zhuang Fourth farm | Fattening farm | 8 | 336 | 40 |
| Shandong Province | Dong'e County New Hope Liuhe Breeding Pig Co., Ltd. | Zhao Zhuang First farm | Fattening farm | 8 | 336 | 49 |
| Shandong Province | Dongying City Xinhao Modern Agriculture and Animal Husbandry Co., Ltd. | Bei Xue Sixth farm | Fattening farm | 8 | 336 | 47 |
| Shandong Province | Gaomi Xinliu Agriculture and Animal Husbandry Technology Co., Ltd. | Zhao Lizhan Pig farm | Fattening farm | 8 | 336 | 37 |
| Shandong Province | Gaotang County Xinhao Agriculture and Animal Husbandry Co., Ltd. | Gao Tang First farm | Fattening farm | 8 | 336 | 36 |
| Shandong Province | Heze Xinhao Agriculture and Animal Husbandry Co., Ltd. | Liang Tang First farm | Fattening farm | 8 | 336 | 39 |
| Shandong Province | Jiaxiang County New Hope Liuhe Pig Breeding Co., Ltd. | Jiaxiang Breeding Pig Service Department | Fattening farm | 8 | 336 | 46 |
| Shandong Province | Jing County Xinhao Agriculture and Animal Husbandry Co., Ltd. | Linyi Duan Dian Lease farm | Fattening farm | 8 | 336 | 40 |
| Shandong Province | Juye Xinhao Agriculture and Animal Husbandry Co., Ltd. | Dongguantun Eighth farm | Fattening farm | 8 | 336 | 43 |
| Shandong Province | Juye Xinhao Agriculture and Animal Husbandry Co., Ltd. | Dongguantun Ninth farm | Fattening farm | 8 | 336 | 31 |
| Shandong Province | Juye Xinhao Agriculture and Animal Husbandry Co., Ltd. | Dongguantun Twelfth farm | Fattening farm | 8 | 336 | 33 |
| Shandong Province | Laixi City New Hope Liuhe Agriculture and Animal Husbandry Co., Ltd. | Cuijiazhuang Pig farm | Fattening farm | 8 | 336 | 27 |
| Shandong Province | Laixi City New Hope Liuhe Agriculture and Animal Husbandry Co., Ltd. | Tuanwang Pig farm | Fattening farm | 8 | 336 | 54 |
| Shandong Province | Laixi City New Hope Liuhe Agriculture and Animal Husbandry Co., Ltd. | Xixiagezhuang Eighth farm | Fattening farm | 8 | 336 | 29 |
| Shandong Province | Laixi City New Hope Liuhe Agriculture and Animal Husbandry Co., Ltd. | Xixiagezhuang Sixth farm | Fattening farm | 8 | 336 | 18 |
| Shandong Province | Laixi City New Hope Liuhe Agriculture and Animal Husbandry Co., Ltd. | Xixiagezhuang Fourth farm | Fattening farm | 8 | 336 | 28 |
| Shandong Province | Laixi City New Hope Liuhe Agriculture and Animal Husbandry Co., Ltd. | Xixiagezhuang Fifth farm | Fattening farm | 8 | 336 | 36 |
| Shandong Province | Laiyang City Ximu Breeding Co., Ltd. | Tanggezhuang Fourth farm | Fattening farm | 8 | 336 | 69 |
| Shandong Province | Laizhou City Xinhao Animal Husbandry Co., Ltd. | Gutai Kou Second farm | Fattening farm | 8 | 336 | 34 |
| Shandong Province | Laizhou City Xinhao Animal Husbandry Co., Ltd. | Gutai Kou First farm | Fattening farm | 8 | 336 | 35 |
| Shandong Province | Laizhou City New Hope Liuhe Agriculture and Animal Husbandry Co., Ltd. | Nanxiang Pig farm | Fattening farm | 8 | 336 | 30 |
| Shandong Province | Liaocheng Xinhao Agriculture and Animal Husbandry Co., Ltd. | Liaocheng Qiji Pig farm | Fattening farm | 8 | 336 | 34 |
| Shandong Province | Liaocheng Xinhao Agriculture and Animal Husbandry Co., Ltd. | Liaocheng Nanguan South Pig farm | Fattening farm | 8 | 336 | 19 |
| Shandong Province | Pingyuan Xinliu Agriculture and Animal Husbandry Technology Co., Ltd. | Pingyuan Service Department | Fattening farm | 8 | 336 | 21 |
| Shandong Province | Pingyuan Xinliu Agriculture and Animal Husbandry Technology Co., Ltd. | Pingyuan Yongsheng Pig farm | Fattening farm | 8 | 336 | 23 |
| Shandong Province | Shandong Liuhe Yinbao Breeding Co., Ltd. | Feicheng Pig farm | Fattening farm | 8 | 336 | 22 |
| Shandong Province | Shandong New Hope Liuhe Agriculture and Animal Husbandry Technology Co., Ltd. | Leiji Experiment farm | Fattening farm | 8 | 336 | 74 |
| Shandong Province | Shandong New Hope Liuhe Agriculture and Animal Husbandry Technology Co., Ltd. | Wucheng Experiment farm | Fattening farm | 8 | 336 | 79 |
| Shandong Province | Wulian Xinhao Agriculture and Animal Husbandry Co., Ltd. | Dongjiaying Seventh farm | Fattening farm | 8 | 336 | 38 |
| Shandong Province | Xiajin New Hope Liuhe Agriculture and Animal Husbandry Co., Ltd. | Bafangta Pig farm | Fattening farm | 8 | 336 | 32 |
| Shandong Province | Xiajin New Hope Liuhe Agriculture and Animal Husbandry Co., Ltd. | Hanqiao Pig farm | Fattening farm | 8 | 336 | 23 |
| Shandong Province | Xiajin New Hope Liuhe Agriculture and Animal Husbandry Co., Ltd. | Jiuying Pig farm | Fattening farm | 8 | 336 | 29 |
| Shandong Province | Xiajin New Hope Liuhe Agriculture and Animal Husbandry Co., Ltd. | Xiajin Houzhuang Pig farm | Fattening farm | 8 | 336 | 50 |
| Shandong Province | Xiajin New Hope Liuhe Agriculture and Animal Husbandry Co., Ltd. | Xiajin Zuowangzhuang Pig farm | Fattening farm | 8 | 336 | 46 |
| Shandong Province | Xiajin New Hope Liuhe Agriculture and Animal Husbandry Co., Ltd. | Xiajin Yueji Pig farm | Fattening farm | 8 | 336 | 20 |
| Shandong Province | Xiajin New Hope Liuhe Breeding Co., Ltd. | Leiji First farm | Fattening farm | 8 | 336 | 21 |
| Shandong Province | Xiajin New Hope Liuhe Breeding Co., Ltd. | Zhangji Pig farm | Fattening farm | 8 | 336 | 63 |
| Shandong Province | Yantai Xinhao Agriculture and Animal Husbandry Co., Ltd. | Yadi Eighteenth farm | Fattening farm | 8 | 336 | 40 |
| Shandong Province | Yantai Xinhao Agriculture and Animal Husbandry Co., Ltd. | Yadi Twelfth farm | Fattening farm | 8 | 336 | 43 |
| Shandong Province | Yantai Xinhao Agriculture and Animal Husbandry Co., Ltd. | Yadi Nineteenth farm | Fattening farm | 8 | 336 | 25 |
| Shandong Province | Yantai Xinhao Agriculture and Animal Husbandry Co., Ltd. | Yadi Sixteenth farm | Fattening farm | 8 | 336 | 25 |
| Shandong Province | Yantai Xinhao Agriculture and Animal Husbandry Co., Ltd. | Yadi Seventeenth farm | Fattening farm | 8 | 336 | 23 |
| Shandong Province | Yantai Xinhao Agriculture and Animal Husbandry Co., Ltd. | Yadi Thirteenth farm | Fattening farm | 8 | 336 | 35 |
| Shandong Province | Yantai Xinhao Agriculture and Animal Husbandry Co., Ltd. | Yadi Fourteenth farm | Fattening farm | 8 | 336 | 36 |
| Shandong Province | Yantai Xinhao Agriculture and Animal Husbandry Co., Ltd. | Yadi Fifteenth farm | Fattening farm | 8 | 336 | 13 |
| Shandong Province | Yantai Xinhao Agriculture and Animal Husbandry Co., Ltd. | Yadi Eleventh farm | Fattening farm | 8 | 336 | 46 |
| Shandong Province | Yucheng City New Hope Liuhe Pig Breeding Co., Ltd. | Yucheng Mengsi Pig farm | Fattening farm | 8 | 336 | 29 |
| Shandong Province | Yucheng City New Hope Liuhe Pig Breeding Co., Ltd. | Yucheng Qingxiang Pig farm | Fattening farm | 8 | 336 | 30 |
| Shandong Province | Zhoucheng Xinliu Agriculture and Animal Husbandry Technology Co., Ltd. | Jiayue Eighth farm | Fattening farm | 8 | 336 | 57 |
| Shandong Province | Zhoucheng Xinliu Agriculture and Animal Husbandry Technology Co., Ltd. | Jiayue Second farm | Fattening farm | 8 | 336 | 43 |
| Shandong Province | Zhoucheng Xinliu Agriculture and Animal Husbandry Technology Co., Ltd. | Jiayue Seventh farm | Fattening farm | 8 | 336 | 47 |
| Shandong Province | Zhoucheng Xinliu Agriculture and Animal Husbandry Technology Co., Ltd. | Jiayue Third farm | Fattening farm | 8 | 336 | 32 |
| Shandong Province | Zhoucheng Xinliu Agriculture and Animal Husbandry Technology Co., Ltd. | Jiayue Fourth farm | Fattening farm | 8 | 336 | 67 |
| Shanxi Province | Shuozhou Xinhao Agriculture and Animal Husbandry Co., Ltd. | Xiatuan First farm | Sow farm | 8 | 336 | 77 |
| Shanxi Province | Shuozhou Xinhao Agriculture and Animal Husbandry Co., Ltd. | Xiatuan Eighth farm | Sow farm | 8 | 336 | 101 |
| Shanxi Province | Shuozhou Xinhao Agriculture and Animal Husbandry Co., Ltd. | Xiatuan Second farm | Sow farm | 8 | 336 | 62 |
| Shanxi Province | Shuozhou Xinhao Agriculture and Animal Husbandry Co., Ltd. | Xiatuan Ninth farm | Sow farm | 8 | 336 | 76 |
| Shanxi Province | Shuozhou Xinhao Agriculture and Animal Husbandry Co., Ltd. | Xiatuan Sixth farm | Sow farm | 8 | 336 | 84 |
| Shanxi Province | Shuozhou Xinhao Agriculture and Animal Husbandry Co., Ltd. | Xiatuan Seventh farm | Sow farm | 8 | 336 | 89 |
| Shanxi Province | Shuozhou Xinhao Agriculture and Animal Husbandry Co., Ltd. | Xiatuan Third farm | Sow farm | 8 | 336 | 64 |
| Shanxi Province | Shuozhou Xinhao Agriculture and Animal Husbandry Co., Ltd. | Xiatuan Fourth farm | Sow farm | 8 | 336 | 68 |
| Shanxi Province | Shuozhou Xinhao Agriculture and Animal Husbandry Co., Ltd. | Xiatuan Fifth farm | Sow farm | 8 | 336 | 75 |
| Shanxi Province | Shuozhou Xinhao Agriculture and Animal Husbandry Co., Ltd. | Xiatuan Tenth farm | Sow farm | 8 | 336 | 93 |
| Shanxi Province | Huairou New Hope Liuhe Agriculture and Animal Husbandry Co., Ltd. | Huair仁 Pig farm | Fattening farm | 8 | 336 | 43 |
| Shaanxi Province | Heyang Xinliu Agriculture and Animal Husbandry Technology Co., Ltd. | Heyang Second farm | Sow farm | 8 | 336 | 67 |
| Shaanxi Province | Heyang Xinliu Agriculture and Animal Husbandry Technology Co., Ltd. | Heyang First farm | Sow farm | 8 | 336 | 33 |
| Shaanxi Province | Heyang Xinliu Agriculture and Animal Husbandry Technology Co., Ltd. | Heyang Pig farm | Sow farm | 8 | 336 | 49 |
| Shaanxi Province | Heyang Xinliu Agriculture and Animal Husbandry Technology Co., Ltd. | Heyang Zhengneng Second farm | Sow farm | 8 | 336 | 71 |
| Shaanxi Province | Heyang Xinliu Agriculture and Animal Husbandry Technology Co., Ltd. | Heyang Zhengneng First farm | Sow farm | 8 | 336 | 57 |
| Shaanxi Province | Pucheng Xinliu Technology Co., Ltd. | Pucheng Parent Stock | Sow farm | 8 | 336 | 29 |
| Shaanxi Province | Pucheng Xinliu Technology Co., Ltd. | Pucheng Parent Stock Second farm | Sow farm | 8 | 336 | 20 |
| Shaanxi Province | Pucheng Xinliu Technology Co., Ltd. | Pucheng Parent Stock Third farm | Sow farm | 8 | 336 | 29 |
| Shaanxi Province | Pucheng Xinliu Technology Co., Ltd. | Pucheng Parent Stock Fourth farm | Sow farm | 8 | 336 | 47 |
| Shaanxi Province | Pucheng Xinliu Technology Co., Ltd. | Pucheng Parent Stock First farm | Sow farm | 8 | 336 | 23 |
| Shaanxi Province | Shaanxi Zhongshan Xingwang Agriculture and Animal Husbandry Technology Co., Ltd. | Jingyang Pig farm | Sow farm | 8 | 336 | 30 |
| Shaanxi Province | Weinan Xinliu Technology Co., Ltd. | Gushi Parent Stock | Sow farm | 8 | 336 | 39 |
| Shaanxi Province | Weinan Xinliu Technology Co., Ltd. | Lindi Parent Stock | Sow farm | 8 | 336 | 54 |
| Shaanxi Province | Weinan Xinliu Technology Co., Ltd. | Gushi Parent Stock Second farm | Sow farm | 8 | 336 | 29 |
| Shaanxi Province | Weinan Xinliu Technology Co., Ltd. | Gushi Parent Stock Third farm | Sow farm | 8 | 336 | 31 |
| Shaanxi Province | Weinan Xinliu Technology Co., Ltd. | Gushi Parent Stock Fourth farm | Sow farm | 8 | 336 | 26 |
| Shaanxi Province | Weinan Xinliu Technology Co., Ltd. | Gushi Parent Stock First farm | Sow farm | 8 | 336 | 35 |
| Shaanxi Province | Weinan Xinliu Technology Co., Ltd. | Lindi Parent Stock Second farm | Sow farm | 8 | 336 | 29 |
| Shaanxi Province | Weinan Xinliu Technology Co., Ltd. | Lindi Parent Stock Third farm | Sow farm | 8 | 336 | 34 |
| Shaanxi Province | Weinan Xinliu Technology Co., Ltd. | Lindi Parent Stock Fourth farm | Sow farm | 8 | 336 | 48 |
| Shaanxi Province | Weinan Xinliu Technology Co., Ltd. | Lindi Parent Stock First farm | Sow farm | 8 | 336 | 53 |
| Shaanxi Province | Wuqi Xinliu Technology Co., Ltd. | Zhangfangtai Parent Stock | Sow farm | 8 | 336 | 69 |
| Shaanxi Province | Wuqi Xinliu Technology Co., Ltd. | Zhangfangtai Second farm | Sow farm | 8 | 336 | 29 |
| Shaanxi Province | Wuqi Xinliu Technology Co., Ltd. | Zhangfangtai First farm | Sow farm | 8 | 336 | 34 |
| Shaanxi Province | Xianyang Yongxiang Agricultural Technology Co., Ltd. | Yongxiang Pig farm | Sow farm | 8 | 336 | 51 |
| Shaanxi Province | Yan'an Benyuan Agricultural Technology Development Co., Ltd. | Huangzhang Parent Stock | Sow farm | 8 | 336 | 64 |
| Shaanxi Province | Yan'an Benyuan Agricultural Technology Development Co., Ltd. | Jiuxian Pig farm | Sow farm | 8 | 336 | 63 |
| Shaanxi Province | Yan'an Benyuan Agricultural Technology Development Co., Ltd. | Huangzhang Second farm | Sow farm | 8 | 336 | 53 |
| Shaanxi Province | Yan'an Benyuan Agricultural Technology Development Co., Ltd. | Huangzhang Third farm | Sow farm | 8 | 336 | 20 |
| Shaanxi Province | Yan'an Benyuan Agricultural Technology Development Co., Ltd. | Huangzhang First farm | Sow farm | 8 | 336 | 33 |
| Shaanxi Province | Yan'an Benyuan Agricultural Technology Development Co., Ltd. | Fuxian Pig farm | Sow farm | 8 | 336 | 36 |
| Shaanxi Province | Yangling Benxiang Agricultural Industry Group Co., Ltd. Lijia Pig farm | Liji Pig farm | Sow farm | 8 | 336 | 36 |
| Shaanxi Province | Yijun Xinliu Technology Co., Ltd. | Yijun Parent Stock | Sow farm | 8 | 336 | 54 |
| Shaanxi Province | Yijun Xinniu Technology Co., Ltd. | Yijun Second farm | Sow farm | 8 | 336 | 43 |
| Shaanxi Province | Yijun Xinniu Technology Co., Ltd. | Yijun Third farm | Sow farm | 8 | 336 | 40 |
| Shaanxi Province | Heyang Zhenghe Modern Animal Husbandry Co., Ltd. | Heyang Fattening Second farm | Fattening farm | 8 | 336 | 40 |
| Shaanxi Province | Heyang Zhenghe Modern Animal Husbandry Co., Ltd. | Heyang Agriculture and Animal Husbandry Fattening farm | Fattening farm | 8 | 336 | 32 |
| Shaanxi Province | Liquan Xinhao Liuhe Agriculture and Animal Husbandry Co., Ltd. | Liquan First farm | Fattening farm | 8 | 336 | 30 |
| Shaanxi Province | Luochuan Xinniu Technology Co., Ltd. | Beigu Second farm | Fattening farm | 8 | 336 | 29 |
| Shaanxi Province | Luochuan Xinniu Technology Co., Ltd. | Beigu First farm | Fattening farm | 8 | 336 | 54 |
| Shaanxi Province | Luochuan Xinniu Technology Co., Ltd. | Wujiao Third farm | Fattening farm | 8 | 336 | 20 |
| Shaanxi Province | Luochuan Xinniu Technology Co., Ltd. | Wujiao First farm | Fattening farm | 8 | 336 | 31 |
| Shaanxi Province | Pucheng Xinniu Technology Co., Ltd. | Pucheng Fattening Second District | Fattening farm | 8 | 336 | 32 |
| Shaanxi Province | Pucheng Xinniu Technology Co., Ltd. | Pucheng Fattening First District | Fattening farm | 8 | 336 | 17 |
| Shaanxi Province | Weinan Xinniu Technology Co., Ltd. | Sangao Fattening Second District | Fattening farm | 8 | 336 | 33 |
| Shaanxi Province | Weinan Xinniu Technology Co., Ltd. | Sangao Fattening Third District | Fattening farm | 8 | 336 | 36 |
| Shaanxi Province | Weinan Xinniu Technology Co., Ltd. | Sangao Fattening First District | Fattening farm | 8 | 336 | 29 |
| Shaanxi Province | Weinan Xinniu Technology Co., Ltd. | Ganquan Fattening farm | Fattening farm | 8 | 336 | 45 |
| Shaanxi Province | Wuqi Xinniu Technology Co., Ltd. | Zhangfangtai Third farm | Fattening farm | 8 | 336 | 43 |
| Shaanxi Province | Wuqi Xinniu Technology Co., Ltd. | Zhangfangtai First farm | Fattening farm | 8 | 336 | 30 |
| Shaanxi Province | Yangling Benxiang Agriculture Industry Group Co., Ltd. Big Gong Pig farm | Bigong Pig farm | Fattening farm | 8 | 336 | 21 |
| Shaanxi Province | Yangling Benxiang Agriculture Industry Group Co., Ltd. Free-Range Center | Benxiang Liquan Free-Ranging Service Department | Fattening farm | 8 | 336 | 29 |
| Sichuan Province | Anyue Xinhao Liuhe Agriculture and Animal Husbandry Co., Ltd. | Jinshan Temple Second farm | Sow farm | 8 | 336 | 31 |
| Sichuan Province | Chengdu Xinjin Xinhao Agriculture and Animal Husbandry Co., Ltd. | Xinjin Pig farm | Sow farm | 8 | 336 | 42 |
| Sichuan Province | Guangan Xinhao Agriculture and Animal Husbandry Co., Ltd. | Longnv Parent Stock farm | Sow farm | 8 | 336 | 47 |
| Sichuan Province | Guangan Xinhao Agriculture and Animal Husbandry Co., Ltd. | Longnv Second farm | Sow farm | 8 | 336 | 20 |
| Sichuan Province | Guangan Xinhao Agriculture and Animal Husbandry Co., Ltd. | Longnv First farm | Sow farm | 8 | 336 | 11 |
| Sichuan Province | Guangyuan Xinhao Agricultural Development Co., Ltd. | Shanxi Parent Stock farm | Sow farm | 8 | 336 | 14 |
| Sichuan Province | Guangyuan Xinhao Agricultural Development Co., Ltd. | Shanxi Second farm | Sow farm | 8 | 336 | 39 |
| Sichuan Province | Guangyuan Xinhao Agricultural Development Co., Ltd. | Shanxi Third farm | Sow farm | 8 | 336 | 25 |
| Sichuan Province | Guangyuan Xinhao Agricultural Development Co., Ltd. | Shanxi First farm | Sow farm | 8 | 336 | 20 |
| Sichuan Province | Guangyuan Xinhao Agricultural Development Co., Ltd. | Shanxi Pig farm Parent Stock farm | Sow farm | 8 | 336 | 43 |
| Sichuan Province | Jiangyou Xinhua Hope Haiboer Pig Breeding Co., Ltd. | Xiping Pig farm | Sow farm | 8 | 336 | 44 |
| Sichuan Province | Langzhong Xinniu Agriculture and Animal Husbandry Technology Co., Ltd. | Deyang Village Parent Stock farm | Sow farm | 8 | 336 | 34 |
| Sichuan Province | Langzhong Xinniu Agriculture and Animal Husbandry Technology Co., Ltd. | Deyang Village Second farm | Sow farm | 8 | 336 | 23 |
| Sichuan Province | Langzhong Xinniu Agriculture and Animal Husbandry Technology Co., Ltd. | Deyang Village First farm | Sow farm | 8 | 336 | 30 |
| Sichuan Province | Langzhong Xinniu Agriculture and Animal Husbandry Technology Co., Ltd. | Deyang Village Breeding Pig farm | Sow farm | 8 | 336 | 39 |
| Sichuan Province | Leshan Xinhao Agriculture and Animal Husbandry Co., Ltd. | Jinfeng Second farm | Sow farm | 8 | 336 | 28 |
| Sichuan Province | Leshan Xinhao Agriculture and Animal Husbandry Co., Ltd. | Jinfeng First farm | Sow farm | 8 | 336 | 53 |
| Sichuan Province | Leshan Xinhao Agriculture and Animal Husbandry Co., Ltd. | Quansheng Pig farm | Sow farm | 8 | 336 | 34 |
| Sichuan Province | Leshan Xinhao Agriculture and Animal Husbandry Co., Ltd. | Dayi Pig farm | Sow farm | 8 | 336 | 37 |
| Sichuan Province | Letian County Ximu Agriculture and Animal Husbandry Co., Ltd. | Shuanghe First farm Parent Stock | Sow farm | 8 | 336 | 42 |
| Sichuan Province | Letian County Ximu Agriculture and Animal Husbandry Co., Ltd. | Shuanghe Second farm Second farm | Sow farm | 8 | 336 | 44 |
| Sichuan Province | Letian County Ximu Agriculture and Animal Husbandry Co., Ltd. | Shuanghe Second farm First farm | Sow farm | 8 | 336 | 14 |
| Sichuan Province | Letian County Ximu Agriculture and Animal Husbandry Co., Ltd. | Shuanghe Second farm | Sow farm | 8 | 336 | 17 |
| Sichuan Province | Letian County Ximu Agriculture and Animal Husbandry Co., Ltd. | Shuanghe Fourth farm | Sow farm | 8 | 336 | 33 |
| Sichuan Province | Letian County Ximu Agriculture and Animal Husbandry Co., Ltd. | Shuanghe First farm Second farm | Sow farm | 8 | 336 | 29 |
| Sichuan Province | Letian County Ximu Agriculture and Animal Husbandry Co., Ltd. | Shuanghe First farm First farm | Sow farm | 8 | 336 | 23 |
| Sichuan Province | Letian County Ximu Agriculture and Animal Husbandry Co., Ltd. | Shuanghe First farm | Sow farm | 8 | 336 | 16 |
| Sichuan Province | Liangshan Xinniu Breeding Co., Ltd. | Mianning Pig farm | Sow farm | 8 | 336 | 34 |
| Sichuan Province | Meishan Ximu Agriculture and Animal Husbandry Co., Ltd. | Wansheng Grandparent Stock farm | Sow farm | 8 | 336 | 53 |
| Sichuan Province | Meishan Ximu Agriculture and Animal Husbandry Co., Ltd. | Wansheng third farm | Sow farm | 8 | 336 | 23 |
| Sichuan Province | Meishan Ximu Agriculture and Animal Husbandry Co., Ltd. | Wansheng Fourth farm | Sow farm | 8 | 336 | 18 |
| Sichuan Province | Meishan Ximu Agriculture and Animal Husbandry Co., Ltd. | Wansheng First farm | Sow farm | 8 | 336 | 30 |
| Sichuan Province | Meishan Ximu Agriculture and Animal Husbandry Co., Ltd. | Wansheng Second farm | Sow farm | 8 | 336 | 18 |
| Sichuan Province | Nanchong Xinhao Agriculture and Animal Husbandry Co., Ltd. | Yaochang First farm | Sow farm | 8 | 336 | 28 |
| Sichuan Province | Nanchong Xinhao Agriculture and Animal Husbandry Co., Ltd. | Yuechi Pig farm | Sow farm | 8 | 336 | 27 |
| Sichuan Province | Rongxian Ximu Agriculture and Animal Husbandry Co., Ltd. | Dafeng Second farm | Sow farm | 8 | 336 | 40 |
| Sichuan Province | Santai Xinhao Agriculture and Animal Husbandry Technology Co., Ltd. | Xiping Pig farm | Sow farm | 8 | 336 | 45 |
| Sichuan Province | Santai Xinhao Agriculture and Animal Husbandry Technology Co., Ltd. | Yongming Pig farm | Sow farm | 8 | 336 | 53 |
| Sichuan Province | Santai Xinhao Agriculture and Animal Husbandry Technology Co., Ltd. | Qingquan Pig farm | Sow farm | 8 | 336 | 31 |
| Sichuan Province | Taian City Xichi Agriculture and Animal Husbandry Co., Ltd. | Sichuan Xinghuo Pig farm | Sow farm | 8 | 336 | 29 |
| Sichuan Province | Yanting Xinhao Agriculture and Animal Husbandry Co., Ltd. | Jianhe Third farm | Sow farm | 8 | 336 | 27 |
| Sichuan Province | Yanting Xinhao Agriculture and Animal Husbandry Co., Ltd. | Yurong Parent Stock farm | Sow farm | 8 | 336 | 42 |
| Sichuan Province | Yanting Xinhao Agriculture and Animal Husbandry Co., Ltd. | Jianhe Isolation Station | Sow farm | 8 | 336 | 49 |
| Sichuan Province | Yanting Xinhao Agriculture and Animal Husbandry Co., Ltd. | Jianhe Fourth farm | Sow farm | 8 | 336 | 27 |
| Sichuan Province | Yanting Xinhao Agriculture and Animal Husbandry Co., Ltd. | Jianhe Second farm | Sow farm | 8 | 336 | 29 |
| Sichuan Province | Yanting Xinhao Agriculture and Animal Husbandry Co., Ltd. | Jianhe First farm | Sow farm | 8 | 336 | 32 |
| Sichuan Province | Yanting Xinhao Agriculture and Animal Husbandry Co., Ltd. | Yurong Third farm | Sow farm | 8 | 336 | 7 |
| Sichuan Province | Yanting Xinhao Agriculture and Animal Husbandry Co., Ltd. | Yurong First farm | Sow farm | 8 | 336 | 19 |
| Sichuan Province | Anyue Xinhao Liuhe Agriculture and Animal Husbandry Co., Ltd. | Xunlong Pig farm | Fattening farm | 8 | 336 | 13 |
| Sichuan Province | Anyue Xinhao Liuhe Agriculture and Animal Husbandry Co., Ltd. | Jinshan Temple First farm | Fattening farm | 8 | 336 | 32 |
| Sichuan Province | Guangan Xinhao Agriculture and Animal Husbandry Co., Ltd. | Diansheng Pig farm | Fattening farm | 8 | 336 | 36 |
| Sichuan Province | Guangan Xinhao Agriculture and Animal Husbandry Co., Ltd. | Longnv Fattening Second farm | Fattening farm | 8 | 336 | 45 |
| Sichuan Province | Guangan Xinhao Agriculture and Animal Husbandry Co., Ltd. | Longnv Fattening Third farm | Fattening farm | 8 | 336 | 20 |
| Sichuan Province | Guangan Xinhao Agriculture and Animal Husbandry Co., Ltd. | Longnv Fattening Fourth farm | Fattening farm | 8 | 336 | 40 |
| Sichuan Province | Guangyuan Xinhao Agricultural Development Co., Ltd. | Shanxi Pig farm Breeding Base | Fattening farm | 8 | 336 | 22 |
| Sichuan Province | Jianwei Xinhao Agriculture and Animal Husbandry Co., Ltd. | Longhua Pig farm | Fattening farm | 8 | 336 | 28 |
| Sichuan Province | Jiangyou Xinhua Hope Haiboer Pig Breeding Co., Ltd. | Dongxing Pig farm | Fattening farm | 8 | 336 | 35 |
| Sichuan Province | Langzhong Xinniu Agriculture and Animal Husbandry Technology Co., Ltd. | Zhongjiang Breeding Base | Fattening farm | 8 | 336 | 33 |
| Sichuan Province | Leshan Xinhao Agriculture and Animal Husbandry Co., Ltd. | Leshan Donglin Pig farm | Fattening farm | 8 | 336 | 38 |
| Sichuan Province | Letian County Ximu Agriculture and Animal Husbandry Co., Ltd. | Xiaojiazhai Pig farm | Fattening farm | 8 | 336 | 35 |
| Sichuan Province | Meishan Ximu Agriculture and Animal Husbandry Co., Ltd. | Xinjin Pig farm | Fattening farm | 8 | 336 | 23 |
| Sichuan Province | Nanchong Xinhao Agriculture and Animal Husbandry Co., Ltd. | Nanchong Jianxing Pig farm | Fattening farm | 8 | 336 | 21 |
| Sichuan Province | Santai Xinhao Agriculture and Animal Husbandry Technology Co., Ltd. | Meihua Pig farm | Fattening farm | 8 | 336 | 21 |
| Sichuan Province | Santai Xinhao Agriculture and Animal Husbandry Technology Co., Ltd. | Santai Jiaxing Pig farm | Fattening farm | 8 | 336 | 23 |
| Sichuan Province | Santai Xinhao Agriculture and Animal Husbandry Technology Co., Ltd. | Santai Zhongxiao Pig farm | Fattening farm | 8 | 336 | 44 |
| Sichuan Province | Santai Xinhao Agriculture and Animal Husbandry Technology Co., Ltd. | Sanwan Pig farm | Fattening farm | 8 | 336 | 29 |
| Sichuan Province | Santai Xinhao Agriculture and Animal Husbandry Technology Co., Ltd. | Shizhu Pig farm | Fattening farm | 8 | 336 | 30 |
| Sichuan Province | Santai Xinhao Agriculture and Animal Husbandry Technology Co., Ltd. | Tongzi Pig farm | Fattening farm | 8 | 336 | 50 |
| Sichuan Province | Santai New Hope Agriculture and Animal Husbandry Technology Co., Ltd. | Santai Nongmu Dongbao Pig farm | Fattening farm | 8 | 336 | 47 |
| Sichuan Province | Santai New Hope Agriculture and Animal Husbandry Technology Co., Ltd. | Tongzi First farm | Fattening farm | 8 | 336 | 19 |
| Sichuan Province | Taian City Xinchih Agriculture and Animal Husbandry Co., Ltd. Shehong Xinchih | Shehong Yongcheng Pig farm | Fattening farm | 8 | 336 | 39 |
| Sichuan Province | Tianjin New Liu Agriculture and Animal Husbandry Technology Co., Ltd. | Pengshan Xiejia Breeding Base | Fattening farm | 8 | 336 | 20 |
| Sichuan Province | Tianjin New Liu Agriculture and Animal Husbandry Technology Co., Ltd. | Ya'an Free-Range Service Department | Fattening farm | 8 | 336 | 31 |
| Sichuan Province | Yanting Xinhao Agriculture and Animal Husbandry Co., Ltd. | Yanting Zhongxiao Pig farm | Fattening farm | 8 | 336 | 27 |
| Sichuan Province | Chongqing Pengshui New Liu Agriculture and Animal Husbandry Technology Co., Ltd. | Nanchong Free-range Service Department | Fattening farm | 8 | 336 | 24 |
| Tianjin Municipality | Tianjin New Hope Liuhe Agriculture and Animal Husbandry Technology Co., Ltd. | Red Star No.5 farm | Sow farm | 8 | 336 | 33 |
| Tianjin Municipality | Tianjin New Hope Liuhe Agriculture and Animal Husbandry Technology Co., Ltd. | Red Star No.3 farm | Sow farm | 8 | 336 | 37 |
| Tianjin Municipality | Tianjin New Hope Liuhe Agriculture and Animal Husbandry Technology Co., Ltd. | Red Star No.4 farm | Sow farm | 8 | 336 | 41 |
| Tianjin Municipality | Tianjin New Hope Liuhe Agriculture and Animal Husbandry Technology Co., Ltd. | Red Star No.2 farm | Sow farm | 8 | 336 | 17 |
| Tianjin Municipality | Tianjin New Hope Liuhe Agriculture and Animal Husbandry Technology Co., Ltd. | Red Star No. 1 farm | Sow farm | 8 | 336 | 29 |
| Tianjin Municipality | Tianjin New Liu Agriculture and Animal Husbandry Technology Co., Ltd. | Nanheshun No.8 farm | Fattening farm | 8 | 336 | 30 |
| Tianjin Municipality | Tianjin New Liu Agriculture and Animal Husbandry Technology Co., Ltd. | Nanheshun No.2 farm | Fattening farm | 8 | 336 | 19 |
| Tianjin Municipality | Tianjin New Liu Agriculture and Animal Husbandry Technology Co., Ltd. | Nanheshun No.9 farm | Fattening farm | 8 | 336 | 38 |
| Tianjin Municipality | Tianjin New Liu Agriculture and Animal Husbandry Technology Co., Ltd. | Nanheshun No.6 farm | Fattening farm | 8 | 336 | 14 |
| Tianjin Municipality | Tianjin New Liu Agriculture and Animal Husbandry Technology Co., Ltd. | Nanheshun No.7 farm | Fattening farm | 8 | 336 | 14 |
| Tianjin Municipality | Tianjin New Liu Agriculture and Animal Husbandry Technology Co., Ltd. | Nanheshun No.18 farm | Fattening farm | 8 | 336 | 31 |
| Tianjin Municipality | Tianjin New Liu Agriculture and Animal Husbandry Technology Co., Ltd. | Nanheshun No.17 farm | Fattening farm | 8 | 336 | 20 |
| Tianjin Municipality | Tianjin New Liu Agriculture and Animal Husbandry Technology Co., Ltd. | Nanheshun No.13 farm | Fattening farm | 8 | 336 | 42 |
| Tianjin Municipality | Tianjin New Liu Agriculture and Animal Husbandry Technology Co., Ltd. | Nanheshun No.11 farm | Fattening farm | 8 | 336 | 17 |
| Tianjin Municipality | Tianjin New Liu Agriculture and Animal Husbandry Technology Co., Ltd. | Nanheshun No.5 farm | Fattening farm | 8 | 336 | 30 |
| Tianjin Municipality | Tianjin New Liu Agriculture and Animal Husbandry Technology Co., Ltd. | Nanheshun No.1 farm | Fattening farm | 8 | 336 | 53 |
| Tianjin Municipality | Tianjin New Liu Agriculture and Animal Husbandry Technology Co., Ltd. | Nanheshun No.12 farm | Fattening farm | 8 | 336 | 28 |
| Tianjin Municipality | Tianjin New Liu Agriculture and Animal Husbandry Technology Co., Ltd. | Nanheshun No.14 farm | Fattening farm | 8 | 336 | 23 |
| Tianjin Municipality | Tianjin New Liu Agriculture and Animal Husbandry Technology Co., Ltd. | Nanheshun No.10 farm | Fattening farm | 8 | 336 | 46 |
| Yunnan Province | Taian City Xinchih Agriculture and Animal Husbandry Co., Ltd. | Yiliang Xinchih Pig farm | Sow farm | 8 | 336 | 47 |
| Yunnan Province | Taian City Xinchih Agriculture and Animal Husbandry Co., Ltd. | Yongsheng Xinchih Pig farm | Sow farm | 8 | 336 | 79 |
| Yunnan Province | Taian City Xinchih Agriculture and Animal Husbandry Co., Ltd. | Yuxi Ruirong No. 2 farm | Sow farm | 8 | 336 | 92 |
| Yunnan Province | Xuanwei New Liu Agriculture and Animal Husbandry Technology Co., Ltd. | Tongdu Breeding farm | Fattening farm | 8 | 336 | 84 |
| Yunnan Province | Xuanwei New Liu Agriculture and Animal Husbandry Technology Co., Ltd. | Xuanwei Service Department (Xuanwei New 6) | Fattening farm | 8 | 336 | 69 |
| Yunnan Province | Yiliang New Liu Agriculture and Animal Husbandry Technology Co., Ltd. | Tuobuka Breeding farm | Fattening farm | 8 | 336 | 80 |
| Yunnan Province | Yunnan New Hope Liuhe Breeding Co., Ltd. | Yunnan New Liu Xuanwei Service Department | Fattening farm | 8 | 336 | 97 |
| Yunnan Province | Yunnan New Hope Liuhe Breeding Co., Ltd. | Yunnan New Liu Yiliang Service Department | Fattening farm | 8 | 336 | 74 |
| Yunnan Province | Yunnan New Hope Liuhe Breeding Co., Ltd. | Yunnan New Liu Pig Raising Service Department | Fattening farm | 8 | 336 | 89 |
| Zhejiang Province | Ningbo New Hope Liuhe Agriculture and Animal Husbandry Co., Ltd. | Chunhu No.3 farm | Sow farm | 8 | 336 | 13 |
| Zhejiang Province | Ningbo New Hope Liuhe Agriculture and Animal Husbandry Co., Ltd. | Chunhu No.2 farm | Sow farm | 8 | 336 | 13 |
| Zhejiang Province | Ningbo New Hope Liuhe Agriculture and Animal Husbandry Co., Ltd. | Chunhu No.1 farm | Sow farm | 8 | 336 | 6 |
| Zhejiang Province | Zhejiang Xindongwan Agricultural Development Co., Ltd. | Xindongwan No.1 farm | Sow farm | 8 | 336 | 17 |
| Zhejiang Province | Ningbo New Hope Liuhe Agriculture and Animal Husbandry Co., Ltd. | Chunhu No.9 farm | Fattening farm | 8 | 336 | 3 |
| Zhejiang Province | Ningbo New Hope Liuhe Agriculture and Animal Husbandry Co., Ltd. | Chunhu No.6 farm | Fattening farm | 8 | 336 | 17 |
| Zhejiang Province | Ningbo New Hope Liuhe Agriculture and Animal Husbandry Co., Ltd. | Chunhu No.4 farm | Fattening farm | 8 | 336 | 14 |
| Zhejiang Province | Taizhou City New Hope Liuhe Agriculture and Animal Husbandry Co., Ltd. | Rui'an Fattening farm | Fattening farm | 8 | 336 | 23 |
| Zhejiang Province | Changxing Peace Huatong Livestock Co., Ltd. | Shangyang No.1 farm | Fattening farm | 8 | 336 | 10 |
| Zhejiang Province | Changxing Peace Huatong Livestock Co., Ltd. | Shangyang No.2 farm | Fattening farm | 8 | 336 | 4 |
| Zhejiang Province | Changxing Peace Huatong Livestock Co., Ltd. | Shangyang No.5 farm | Fattening farm | 8 | 336 | 25 |
| Zhejiang Province | Changxing Peace Huatong Livestock Co., Ltd. | Shangyang No.3 farm | Fattening farm | 8 | 336 | 5 |
| Zhejiang Province | Zhejiang Yihai Agricultural Development Co., Ltd. | Duzhe No. 7 farm | Fattening farm | 8 | 336 | 3 |
| Chongqing Municipality | Chongqing New Hope Pig Resource Development Co., Ltd. | Rongchang Pig farm | Sow farm | 8 | 336 | 23 |
| Chongqing Municipality | Chongqing Pengshui New Liu Agriculture and Animal Husbandry Technology Co., Ltd. | Pengshui Raising Service Department | Fattening farm | 8 | 336 | 14 |
| Chongqing Municipality | Chongqing Pengshui New Liu Agriculture and Animal Husbandry Technology Co., Ltd. | Tongnan Chenxiu farm | Fattening farm | 8 | 336 | 9 |
| Chongqing Municipality | Chongqing New Hope Pig Resource Development Co., Ltd. | Pengshui Raising Service Department | Fattening farm | 8 | 336 | 4 |
| Chongqing Municipality | Chongqing New Hope Pig Resource Development Co., Ltd. | Rongchang Hongde Pig farm | Fattening farm | 8 | 336 | 21 |
