# Supplementary material for: Epidemiological analysis of porcine reproductive and respiratory syndrome viruses in 2020–2023 in China and the impact of serum acclimatization on production performance of sows farm
Source: Front Vet Sci. 2025 Jun 23;12:1614039. doi: 10.3389/fvets.2025.1614039 (PMC12229867; doi:10.3389/fvets.2025.1614039)
Supplement: Supplementary file 4 [file Table_3.docx]

The information of blood and throat swab samples from July 2021 to June 2023 in 24 provinces and municipalities was listed in Table S3.

**Table S3.** **The information of blood and throat swab samples**

| **Sampling** **time** | **Blood samples** | | | **Throat swap samples** | | |
| --- | --- | --- | --- | --- | --- | --- |
|  | **The positive number** | **The total number** | **Positive rate** | **The positive number** | **The total number** | **Positive rate** |
| Jul 2021 | 1691 | 10329 | 16.37% | 190 | 2193 | 8.66% |
| Aug 2021 | 1062 | 9718 | 10.93% | 526 | 6992 | 7.52% |
| Sep 2021 | 2038 | 11839 | 17.21% | 860 | 8411 | 10.22% |
| Oct 2021 | 1328 | 11119 | 11.94% | 1050 | 10893 | 9.64% |
| Nov 2021 | 1404 | 10630 | 13.21% | 1407 | 10001 | 14.07% |
| Dec 2021 | 1534 | 11264 | 13.62% | 1390 | 10389 | 13.38% |
| Jan 2022 | 809 | 8229 | 9.83% | 1023 | 8113 | 12.61% |
| Feb 2022 | 862 | 7913 | 10.89% | 1106 | 9416 | 11.75% |
| Mar 2022 | 1233 | 9613 | 12.83% | 881 | 10668 | 8.26% |
| Apr 2022 | 1113 | 10355 | 10.75% | 986 | 11187 | 8.81% |
| May 2022 | 1926 | 18712 | 10.29% | 744 | 11438 | 6.50% |
| Jun 2022 | 1486 | 22278 | 6.67% | 693 | 9572 | 7.24% |
| Jul 2022 | 1284 | 21652 | 5.93% | 606 | 11251 | 5.39% |
| Aug 2022 | 2711 | 30983 | 8.75% | 564 | 11002 | 5.13% |
| Sep 2022 | 3281 | 30762 | 10.67% | 1181 | 15239 | 7.75% |
| Oct 2022 | 4923 | 29464 | 16.71% | 1515 | 17745 | 8.54% |
| Nov 2022 | 3673 | 28522 | 12.88% | 2840 | 20870 | 13.61% |
| Dec 2022 | 3399 | 33508 | 10.14% | 3794 | 35810 | 10.59% |
| Jan 2023 | 2241 | 18381 | 12.19% | 2927 | 30679 | 9.54% |
| Feb 2023 | 3729 | 28679 | 13.00% | 3234 | 28812 | 11.22% |
| Mar 2023 | 4697 | 32990 | 14.24% | 3664 | 41663 | 8.79% |
| Apr 2023 | 3953 | 36056 | 10.96% | 2759 | 36848 | 7.49% |
| May 2023 | 3041 | 41685 | 7.30% | 3252 | 41505 | 7.84% |
| Jun 2023 | 1465 | 21030 | 6.97% | 1177 | 18091 | 6.51% |
