# Supplementary material for: Epidemiological analysis of porcine reproductive and respiratory syndrome viruses in 2020–2023 in China and the impact of serum acclimatization on production performance of sows farm
Source: Front Vet Sci. 2025 Jun 23;12:1614039. doi: 10.3389/fvets.2025.1614039 (PMC12229867; doi:10.3389/fvets.2025.1614039)
Supplement: Supplementary file 5 [file Table_4.docx]

The information for analyzing the number of pig farms with PRRS From January 2021 to December 2022 was listed in Table S4. The total of 718 pig farms affiliated to 147 companies were statistically analyzed. All sow farms are the standard farm with a scale of 3,000. The scale of fattening farms are 1,500. PRRS vaccine for sow farms is administered three times a year. The fattening farms are not immunized with PRRS vaccine.

**Table S4. The information for** **analyzing the number of pig farms with PRRS**

| **Jan 2021** | | **Feb 2021** | | **Mar 2021** | |
| --- | --- | --- | --- | --- | --- |
| **Company (Co., Ltd.)** | **Type of pig farm** | **Company (Co., Ltd.)** | **Type of pig farm** | **Company (Co., Ltd.)** | **Type of pig farm** |
| Dongying Xinhao | Sow farm | Anyang Xinliu | Sow farm | Anyang Xinliu | Sow farm |
| Hengnan Muyun | Fattening farm | Zhangjiakou Xinwang | Sow farm | Laizhou Xinhai | Sow farm |
| Lezhi Xinhai | Fattening farm | Ningming Xinhao | Fattening farm | Ningming Xinhao | Sow farm |
| Longzhou Xinhao | Fattening farm | Guanling Xinhai | Fattening farm | Shibing Xinliu | Sow farm |
| Dezhou Xinhao | Sow farm | Shanxian Xinliu | Sow farm | Weinan Xinliu | Sow farm |
| Wuhe Xinhai | Fattening farm | Dongying Xinhao | Fattening farm | Hezhou Xinhao | Sow farm |
| Tangshan Xinhao | Fattening farm | Yantai Xinhao | Sow farm | Liaocheng Xinhao | Sow farm |
| Ningming Xinhao | Fattening farm | Tangshan Xinhao | Fattening farm | Yingcheng Xinhao | Sow farm |
| Gaomi Xinliu | Sow farm | Hezhou Xinhao | Sow farm | Wuhe Xinhai | Sow farm |
| Ji'an Xinchi | Sow farm | Laixi Xinliu | Fattening farm | Heishan Xinliu | Sow farm |
| Yantai Xinhao | Sow farm | Wannian Xinhai | Fattening farm | Laizhou Xinhai | Sow farm |
| Lezhi Xinhai | Fattening farm | Pengshui Xinliu | Fattening farm | Yantai Xinhao | Sow farm |
| Hezhou Xinhao | Fattening farm | Sichuan Xinhao | Sow farm | Wuhe Xinhai | Sow farm |
| Guang'an Xinhao | Sow farm | Chenzhou Xinhao | Fattening farm | Shibing Xinliu | Sow farm |
| Hubei Xinhao | Fattening farm | Huanghua Xinhao | Sow farm | Linyi Xinhao | Sow farm |
| Shuozhou Xinhao | Sow farm | Huanghua Xinhao | Sow farm | Tangshan Xinhao | Fattening farm |
| Shibing Xinliu | Sow farm | Wannian Xinhai | Sow farm | Huanghua Xinhao | Sow farm |
| Zhangjiakou Xinwang | Sow farm | Weinan Xinliu | Sow farm | Zhangwu Xinwang | Sow farm |
| Weinan Xinliu | Sow farm | Xinmin New Hope | Sow farm | Ruyuan Xinhao | Sow farm |
| Heishan Woniou | Sow farm | HeishanWoniu | Sow farm | HeishanWoniu | Sow farm |
| Nanning Xinhao | Fattening farm | Guangan Xinhao | Fattening farm | Linyi Xinhao | Sow farm |
| Ruyuan Xinhao | Fattening farm | Huanghua Xinhao | Fattening farm | Guigang Xinliu | Sow farm |
| Chenzhou Xinhao | Sow farm | Hubei Xinhao | Fattening farm | Wuhe Xinhai | Sow farm |
| Xinmin Xinwang | Sow farm | Shanxian Xinhao | Sow farm | Raoyang Xinhao | Sow farm |
| Hengnan Muyun | Fattening farm | Hezhou Xinhao | Fattening farm | Danxian Xinliu | Sow farm |
| Hubei Xinhao | Sow farm | Suining Xinliu | Sow farm | Jiexi Xinliu | Fattening farm |
| Guang'an Xinhao | Sow farm | Junan Breeding Pig | Sow farm | Wulian Xinhao | Sow farm |
| Hainan Xinliu | Sow farm | Gaomi Xinliu | Sow farm | Zhangwu Xinwang | Sow farm |
| Hezhou Xinhao | Fattening farm | Laiyang Xinmu | Sow farm | Zhangwu Xinwang | Sow farm |
| Weihui Xinchi | Sow farm | Zhaoqing Xinhao | Sow farm | Jiangxi Xinliu | Sow farm |
| Weinan Xinliu | Sow farm | Lingbao Xinliu | Sow farm | Chenzhou Xinhao | Fattening farm |
| Dingzhou Xinhao | Sow farm | Linyi Xinhao | Sow farm | Wuhe Xinhai | Sow farm |
| Lingbao Xinliu | Sow farm | Xiangyang Xinhao | Sow farm | Ningbo Xinhai | Sow farm |
| Guang'an Xinhao | Sow farm | Dongying Xinhao | Sow farm | Laizhou Xinhai | Sow farm |
| Longzhou Xinhao | Sow farm | Raoyang Xinhao | Sow farm | Tongliao Xinhao | Sow farm |
| Guanling Xinhai | Sow farm | Linyi Xinhao | Sow farm | Zhuocheng Xinliu | Sow farm |
| Dongying Xinhao | Sow farm | Shibing Xinliu | Sow farm | Rugao Xinhao | Fattening farm |
| Longzhou Xinhao | Sow farm | Kangping New Hope | Sow farm | Hezhou Xinhao | Sow farm |
| Xiajiang Xinliu | Sow farm | Huanghua Xinhao | Sow farm | Tianxin Xinliu | Sow farm |
| Shuozhou Xinhao | Sow farm | Zhangwu New Hope | Sow farm | Yantai Xinhao | Sow farm |
| Luocheng Xinhao | Fattening farm | Yingcheng Xinhao | Fattening farm | Dongying Xinhao | Sow farm |
| Zhangjiakou Xinwang | Sow farm | Anqiu Xinhai | Sow farm | Jingxian Xinhao | Sow farm |
| Ruyuan Xinhao | Fattening farm | Ruyuan Xinhao | Fattening farm | Santai Agriculture and Animal Husbandry | Sow farm |
| Xuanwei Xinliu | Sow farm | Dongying Xinhao | Sow farm | Dacheng Xinhao | Sow farm |
| Zhu Cheng Xinliu | Sow farm | Yantai Xinhao | Sow farm | Guanling Xinhai | Sow farm |
| Liaocheng Xinhao | Fattening farm | Zoucheng Xinliu | Sow farm | Donghai Xinhao | Sow farm |
| Shangcai Xinliu | Fattening farm | Wulian Xinhao | Sow farm | Chongren Xinhai | Sow farm |
| Dacheng Xinhao | Sow farm | Xiangyang Xinhao | Sow farm | Guang'an Xinhao | Sow farm |
| Sichuan Xinhao | Sow farm | Anyang Xinliu | Sow farm | Donghai Xinhao | Sow farm |
| Neiqiu Xinliu | Sow farm | Lezhi Xinhai | Fattening farm | Xinmin Xinwang | Sow farm |
| Dongying Xinhao | Sow farm | Shanxian Xinhao | Sow farm | Hubei Xinhao | Sow farm |
| Anyue Xing Xin Xin | Sow farm | Guangan Xinhao | Sow farm | Huanghua Xinhao | Sow farm |
| Wuqi Xinliu | Sow farm | Pengshui Xinliu | Fattening farm | Guangyuan Xinhao | Sow farm |
| Leshan Agricultural and Animal Husbandry | Fattening farm | Sichuan Xinhao | Sow farm | Guanling Xinhai | Fattening farm |
| Linshu Breeding Pig | Sow farm | Nanning Xinhao | Fattening farm | Laizhou Xinhai | Sow farm |
| Kangping Xinwang | Sow farm | Guangan Xinhao | Fattening farm | Junan Breeding Pigs | Sow farm |
| Zhejiang Xinhai | Sow farm | Zhangjiakou New Hope | Sow farm | Santai Agriculture and Animal Husbandry | Sow farm |
| Meishan Xinhai | Fattening farm | Dongying Xinhao | Sow farm | Laizhou Xinhai | Sow farm |
| Nangong Xinhao | Sow farm | Huanghua Xinhao | Sow farm | Dongying Xinhao | Sow farm |
| Zhejiang Xinhai | Sow farm | Zoucheng Xinliu | Sow farm | Anyang Xinliu | Sow farm |
| Heishan Xinliu | Sow farm | Hubei Xinhao | Sow farm | Tongliao Xinhao | Sow farm |
| Dongying Xinhao | Sow farm | Nangong Xinhao | Sow farm | Lianjiang Xinhao | Fattening farm |
| Laixi Xinliu | Sow farm | Hezhou Xinhao | Fattening farm | Laizhou Xinhai | Sow farm |
| Zhu Cheng Xinliu | Sow farm | Qingyuan Xinhao | Fattening farm | Sichuan Xinhao | Sow farm |
| Gaomi Xinliu | Sow farm | Lezhi Xinhai | Fattening farm | Hainan Agricultural Reclamation | Sow farm |
| Gansu Xinhao | Sow farm | Jian New Gallop | Sow farm | Chenzhou Xinhao | Sow farm |
| Xinmin Xinwang | Sow farm | Nantong Xinhai | Sow farm | Weinan Xinliu | Sow farm |
| Wulian Xinhao | Sow farm | Guangan Xinhao | Sow farm | Tongliao Xinhao | Sow farm |
| Laixi Xinliu | Sow farm | Yantai Xinhao | Sow farm | Longhui Xinhai | Fattening farm |
| Anyang Xinliu | Sow farm | Zhengzhou Quansheng | Sow farm | Dongying Xinhao | Sow farm |
| Danxian Xinhao | Sow farm | Dongying Xinhao | Sow farm | Donghai Xinhao | Sow farm |
| Ruzhou Quansheng | Sow farm | Laixi Xinliu | Sow farm | Heishan Xinliu | Fattening farm |
| Donghai Xinhao | Sow farm | Yantai Xinhao | Sow farm | Linshu Breeding Pigs | Sow farm |
| Binzhou Xinhai | Sow farm | Shuozhou Xinhao | Fattening farm | Dongying Xinhao | Sow farm |
| Heishan Xinliu | Sow farm | Tongcheng Xinliu | Sow farm | Kangping Xinwang | Sow farm |
| Yingcheng Xinhao | Fattening farm | Gaomi Xinliu | Sow farm | Laixi Xinliu | Sow farm |
| Fuping Xinliu | Sow farm | Laixi Xinliu | Sow farm | Anqiu Xinhai | Sow farm |
| Tangshan Xinhao | Fattening farm | Tongliao Xinhao | Sow farm | Tangshan Xinhao | Sow farm |
| Danxian Xinhao | Sow farm | Longzhou Xinhao | Fattening farm | Anyang Xinliu | Sow farm |
| Huanghua Xinhao | Fattening farm | Donghai Xinhao | Sow farm | Anyang Xinliu | Sow farm |
| Fuping Xinliu | Fattening farm | Santai Agricultural and Animal Husbandry | Sow farm | Raoyang Xinhao | Sow farm |
| Raoyang Xinhao | Sow farm | Xinmin New Hope | Sow farm | Laixi Xinliu | Sow farm |
| Neiqiu Xinliu | Sow farm | Zoucheng Xinliu | Sow farm | Shuozhou Xinhao | Fattening farm |
| Luocheng Xinhao | Fattening farm | Binzhou Xinhai | Sow farm | Santai Agriculture and Animal Husbandry | Sow farm |
| Dongying Xinhao | Fattening farm | Guangan Xinhao | Sow farm | Chenzhou Xinhao | Sow farm |
| Zhu Cheng Xinliu | Sow farm | Shangcai Xinliu | Fattening farm | Gaomi Xinliu | Sow farm |
| Heishan Xinliu | Sow farm | Guangan Xinhao | Fattening farm | Huanghua Xinhao | Sow farm |
| Yingtan Xinliu | Sow farm | Tongliao Xinhao | Sow farm | Danxian Xinhao | Sow farm |
| Longhui Xinhai | Fattening farm | Anyang Xinliu | Sow farm | Xiajin Agriculture and Animal Husbandry | Sow farm |
| Sichuan Xinhao | Sow farm | Tangshan Xinhao | Fattening farm | Yantai Xinhao | Sow farm |
| Nanning Xinliu | Sow farm | Shanxian Xinhao | Sow farm | Tongliao Xinhao | Sow farm |
| Jingxian Xinhao | Sow farm | Heishan Xinliu | Sow farm | Donghai Xinhao | Sow farm |
| Liaoning Xinwang | Sow farm | Longzhou Xinhao | Fattening farm | Laizhou Xinhai | Sow farm |
| Laixi Xinliu | Fattening farm | Yingcheng Xinhao | Sow farm | Dingzhou Xinhao | Sow farm |
| Heishan Xinliu | Sow farm | Zhenjiang Xinhai | Fattening farm | Yantai Xinhao | Sow farm |
| Gaotang Xinhao | Sow farm | Yantai Xinhao | Sow farm | Tongliao Xinhao | Sow farm |
| Yingcheng Xinhao | Sow farm | Gansu Xinhao | Sow farm | Hezhou Xinhao | Sow farm |
| Dongying Xinhao | Fattening farm | Junan Breeding Pig | Sow farm | Kangping Xinwang | Sow farm |
| Dongying Xinhao | Sow farm | Anyang Xinliu | Sow farm | Wannian Xinhai | Sow farm |
| Rugao Xinhao | Sow farm | Xiajin Agricultural and Animal Husbandry | Sow farm | Tongcheng Xinliu | Sow farm |
| Chenzhou Xinhao | Fattening farm | Raoyang Xinhao | Sow farm | Guangyuan Xinhao | Sow farm |
| Jizhou Xinhao | Fattening farm | Hainan Xinliu | Sow farm | Dingzhou Xinhao | Sow farm |
| Taian Xinliu | Fattening farm | Binzhou Xinhai | Sow farm | Xiangyang Xinhao | Sow farm |
| Gansu Xinliu | Fattening farm | Sichuan Xinhao | Sow farm | Shangcai Xinliu | Fattening farm |
| Dongying Xinhao | Sow farm | Anyue Xing Xin Xin | Sow farm | Huanghua Xinhao | Sow farm |
| Binzhou Xinhai | Sow farm | Binzhou Xinhai | Sow farm | Chenzhou Xinhao | Sow farm |
| Shuozhou Xinhao | Sow farm | Chenzhou Xinhao | Fattening farm | Guangyuan Xinhao | Sow farm |
| Neiqiu Xinliu | Sow farm | Longzhou Xinhao | Sow farm | Xiangyang Xinhao | Sow farm |
| Taian Xinliu | Fattening farm | Linyi New Pig | Sow farm | Gaotang Xinhao | Sow farm |
| Kangping Xinwang | Sow farm | Dongying Xinhao | Sow farm | Chenzhou Xinhao | Sow farm |
| Suining Xinliu | Sow farm | Taian Xinliu | Fattening farm | Anyang Xinliu | Sow farm |
| Laixi Xinliu | Sow farm | Dongying Xinhao | Sow farm | Hainan Agricultural Reclamation | Fattening farm |
| Yichun Xinwang | Fattening farm | Donghai Xinhao | Sow farm | Laizhou Xinhai | Sow farm |
| Hubei Xinhao | Sow farm | Dingzhou Xinhao | Sow farm | Dingzhou Xinhao | Sow farm |
| Jingxian Xinhao | Sow farm | Wuhe Xinhai | Sow farm | Letang Xinhai | Sow farm |
| Gansu Xinhao | Sow farm | Weinan Xinliu | Sow farm | Sichuan Xinhao | Sow farm |
| Chenzhou Xinhao | Fattening farm | Dongying Xinhao | Sow farm | Caoxian Xinhao | Sow farm |
| Hengnan Muyun | Sow farm | Xinmin New Hope | Sow farm | Guang'an Xinhao | Sow farm |
| Yingcheng Xinhao | Fattening farm | Sichuan Xinhao | Sow farm | Tongliao Xinhao | Fattening farm |
| Dongying Xinhao | Sow farm | Hainan Agricultural Reclamation | Sow farm | Guang'an Xinhao | Sow farm |
| Nanning Xinhao | Sow farm | Nanning Xinliu | Fattening farm | Danxian Xinliu | Sow farm |
| Zhaoqing Xinhao | Fattening farm | Nanning Xinliu | Sow farm | Zhaoqing Xinhao | Sow farm |
| Yantai Xinhao | Sow farm | Dingzhou Xinhao | Sow farm | Laixi Xinliu | Sow farm |
| Neiqiu Xinliu | Sow farm | Liaocheng Xinhao | Fattening farm | Dongying Xinhao | Sow farm |
| Neiqiu Xinliu | Sow farm | Anyang Xinliu | Sow farm | Dongying Xinhao | Sow farm |
| Xiangyang Xinhao | Sow farm | Heishan Xinliu | Sow farm | Dongying Xinhao | Sow farm |
| Yantai Xinhao | Sow farm | Xianyang Yongxiang | Sow farm | Gansu Xinhao | Sow farm |
| Nanning Xinhao | Sow farm | Jingxian Xinhao | Sow farm | Jingxian Xinhao | Sow farm |
| Dongying Xinhao | Sow farm | Yantai Xinhao | Sow farm | Taian Xinliu | Fattening farm |
| Sichuan Xinhao | Sow farm | Gaotang Xinhao | Sow farm | Xinmin Xinwang | Sow farm |
| Sichuan Xinhao | Sow farm | Leshan Agricultural and Animal Husbandry | Fattening farm | Yantai Xinhao | Sow farm |
| Gansu Xinhao | Fattening farm | Fuping Xinliu | Sow farm | Raoyang Xinhao | Sow farm |
| Laizhou Xinhai | Sow farm | Tongliao Xinhao | Sow farm | Anyang Xinliu | Sow farm |
| Laizhou Xinhai | Sow farm | Anyang Xinliu | Sow farm | Liaocheng Xinhao | Sow farm |
| Neiqiu Xinliu | Sow farm | Laixi Xinliu | Sow farm | Guang'an Xinhao | Sow farm |
| Dongying Xinhao | Sow farm | Xinmin New Hope | Sow farm | Laiyang Xinmu | Sow farm |
| Anyang Xinliu | Sow farm | Hengnan Pastoral Farming | Sow farm | Zhaoqing Xinhao | Sow farm |
| Gaomi Xinliu | Sow farm | Taian Xinliu | Fattening farm | Zhejiang Xinhai | Sow farm |
| Luocheng Xinhao | Fattening farm | Ruyuan Xinhao | Fattening farm | Laixi Xinliu | Sow farm |
| Neiqiu Xinliu | Sow farm | Gansu Xinhao | Fattening farm | Binzhou Xinhai | Sow farm |
| Yingcheng Xinhao | Sow farm | Donghai Xinhao | Sow farm | Zhengzhou Quansheng | Sow farm |
| Danxian Xinhao | Sow farm | Guanling Xinhai | Sow farm | Gansu Xinhao | Sow farm |
| Gansu Xinhao | Sow farm | Anyang Xinliu | Sow farm | Binzhou Xinhai | Sow farm |
| Weinan Xinliu | Sow farm | Sichuan Xinhao | Sow farm | Fucheng Xinhao | Sow farm |
| Longzhou Xinhao | Sow farm | Shuozhou Xinhao | Sow farm | Dongying Xinhao | Sow farm |
| Linshu Breeding Pig | Sow farm | Dongying Xinhao | Sow farm | Nanning Xinliu | Sow farm |
| Santai Agricultural and Animal Husbandry | Sow farm | Zhaoqing Xinhao | Fattening farm | Pengshui Xinliu | Sow farm |
| Gaomi Xinliu | Sow farm | Jizhou Xinhao | Fattening farm | Heishan Xinliu | Sow farm |
| Hezhou Xinhao | Fattening farm | Anhui Xinliu | Sow farm | Binzhou Xinhai | Sow farm |
| Laiyang New Animal Husbandry | Sow farm | Dongying Xinhao | Sow farm | Liaocheng Xinhao | Sow farm |
| Yantai Xinhao | Sow farm | Yantai Xinhao | Sow farm | Guangyuan Xinhao | Sow farm |
| Heyang Agricultural and Animal Husbandry | Fattening farm | Zhejiang Xinhai | Sow farm | Heishan Xinliu | Sow farm |
| Tongliao Xinhao | Sow farm | Lezhi Xinhai | Fattening farm | Raoyang Xinhao | Sow farm |
| Yingcheng Xinhao | Sow farm | Guangan Xinhao | Sow farm | Lingbao Xinliu | Sow farm |
| Liaoning Xintao Prospect | Sow farm | Dacheng Xinhao | Sow farm | Jingxian Xinhao | Sow farm |
| Liaoning Xintao Prospect | Sow farm | Laiyang Xinmu | Sow farm | Fuping Xinliu | Sow farm |
| Yantai Xinhao | Sow farm | Dongying Xinhao | Sow farm | Taian Xinliu | Sow farm |
| Nanning Xinhao | Sow farm | Laizhou Xinhai | Sow farm | Guigang Xinliu | Sow farm |
| Dongying Xinhao | Sow farm | Laizhou Xinhai | Sow farm | Sichuan Xinhao | Sow farm |
| Zhangjiakou Xinwang | Sow farm | Laixi Xinliu | Sow farm | Dongying Xinhao | Sow farm |
| Fuxin Xinwang | Sow farm | Hezhou Xinhao | Fattening farm | Dongying Xinhao | Sow farm |
| Anhui Xinliu | Sow farm | Heyang Agricultural and Animal Husbandry | Fattening farm | Dingzhou Xinhao | Sow farm |
| Guanling Xinhai | Sow farm | Nanchong Xinhao | Sow farm | Laiyang Xinmu | Sow farm |
| Ruyuan Xinhao | Fattening farm | Donghai Xinhao | Sow farm | Sichuan Xinhao | Sow farm |
| Dingzhou Xinhao | Sow farm | Gaomi Xinliu | Sow farm | Hainan Xinliu | Sow farm |
| Shuozhou Xinhao | Sow farm | Longzhou Xinhao | Sow farm | Laizhou Xinha | Sow farm |
| Qingfeng Xinliu | Sow farm | Yingcheng Xinhao | Sow farm | Yichun Xiwang | Sow farm |
| Dingzhou Xinhao | Sow farm | Dingzhou Xinhao | Sow farm | Xinmin Xiwang | Sow farm |
| Laixi Xinliu | Sow farm | Liaoning Xintao Wang | Sow farm | Dongying Xinhao | Fattening farm |
| Tongliao Xinhao | Sow farm | Liaoning Xintao Wang | Sow farm | Huanghua Xinhao | Sow farm |
| Shanxian Xinhao | Sow farm | Laixi Xinliu | Sow farm | Shanxian Xinliu | Sow farm |
| Dongying Xinhao | Sow farm | Guangan Xinhao | Sow farm | Yantai Xinhao | Sow farm |
| Rongchang Pig Farm | Sow farm | Guangyuan Xinhao | Sow farm | Heyang Nongmu | Fattening farm |
| Wuhe Xinhai | Sow farm | Wuhe Xinhai | Sow farm | Hengnan Muyun | Sow farm |
| Yantai Xinhao | Sow farm | Gaomi Xinliu | Sow farm | Jizhou Xinhao | Sow farm |
| Longzhou Xinhao | Sow farm | Chenzhou Xinhao | Fattening farm | Anhui Xinliu | Sow farm |
| Liaoning Xintao Wang | Sow farm | Luocheng Xinhao | Fattening farm | Wuhe Xinha | Sow farm |
| Zhoucheng Xinliu | Sow farm | Shanxian Xinhao | Sow farm | Leting Xinha | Sow farm |
| Shanxian Xinhao | Sow farm | Hubei Xinhao | Sow farm | Guigang Xinliu | Sow farm |
| Lingxin Xinliu | Fattening farm | Rongchang Pig Farm | Sow farm | Laixi Xinliu | Sow farm |
| Shanxian Xinhao | Sow farm | Dongying Xinhao | Sow farm | Leshan Nongmu | Sow farm |
| Shanxian Xinhao | Sow farm | Dingzhou Xinhao | Sow farm | Shanxian Xinhao | Sow farm |
| Zhoucheng Xinliu | Sow farm | Zhu Cheng Xinliu | Sow farm | Zhaoqing Xinhao | Sow farm |
| Longzhou Xinhao | Sow farm | Dongying Xinhao | Fattening farm | Nantong Xinha | Sow farm |
| Dongying Xinhao | Sow farm | Shanxian Xinhao | Sow farm | Laizhou Xinha | Sow farm |
| Dongying Xinhao | Sow farm | Xiajiang Xinliu | Sow farm | Dongying Xinhao | Sow farm |
| Gaomi Xinliu | Sow farm | Shanxian Xinhao | Sow farm | Lingxin Xinliu | Sow farm |
| Junan Breeding Pigs | Sow farm | Yantai Xinhao | Sow farm | Jian Xinchi | Fattening farm |
| Raoyang Xinhao | Sow farm | Dongying Xinhao | Sow farm | Dingzhou Xinhao | Sow farm |
| Linyi Xinhao | Sow farm | Gansu Xinhao | Sow farm | Wannian Xinha | Sow farm |
| Linyi Xinhao | Sow farm | Yantai Xinhao | Sow farm | Huanghua Xinhao | Sow farm |
| Laixi Xinliu | Fattening farm | Laixi Xinliu | Sow farm | Anyue Xinxinxin | Sow farm |
| Anyue Xinxin | Fattening farm | Shanxian Xinhao | Sow farm | Wuming Xinliu | Sow farm |
| Laixi Xinliu | Sow farm | Dongying Xinhao | Fattening farm | Gaomi Xinliu | Sow farm |
| Qingfeng Xinliu | Fattening farm | Dongying Xinhao | Sow farm | Fucheng Xinhao | Sow farm |
| Hainan State Farms | Sow farm | Lezhi Xinhai | Fattening farm | Guangan Xinhao | Sow farm |
| Yingcheng Xinhao | Sow farm | Liaoning Xintao Wang | Sow farm | Pengshui Xinliu | Sow farm |
| Gansu Xinhao | Sow farm | Hubei Xinhao | Sow farm | Weinan Xinliu | Sow farm |
| Dongying Xinhao | Sow farm | Yantai Xinhao | Sow farm | Guangan Xinhao | Sow farm |
| Nangong Xinhao | Sow farm | Laixi Xinliu | Sow farm | Yingcheng Xinhao | Sow farm |
| Dongying Xinhao | Sow farm | Zhaoqing Xinhao | Sow farm | Ruyuan Xinhao | Sow farm |
| Yingcheng Xinhao | Sow farm | Jingxian Xinhao | Sow farm | Jian Xinchi | Sow farm |
| Tongcheng Xinliu | Sow farm | Raoyang Xinhao | Sow farm | Qingfeng Xinliu | Sow farm |
| Longzhou Xinhao | Sow farm | Gansu Xinhao | Sow farm | Liaoning Xintao Wang | Sow farm |
| Binzhou Xinhai | Sow farm | Gansu Xinhao | Sow farm | Longzhou Xinhao | Sow farm |
| Longzhou Xinhao | Sow farm | Laizhou Xinhai | Fattening farm | Gaomi Xinliu | Sow farm |
| Dongying Xinhao | Sow farm | Dongying Xinhao | Sow farm | Dongying Xinhao | Sow farm |
| Laixi Xinliu | Sow farm | Shuozhou Xinhao | Sow farm | Henan Nongmu | Sow farm |
| Tongliao Xinhao | Sow farm | Nanning Xinhao | Sow farm | Liaoning Xintao Wang | Sow farm |
| Lingbao Xinliu | Sow farm | Longzhou Xinhao | Sow farm | Guangan Xinhao | Sow farm |
| Anyang Xinliu | Sow farm | Dongying Xinhao | Sow farm | Xiajin Nongmu | Sow farm |
| Nanchong Xinhao | Sow farm | Xuanwei Xinliu | Sow farm | Laixi Xinliu | Sow farm |
| Yinbao Breeding | Sow farm | Ruyuan Xinhao | Fattening farm | Dongying Xinhao | Sow farm |
| Gaomi Xinliu | Sow farm | Binzhou Xinhai | Sow farm | Rongchang Pig Farm | Sow farm |
| Dongying Xinhao | Sow farm | Wuhe Xinhai | Fattening farm | Yantai Xinhao | Sow farm |
| Anyang Xinliu | Sow farm | Zhu Cheng Xinliu | Sow farm | Luding Xinyue | Sow farm |
| Zhangjiakou Xinwang | Sow farm | Longzhou Xinhao | Sow farm | Zhangwu Xinwang | Sow farm |
| Hubei Xinhao | Fattening farm | Longzhou Xinhao | Sow farm | Wuhe Xinha | Sow farm |
| Liaoning Xintao Wang | Sow farm | Fucheng Xinhao | Sow farm | Xiajin Nongmu | Sow farm |
| Shuozhou Xinhao | Sow farm | Gaomi Xinliu | Sow farm | Fucheng Xinhao | Sow farm |
| Binzhou Xinhai | Sow farm | Heishan Xinliu | Sow farm | Zhucheng Xinliu | Sow farm |
| Qingyuan Xinhao | Fattening farm | Lingxian Xinliu | Fattening farm | Shanxian Xinhao | Sow farm |
| Zhangjiakou Xinwang | Sow farm | Caoxian Xinhao | Sow farm | Longzhou Xinhao | Sow farm |
| Leshan Agriculture and Animal Husbandry | Sow farm | Laiyang Xinmu | Sow farm | Shanxian Xinhao | Sow farm |
| Jiaxiang Xinliu | Sow farm | Longzhou Xinhao | Sow farm | Nanning Xinhao | Sow farm |
| Weinan Xinliu | Sow farm | Tongliao Xinhao | Sow farm | Linyi Zhongzhu | Sow farm |
| Chenzhou Xinhao | Fattening farm | Lingbao Xinliu | Sow farm | Yantai Xinhao | Sow farm |
| Liuzhou Xinhao | Fattening farm | Laixi Xinliu | Sow farm | Raoyang Xinhao | Sow farm |
| Xingren Xinliu | Sow farm | Yinbao Breeding | Sow farm | Leting Xinha | Sow farm |
| Gaotang Xinhao | Sow farm | Zhangjiakou Xinwang | Sow farm | Nanchong Xinhao | Sow farm |
| Liaoning Xintao Wang | Sow farm | Dongying Xinhao | Sow farm | Liaoning Xintao Wang | Sow farm |
| Liaoning Xinwang | Sow farm | Shibing Xinliu | Sow farm | Yingcheng Xinhao | Sow farm |
| Kangping Xinwang | Sow farm | Binzhou Xinhai | Sow farm | Sichuan Xinhao | Sow farm |
| Santai Agriculture and Animal Husbandry | Sow farm | Xinmin Xinwang | Sow farm | Shanxian Xinhao | Sow farm |
| Hubei Xinhao | Sow farm | Xinmin Xinwang | Sow farm | Gansu Xinhao | Sow farm |
| Yan'an Benyuan | Sow farm | Laixi Xinliu | Sow farm | Yantai Xinhao | Sow farm |
| Guizhou Xinliu | Sow farm | Xiangyang Xinhao | Sow farm | Lingbao Xinliu | Sow farm |
| Yancheng Zhongtai | Sow farm | Tongliao Xinhao | Sow farm | Guangan Xinhao | Sow farm |
| Shibing Xinliu | Sow farm | Ruyuan Xinhao | Fattening farm | Laixi Xinliu | Sow farm |
| Zhangjiakou Xinwang | Sow farm | Longzhou Xinhao | Sow farm | Longzhou Xinhao | Sow farm |
| Laizhou Xinhai | Fattening farm | Fucheng Xinhao | Sow farm | Dongying Xinhao | Sow farm |
| Zhaoqing Xinhao | Fattening farm | Liaoning Xintao Wang | Sow farm | Tongliao Xinhao | Sow farm |
| Jian Xinchi | Sow farm | Leshan Agricultural and Livestock | Sow farm | Xinji Xinliu | Sow farm |
| Kangping Xinwang | Sow farm | Jiaxiang Xinliu | Sow farm | Laixi Xinliu | Sow farm |
| Guanling Xinhai | Fattening farm | Meishan Xinhai | Fattening farm | Gansu Xinhao | Sow farm |
| Qingfeng Xinliu | Sow farm | Gansu Xinhao | Sow farm | Ruyuan Xinhao | Sow farm |
| Gansu Xinhao | Sow farm | Wuhe Xinhai | Fattening farm | Dongying Xinhao | Sow farm |
| Bijie Xinliu | Sow farm | Weinan Xinliu | Sow farm | Dongying Xinhao | Sow farm |
| Liaoning Xintao Wang | Sow farm | Qingyuan Xinhao | Fattening farm | Dongying Xinhao | Sow farm |
| Qinghua Agriculture and Animal Husbandry | Sow farm | Gaomi Xinliu | Sow farm | Tangshan Xinhao | Sow farm |
| Henan Agriculture and Animal Husbandry | Sow farm | Gaotang Xinhao | Sow farm | Heishan Xinliu | Sow farm |
| Liaoning Food | Sow farm | Yan'an Benyuan | Sow farm | Xiangshui Xincheng | Sow farm |
| Liangshan Xinliu | Sow farm | Gansu Xinliu | Fattening farm | Zhucheng Xinliu | Sow farm |
| Xinmin Xinwang | Sow farm | Guizhou Xinliu | Sow farm | Shuozhou Xinhao | Sow farm |
| Xinmin Xinwang | Sow farm | Luocheng Xinhao | Fattening farm | Longzhou Xinhao | Sow farm |
| Hezhou Xinhao | Sow farm | Guanling Xinhai | Sow farm | Gansu Xinhao | Sow farm |
| Gaomi Xinliu | Sow farm | Santai Agricultural and Livestock | Sow farm | Laixi Xinliu | Sow farm |
| Liaoning Food | Sow farm | Hubei Xinhao | Sow farm | Yinbao Breeding | Sow farm |
| Guanling Xinhai | Sow farm | Shuozhou Xinhao | Sow farm | Binzhou Xinha | Sow farm |
| Caoxian Xinhao | Sow farm | Gaomi Xinliu | Sow farm | Longzhou Xinhao | Sow farm |
| Fucheng Xinhao | Sow farm | Kangping Xinwang | Sow farm | Xinmin Xiwang | Sow farm |
| Xinmin Xinwang | Sow farm | Nanning Xinhao | Sow farm | Longzhou Xinhao | Sow farm |
| Dingzhou Xinhao | Sow farm | Zhangjiakou Xinwang | Sow farm | Hebei Xinhao | Sow farm |
| Zhoucheng Xinliu | Sow farm | Heishan Xinliu | Sow farm | Xinmin Xiwang | Sow farm |
| Laiyang Xinhao | Sow farm | Liangshan Xinliu | Sow farm | Longzhou Xinhao | Sow farm |
| Gansu Xinhao | Sow farm | Liaoning Xintao Wang | Sow farm | Leting Xinha | Sow farm |
| Gaomi Xinliu | Sow farm | Zhu Cheng Xinliu | Sow farm | Jiaxiang Xinliu | Sow farm |
| Pingfu Agriculture and Animal Husbandry | Sow farm | Tongliao Xinhao | Sow farm | Hubei Xinhao | Sow farm |
| Tongliao Xinhao | Sow farm | Liaoning Xintao Wang | Sow farm | Qingyuan Xinhao | Sow farm |
| Chenzhou Xinhao | Fattening farm | Nanning Xinhao | Sow farm | Xiangyang Xinhao | Sow farm |
| Heishan Xinliu | Sow farm | Fucheng Xinhao | Sow farm | Liaoning Xintao Wang | Sow farm |
| Gansu Xinhao | Sow farm | Laiyang Xinhao | Sow farm | Leshan Nongmu | Sow farm |
| Fucheng Xinhao | Sow farm | Gansu Xinhao | Sow farm | Binzhou Xinha | Sow farm |
| Liaoning Xintao Wang | Sow farm | Gansu Xinhao | Sow farm | Gaotang Xinhao | Sow farm |
| Taian Xinliu | Sow farm | Juye Xinhao | Sow farm | Qingfeng Xinliu | Sow farm |
| Gansu Xinhao | Sow farm | Raoyang Xinhao | Sow farm | Weinan Xinliu | Sow farm |
| Yichun Xinwang | Sow farm | Tongliao Xinhao | Sow farm | Fucheng Xinhao | Sow farm |
| Junan Breeding Pigs | Sow farm | Ruzhou Quansheng | Sow farm | Weihui Xincheng | Sow farm |
| Gansu Xinhao | Sow farm | Pingfu Agricultural and Livestock | Sow farm | Xuanwei Xinliu | Sow farm |
| Tongliao Xinhao | Sow farm | Longhui Xinhai | Fattening farm | Zhangjiakou Xiwang | Sow farm |
| Laixi Xinliu | Sow farm | Donghai Xinhao | Sow farm | Hubei Xinhao | Sow farm |
| Xinmin New Hope | Sow farm | Liaocheng Xinhao | Sow farm | Gaomi Xinliu | Sow farm |
| Liaocheng Xinhao | Sow farm | Liuzhou Xinhao | Fattening farm | Santai Agriculture and Animal Husbandry | Sow farm |
| Heishan Xinliu | Sow farm | Taian Xinliu | Sow farm | Gaomi Xinliu | Sow farm |
| Shibing Xinliu | Sow farm | Gansu Xinhao | Sow farm | Raoyang Xinhao | Sow farm |
| Laixi Xinliu | Sow farm | Hezhou Xinhao | Sow farm | Shuozhou Xinhao | Sow farm |
| Wuhe Xinhai | Sow farm | Qingfeng Xinliu | Sow farm | Zhu Cheng Xinliu | Sow farm |
| Huanghua Xinhao | Sow farm | Qinghua Nongmu | Sow farm | Yan'an Ben Yuan | Sow farm |
| Zhongshan Agriculture and Animal Husbandry | Sow farm | Xinmin Xinwang | Sow farm | Xin Ji Xinliu | Sow farm |
| Tongliao Xinhao | Sow farm | Zhuocheng Xinliu | Sow farm | Tongliao Xinhao | Sow farm |
| Anyang Xinliu | Sow farm | Zhongshan Nongmu | Sow farm | Ru Zhou Quan Sheng | Sow farm |
| Jiangyou Pig Farm | Sow farm | Gansu Xinhao | Sow farm | Kang Ping Xin Wang | Sow farm |
| Zhuocheng Xinliu | Sow farm | Jiangyou Pig Farm | Sow farm | Guizhou Xinliu | Sow farm |
| Shuozhou Xinhao | Sow farm | Henan Nongmu | Sow farm | Gansu Xinhao | Sow farm |
| Donghai Xinhao | Sow farm | Laixi Xinliu | Sow farm | Gaomi Xinliu | Sow farm |
| Liaoning Food | Sow farm | Chenzhou Xinhao | Sow farm | Gansu Xinhao | Sow farm |
| Laixi Xinliu | Sow farm | Tongliao Xinhao | Sow farm | Liaocheng Xinhao | Sow farm |
| Hubei Xinhao | Sow farm | Shuozhou Xinhao | Sow farm | Ru Zhou Quan Sheng | Sow farm |
| Donghai Xinhao | Sow farm | Yingcheng Xinhao | Sow farm | Tai'an Xin Chi | Sow farm |
| Bijie Xinliu | Sow farm | Weinan Xinliu | Sow farm | Liaoning Xin Tao Wang | Sow farm |
| Anyang Xinliu | Sow farm | Yichun Xinwang | Sow farm | Ju Ye Xinhao | Sow farm |
| Heishan Xinliu | Sow farm | Hezhou Xinhao | Sow farm | Lai Yang Xinhao | Sow farm |
| Anyue Xing Xinxin | Sow farm | Liaoning Xintao Wang | Sow farm | Gansu Xinhao | Sow farm |
| Nanning Xinliu | Sow farm | Gansu Xinhao | Sow farm | Xin Ji Xinliu | Sow farm |
| Tangshan Xinhao | Sow farm | Zhangjiakou Xinwang | Sow farm | Liangshan Xinliu | Sow farm |
| Laizhou Xinhai | Sow farm | Yichun Xinwang | Fattening farm | Yingtan Xinliu | Sow farm |
| Juye Xinhao | Sow farm | Xiangshui Xinchi | Fattening farm | Xin Ji Xinliu | Sow farm |
| Liaoning Xinwang | Sow farm | Wuhe Xinhai | Sow farm | Changyi Xinhao | Sow farm |
| Kangping Xinwang | Sow farm | Laixi Xinliu | Sow farm | Liaoning Xin Tao Wang | Sow farm |
| Gansu Xinhao | Sow farm | Laizhou Xinhai | Sow farm | Xianyang Yong Xiang | Sow farm |
| Guangyuan Xinhao | Sow farm | Nanning Xinliu | Sow farm | Xiajin Agriculture and Animal Husbandry | Sow farm |
| Xiajin Agriculture and Animal Husbandry | Sow farm | Shenze Xinhao | Sow farm | Gansu Xinhao | Sow farm |
| Laixi Xinliu | Sow farm | Juye Xinhao | Sow farm | Yichun Xin Wang | Sow farm |
| Liaoning New Taowang | Sow farm | Liaoning Xinwang | Sow farm | Zhenjiang Xin Hai | Sow farm |
| Shuozhou Xinhao | Sow farm | Zhejiang Xinhai | Sow farm | Qingfeng Xinliu | Sow farm |
| Yingtan Xinliu | Fattening farm | Laixi Xinliu | Sow farm | Ruyuan Xinhao | Sow farm |
| Dingzhou Xinhao | Sow farm | Anyue Xinxin | Sow farm | Zhaoqing Xinhao | Sow farm |
| Hezhou Xinhao | Sow farm | Shuozhou Xinhao | Sow farm | Tongliao Xinhao | Sow farm |
| Raoyang Xinhao | Sow farm | Zhejiang Xinhai | Sow farm | Laizhou Xin Hai | Sow farm |
| Weinan Xinliu | Sow farm | Tangshan Xinhao | Sow farm | Pingfu Agriculture and Animal Husbandry | Sow farm |
| Zhejiang Xinhai | Sow farm | Ruzhou Quansheng | Sow farm | Ru Zhou Quan Sheng | Sow farm |
| Nanning Xinhao | Sow farm | Hubei Xinhao | Sow farm | Heishan Xinliu | Sow farm |
| Wuhe Xinhai | Fattening farm | Lingbao Xinliu | Sow farm | Zhaoqing Xinhao | Sow farm |
| Taian Xinliu | Sow farm | Huanghua Xinhao | Sow farm | Taian Xinliu | Sow farm |
| Xiangshui Xinchi | Fattening farm | Changyi Xinhao | Sow farm | Zhangjiakou Xin Wang | Sow farm |
| Ningming Xinhao | Sow farm | Jingxian Xinhao | Sow farm | Zhu Cheng Xinliu | Sow farm |
| Yichun Xinwang | Sow farm | Xinji Xinliu | Sow farm | Shenze Xinhao | Sow farm |
| Tangshan Xinhao | Sow farm | Liaoning Xintao Wang | Sow farm | Luo Cheng Xinhao | Sow farm |
| Yingcheng Xinhao | Sow farm | Taian Xinliu | Sow farm | Dezhou Xinhao | Sow farm |
| Ningming Xinhao | Sow farm | Liaoning Xinwang | Sow farm | Qinghua Agriculture and Animal Husbandry | Sow farm |
| Shuozhou Xinhao | Sow farm | Changyi Xinhao | Sow farm | Xin Ji Xinliu | Sow farm |
| Juye Xinhao | Sow farm | Ningbo Xinhai | Sow farm | Shuozhou Xinhao | Sow farm |
| Ruzhou Quansheng | Sow farm | Juye Xinhao | Sow farm | Liuzhou Xinhao | Sow farm |
| Ruzhou Quansheng | Sow farm | Heishan Xinliu | Sow farm | Guangyuan Xinhao | Sow farm |
| Raoyang Xinhao | Sow farm | Xinji Xinliu | Sow farm | Ji'an Xin Chi | Sow farm |
| Tangshan Xinhao | Sow farm | Bijie Xinliu | Sow farm | Laixi Xinliu | Fattening farm |
| Tongliao Xinhao | Sow farm | Shenze Xinhao | Sow farm | Tangshan Xinhao | Sow farm |
| Heishan Xinliu | Sow farm | Zhaoqing Xinhao | Fattening farm | Ruyuan Xinhao | Sow farm |
| Tongliao Xinhao | Sow farm | Xinji Xinliu | Sow farm | Zhongshan Agriculture and Animal Husbandry | Sow farm |
| Shibing Xinliu | Sow farm | Gansu Xinhao | Sow farm | Shuozhou Xinhao | Sow farm |
| Taian Xinliu | Sow farm | Juye Xinhao | Sow farm | Laixi Xinliu | Sow farm |
| Shuozhou Xinhao | Fattening farm | Kangping Xinwang | Sow farm | Jiangyou Pig Farm | Sow farm |
| Dingzhou Xinhao | Sow farm | Tongliao Xinhao | Sow farm | Dezhou Xinhao | Sow farm |
| Tangshan Xinhao | Sow farm | Dingzhou Xinhao | Sow farm | Changyi Xinhao | Sow farm |
| Xinji Xinliu | Sow farm | Chenzhou Xinhao | Fattening farm | Tongliao Xinhao | Sow farm |
| Liaoning Food | Sow farm | Changyi Xinhao | Sow farm | Shenze Xinhao | Sow farm |
| Ningming Xinhao | Sow farm | Tangshan Xinhao | Sow farm | Chenzhou Xinhao | Sow farm |
| Anhui Xinliu | Sow farm | Yichun Xinwang | Sow farm | Juye Xinhao | Sow farm |
| Guangyuan Xinhao | Sow farm | Bijie Xinliu | Sow farm | Shenze Xinhao | Sow farm |
| Hubei Xinhao | Sow farm | Weihui Xinchi | Sow farm | Tongliao Xinhao | Sow farm |
| Zhangjiakou Xinwang | Sow farm | Shibing Xinliu | Sow farm | Xiajin Agriculture and Animal Husbandry | Sow farm |
| Ningming Xinhao | Sow farm | Santai Nongmu | Sow farm | Heishan Xinliu | Sow farm |
| Shenze Xinhao | Sow farm | Qingfeng Xinliu | Sow farm | Zhaoqing Xinhao | Sow farm |
| Taian Xinliu | Sow farm | Nanning Xinhao | Sow farm | Xin Ji Xinliu | Sow farm |
| Gansu Xinhao | Sow farm | Shenze Xinhao | Sow farm | Hubei Xinhao | Sow farm |
| Jingxian Xinhao | Sow farm | Yingtan Xinliu | Fattening farm | Tongliao Xinhao | Sow farm |
| Yingcheng Xinhao | Sow farm | Huanghua Xinhao | Sow farm | Juye Xinhao | Sow farm |
| Nanning Xinhao | Sow farm | Ruzhou Quansheng | Sow farm | Anyue Xing Xin Xin | Sow farm |
| Hubei Xinhao | Sow farm | Tangshan Xinhao | Sow farm | Huanghua Xinhao | Sow farm |
| Santai Agriculture and Animal Husbandry | Sow farm | Yingcheng Xinhao | Sow farm | Liaoning Xin Tao Wang | Sow farm |
| Xinji Xinliu | Sow farm | Taian Xinliu | Sow farm | Gansu Xinhao | Sow farm |
| Hezhou Xinhao | Sow farm | Tangshan Xinhao | Sow farm | Donghai Xinhao | Sow farm |
| Leshan Agriculture and Animal Husbandry | Sow farm | Kangping Xinwang | Sow farm | Liaoning Xin Wang | Sow farm |
| Ningming Xinhao | Sow farm | Hubei Xinhao | Sow farm | Juye Xinhao | Sow farm |
| Ningming Xinhao | Sow farm | Tangshan Xinhao | Sow farm | Laixi Xinliu | Sow farm |
| Gansu Xinhao | Sow farm | Tongliao Xinhao | Sow farm | Changyi Xinhao | Sow farm |
| Longzhou Xinhao | Fattening farm | Xinji Xinliu | Sow farm | Guanling Xin Hai | Sow farm |
| Qingfeng Xinliu | Sow farm | Dezhou Xinhao | Sow farm | Shibing Xinliu | Sow farm |
| Juye Xinhao | Sow farm | Dezhou Xinhao | Sow farm | Hubei Xinhao | Sow farm |
| Donghai Xinhao | Sow farm | Heishan Xinliu | Sow farm | Nanning Xinhao | Sow farm |
| Tangshan Xinhao | Sow farm | Tongliao Xinhao | Sow farm | Xiajin Agriculture and Animal Husbandry | Sow farm |
| Tangshan Xinhao | Sow farm | Fuxin Xinwang | Sow farm | Gansu Xinhao | Sow farm |
| Weihui Xinchi | Sow farm | Tongliao Xinhao | Sow farm | Lingbao Xinliu | Sow farm |
| Gansu Xinhao | Sow farm | Xiajin Nongmu | Sow farm | Ru Zhou Quan Sheng | Sow farm |
| Wuhe Xinhai | Sow farm | Heishan Xinliu | Sow farm | Zhangjiakou Xin Wang | Sow farm |
| Jingxian Xinhao | Sow farm | Guangan Xinhao | Fattening farm | Dong'e Xinliu | Sow farm |
| Tongliao Xinhao | Sow farm | Leshan Nongmu | Sow farm | Xin Ji Xinliu | Sow farm |
| Heishan Xinliu | Sow farm | Dong'e Xinliu | Sow farm | Tongliao Xinhao | Sow farm |
| Shuozhou Xinhao | Sow farm | Yingcheng Xinhao | Sow farm | Liaoning Xin Tao Wang | Sow farm |
| Anyang Xinliu | Sow farm | Tangshan Xinhao | Sow farm | Tongliao Xinhao | Sow farm |
| Linghe Xinliu | Sow farm | Xinji Xinliu | Sow farm | Nanning Xinliu | Sow farm |
| Hainan Xinliu | Sow farm | Hezhou Xinhao | Sow farm | Xiajin Agriculture and Animal Husbandry | Sow farm |
| Qingfeng Xinliu | Sow farm | Taian Xinliu | Sow farm | Huanghua Xinhao | Sow farm |
| Laixi Xinliu | Sow farm | Xinji Xinliu | Sow farm | Yingcheng Xinhao | Sow farm |
| Gansu Xinhao | Sow farm | Anhui Xinliu | Sow farm | Longzhou Xinhao | Sow farm |
| Hezhou Xinhao | Fattening farm | Ruzhou Quansheng | Sow farm | Nanning Xinhao | Sow farm |
| Liaoning Xinwang | Sow farm | Xiajin Nongmu | Sow farm | Shenze Xinhao | Sow farm |
| Juye Xinhao | Sow farm | Fucheng Xinhao | Sow farm | Dezhou Xinhao | Sow farm |
| Tangshan Xinhao | Sow farm | Dezhou Xinhao | Sow farm | Gansu Xinliu | Sow farm |
| Guangan Xinhao | Fattening farm | Kangping Xinwang | Sow farm | Gansu Xinhao | Sow farm |
| Tangshan Xinhao | Sow farm | Tianxin Xinliu | Fattening farm | Dezhou Xinhao | Sow farm |
| Tangshan Xinhao | Sow farm | Ningming Xinhao | Sow farm | Heishan Xinliu | Sow farm |
| Hezhou Xinhao | Sow farm | Qingfeng Xinliu | Sow farm | Meishan Xin Hai | Sow farm |
| Zhongshan Agriculture and Animal Husbandry | Sow farm | Tongliao Xinhao | Sow farm | Tongliao Xinhao | Sow farm |
| Tangshan Xinhao | Sow farm | Xinji Xinliu | Sow farm | Hezhou Xinhao | Sow farm |
| Hengnan Muyun | Sow farm | Tangshan Xinhao | Sow farm | Xiajin Agriculture and Animal Husbandry | Sow farm |
| Tangshan Xinhao | Sow farm | Hebei Xinhao | Sow farm | Hebei Xinhao | Sow farm |
| Tongliao Xinhao | Sow farm | Guanling Xinhai | Sow farm | Tangshan Xinhao | Sow farm |
| Zhuocheng Xinliu | Sow farm | Tangshan Xinhao | Sow farm | Dezhou Xinhao | Sow farm |
| Ningming Xinhao | Sow farm | Huanghua Xinhao | Fattening farm | Caoxian Xinhao | Sow farm |
| Tongliao Xinhao | Sow farm | Hubei Xinhao | Sow farm | Hebei Xinhao | Sow farm |
| Xinji Xinliu | Sow farm | Laixi Xinliu | Sow farm | Kangping Xin Wang | Sow farm |
| Tongliao Xinhao | Sow farm | Liaoning Xinwang | Sow farm | Tangshan Xinhao | Sow farm |
| Xinji Xinliu | Sow farm | Dezhou Xinhao | Sow farm | Guigang Xinliu | Sow farm |
| Wuhe Xinhai | Sow farm | Tangshan Xinhao | Sow farm | Taian Xinliu | Sow farm |
| Changyi Xinhao | Sow farm | Zhangjiakou Xinwang | Sow farm | Yingcheng Xinhao | Sow farm |
| Tongliao Xinhao | Sow farm | Taian Xinchi | Sow farm | Shuozhou Xinhao | Sow farm |
| Changyi Xinhao | Sow farm | Tangshan Xinhao | Sow farm | Santai Agriculture and Animal Husbandry | Sow farm |
| Ruzhou Quansheng | Sow farm | Wuhe Xinhai | Sow farm | Liaocheng Xinhao | Sow farm |
| Tangshan Xinhao | Sow farm | Weinan Xinliu | Sow farm | Xin Ji Xinliu | Sow farm |
| Baiyin Agriculture and Animal Husbandry | Sow farm | Tangshan Xinhao | Sow farm | Tangshan Xinhao | Sow farm |
| Baiyin Agriculture and Animal Husbandry | Sow farm | Tongliao Xinhao | Sow farm | Ru Zhou Quan Sheng | Sow farm |
| Huanghua Xinhao | Sow farm | Heishan Xinliu | Sow farm | Tangshan Xinhao | Sow farm |
| Kangping Xinwang | Sow farm | Hezhou Xinhao | Sow farm | Weinan Xinliu | Sow farm |
| Xinji Xinliu | Sow farm | Zhaoqing Xinhao | Sow farm | Zhejiang Xin Hai | Sow farm |
| Hubei Xinhao | Sow farm | Zhuocheng Xinliu | Sow farm | Taian Xinliu | Sow farm |
| Xinji Xinliu | Sow farm | Nanning Xinhao | Sow farm | Xin Ji Xinliu | Sow farm |
| Liaoning Xinwang | Sow farm | Shuozhou Xinhao | Sow farm | Taian Xinliu | Sow farm |
| Laixi Xinliu | Sow farm | Yingtan Xinliu | Sow farm | Wuhe Xin Hai | Sow farm |
| Hainan Xinliu | Sow farm | Dezhou Xinhao | Sow farm | Kangping Xin Wang | Sow farm |
| Hubei Xinhao | Sow farm | Xingren Xinliu | Sow farm | Leshan Agriculture and Animal Husbandry | Sow farm |
| Taian Xinchi | Sow farm | Qingfeng Xinliu | Sow farm | Nanning Xinhao | Sow farm |
| Tianxin Xinliu | Fattening farm | Linyi Zhongzhu | Sow farm | Ru Zhou Quan Sheng | Sow farm |
| Nanchong Xinhao | Sow farm | Taian Xinliu | Sow farm | Tongliao Xinhao | Sow farm |
| Taian Xinliu | Sow farm | Lingao Xinliu | Sow farm | Heishan Xinliu | Sow farm |
| Linghe Xinliu | Sow farm | Gansu Xinhao | Sow farm | Qingfeng Xinliu | Sow farm |
| Tongliao Xinhao | Sow farm | Wuhe Xinhai | Sow farm | Gaotang Xinhao | Sow farm |
| Wuqi Xinliu | Sow farm | Ningming Xinhao | Sow farm | Tangshan Xinhao | Sow farm |
| Xinji Xinliu | Sow farm | Tongliao Xinhao | Sow farm | Tongliao Xinhao | Sow farm |
| Heishan Xinliu | Sow farm | Baiyin Nongmu | Sow farm | Heishan Xinliu | Sow farm |
| Yucheng Xinliu | Sow farm | Xiangzhou Xinhao | Sow farm | Liaocheng Xinhao | Sow farm |
| Changyi Xinhao | Sow farm | Shuozhou Xinhao | Sow farm | Liaocheng Xinhao | Sow farm |
| Baiyin Agriculture and Animal Husbandry | Sow farm | Anyue Xinxin | Fattening farm | Laixi Xinliu | Sow farm |
| Yucheng Xinliu | Sow farm | Hezhou Xinhao | Sow farm | Anhui Xinliu | Sow farm |
| Gansu Xinhao | Sow farm | Raoyang Xinhao | Sow farm | Yucheng Xinliu | Sow farm |
| Hezhou Xinhao | Sow farm | Tangshan Xinhao | Sow farm | Guangan Xinhao | Sow farm |
| Heishan Xinliu | Sow farm | Hubei Xinhao | Sow farm | Tongliao Xinhao | Sow farm |
| Xinji Xinliu | Sow farm | Xiangzhou Xinhao | Sow farm | Yichun New Hope | Sow farm |
| Wuhe Xinhai | Fattening farm | Xiajin Agriculture and Animal Husbandry | Sow farm | Bijie Xinliu | Sow farm |
| Kangping New Hope | Sow farm | Hainan Xinliu | Sow farm | Chenzhou Xinhao | Sow farm |
| Guangdong Xinhao | Sow farm | Liaoning New Hope | Sow farm | Laiyang Xinmu | Sow farm |
| Baiyin Agriculture and Animal Husbandry | Sow farm | Kangping New Hope | Sow farm | Tangshan Xinhao | Sow farm |
| Heyang Xinliu | Sow farm | Yucheng Xinliu | Sow farm | Zhangjiakou New Hope | Sow farm |
| Baiyin Agriculture and Animal Husbandry | Sow farm | Ningming Xinhao | Sow farm | Liaoning New Hope | Sow farm |
| Liaoning New Hope | Sow farm | Liaocheng Xinhao | Sow farm | Heishan Xinliu | Sow farm |
| Yingcheng Xinhao | Sow farm | Heishan Xinliu | Sow farm | Tangshan Xinhao | Sow farm |
| Danxian Xinliu | Sow farm | Xinji Xinliu | Sow farm | Tangshan Xinhao | Sow farm |
| Tongliao Xinhao | Sow farm | Caoxian Xinhao | Sow farm | Hubei Xinhao | Sow farm |
| Baiyin Agriculture and Animal Husbandry | Sow farm | Heishan Xinliu | Sow farm | Tangshan Xinhao | Sow farm |
| Shibing Xinliu | Sow farm | Hubei Xinhao | Sow farm | Zhuocheng Xinliu | Sow farm |
| Liaoning New Hope | Sow farm | Hebei Xinhao | Sow farm | Shenze Xinhao | Sow farm |
| Tongliao Xinhao | Sow farm | Yingcheng Xinhao | Sow farm | Hezhou Xinhao | Sow farm |
| Jiaxiang Xinliu | Sow farm | Nanchong Xinhao | Sow farm | Caoxian Xinhao | Sow farm |
| Shenze Xinhao | Sow farm | Baiyin Agriculture and Animal Husbandry | Sow farm | Nanning Xinliu | Sow farm |
| Hengnan Animal Husbandry | Sow farm | Weinan Xinliu | Sow farm | Liaoning New Hope | Sow farm |
| Tongliao Xinhao | Sow farm | Heyang Xinliu | Sow farm | Tianxin Xinliu | Sow farm |
| Laiyang Xinmu | Sow farm | Ningming Xinhao | Sow farm | Taian Xinliu | Sow farm |
| Heyang Xinliu | Sow farm | Hengnan Pastoral | Sow farm | Hubei Xinhao | Sow farm |
| Tangshan Xinhao | Sow farm | Yucheng Xinliu | Sow farm | Zhejiang Xinhai | Sow farm |
| Laixi Xinliu | Sow farm | Ningming Xinhao | Sow farm | Liaoning New Hope | Sow farm |
| Tongliao Xinhao | Sow farm | Gansu Xinhao | Sow farm | Nanning Xinhao | Sow farm |
| Laixi Xinliu | Sow farm | Gansu Xinhao | Sow farm | Xiajiang Xinliu | Sow farm |
| Tongliao Xinhao | Sow farm | Shenze Xinhao | Sow farm | Hubei Xinhao | Sow farm |
| Caoxian Xinhao | Sow farm | Tongliao Xinhao | Sow farm | Bijie Xinliu | Sow farm |
| Wuhe Xinhai | Sow farm | Jiaxiang Xinliu | Sow farm | Baiyin Agriculture and Animal Husbandry | Sow farm |
| Shenze Xinhao | Sow farm | Lingao Xinliu | Sow farm | Tangshan Xinhao | Sow farm |
| Yijun Xinliu | Sow farm | Xiangzhou Xinhao | Sow farm | Hezhou Xinhao | Sow farm |
| Liaoning New Hope | Sow farm | Tangshan Xinhao | Sow farm | Shibing Xinliu | Sow farm |
| Tongliao Xinhao | Sow farm | Heyang Xinliu | Sow farm | Wuhe Xinhai | Sow farm |
| Dezhou Xinhao | Sow farm | Tongliao Xinhao | Sow farm | Hebei Xinhao | Sow farm |
| Tongliao Xinhao | Sow farm | Shuozhou Xinhao | Sow farm | Luocheng Xinhao | Sow farm |
| Liaoning New Hope | Sow farm | Baiyin Agriculture and Animal Husbandry | Sow farm | Hubei Xinhao | Sow farm |
| Tongliao Xinhao | Sow farm | Liaoning New Hope | Sow farm | Shuozhou Xinhao | Sow farm |
| Shenze Xinhao | Sow farm | Kangping New Hope | Sow farm | Weinan Xinliu | Sow farm |
| Guangdong Xinhao | Sow farm | Hezhou Xinhao | Sow farm | Xiangzhou Xinhao | Sow farm |
| Liaoning New Hope | Sow farm | Tongliao Xinhao | Sow farm | Liaoning New Hope | Sow farm |
| Santai Agriculture and Animal Husbandry | Sow farm | Baiyin Agriculture and Animal Husbandry | Sow farm | Liaoning New Hope | Sow farm |
| Xiangzhou Xinhao | Sow farm | Baiyin Agriculture and Animal Husbandry | Sow farm | Dong'e Xinliu | Sow farm |
| Liaoning New Hope | Sow farm | Tongliao Xinhao | Sow farm | Xiangzhou Xinhao | Sow farm |
| Yijun Xinliu | Sow farm | Gansu Xinhao | Sow farm | Kangping New Hope | Sow farm |
| Liaoyang Xinmu | Sow farm | Ningming Xinhao | Sow farm | Tangshan Xinhao | Sow farm |
| Liaocheng Xinhao | Sow farm | Dong'e Xinliu | Sow farm | Heyang Xinliu | Sow farm |
| Laibin Xinhao | Sow farm | Raoyang Xinhao | Sow farm | Yucheng Xinliu | Sow farm |
| Tongliao Xinhao | Sow farm | Guangdong Xinhao | Sow farm | Baiyin Agriculture and Animal Husbandry | Sow farm |
| Yijun Xinliu | Sow farm | Hebei Xinhao | Sow farm | Jiaxiang Xinliu | Sow farm |
| Kangping New Hope | Sow farm | Baiyin Agriculture and Animal Husbandry | Sow farm | Yichun New Hope | Sow farm |
| Guangdong Xinhao | Sow farm | Wuqi Xinliu | Sow farm | Wuhe Xinhai | Sow farm |
| Qinghua Agriculture and Animal Husbandry | Sow farm | Shenze Xinhao | Sow farm | Tongliao Xinhao | Sow farm |
| Laiyang Xinmu | Sow farm | Yingcheng Xinhao | Sow farm | Ningming Xinhao | Sow farm |
| Yanting Xinhao | Sow farm | Hengnan Pastoral | Sow farm | Shuozhou Xinhao | Sow farm |
| Liaoning New Hope | Sow farm | Tongliao Xinhao | Sow farm | Caoxian Xinhao | Sow farm |
| Dezhou Xinhao | Sow farm | Gaotang Xinhao | Sow farm | Gansu Xinhao | Sow farm |
| Raoyang Xinhao | Sow farm | Shuozhou Xinhao | Sow farm | Shenze Xinhao | Sow farm |
| Nanchong Xinhao | Fattening farm | Xiajin Agriculture and Animal Husbandry | Sow farm | Dezhou Xinhao | Sow farm |
| Laibin Xinhao | Sow farm | Liaoning New Hope | Sow farm | Hezhou Xinhao | Sow farm |
| Liaocheng Xinhao | Sow farm | Liaoning New Hope | Sow farm | Lingxin Xinliu | Sow farm |
| Shibing Xinliu | Sow farm | Xiajin Agriculture and Animal Husbandry | Sow farm | Nanchong Xinhao | Sow farm |
| Weinan Xinliu | Sow farm | Laixi Xinliu | Sow farm | Xiangzhou Xinhao | Sow farm |
| Lingbao Xinliu | Sow farm | Shenze Xinhao | Sow farm | Gansu Xinhao | Sow farm |
| Xiajin Agriculture and Animal Husbandry | Sow farm | Wuhe Xinhai | Sow farm | Hezhou Xinhao | Sow farm |
| Shenze Xinhao | Sow farm | Danxian Xinliu | Sow farm | Kangping New Hope | Sow farm |
| Guangdong Xinhao | Sow farm | Tongliao Xinhao | Sow farm | Gansu Xinhao | Sow farm |
| Liaoning New Hope | Sow farm | Shibing Xinliu | Sow farm | Hezhou Xinhao | Sow farm |
| Xiajin Agriculture and Animal Husbandry | Sow farm | Guangyuan Xinhao | Sow farm | Kangping New Hope | Sow farm |
| Liaoning New Hope | Sow farm | Liaoning New Hope | Sow farm | Hengnan Pastoral Farming | Sow farm |
| Dezhou Xinhao | Sow farm | Guangdong Xinhao | Sow farm | Xiajin Agriculture and Animal Husbandry | Sow farm |
| Shenze Xinhao | Sow farm | Ningming Xinhao | Sow farm | Baiyin Agriculture and Animal Husbandry | Sow farm |
| Kangping New Hope | Sow farm | Xiajin Agriculture and Animal Husbandry | Sow farm | Tongliao Xinhao | Sow farm |
| Xiajin Agriculture and Animal Husbandry | Sow farm | Tongliao Xinhao | Sow farm | Baiyin Agriculture and Animal Husbandry | Sow farm |
| Laibin Xinhao | Sow farm | Lingbao Xinliu | Sow farm | Hengnan Pastoral Farming | Sow farm |
| Xiajin Agriculture and Animal Husbandry | Sow farm | Liaocheng Xinhao | Sow farm | Weinan Xinliu | Sow farm |
| Lingbao Xinliu | Sow farm | Liaoning New Hope | Sow farm | Gansu Xinhao | Sow farm |
| Xiajin Agriculture and Animal Husbandry | Sow farm | Guangdong Xinhao | Sow farm | Heyang Xinliu | Sow farm |
| Tongliao Xinhao | Sow farm | Hainan Xinliu | Sow farm | Baiyin Agriculture and Animal Husbandry | Sow farm |
| Weinan Xinliu | Sow farm | Kangping New Hope | Sow farm | Yingcheng Xinhao | Sow farm |
| Baiyin Agriculture and Animal Husbandry | Sow farm | Tongliao Xinhao | Sow farm | Tongliao Xinhao | Sow farm |
| Xiangzhou Xinhao | Sow farm | Shibing Xinliu | Sow farm | Tongliao Xinhao | Sow farm |
| Dezhou Xinhao | Sow farm | Yijun Xinliu | Sow farm | Baiyin Agriculture and Animal Husbandry | Sow farm |
| Qinghua Agriculture and Animal Husbandry | Sow farm | Xiajin Agriculture and Animal Husbandry | Sow farm | Ningming Xinhao | Sow farm |
| Xiajin Agriculture and Animal Husbandry | Sow farm | Dezhou Xinhao | Sow farm | Yingcheng Xinhao | Sow farm |
| Hebei Xinhao | Sow farm | Yijun Xinliu | Sow farm | Nanning Xinhao | Sow farm |
| Huanghua Xinhao | Sow farm | Weinan Xinliu | Sow farm | Liaoning New Hope | Sow farm |
| Xiangzhou Xinhao | Sow farm | Santai Agriculture and Animal Husbandry | Sow farm | Laixi Xinliu | Sow farm |
| Dong'e Xinliu | Sow farm | Laibin Xinhao | Sow farm | Shuozhou Xinhao | Sow farm |
| Yan'an Benyuan | Sow farm | Huanghua Xinhao | Sow farm | Yingcheng Xinhao | Sow farm |
| Kangping New Hope | Sow farm | Xiangzhou Xinhao | Sow farm | Wuhe Xinhai | Fattening farm |
| Fucheng Xinhao | Sow farm | Liaocheng Xinhao | Sow farm | Guangdong Xinhao | Sow farm |
| Jiaxiang Xinliu | Sow farm | Caoxian Xinhao | Sow farm | Wuqi Xinliu | Sow farm |
| Liaocheng Xinhao | Sow farm | Weinan Xinliu | Sow farm | Ningming Xinhao | Sow farm |
| Dong'e Xinliu | Sow farm | Laiyang Xinmu | Sow farm | Guangdong Xinhao | Sow farm |
| Zhangwu New Hope | Sow farm | Guangdong Xinhao | Sow farm | Liaoning New Hope | Sow farm |
| Hebei Xinhao | Sow farm | Liaoning New Hope | Sow farm | Guangdong Xinhao | Sow farm |
| Juye Xinhao | Sow farm | Dong'e Xinliu | Sow farm | Jiaxiang Xinliu | Sow farm |
| Dong'e Xinliu | Sow farm | Liaoning New Hope | Sow farm | Ningming Xinhao | Sow farm |
| Laibin Xinhao | Sow farm | Jiaxiang Xinliu | Sow farm | Huanghua Xinhao | Sow farm |
| Raoyang Xinhao | Sow farm | Shibing Xinliu | Sow farm | Longhui Xinhai | Sow farm |
| Liaocheng Xinhao | Sow farm | Xiajin Agriculture and Animal Husbandry | Sow farm | Xiangzhou Xinhao | Sow farm |
| Huanghua Xinhao | Sow farm | Yijun Xinliu | Sow farm | Liaoning New Hope | Sow farm |
| Huairou Agriculture and Animal Husbandry | Sow farm | Yan'an Benyuan | Sow farm | Xiajin Agriculture and Animal Husbandry | Sow farm |
| Laiyang Xinhao | Sow farm | Hebei Xinhao | Sow farm | Ningming Xinhao | Sow farm |
| Laibin Xinhao | Sow farm | Liaoning New Hope | Sow farm | Liaoning New Hope | Sow farm |
| Yan'an Benyuan | Sow farm | Liaoning New Hope | Sow farm | Tongliao Xinhao | Sow farm |
| Laibin Xinhao | Sow farm | Kangping New Hope | Sow farm | Shibing Xinliu | Sow farm |
| Laiyang Xinhao | Sow farm | Laiyang Xinhao | Sow farm | Guangdong Xinhao | Sow farm |
| Hebei Xinhao | Sow farm | Yanting Xinhao | Sow farm | Weinan Xinliu | Sow farm |
| Hubei Xinhao | Sow farm | Laixi Xinliu | Sow farm | Laibin Xinhao | Sow farm |
| Dong'e Xinliu | Sow farm | Caoxian Xinhao | Sow farm | Caoxian Xinhao | Sow farm |
| Laibin Xinhao | Sow farm | Juye Xinhao | Sow farm | Lingxin Xinliu | Sow farm |
| Caoxian Xinhao | Sow farm | Yancheng Zhongtai | Sow farm | Dong'e Xinliu | Sow farm |
| Huaiyin Xinchi | Sow farm | Kangping New Hope | Sow farm | Tongliao Xinhao | Sow farm |
| Xiajin Agriculture and Animal Husbandry | Sow farm | Laibin Xinhao | Sow farm | Kangping New Hope | Sow farm |
| Weinan Xinliu | Sow farm | Laibin Xinhao | Sow farm | Juye Xinhao | Sow farm |
|  |  | Tongliao Xinhao | Sow farm | Ningming Xinhao | Sow farm |
|  |  | Zhongshan Agriculture and Animal Husbandry | Sow farm | Dong'a Xinliu | Sow farm |
|  |  | Xiangzhou Xinhao | Sow farm | Yijun Xinliu | Sow farm |
|  |  | Weinan Xinliu | Sow farm | Yijun Xinliu | Sow farm |
|  |  | Yan'an Benyuan | Sow farm | Santai Agriculture and Animal Husbandry | Sow farm |
|  |  | Huanghua Xinhao | Sow farm | Zhongshan Agriculture and Animal Husbandry | Sow farm |
|  |  | Laiyang Xinmu | Sow farm | Hainan Xinliu | Sow farm |
|  |  | Kangping Xinwang | Sow farm | Guangdong Xinhao | Sow farm |
|  |  | Dong'e Xinliu | Sow farm | Shibing Xinliu | Sow farm |
|  |  | Laiyang Xinhao | Sow farm | Weinan Xinliu | Sow farm |
|  |  | Liaocheng Xinhao | Sow farm | Huanghua Xinhao | Sow farm |
|  |  |  |  | Hainan Xinliu | Sow farm |
|  |  |  |  | Shibing Xinliu | Sow farm |
|  |  |  |  | Laibin Xinhao | Sow farm |
|  |  |  |  | Tongliao Xinhao | Sow farm |
|  |  |  |  | Laiyang Xinhao | Sow farm |
|  |  |  |  | Dong'a Xinliu | Sow farm |
|  |  |  |  | Yancheng Zhongtai | Sow farm |
|  |  |  |  | Gaotang Xinhao | Sow farm |
|  |  |  |  | Kangping Xinwang | Sow farm |
|  |  |  |  | Laiyang Xinmu | Sow farm |
|  |  |  |  | Kangping Xinwang | Sow farm |
|  |  |  |  | Kangping Xinwang | Sow farm |
|  |  |  |  | Yijun Xinliu | Sow farm |
|  |  |  |  | Dong'a Xinliu | Sow farm |
|  |  |  |  | Yan'an Benyuan | Sow farm |
|  |  |  |  | Weinan Xinliu | Sow farm |
|  |  |  |  | Laixi Xinliu | Sow farm |
|  |  |  |  | Liaoning Xinwang | Sow farm |
|  |  |  |  | Hubei Xinhao | Sow farm |
|  |  |  |  | Laibin Xinhao | Sow farm |
|  |  |  |  | Huairén Agriculture and Animal Husbandry | Sow farm |
|  |  |  |  | Nanchong Xinhao | Sow farm |
|  |  |  |  | Juye Xinhao | Sow farm |
|  |  |  |  | Laiyang Xinhao | Sow farm |
|  |  |  |  | Liaoning Xinwang | Sow farm |
|  |  |  |  | Anyue Xinxinxin | Sow farm |
|  |  |  |  | Xiajin Agriculture and Animal Husbandry | Sow farm |
|  |  |  |  | Laibin Xinhao | Sow farm |
|  |  |  |  | Yanting Xinhao | Sow farm |
|  |  |  |  | Yan'an Benyuan | Sow farm |
|  |  |  |  |  |  |
| **Apr 2021** | | **May 2021** | | **Jun 2021** | |
| **Company (Co., Ltd.)** | **Type of pig farm** | **Company (Co., Ltd.)** | **Type of pig farm** | **Company (Co., Ltd.)** | **Type of pig farm** |
| Laixi Xinliu | Sow farm | Shibing Xinliu | Sow farm | Xinji Xinliu | Sow farm |
| Xiangyang Xinhao | Sow farm | Dingzhou Xinhao | Sow farm | Shibing Xinliu | Sow farm |
| Ningming Xinhao | Fattening farm | Heishan Xinliu | Sow farm | Huanghua Xinhao | Fattening farm |
| Zhenjiang Xinhai | Sow farm | Tongliao Xinhao | Fattening farm | Laiyang Xinmu | Sow farm |
| Binzhou Xinhai | Fattening farm | Shenze Xinhao | Sow farm | Pingfu Nongmu | Sow farm |
| Tangshan Xinhao | Fattening farm | Xinmin New Hope | Sow farm | Guangan Xinhao | Fattening farm |
| Shibing Xinliu | Sow farm | Laiyang Xinmu | Fattening farm | Leshan Nongmu | Fattening farm |
| Neiqiu Xinliu | Fattening farm | Raoyang Xinhao | Sow farm | Laiyang Xinmu | Sow farm |
| Laizhou Xinhai | Sow farm | Laixi Xinliu | Sow farm | Raoyang Xinhao | Sow farm |
| Jiexi Xinliu | Fattening farm | Laizhou Xinhai | Sow farm | Binzhou Xinhai | Sow farm |
| Linhai Xinliu | Fattening farm | Tongliao Xinhao | Sow farm | Guangdong Xinhao | Sow farm |
| Dingzhou Xinhao | Sow farm | Qingfeng Xinliu | Sow farm | Hainan Nongken | Sow farm |
| Zhejiang Xinhai | Fattening farm | Wuhe Xinhai | Sow farm | Anyang Xinliu | Sow farm |
| Jingxian Xinhao | Sow farm | Xiajin Agriculture and Animal Husbandry | Sow farm | Longhui Xinhai | Fattening farm |
| Huanghua Xinhao | Fattening farm | Laizhou Xinhai | Sow farm | Heishan Xinliu | Sow farm |
| Laixi Xinliu | Sow farm | Tongliao Xinhao | Sow farm | Laizhou Xinhai | Fattening farm |
| Wuhe Xinhai | Sow farm | Zhuocheng Xinliu | Fattening farm | Changle Xinhai | Fattening farm |
| Xiangshui Xinchi | Fattening farm | Qinzhou Xinhao | Fattening farm | Tangshan Xinhao | Sow farm |
| Dacheng Xinhao | Sow farm | Pingyuan Xinliu | Sow farm | Anyang Xinliu | Sow farm |
| Heishan Woniou | Sow farm | Jieyang Xinliu | Fattening farm | Hebei Xinhao | Sow farm |
| Gansu Xinliu | Sow farm | Raoyang Xinhao | Sow farm | Xingren Xinliu | Sow farm |
| Chenzhou Xinhao | Fattening farm | Laizhou Xinhai | Fattening farm | Anyang Xinliu | Sow farm |
| Tongliao Xinhao | Sow farm | Tongliao Xinhao | Fattening farm | Changyi Xinhao | Fattening farm |
| Donghai Xinhao | Sow farm | Shuyang Xinliu | Sow farm | Laibin Xinhao | Sow farm |
| Fuxin Xinwang | Sow farm | Raoyang Xinhao | Sow farm | Zhangwu Xinwang | Sow farm |
| Tongliao Xinhao | Sow farm | Huanghua Xinhao | Sow farm | Wannian Xinhai | Sow farm |
| Pengshui Xinliu | Fattening farm | Nanning Xinhao | Sow farm | Ningbo Xinhai | Sow farm |
| Jiexi Xinliu | Fattening farm | Xinmin New Hope | Sow farm | Shanxian Xinhao | Fattening farm |
| Caoxian Xinhao | Fattening farm | Zhenjiang Xinhai | Sow farm | Taian Xincheng | Sow farm |
| Tongliao Xinhao | Sow farm | Fucheng Xinhao | Sow farm | Dingzhou Xinhao | Sow farm |
| Luocheng Xinhao | Sow farm | Huanghua Xinhao | Sow farm | Laizhou Xinhai | Sow farm |
| Tangshan Xinhao | Sow farm | Linshu Breeding Pig | Sow farm | Xianyang Yongxiang | Sow farm |
| Pengshui Xinliu | Fattening farm | Huanghua Xinhao | Sow farm | Lingbao Xinliu | Sow farm |
| Dingzhou Xinhao | Sow farm | Xinji Xinliu | Sow farm | Suining Xinliu | Sow farm |
| Ruyuan Xinhao | Fattening farm | Lingbao Xinliu | Sow farm | Liaocheng Xinhao | Fattening farm |
| Zhangjiakou Xinwang | Sow farm | Xinmin New Hope | Sow farm | Taian Xinliu | Fattening farm |
| Linyi New Pig | Sow farm | Nanning Xinhao | Sow farm | Wuhe Xinhai | Sow farm |
| Raoyang Xinhao | Sow farm | Jian New Drive | Sow farm | Tongliao Xinhao | Fattening farm |
| Yantai Xinhao | Sow farm | Jingxian Xinhao | Sow farm | Tongliao Xinhao | Sow farm |
| Raoyang Xinhao | Sow farm | Dongying Xinhao | Sow farm | Xiangyang Xinhao | Sow farm |
| Xinmin Xinwang | Sow farm | Pucheng Xinliu | Sow farm | Xiajin Nongmu | Sow farm |
| Dingzhou Xinhao | Sow farm | Xiangyang Xinhao | Sow farm | Dingzhou Xinhao | Sow farm |
| Raoyang Xinhao | Sow farm | Xinji Xinliu | Sow farm | Shenze Xinhao | Sow farm |
| Qinzhou Xinhao | Fattening farm | Fucheng Xinhao | Sow farm | Guigang Xinliu | Sow farm |
| Tongliao Xinhao | Sow farm | Xiangshui New Drive | Sow farm | Jian Xinchi | Sow farm |
| Laizhou Xinhai | Fattening farm | Dong'e Xinliu | Sow farm | Jie Xi Xinliu | Fattening farm |
| Dongying Xinhao | Fattening farm | Jian New Drive | Sow farm | Jiangxi Xinliu | Sow farm |
| Lingbao Xinliu | Sow farm | Ruyuan Xinhao | Fattening farm | Nanning Xinliu | Sow farm |
| Jian Xinchi | Sow farm | Xinmin New Hope | Sow farm | Ruzhou Quansheng | Sow farm |
| Pingyuan Xinliu | Sow farm | Zhengzhou Quansheng | Sow farm | Xiajin Nongmu | Sow farm |
| Shanxian Xinliu | Sow farm | Beijing Xinliu | Sow farm | Laixi Xinliu | Sow farm |
| Xiajin Agriculture and Animal Husbandry | Sow farm | Anyang Xinliu | Sow farm | Wuhe Xinhai | Sow farm |
| Raoyang Xinhao | Sow farm | Laiyang Xinmu | Sow farm | Xiajin Nongmu | Sow farm |
| Huanghua Xinhao | Sow farm | Zhuocheng Xinliu | Fattening farm | Ruzhou Quansheng | Fattening farm |
| Gansu Xinhao | Sow farm | Wuhe Xinhai | Sow farm | Dongying Xinhao | Sow farm |
| Zhangwu Xinwang | Sow farm | Pucheng Xinliu | Sow farm | Jian Xinchi | Sow farm |
| Yantai Xinhao | Sow farm | Anyang Xinliu | Sow farm | Linyi Xinhao | Sow farm |
| Wulian Xinhao | Sow farm | Tongliao Xinhao | Sow farm | Xiangyang Xinhao | Sow farm |
| Jian Xinchi | Sow farm | Xiangyang Xinhao | Sow farm | Laixi Xinliu | Sow farm |
| Huanghua Xinhao | Sow farm | Liaocheng Xinhao | Fattening farm | Zhenjiang Xinhai | Sow farm |
| Dongying Xinhao | Sow farm | Wuming Xinliu | Sow farm | Liaocheng Xinhao | Fattening farm |
| Anyang Xinliu | Sow farm | Meishan Xinhai | Fattening farm | Tongliao Xinhao | Fattening farm |
| Tongliao Xinhao | Sow farm | Xinmin New Hope | Sow farm | Jiangxi Xinliu | Sow farm |
| Wuqi Xinliu | Sow farm | Guigang Xinliu | Sow farm | Guizhou Xinliu | Sow farm |
| Laizhou Xinhai | Fattening farm | Luocheng Xinhao | Sow farm | Shanxian Xinliu | Sow farm |
| Zhangwu Xinwang | Sow farm | Zhejiang Xinhai | Fattening farm | Shenze Xinhao | Sow farm |
| Dongying Xinhao | Fattening farm | Zhenjiang Xinhai | Sow farm | Qingyuan Xinhao | Fattening farm |
| Liaocheng Xinhao | Sow farm | Gaomi Xinliu | Sow farm | Guigang Xinliu | Sow farm |
| Tongliao Xinhao | Sow farm | Qingyuan Xinhao | Fattening farm | Linyi Xinhao | Sow farm |
| Hezhou Xinhao | Fattening farm | Liaocheng Xinhao | Fattening farm | Anyang Xinliu | Sow farm |
| Ruyuan Xinhao | Sow farm | Huanghua Xinhao | Sow farm | Shenze Xinhao | Sow farm |
| Anyang Xinliu | Fattening farm | Zhangwu New Hope | Sow farm | Pucheng Xinliu | Sow farm |
| Donghai Xinhao | Sow farm | Zhenjiang Xinhai | Sow farm | Guangan Xinhao | Fattening farm |
| Shibing Xinliu | Sow farm | Leshan Agriculture and Animal Husbandry | Fattening farm | Zhangwu Xinwang | Sow farm |
| Nantong Xinhai | Sow farm | Ruyuan Xinhao | Fattening farm | Pucheng Xinliu | Sow farm |
| Luocheng Xinhao | Sow farm | Zhaoqing Xinhao | Fattening farm | Shuyang Xinliu | Sow farm |
| Luocheng Xinhao | Sow farm | Henan Agriculture and Animal Husbandry | Fattening farm | Hebei Xinhao | Sow farm |
| Zhangwu Xinwang | Fattening farm | Dongying Xinhao | Sow farm | Laiyang Xinmu | Fattening farm |
| Laizhou Xinhai | Sow farm | Hengyang Xinhao | Fattening farm | Tianquan Xinliu | Sow farm |
| Luocheng Xinhao | Sow farm | Liaocheng Xinhao | Fattening farm | Dongying Xinhao | Sow farm |
| Wuhe Xinhai | Sow farm | Tongliao Xinhao | Fattening farm | Tianquan Xinliu | Sow farm |
| Dongying Xinhao | Sow farm | Xiajin Agriculture and Animal Husbandry | Sow farm | Xinji Xinliu | Sow farm |
| Xiajin Agriculture and Animal Husbandry | Sow farm | Taian New Drive | Sow farm | Tangshan Xinhao | Sow farm |
| Luocheng Xinhao | Sow farm | Binzhou Xinhai | Fattening farm | Xiangshui Xincheng | Fattening farm |
| Weinan Xinliu | Sow farm | Laixi Xinliu | Sow farm | Huanghua Xinhao | Sow farm |
| Laixi Xinliu | Sow farm | Xiajin Agriculture and Animal Husbandry | Sow farm | Santai Nongmu | Sow farm |
| Dongying Xinhao | Sow farm | Lingao Xinliu | Fattening farm | Nanning Xinhao | Sow farm |
| Xiangshui Xinchi | Fattening farm | Neiqiu Xinliu | Fattening farm | Huaiyin Xincheng | Sow farm |
| Huaiyin Xinchi | Sow farm | Wuhe Xinhai | Sow farm | Xiangshui Xincheng | Fattening farm |
| Anyang Xinliu | Sow farm | Tangshan Xinhao | Sow farm | Kangping Xinwang | Sow farm |
| Tongliao Xinhao | Sow farm | Anyang Xinliu | Sow farm | Anyue Xingxin | Sow farm |
| Qingfeng Xinliu | Sow farm | Changyi Xinhao | Fattening farm | Dezhou Xinhao | Sow farm |
| Shuozhou Xinhao | Fattening farm | Gansu Xinhao | Sow farm | Leting Xinhai | Fattening farm |
| Laixi Xinliu | Sow farm | Tianxin Xinliu | Sow farm | Leting Xinhai | Fattening farm |
| Zhenyuan Xinhai | Sow farm | Weinan Xinliu | Sow farm | Nantong Xinhai | Sow farm |
| Sichuan Xinhao | Sow farm | Heishan Xinliu | Sow farm | Fucheng Xinhao | Sow farm |
| Jingxian Xinhao | Sow farm | Yantai Xinhao | Sow farm | Luocheng Xinhao | Fattening farm |
| Huanghua Xinhao | Sow farm | Tangshan Xinhao | Sow farm | Xiajin Nongmu | Sow farm |
| Jiangxi Xinliu | Sow farm | Xiangshui New Drive | Fattening farm | Xiajin Nongmu | Sow farm |
| Fuxin Xinwang | Sow farm | Xiajin Agriculture and Animal Husbandry | Sow farm | Hebei Xinhao | Sow farm |
| Fucheng Xinhao | Sow farm | Yichun New Hope | Fattening farm | Heishan Xinliu | Sow farm |
| Guanling Xinhai | Fattening farm | Caoxian Xinhao | Fattening farm | Linshu Zhongzhu | Sow farm |
| Fucheng Xinhao | Sow farm | Ruzhou Quansheng | Fattening farm | Jingxian Xinhao | Sow farm |
| Gaomi Xinliu | Sow farm | Shanxian Xinhao | Sow farm | Zhejiang Xinhai | Fattening farm |
| Chenzhou Xinhao | Sow farm | Zhangwu New Hope | Sow farm | Gaotang Xinhao | Sow farm |
| Raoyang Xinhao | Sow farm | Xiajin Agriculture and Animal Husbandry | Sow farm | Liuzhou Xinhao | Fattening farm |
| Wulian Xinhao | Sow farm | Wuhe Xinhai | Sow farm | Baoji Jinfeng | Sow farm |
| Zhangwu Xinwang | Fattening farm | Xinmin New Hope | Sow farm | Suining Xinliu | Sow farm |
| Tongliao Xinhao | Sow farm | Yantai Xinhao | Sow farm | Lingao Xinliu | Fattening farm |
| Tianxin Xinliu | Sow farm | Shenze Xinhao | Sow farm | Shanxian Xinhao | Sow farm |
| Ruyuan Xinhao | Fattening farm | Wuqi Xinliu | Sow farm | Gaomi Xinliu | Fattening farm |
| Xiangshui Xinchi | Fattening farm | Tongliao Xinhao | Fattening farm | Fucheng Xinhao | Sow farm |
| Heyang Agriculture and Animal Husbandry | Fattening farm | Huanghua Xinhao | Sow farm | Zhu Cheng Xinliu | Fattening farm |
| Dingzhou Xinhao | Sow farm | Xinji Xinliu | Sow farm | Qingfeng Xinliu | Sow farm |
| Yantai Xinhao | Sow farm | Zhangwu New Hope | Sow farm | Heishan Xinliu | Sow farm |
| Huanghua Xinhao | Sow farm | Guangyuan Xinhao | Sow farm | Binzhou Xinhai | Fattening farm |
| Laiyang Xinmu | Sow farm | Gaotang Xinhao | Sow farm | Pengshui Xinliu | Fattening farm |
| Pucheng Xinliu | Sow farm | Zhenjiang Xinhai | Sow farm | Luocheng Xinhao | Sow farm |
| Fucheng Xinhao | Sow farm | Shangcai Xinliu | Fattening farm | Nanning Xinliu | Fattening farm |
| Xuanwei Xinliu | Sow farm | Yantai Xinhao | Sow farm | Hengyang Xinhao | Fattening farm |
| Tongliao Xinhao | Sow farm | Dingzhou Xinhao | Sow farm | Jian Xinchi | Fattening farm |
| Yantai Xinhao | Sow farm | Lianjiang Xinhao | Fattening farm | Yantai Xinhao | Sow farm |
| Danxian Xinhao | Sow farm | Liuzhou Xinhao | Fattening farm | Jingxian Xinhao | Sow farm |
| Chenzhou Xinhao | Sow farm | Laizhou Xinhai | Sow farm | Yantai Xinhao | Sow farm |
| Heishan Xinliu | Sow farm | Dongying Xinhao | Sow farm | Xiangshui Xincheng | Fattening farm |
| Shangcai Xinliu | Fattening farm | Santai Agriculture and Animal Husbandry | Sow farm | Laixi Xinliu | Sow farm |
| Ruyuan Xinhao | Fattening farm | Donghai Xinhao | Sow farm | Dongying Xinhao | Sow farm |
| Caoxian Xinhao | Sow farm | Kangping New Hope | Sow farm | Lingao Xinliu | Fattening farm |
| Anyang Xinliu | Sow farm | Dingzhou Xinhao | Sow farm | Dongying Xinhao | Sow farm |
| Jizhou Xinhao | Fattening farm | Fuping Xinliu | Sow farm | Luding Xinyue | Fattening farm |
| Laizhou Xinhai | Sow farm | Huaiyin New Drive | Sow farm | Laixi Xinliu | Sow farm |
| Sichuan Xinhao | Sow farm | Changle Xinhai | Fattening farm | Henan Nongmu | Fattening farm |
| Gaotang Xinhao | Sow farm | Guang'an Xinhao | Fattening farm | Ruyuan Xinhao | Fattening farm |
| Zhengzhou Quansheng | Sow farm | Laiyang Xinmu | Sow farm | Longhui Xinhai | Sow farm |
| Guang'an Xinhao | Sow farm | Pengshui Xinliu | Fattening farm | Laizhou Xinhai | Sow farm |
| Guigang Xinliu | Fattening farm | Chenzhou Xinhao | Fattening farm | Hainan Xinliu | Sow farm |
| Liaocheng Xinhao | Fattening farm | Laizhou Xinhai | Sow farm | Laizhou Xinhai | Sow farm |
| Dingzhou Xinhao | Sow farm | Nanchong Xinhao | Fattening farm | Henan Nongmu | Sow farm |
| Laizhou Xinhai | Sow farm | Laizhou Xinhai | Sow farm | Shangcai Xinliu | Fattening farm |
| Laiyang Xinmu | Sow farm | Caoxian Xinhao | Sow farm | Sichuan Xinhao | Sow farm |
| Taian Xinchi | Sow farm | Xinji Xinliu | Sow farm | Laiyang Xinmu | Sow farm |
| Zhuocheng Xinliu | Fattening farm | Sichuan Xinhao | Sow farm | Yantai Xinhao | Sow farm |
| Wannian Xinhai | Sow farm | Qingfeng Xinliu | Sow farm | Laizhou Xinhai | Sow farm |
| Xinmin Xinwang | Sow farm | Jieyang Xinliu | Fattening farm | Dong'e Xinliu | Fattening farm |
| Anyang Xinliu | Sow farm | Laixi Xinliu | Sow farm | Hebei Xinhao | Sow farm |
| Jiangxi Xinliu | Sow farm | Shibing Xinliu | Sow farm | Caoxian Xinhao | Sow farm |
| Hainan Xinliu | Sow farm | Hainan Xinliu | Sow farm | Dongying Xinhao | Sow farm |
| Zhangwu Xinwang | Fattening farm | Yingtan Xinliu | Sow farm | Sichuan Xinhao | Sow farm |
| Sichuan Xinhao | Sow farm | Hengnan Muyun | Sow farm | Sichuan Xinhao | Sow farm |
| Dongying Xinhao | Sow farm | Anyang Xinliu | Sow farm | Laixi Xinliu | Sow farm |
| Taian Xinliu | Fattening farm | Wulian Xinhao | Sow farm | Dezhou Xinhao | Sow farm |
| Laixi Xinliu | Sow farm | Guang'an Xinhao | Sow farm | Laizhou Xinhai | Sow farm |
| Xiangfan Xinhao | Sow farm | Ruzhou Quansheng | Sow farm | Taian Xinliu | Fattening farm |
| Binzhou Xinhai | Sow farm | Wannian Xinhai | Sow farm | Jizhou Xinhao | Fattening farm |
| Langzhong Xinliu | Fattening farm | Heishan Wolniu | Sow farm | Qingyuan Xinhao | Fattening farm |
| Fuping Xinliu | Sow farm | Dezhou Xinhao | Sow farm | Laixi Xinliu | Sow farm |
| Nanning Xinliu | Sow farm | Sichuan Xinhao | Sow farm | Sichuan Xinhao | Sow farm |
| Xinmin Xinwang | Sow farm | Lingao Xinliu | Fattening farm | Jiangxi Xin Liu | Sow farm |
| Danxian Xinliu | Sow farm | Jizhou Xinhao | Fattening farm | Taiqian Xin Liu | Fattening farm |
| Xiajin Agriculture and Animal Husbandry | Sow farm | Laixi Xinliu | Sow farm | Qingfeng Xin Liu | Sow farm |
| Dongying Xinhao | Sow farm | Tongliao Xinhao | Sow farm | Neiqiu Xin Liu | Fattening farm |
| Yichun Xinwang | Sow farm | Yantai Xinhao | Sow farm | Rugao Xin Hao | Fattening farm |
| Dongying Xinhao | Sow farm | Sichuan Xinhao | Sow farm | Guanglv Xin Liu | Sow farm |
| Xinji Xinliu | Sow farm | Guanling Xinhai | Fattening farm | Zhejiang Xin Hai | Sow farm |
| Shenze Xinhao | Sow farm | Weinan Xin Liu | Sow farm | Linyi Zhongzhu | Sow farm |
| Xiangyang Xinhao | Sow farm | Taiqian Xin Liu | Fattening farm | Caoxian Xin Hao | Fattening farm |
| Taian Xinliu | Fattening farm | Xiangyang Xinhao | Sow farm | Qingfeng Xin Liu | Sow farm |
| Laixi Xinliu | Sow farm | Jingxian Xinhao | Sow farm | Liaoning Xin Wang | Sow farm |
| Xiajin Agriculture and Animal Husbandry | Sow farm | Henan Nongmu | Sow farm | Wuqi Xin Liu | Sow farm |
| Zhangwu New Hope | Fattening farm | Longhui Xin Hai | Sow farm | Nanning Xin Hao | Fattening farm |
| Zhangwu New Hope | Sow farm | Heishan Xin Liu | Sow farm | Tongliao Xin Hao | Fattening farm |
| Kangping New Hope | Sow farm | Taiqian Xin Liu | Fattening farm | Lingbao Xin Liu | Sow farm |
| Xiajin Agriculture and Animal Husbandry | Sow farm | Laixi Xin Liu | Sow farm | Guanling Xin Hai | Fattening farm |
| Anhui Xinliu | Sow farm | Fucheng Xinhao | Sow farm | Anyang Xin Liu | Sow farm |
| Wuhe Xinhai | Sow farm | Laixi Xin Liu | Sow farm | Shuozhou Xin Hao | Fattening farm |
| Lingao Xinliu | Fattening farm | Wuhe Xin Hai | Sow farm | Gaomi Xin Liu | Sow farm |
| Fucheng Xinhao | Sow farm | Luocheng Xinhao | Sow farm | Yantai Xin Hao | Sow farm |
| Binzhou Xinhai | Sow farm | Zhangjiakou Xin Wang | Sow farm | Zhu Cheng Xin Liu | Fattening farm |
| Sichuan Xinhao | Sow farm | Binzhou Xin Hai | Sow farm | Dongying Xin Hao | Sow farm |
| Weinan Xinliu | Fattening farm | Dongying Xinhao | Sow farm | Lezhi Xin Hai | Fattening farm |
| Linyi Breeding Pig | Sow farm | Linyi Zhongzhu | Sow farm | Lezhi Xin Hai | Fattening farm |
| Yantai Xinhao | Sow farm | Tongcheng Xin Liu | Sow farm | Ji'an Xin Chi | Sow farm |
| Tongcheng Xinliu | Sow farm | Huanghua Xinhao | Sow farm | Laixi Xin Liu | Sow farm |
| Zhejiang Xinhai | Sow farm | Qingfeng Xin Liu | Sow farm | Shibing Xin Liu | Sow farm |
| Shanxian Xinhao | Sow farm | Shuozhou Xinhao | Fattening farm | Guangan Xin Hao | Sow farm |
| Laizhou Xinhai | Fattening farm | Anyang Xin Liu | Sow farm | Zhangwu Xin Wang | Fattening farm |
| Laizhou Xinhai | Sow farm | Luocheng Xinhao | Sow farm | Sichuan Xin Hao | Sow farm |
| Guigang Xinliu | Sow farm | Meishan Xin Hai | Sow farm | Zhenjiang Xin Hai | Sow farm |
| Guangyuan Xinhao | Sow farm | Luocheng Xinhao | Sow farm | Xinji Xin Liu | Sow farm |
| Dongying Xinhao | Sow farm | Luocheng Xinhao | Sow farm | Pengshui Xin Liu | Fattening Farm |
| Yantai Xinhao | Sow farm | Guizhou Xin Liu | Sow farm | Wuhe Xin Hai | Sow farm |
| Xinji Xinliu | Sow farm | Gaomi Xin Liu | Sow farm | Huanghua Xin Hao | Sow farm |
| Xinji Xinliu | Sow farm | Huanghua Xinhao | Sow farm | Xiangshui Xin Chi | Fattening Farm |
| Xinmin New Hope | Sow farm | Dongying Xinhao | Sow farm | Tangshan Xin Hao | Sow farm |
| Zhangwu New Hope | Sow farm | Anhui Xin Liu | Sow farm | Dongying Xin Hao | Sow farm |
| Laizhou Xinhai | Sow farm | Jian Xinchi | Sow farm | Heishan Xin Liu | Sow farm |
| Jingxian Xinhao | Sow farm | Dongying Xinhao | Sow farm | Anhui Xin Liu | Sow farm |
| Heishan Xinliu | Sow farm | Dongying Xinhao | Sow farm | Ruzhou Quansheng | Sow farm |
| Liuzhou Xinhao | Fattening farm | Bijie Xin Liu | Fattening farm | Zhangwu Xin Wang | Sow farm |
| Laizhou Xinhai | Sow farm | Weinan Xin Liu | Sow farm | Fucheng Xin Hao | Sow farm |
| Chenzhou Xinhao | Sow farm | Xiangshui Xin Chi | Fattening farm | Gaomi Xin Liu | Sow farm |
| Xiajin Agriculture and Animal Husbandry | Sow farm | Qingfeng Xin Liu | Sow farm | Shanxian Xin Hao | Sow farm |
| Guigang Xinliu | Fattening farm | Zhejiang Xin Hai | Sow farm | Gansu Xin Hao | Sow farm |
| Chenzhou Xinhao | Fattening farm | Sichuan Xinhao | Sow farm | Xiangyang Xin Hao | Sow farm |
| Guangan Xinhao | Sow farm | Shanxian Xinhao | Sow farm | Liaoning Xintao Wang | Sow farm |
| Guigang Xinliu | Sow farm | Xiangshui Xin Chi | Fattening farm | Guigang Xin Liu | Sow farm |
| Shenze Xinhao | Sow farm | Xinji Xin Liu | Sow farm | Wulian Xin Hao | Sow farm |
| Henan Agriculture and Animal Husbandry | Sow farm | Dongying Xinhao | Sow farm | Yantai Xin Hao | Sow farm |
| Santai Agriculture and Animal Husbandry | Sow farm | Gansu Xin Liu | Sow farm | Dongying Xin Hao | Sow farm |
| Yantai Xinhao | Sow farm | Heishan Xin Liu | Sow farm | Laizhou Xin Hai | Sow farm |
| Ruzhou Quansheng | Sow farm | Huanghua Xinhao | Fattening farm | Yingtan Xin Liu | Sow farm |
| Laizhou Xinhai | Sow farm | Pengshui Xin Liu | Fattening farm | Liaoning Xintao Wang | Sow farm |
| Binzhou Xinhai | Sow farm | Laizhou Xin Hai | Sow farm | Gansu Xin Liu | Sow farm |
| Dongying Xinhao | Sow farm | Laizhou Xin Hai | Sow farm | Guangan Xin Hao | Sow farm |
| Jian New Drive | Fattening farm | Yantai Xinhao | Sow farm | Donghai Xin Hao | Sow farm |
| Gaomi Xinliu | Sow farm | Zhenyuan Xin Hai | Sow farm | Yantai Xin Hao | Sow farm |
| Lianjiang Xinhao | Fattening farm | Laixi Xin Liu | Sow farm | Zhenjiang Xin Hai | Sow farm |
| Liaoning New Taowang | Sow farm | Binzhou Xin Hai | Sow farm | Laizhou Xin Hai | Sow farm |
| Dongying Xinhao | Sow farm | Fuxin Xin Wang | Sow farm | Laizhou Xin Hai | Sow farm |
| Weinan Xinliu | Sow farm | Xinji Xin Liu | Sow farm | Shanxian Xin Hao | Sow farm |
| Tongliao Xinhao | Sow farm | Jian Xinchi | Fattening farm | Rongchang Pig Farm | Sow farm |
| Gaomi Xinliu | Sow farm | Lingbao Xin Liu | Sow farm | Zhenyuan Xin Hai | Sow farm |
| Pengshui Xinliu | Fattening farm | Yantai Xinhao | Sow farm | Dongying Xin Hao | Sow farm |
| Qingfeng Xinliu | Sow farm | Laizhou Xin Hai | Sow farm | Dongying Xin Hao | Sow farm |
| Liaoning New Taowang | Sow farm | Hezhou Xinhao | Fattening farm | Laizhou Xin Hai | Sow farm |
| Lezhi Xinhai | Fattening farm | Laizhou Xin Hai | Sow farm | Yantai Xin Hao | Sow farm |
| Rongchang Pig Farm | Sow farm | Liaoning Xin Tao Wang | Sow farm | Luocheng Xin Hao | Sow farm |
| Lingbao Xinliu | Sow farm | Dongying Xinhao | Sow farm | Zhangwu Xin Wang | Fattening Farm |
| Laixi Xinliu | Sow farm | Fucheng Xinhao | Sow farm | Xinji Xin Liu | Sow farm |
| Chongren Xinhai | Fattening farm | Rongchang Pig Farm | Sow farm | Binzhou Xin Hai | Sow farm |
| Laixi Xinliu | Sow farm | Zhangwu Xin Wang | Fattening farm | Shanxian Xin Hao | Sow farm |
| Shanxian Xinhao | Sow farm | Gaomi Xin Liu | Sow farm | Gaotang Xin Hao | Sow farm |
| Tongliao Xinhao | Sow farm | Yantai Xinhao | Sow farm | Zhangjiakou Xin Wang | Sow farm |
| Rugao Xinhao | Fattening farm | Liaoning Xin Tao Wang | Sow farm | Dongying Xin Hao | Sow farm |
| Heishan Xinliu | Fattening farm | Pucheng Xin Liu | Sow farm | Dongying Xin Hao | Sow farm |
| Leshan Agriculture and Animal Husbandry | Fattening farm | Jiangxi Xin Liu | Sow farm | Jiangxi Xin Liu | Sow farm |
| Hainan Agricultural Reclamation | Sow farm | Wulian Xinhao | Sow farm | Luocheng Xin Hao | Sow farm |
| Dongying Xinhao | Sow farm | Guang'an Xinhao | Sow farm | Gansu Xin Hao | Sow farm |
| Kangping New Hope | Fattening farm | Pengshui Xin Liu | Fattening farm | Jingxian Xin Hao | Sow farm |
| Santai Agriculture and Animal Husbandry | Fattening farm | Lingbao Xin Liu | Sow farm | Luocheng Xin Hao | Sow farm |
| Kangping New Hope | Sow farm | Kangping Xin Wang | Sow farm | Wuhe Xin Hai | Sow farm |
| Xiajin Agriculture and Animal Husbandry | Sow farm | Shanxian Xinhao | Sow farm | Kangping Xin Wang | Sow farm |
| Dongying Xinhao | Sow farm | Shanxian Xin Liu | Sow farm | Liaoning Xintao Wang | Sow farm |
| Lingbao Xinliu | Sow farm | Tongliao Xinhao | Sow farm | Wannian Xin Hai | Fattening Farm |
| Liaoning New Taowang | Sow farm | Laizhou Xin Hai | Sow farm | Luocheng Xin Hao | Sow farm |
| Guangan Xinhao | Fattening farm | Heishan Xin Liu | Sow farm | Tangshan Xin Hao | Sow farm |
| Shanxian Xinhao | Sow farm | Shanxian Xinhao | Sow farm | Anyang Xin Liu | Sow farm |
| Zhucheng Xinliu | Sow farm | Dongying Xinhao | Sow farm | Nanning Xin Hao | Sow farm |
| Hainan Agricultural Reclamation | Sow farm | Santai Nongmu | Sow farm | Pucheng Xin Liu | Sow farm |
| Yingcheng Xinhao | Sow farm | Dongying Xinhao | Sow farm | Wuhe Xin Hai | Sow farm |
| Guangan Xinhao | Sow farm | Weihui Xin Chi | Sow farm | Ji'an Xin Chi | Sow farm |
| Dongying Xinhao | Sow farm | Liaoning Xin Tao Wang | Sow farm | Dongying Xin Hao | Sow farm |
| Xiangshui New Drive | Fattening farm | Meishan Xin Hai | Sow farm | Dongying Xin Hao | Sow farm |
| Guangan Xinhao | Sow farm | Lezhi Xin Hai | Fattening farm | Liaocheng Xin Hao | Sow farm |
| Xingren Xinliu | Sow farm | Anyue Xing Xin Xin | Sow farm | Hezhou Xin Hao | Fattening Farm |
| Tangshan Xinhao | Sow farm | Chenzhou Xinhao | Sow farm | Dongying Xin Hao | Sow farm |
| Yantai Xinhao | Sow farm | Yantai Xinhao | Sow farm | Guangan Xin Hao | Sow farm |
| Xinmin New Hope | Sow farm | Laixi Xin Liu | Sow farm | Lingbao Xin Liu | Sow farm |
| Binzhou Xinhai | Sow farm | Xiangyang Xinhao | Sow farm | Pengshui Xin Liu | Fattening Farm |
| Laixi Xinliu | Sow farm | Yantai Xinhao | Sow farm | Dongying Xin Hao | Sow farm |
| Laixi Xinliu | Sow farm | Dongying Xinhao | Sow farm | Laizhou Xin Hai | Sow farm |
| Heishan Xinliu | Sow farm | Zhuocheng Xin Liu | Sow farm | Yantai Xin Hao | Sow farm |
| Yantai Xinhao | Sow farm | Jiangxi Xin Liu | Sow farm | Yinbao Breeding | Sow farm |
| Longzhou Xinhao | Sow farm | Wuhe Xin Hai | Sow farm | Yantai Xin Hao | Sow farm |
| Huanghua Xinhao | Fattening farm | Guigang Xin Liu | Fattening farm | Binzhou Xin Hai | Sow farm |
| Wuhe Xinhai | Sow farm | Wuming Xin Liu | Fattening farm | Yingtan Xin Liu | Fattening Farm |
| Lezhi Xinhai | Fattening farm | Gaotang Xinhao | Sow farm | Zhu Cheng Xin Liu | Sow farm |
| Xiangyang Xinhao | Sow farm | Guang'an Xinhao | Sow farm | Guangan Xin Hao | Sow farm |
| Longzhou Xinhao | Sow farm | Laixi Xin Liu | Sow farm | Guigang Xin Liu | Fattening Farm |
| Gansu Xinliu | Fattening farm | Meishan Xin Hai | Fattening farm | Xiangyang Xin Hao | Sow farm |
| Dongying Xinhao | Sow farm | Liaocheng Xinhao | Sow farm | Fuxin Xin Wang | Sow farm |
| Ruzhou Quansheng | Fattening farm | Dongying Xinhao | Sow farm | Juye Xin Hao | Sow farm |
| Guangan Xinhao | Sow farm | Guang'an Xinhao | Sow farm | Laixi Xin Liu | Sow farm |
| Yinbao Breeding | Sow farm | Yinbao Breeding | Sow farm | Longzhou Xin Hao | Fattening Farm |
| Donghai Xinhao | Sow farm | Jingxian Xinhao | Sow farm | Gansu Xin Hao | Sow farm |
| Tongliao Xinhao | Sow farm | Longzhou Xinhao | Sow farm | Wuming Xin Liu | Sow farm |
| Gansu Xinhao | Sow farm | Liaocheng Xinhao | Sow farm | Longzhou Xin Hao | Sow farm |
| Weihui New Drive | Sow farm | Hebei Xinhao | Sow farm | Wuhe Xin Hai | Sow farm |
| Xiangshui New Drive | Fattening farm | Dongying Xinhao | Sow farm | Weinan Xin Liu | Sow farm |
| Zhucheng Xinliu | Sow farm | Ruzhou Quansheng | Sow farm | Heishan Xin Liu | Sow farm |
| Lezhi Xinhai | Fattening farm | Xinji Xin Liu | Sow farm | Guangan Xin Hao | Sow farm |
| Anyue Xing Xin Xin | Sow farm | Nantong Xinha | Sow farm | Guangyuan Xinhao | Sow farm |
| Longzhou Xinhao | Sow farm | Xinji Xinliu | Sow farm | Shanxian Xinhao | Sow farm |
| Dongying Xinhao | Sow farm | Shenze Xinhao | Sow farm | Jiaxiang Xinliu | Sow farm |
| Shanxian Xinhao | Sow farm | Zhangwu Xinwang | Fattening farm | Longzhou Xinhao | Sow farm |
| Dongying Xinhao | Sow farm | Tongliao Xinhao | Sow farm | Dongying Xinhao | Sow farm |
| Yingtan Xin Liu | Fattening farm | Xuanwei Xinliu | Sow farm | Bijie Xinliu | Fattening Farm |
| Gansu Xinhao | Fattening farm | Changyi Xinhao | Sow farm | Gansu Xinhao | Sow farm |
| Nanchong Xinhao | Fattening farm | Guangyuan Xinhao | Sow farm | Weinan Xinliu | Sow farm |
| Qingfeng Xin Liu | Sow farm | Gansu Xinhao | Sow farm | Hebei Xinhao | Sow farm |
| Qingfeng Xin Liu | Sow farm | Guangan Xinhao | Sow farm | Shanxian Xinliu | Sow farm |
| Liaocheng Xinhao | Sow farm | Letong Xinhai | Fattening farm | Zhangwu Xinwang | Sow farm |
| Changyi Xinhao | Sow farm | Gansu Xinliu | Sow farm | Weinan Xinliu | Sow farm |
| Xinji Xin Liu | Sow farm | Rugao Xinhao | Fattening farm | Liaocheng Xinhao | Sow farm |
| Ruyuan Xinhao | Fattening farm | Gansu Xinliu | Sow farm | Longzhou Xinhao | Sow farm |
| Hebei Xinhao | Sow farm | Longzhou Xinhao | Sow farm | Liaoning Xintao Wang | Sow farm |
| Longzhou Xinhao | Sow farm | Letong Xinhai | Fattening farm | Laiyang Xinhao | Sow farm |
| Liaocheng Xinhao | Sow farm | Yingcheng Xinhao | Sow farm | Guangyuan Xinhao | Sow farm |
| Xinji Xin Liu | Sow farm | Zhu Cheng Xinliu | Sow farm | Liaocheng Xinhao | Sow farm |
| Shenze Xinhao | Sow farm | Changyi Xinhao | Sow farm | Ruyuan Xinhao | Sow farm |
| Gansu Xinhao | Sow farm | Letong Xinhai | Fattening farm | Zhu Cheng Xinliu | Fattening farm |
| Gaotang Xinhao | Sow farm | Dongying Xinhao | Sow farm | Changyi Xinhao | Sow farm |
| Heishan Xin Liu | Sow farm | Yingtan Xinliu | Fattening farm | Weinan Xinliu | Sow farm |
| Jiaxiang Xin Liu | Sow farm | Shenze Xinhao | Sow farm | Wulian Xinhao | Sow farm |
| Huanghua Xinhao | Sow farm | Xiajin Nongmu | Sow farm | Longzhou Xinhao | Sow farm |
| Xinji Xin Liu | Sow farm | Dongying Xinhao | Sow farm | Changyi Xinhao | Sow farm |
| Qingyuan Xinhao | Fattening farm | Nanning Xinliu | Sow farm | Laixi Xinliu | Sow farm |
| Longzhou Xinhao | Sow farm | Tongliao Xinhao | Sow farm | Longzhou Xinhao | Sow farm |
| Zhucheng Xin Liu | Sow farm | Chenzhou Xinhao | Sow farm | Santai Nongmu | Sow farm |
| Tongliao Xinhao | Sow farm | Shenze Xinhao | Sow farm | Leshan Nongmu | Sow farm |
| Leshan Nongmu | Sow farm | Guanglü Xinliu | Sow farm | Ruzhou Quansheng | Sow farm |
| Liaoning Xin Taowang | Sow farm | Xiangyang Xinhao | Sow farm | Longzhou Xinhao | Sow farm |
| Nantong Xin Hai | Sow farm | Longzhou Xinhao | Sow farm | Zhangwu Xinwang | Sow farm |
| Tongliao Xinhao | Sow farm | Danxian Xinhao | Sow farm | Changle Xinhai | Fattening farm |
| Longzhou Xinhao | Sow farm | Xianyang Yongxiang | Sow farm | Liangshan Xinliu | Sow farm |
| Wannian Xin Hai | Fattening farm | Weinan Xinliu | Sow farm | Hubei Xinhao | Sow farm |
| Gansu Xin Liu | Sow farm | Gansu Xinhao | Sow farm | Heishan Woniou | Sow farm |
| Lezhi Xin Hai | Fattening farm | Liaoning Xintao Wang | Sow farm | Yan'an Benyuan | Sow farm |
| Gaomi Xin Liu | Sow farm | Laixi Xinliu | Sow farm | Xuanwei Xinliu | Sow farm |
| Guangyuan Xinhao | Sow farm | Leshan Nongmu | Sow farm | Gansu Xinhao | Sow farm |
| Tongliao Xinhao | Sow farm | Longzhou Xinhao | Sow farm | Xinji Xinliu | Sow farm |
| Weinan Xin Liu | Sow farm | Weinan Xinliu | Sow farm | Tangshan Xinhao | Sow farm |
| Hengnan Muyun | Sow farm | Zhu Cheng Xinliu | Sow farm | Xinji Xinliu | Sow farm |
| Hubei Xinhao | Sow farm | Xinji Xinliu | Sow farm | Qinghua Nongmu | Sow farm |
| Santai Nongmu | Sow farm | Tongliao Xinhao | Sow farm | Liaoning Xintao Wang | Sow farm |
| Huanghua Xinhao | Sow farm | Longzhou Xinhao | Sow farm | Zhu Cheng Xinliu | Sow farm |
| Yichun Xinwang | Fattening farm | Yichun Xinwang | Sow farm | Laixi Xinliu | Sow farm |
| Guizhou Xin Liu | Sow farm | Ruyuan Xinhao | Sow farm | Zhenjiang Xinhai | Sow farm |
| Tongliao Xinhao | Sow farm | Jiaxiang Xinliu | Sow farm | Tongliao Xinhao | Sow farm |
| Gansu Xinhao | Sow farm | Longzhou Xinhao | Sow farm | Gaotang Xinhao | Sow farm |
| Guang'an Xinhao | Fattening farm | Juye Xinhao | Sow farm | Gansu Xinhao | Sow farm |
| Laiyang Xinhao | Sow farm | Zhangwu Xinwang | Sow farm | Pingfu Nongmu | Sow farm |
| Hebei Xinhao | Sow farm | Santai Nongmu | Sow farm | Chenzhou Xinhao | Sow farm |
| Shenze Xinhao | Sow farm | Guangyuan Xinhao | Sow farm | Tongliao Xinhao | Sow farm |
| Gansu Xinhao | Sow farm | Laiyang Xinhao | Sow farm | Shuozhou Xinhao | Sow farm |
| Kangping Xinwang | Sow farm | Hubei Xinhao | Sow farm | Hengnan Muyun | Sow farm |
| Yan'an Benyuan | Sow farm | Zhangwu Xinwang | Sow farm | Meishan Xinhai | Sow farm |
| Liangshan Xin Liu | Sow farm | Liaoning Xintao Wang | Fattening farm | Zhangjiakou Xinwang | Sow farm |
| Liaoning Xin Taowang | Sow farm | Hainan Nongken | Sow farm | Caoxian Xinhao | Sow farm |
| Longzhou Xinhao | Fattening farm | Liangshan Xinliu | Sow farm | Jiangxi Xinliu | Sow farm |
| Hebei Xinhao | Sow farm | Shuozhou Xinhao | Sow farm | Ruzhou Quansheng | Sow farm |
| Taian Xin Liu | Sow farm | Liaocheng Xinhao | Sow farm | Gansu Xinhao | Sow farm |
| Gansu Xinhao | Sow farm | Hebei Xinhao | Sow farm | Laixi Xinliu | Sow farm |
| Guang'an Xinhao | Fattening farm | Heishan Xinliu | Sow farm | Laixi Xinliu | Sow farm |
| Guanglü Xin Liu | Sow farm | Laixi Xinliu | Sow farm | Taian Xinliu | Sow farm |
| Zhaoqing Xinhao | Fattening farm | Gansu Xinhao | Sow farm | Jian Xinchi | Sow farm |
| Tongliao Xinhao | Sow farm | Liaoning Xintao Wang | Sow farm | Nanchong Xinhao | Fattening farm |
| Xinji Xin Liu | Sow farm | Yan'an Benyuan | Sow farm | Tongliao Xinhao | Sow farm |
| Longhui Xin Hai | Fattening farm | Wannian Xinhai | Fattening farm | Laixi Xinliu | Sow farm |
| Liaoning Xin Taowang | Sow farm | Guigang Xinliu | Fattening farm | Tangshan Xinhao | Sow farm |
| Ruyuan Xinhao | Sow farm | Taian Xinliu | Sow farm | Xiajin Nongmu | Sow farm |
| Gaomi Xin Liu | Sow farm | Laixi Xinliu | Sow farm | Luding Xinyue | Fattening farm |
| Pingfu Nongmu | Sow farm | Dong'e Xinliu | Sow farm | Weinan Xinliu | Sow farm |
| Shuozhou Xinhao | Sow farm | Gansu Xinhao | Sow farm | Tongliao Xinhao | Sow farm |
| Xiangyang Xinhao | Sow farm | Caoxian Xinhao | Sow farm | Tangshan Xinhao | Sow farm |
| Liaocheng Xinhao | Sow farm | Fuxin Xinwang | Sow farm | Gansu Xinliu | Sow farm |
| Laixi Xin Liu | Sow farm | Pingfu Nongmu | Sow farm | Kangping Xinwang | Sow farm |
| Xinji Xin Liu | Sow farm | Gansu Xinhao | Sow farm | Tongliao Xinhao | Sow farm |
| Juye Xinhao | Sow farm | Liaoning Xintao Wang | Sow farm | Liaoning Xintao Wang | Sow farm |
| Zhuocheng Xin Liu | Sow farm | Qinghua Nongmu | Sow farm | Yingcheng Xinhao | Sow farm |
| Caoxian Xinhao | Sow farm | Liaoning Xinwang | Sow farm | Dezhou Xinhao | Sow farm |
| Ruzhou Quansheng | Sow farm | Zhangwu Xinwang | Fattening farm | Dezhou Xinhao | Sow farm |
| Changyi Xinhao | Sow farm | Gansu Xinhao | Sow farm | Laiyang Xinmu | Sow farm |
| Shuozhou Xinhao | Sow farm | Kangping Xinwang | Sow farm | Tangshan Xinhao | Sow farm |
| Laixi Xin Liu | Sow farm | Dezhou Xinhao | Sow farm | Hebei Xinhao | Sow farm |
| Qinghua Nongmu | Sow farm | Zhangwu Xinwang | Sow farm | Zhongshan Nongmu | Sow farm |
| Tangshan Xinhao | Sow farm | Zhangjiakou Xinwang | Sow farm | Xuanwei Xinliu | Sow farm |
| Gaomi Xin Liu | Sow farm | Gaotang Xinhao | Sow farm | Liaocheng Xinhao | Sow farm |
| Liaocheng Xinhao | Sow farm | Zhu Cheng Xinliu | Sow farm | Nantong Xinhai | Sow farm |
| Yichun Xinwang | Fattening farm | Xiajin Nongmu | Sow farm | Longhui Xinhai | Sow farm |
| Dong'e Xin Liu | Sow farm | Liaocheng Xinhao | Sow farm | Pucheng Xinliu | Sow farm |
| Juye Xinhao | Sow farm | Xiangshui Xincheng | Fattening farm | Xinji Xinliu | Sow farm |
| Tongliao Xinhao | Sow farm | Binzhou Xinhai | Sow farm | Dezhou Xinhao | Sow farm |
| Changyi Xinhao | Sow farm | Tangshan Xinhao | Sow farm | Yichun Xinwang | Sow farm |
| Zhangwu Xinwang | Sow farm | Ruzhou Quansheng | Sow farm | Kangping Xinwang | Sow farm |
| Pengshui Xin Liu | Fattening farm | Binzhou Xinhai | Sow farm | Juye Xinhao | Sow farm |
| Shenze Xinhao | Sow farm | Luding Xinyue | Fattening farm | Hainan Nongken | Sow farm |
| Tongliao Xinhao | Sow farm | Hainan Nongken | Sow farm | Tongliao Xinhao | Sow farm |
| Shenze Xinhao | Sow farm | Nantong Xinhai | Sow farm | Juye Xinliu | Sow farm |
| Luding Xinyue | Fattening farm | Ruzhou Quansheng | Sow farm | Jiangyou Zhuchang | Sow farm |
| Yingcheng Xinhao | Sow farm | Ruyuan Xinhao | Sow farm | Xiangshui Xincheng | Fattening farm |
| Zhangjiakou Xinwang | Sow farm | Juye Xinhao | Sow farm | Tongliao Xinhao | Sow farm |
| Chenzhou Xinhao | Sow farm | Changyi Xinhao | Sow farm | Zhu Cheng Xinliu | Sow farm |
| Liaoning Xinwang | Sow farm | Zhongshan Nongmu | Sow farm | Jie Xi Xinliu | Fattening farm |
| Zhongshan Nongmu | Sow farm | Dezhou Xinhao | Sow farm | Changyi Xinhao | Sow farm |
| Tongliao Xinhao | Sow farm | Tongliao Xinhao | Sow farm | Zhu Cheng Xinliu | Sow farm |
| Luocheng Xinhao | Fattening farm | Yingcheng Xinhao | Sow farm | Dong'e Xinliu | Sow farm |
| Tongliao Xinhao | Sow farm | Jiangyou Pig Farm | Sow farm | Heishan Xinliu | Sow farm |
| Jiangyou Zhuchang | Sow farm | Juye Xinhao | Sow farm | Liaoning Xintao Wang | Sow farm |
| Wuming Xin Liu | Fattening farm | Tongliao Xinhao | Sow farm | Meishan Xinhai | Fattening farm |
| Ruzhou Quansheng | Sow farm | Tongliao Xinhao | Sow farm | Chongren Xinhai | Fattening farm |
| Tongliao Xinhao | Sow farm | Laixi Xinliu | Sow farm | Yucheng Xinliu | Sow farm |
| Juye Xinhao | Sow farm | Jian Xinchi | Sow farm | Yingtan Xinliu | Sow farm |
| Gansu Xinhao | Sow farm | Gansu Xinhao | Sow farm | Tongliao Xinhao | Sow farm |
| Ruzhou Quansheng | Sow farm | Gaomi Xinliu | Sow farm | Gaomi Xinliu | Sow farm |
| Liaoning Xin Taowang | Sow farm | Shuozhou Xinhao | Sow farm | Laixi Xinliu | Sow farm |
| Dezhou Xinhao | Sow farm | Tangshan Xinhao | Sow farm | Gaomi Xinliu | Sow farm |
| Nanchong Xinhao | Sow farm | Liaoning Xintao Wang | Sow farm | Liaoning Xintao Wang | Sow farm |
| Xinji Xin Liu | Sow farm | Chenzhou Xinhao | Sow farm | Ruzhou Quansheng | Sow farm |
| Guangyuan Xinhao | Sow farm | Gaomi Xinliu | Sow farm | Xiajin Nongmu | Sow farm |
| Gansu Xinhao | Sow farm | Hebei Xinhao | Sow farm | Chenzhou Xinhao | Sow farm |
| Heishan Xin Liu | Sow farm | Longzhou Xinhao | Fattening farm | Juye Xinliu | Sow farm |
| Hubei Xinhao | Sow farm | Zhangwu Xinwang | Fattening farm | Gaomi Xinliu | Sow farm |
| Zhaoqing Xinhao | Sow farm | Xuanwei Xinliu | Sow farm | Gansu Xinhao | Sow farm |
| Dezhou Xinhao | Sow farm | Longhui Xinhai | Fattening farm | Gansu Xinhao | Sow farm |
| Binzhou Xin Hai | Sow farm | Hubei Xinhao | Sow farm | Guigang Xinliu | Fattening farm |
| Zhangwu Xinwang | Sow farm | Yucheng Xinliu | Sow farm | Lingxin Xinliu | Sow farm |
| Liaoning Xin Taowang | Sow farm | Liaoning Xintao Wang | Sow farm | Hubei Xinhao | Sow farm |
| Hubei Xinhao | Sow farm | Gaomi Xinliu | Sow farm | Zhangjiakou Xinwang | Sow farm |
| Chenzhou Xinhao | Fattening farm | Heishan Xinliu | Sow farm | Shuozhou Xinhao | Sow farm |
| Tongliao Xinhao | Sow farm | Hubei Xinhao | Sow farm | Heishan Xinliu | Sow farm |
| Hebei Xinhao | Sow farm | Tangshan Xinhao | Sow farm | Gansu Xinliu | Sow farm |
| Xuanwei Xin Liu | Sow farm | Langzhong Xinliu | Fattening farm | Hubei Xinhao | Sow farm |
| Heishan Xin Liu | Sow farm | Chenzhou Xinhao | Sow farm | Xiajin Nongmu | Sow farm |
| Shuozhou Xinhao | Sow farm | Tangshan Xinhao | Sow farm | Fuxin Xinwang | Sow farm |
| Zhangjiakou Xinwang | Sow farm | Juye Xinhao | Sow farm | Kangping Xinwang | Sow farm |
| Tongliao Xinhao | Sow farm | Tongliao Xinhao | Sow farm | Qingyuan Xinhao | Fattening farm |
| Tangshan Xinhao | Sow farm | Luocheng Xinhao | Fattening farm | Zhangjiakou Xinwang | Sow farm |
| Anyue Xing Xin Xin | Sow farm | Gansu Xinhao | Sow farm | Nanning Xinhao | Sow farm |
| Gansu Xinhao | Sow farm | Binzhou Xinhai | Sow farm | Ruyuan Xinhao | Sow farm |
| Yucheng Xin Liu | Sow farm | Ruzhou Quansheng | Sow farm | Yichun Xinwang | Fattening farm |
| Heishan Xin Liu | Sow farm | Kangping Xinwang | Sow farm | Zhangjiakou Xinwang | Sow farm |
| Tongliao Xinhao | Sow farm | Chongren Xinhai | Fattening farm | Ruzhou Quansheng | Sow farm |
| Jian Xinchi | Sow farm | Laiyang Xinmu | Sow farm | Gansu Xinhao | Sow farm |
| Juye Xinhao | Sow farm | Heishan Xinliu | Sow farm | Kangping Xinwang | Sow farm |
| Tangshan Xinhao | Sow farm | Lingxin Xinliu | Sow farm | Heyang Xinliu | Sow farm |
| Gansu Xinhao | Sow farm | Santai Nongmu | Sow farm | Laixi Xinliu | Sow farm |
| Kangping Xinwang | Sow farm | Tangshan Xinhao | Sow farm | Hubei Xinhao | Sow farm |
| Yingcheng Xinhao | Sow farm | Nanning Xinhao | Fattening farm | Liaoning Xinwang | Sow farm |
| Ruyuan Xinhao | Sow farm | Gansu Xinhao | Sow farm | Shuozhou Xinhao | Sow farm |
| Yichun Xinwang | Sow farm | Hebei Xinhao | Sow farm | Gansu Xinhao | Sow farm |
| Tangshan Xinhao | Sow farm | Zhangjiakou New Hope | Sow farm | Caoxian Xinhao | Sow farm |
| Luocheng Xinhao | Fattening farm | Zhangwu New Hope | Fattening farm | Tangshan Xinhao | Sow farm |
| Dezhou Xinhao | Sow farm | Anyue Xinxin | Sow farm | Dezhou Xinhao | Sow farm |
| Shuozhou Xinhao | Sow farm | Zhenjiang Xinhai | Fattening farm | Xiajin Nongmu | Sow farm |
| Nanning Xinhao | Sow farm | Zhangjiakou New Hope | Sow farm | Laixi Xinliu | Sow farm |
| Zhangjiakou New Hope | Sow farm | Liaoning New Hope | Sow farm | Hainan Nongken | Sow farm |
| Liaoning New Hope | Sow farm | Yichun New Hope | Fattening farm | Anyue Xinxinxin | Fattening farm |
| Santai Agriculture and Animal Husbandry | Sow farm | Dezhou Xinhao | Sow farm | Guangyuan Xinhao | Sow farm |
| Hezhou Xinhao | Sow farm | Gansu Xinhao | Sow farm | Xiajin Nongmu | Sow farm |
| Tangshan Xinhao | Sow farm | Guangan Xinhao | Fattening farm | Liaoning Xinwang | Sow farm |
| Laixi Xinliu | Sow farm | Guangyuan Xinhao | Sow farm | Dong'e Xinliu | Sow farm |
| Gaotang Xinhao | Sow farm | Zhangjiakou New Hope | Sow farm | Weihui Xinchi | Sow farm |
| Taian Xinliu | Sow farm | Longzhou Xinhao | Sow farm | Zhuocheng Xinliu | Sow farm |
| Heishan Xinliu | Sow farm | Caoxian Xinhao | Sow farm | Heishan Xinliu | Sow farm |
| Liaoning New Hope | Sow farm | Dezhou Xinhao | Sow farm | Tianxin Xinliu | Fattening farm |
| Tongliao Xinhao | Sow farm | Xiajin Agriculture and Animal Husbandry | Sow farm | Pucheng Xinliu | Sow farm |
| Zhangjiakou New Hope | Sow farm | Hengnan Muyun | Sow farm | Xiangyang Xinhao | Sow farm |
| Nanning Xinhao | Sow farm | Guigang Xinliu | Sow farm | Yingcheng Xinhao | Sow farm |
| Laiyang Xinmu | Sow farm | Kangping New Hope | Sow farm | Anhui Xinliu | Sow farm |
| Nanning Xinhao | Sow farm | Tangshan Xinhao | Sow farm | Meishan Xinhai | Sow farm |
| Sichuan Xinhao | Sow farm | Tangshan Xinhao | Sow farm | Liaoning Xinwang | Sow farm |
| Tongliao Xinhao | Sow farm | Anhui Xinliu | Sow farm | Zhejiang Xinhai | Sow farm |
| Dezhou Xinhao | Sow farm | Santai Agriculture and Animal Husbandry | Fattening farm | Nanning Xinliu | Sow farm |
| Xianyang Yongxiang | Sow farm | Ruzhou Quansheng | Sow farm | Santai Nongmu | Fattening farm |
| Laixi Xinliu | Sow farm | Dong'e Xinliu | Sow farm | Meishan Xinhai | Sow farm |
| Yingcheng Xinhao | Sow farm | Zhejiang Xinhai | Sow farm | Santai Nongmu | Sow farm |
| Kangping New Hope | Sow farm | Shuozhou Xinhao | Sow farm | Yucheng Xinliu | Sow farm |
| Gansu Xinhao | Sow farm | Tangshan Xinhao | Sow farm | Liaoning Xinwang | Sow farm |
| Leshan Agriculture and Animal Husbandry | Sow farm | Xiajin Agriculture and Animal Husbandry | Sow farm | Gaotang Xinhao | Sow farm |
| Anhui Xinliu | Sow farm | Tangshan Xinhao | Sow farm | Liaoning Xinwang | Sow farm |
| Tangshan Xinhao | Sow farm | Zhu Cheng Xinliu | Sow farm | Ruyuan Xinhao | Sow farm |
| Taian Xinliu | Sow farm | Liaoning New Hope | Sow farm | Binzhou Xinhai | Sow farm |
| Zhejiang Xinhai | Sow farm | Hubei Xinhao | Sow farm | Liaoning Xinwang | Sow farm |
| Tangshan Xinhao | Sow farm | Tangshan Xinhao | Sow farm | Guangyuan Xinhao | Sow farm |
| Tangshan Xinhao | Sow farm | Shuozhou Xinhao | Sow farm | Binzhou Xinhai | Sow farm |
| Caoxian Xinhao | Sow farm | Hubei Xinhao | Sow farm | Shuozhou Xinhao | Sow farm |
| Weinan Xinliu | Sow farm | Weinan Xinliu | Fattening farm | Baiyin Nongmu | Sow farm |
| Meishan Xinhai | Fattening farm | Yingcheng Xinhao | Sow farm | Langzhong Xinliu | Fattening farm |
| Nanning Xinhao | Fattening farm | Shuozhou Xinhao | Sow farm | Baiyin Nongmu | Sow farm |
| Dong'e Xinliu | Sow farm | Chenzhou Xinhao | Fattening farm | Weinan Xinliu | Sow farm |
| Hubei Xinhao | Sow farm | Liaoning New Hope | Sow farm | Hubei Xinhao | Sow farm |
| Zhuocheng Xinliu | Sow farm | Qingyuan Xinhao | Fattening farm | Nanchong Xinhao | Sow farm |
| Tangshan Xinhao | Sow farm | Taipeng Xinliu | Sow farm | Kangping Xinwang | Sow farm |
| Dezhou Xinhao | Sow farm | Baiyin Agriculture and Animal Husbandry | Sow farm | Yingcheng Xinhao | Sow farm |
| Tangshan Xinhao | Sow farm | Baiyin Agriculture and Animal Husbandry | Sow farm | Hengnan Muyun | Sow farm |
| Hubei Xinhao | Sow farm | Xiajin Agriculture and Animal Husbandry | Sow farm | Hubei Xinhao | Sow farm |
| Weinan Xinliu | Sow farm | Taipeng Xinliu | Sow farm | Taian Xinliu | Sow farm |
| Guanling Xinhai | Sow farm | Liaoning New Hope | Sow farm | Guigang Xinliu | Sow farm |
| Hubei Xinhao | Sow farm | Weinan Xinliu | Sow farm | Wuhe Xinhai | Sow farm |
| Shibing Xinliu | Sow farm | Hubei Xinhao | Sow farm | Shuozhou Xinhao | Sow farm |
| Hebei Xinhao | Sow farm | Hengshui Agriculture and Animal Husbandry | Sow farm | Wuhe Xinhai | Sow farm |
| Longzhou Xinhao | Sow farm | Dezhou Xinhao | Sow farm | Gansu Xinhao | Sow farm |
| Liaoning New Hope | Sow farm | Kangping New Hope | Sow farm | Tangshan Xinhao | Sow farm |
| Wuhe Xinhai | Sow farm | Nanning Xinhao | Sow farm | Yingcheng Xinhao | Sow farm |
| Gansu Xinhao | Sow farm | Wuhe Xinhai | Sow farm | Gansu Xinhao | Sow farm |
| Taian Xinliu | Sow farm | Heyang Xinliu | Sow farm | Liaoning Xinwang | Sow farm |
| Baiyin Agriculture and Animal Husbandry | Sow farm | Laibin Xinhao | Sow farm | Tangshan Xinhao | Sow farm |
| Nanning Xinhao | Sow farm | Gansu Xinhao | Sow farm | Wuqi Xinliu | Sow farm |
| Xiajin Agriculture and Animal Husbandry | Sow farm | Liaoning New Hope | Sow farm | Chenzhou Xinhao | Sow farm |
| Xiajin Agriculture and Animal Husbandry | Sow farm | Gansu Xinhao | Sow farm | Laibin Xinhao | Sow farm |
| Wuhe Xinhai | Sow farm | Yucheng Xinliu | Sow farm | Tangshan Xinhao | Sow farm |
| Taian Xinliu | Sow farm | Liaoning New Hope | Sow farm | Nanning Xinhao | Sow farm |
| Nanning Xinliu | Sow farm | Xingren Xinliu | Sow farm | Chenzhou Xinhao | Sow farm |
| Shuozhou Xinhao | Sow farm | Wuhe Xinhai | Sow farm | Nanchong Xinhao | Sow farm |
| Heyang Xinliu | Sow farm | Nanning Xinhao | Sow farm | Nanning Xinhao | Sow farm |
| Xiangzhou Xinhao | Sow farm | Tangshan Xinhao | Sow farm | Jiaxiang Xinliu | Sow farm |
| Tangshan Xinhao | Sow farm | Hezhou Xinhao | Sow farm | Tangshan Xinhao | Sow farm |
| Yucheng Xinliu | Sow farm | Nanning Xinhao | Sow farm | Hengnan Muyun | Sow farm |
| Liaoning New Hope | Sow farm | Kangping New Hope | Sow farm | Liaoning Xinwang | Sow farm |
| Guang'an Xinhao | Fattening farm | Tangshan Xinhao | Sow farm | Guangan Xinhao | Fattening farm |
| Liaoning New Hope | Sow farm | Laixi Xinliu | Sow farm | Kangping Xinwang | Sow farm |
| Zhejiang Xinhai | Sow farm | Heishan Xinliu | Sow farm | Yingcheng Xinhao | Sow farm |
| Laibin Xinhao | Sow farm | Yingcheng Xinhao | Sow farm | Ningming Xinhao | Sow farm |
| Heishan Xinliu | Sow farm | Tianxin Xinliu | Fattening farm | Laiyang Xinhao | Sow farm |
| Lingxin Xinliu | Sow farm | Dong'e Xinliu | Sow farm | Baiyin Nongmu | Sow farm |
| Baiyin Agriculture and Animal Husbandry | Sow farm | Pengshui Xinliu | Fattening farm | Tangshan Xinhao | Sow farm |
| Tianxin Xinliu | Fattening farm | Taipeng Xinliu | Sow farm | Hengnan Muyun | Sow farm |
| Caoxian Xinhao | Sow farm | Xiajin Agriculture and Animal Husbandry | Sow farm | Weinan Xinliu | Fattening farm |
| Huanghua Xinhao | Sow farm | Laixi Xinliu | Sow farm | Hezhou Xinhao | Sow farm |
| Anyue Xinxin | Fattening farm | Nanchong Xinhao | Sow farm | Zhejiang Xinhai | Sow farm |
| Kangping New Hope | Sow farm | Zhejiang Xinhai | Sow farm | Yingcheng Xinhao | Sow farm |
| Wuhe Xinhai | Sow farm | Leshan Agriculture and Animal Husbandry | Sow farm | Taian Xinliu | Sow farm |
| Tangshan Xinhao | Sow farm | Heishan Xinliu | Sow farm | Hubei Xinhao | Sow farm |
| Shuozhou Xinhao | Sow farm | Taipeng Xinliu | Sow farm | Guigang Xinliu | Sow farm |
| Xiangzhou Xinhao | Sow farm | Kangping New Hope | Sow farm | Beijing Xinliu | Sow farm |
| Gansu Xinhao | Sow farm | Yingcheng Xinhao | Sow farm | Xingren Xinliu | Sow farm |
| Nanning Xinhao | Sow farm | Shuozhou Xinhao | Sow farm | Dong'e Xinliu | Sow farm |
| Hubei Xinhao | Sow farm | Guanling Xinhai | Sow farm | Heishan Xinliu | Sow farm |
| Kangping New Hope | Sow farm | Anyue Xinxin | Fattening farm | Dong'e Xinliu | Sow farm |
| Hengnan Muyun | Sow farm | Jiaxiang Xinliu | Sow farm | Hezhou Xinhao | Sow farm |
| Nanchong Xinhao | Sow farm | Xiangzhou Xinhao | Sow farm | Leshan Nongmu | Sow farm |
| Baiyin Agriculture and Animal Husbandry | Sow farm | Laiyang Xinhao | Sow farm | Tongliao Xinhao | Sow farm |
| Ningming Xinhao | Sow farm | Hengnan Muyun | Sow farm | Xiangyang Xinhao | Sow farm |
| Jiaxiang Xinliu | Sow farm | Ningming Xinhao | Sow farm | Xiangzhou Xinhao | Sow farm |
| Dezhou Xinhao | Sow farm | Xiangzhou Xinhao | Sow farm | Guangdong Xinhao | Sow farm |
| Liaoning New Hope | Sow farm | Baiyin Agriculture and Animal Husbandry | Sow farm | Guanling Xinhai | Sow farm |
| Baiyin Agriculture and Animal Husbandry | Sow farm | Liaoning New Hope | Sow farm | Baiyin Nongmu | Sow farm |
| Hengnan Muyun | Sow farm | Baiyin Agriculture and Animal Husbandry | Sow farm | Dong'e Xinliu | Sow farm |
| Laixi Xinliu | Sow farm | Hengnan Muyun | Sow farm | Dong'e Xinliu | Sow farm |
| Hezhou Xinhao | Sow farm | Dong'e Xinliu | Sow farm | Jiaxiang Xinliu | Sow farm |
| Baiyin Agriculture and Animal Husbandry | Sow farm | Yingcheng Xinhao | Sow farm | Shuozhou Xinhao | Sow farm |
| Hezhou Xinhao | Sow farm | Guigang Xinliu | Sow farm | Nanning Xinhao | Sow farm |
| Kangping New Hope | Sow farm | Baiyin Agriculture and Animal Husbandry | Sow farm | Caoxian Xinhao | Sow farm |
| Xiajin Agriculture and Animal Husbandry | Sow farm | Baiyin Agriculture and Animal Husbandry | Sow farm | Baiyin Nongmu | Sow farm |
| Zhenjiang Xinhai | Fattening farm | Caoxian Xinhao | Sow farm | Hezhou Xinhao | Sow farm |
| Baiyin Agriculture and Animal Husbandry | Sow farm | Wuqi Xinliu | Sow farm | Nanning Xinhao | Sow farm |
| Shuozhou Xinhao | Sow farm | Hezhou Xinhao | Sow farm | Xiangzhou Xinhao | Sow farm |
| Xiajin Agriculture and Animal Husbandry | Sow farm | Ruyuan Xinhao | Sow farm | Guigang Xinliu | Fattening farm |
| Yingcheng Xinhao | Sow farm | Shibing Xinliu | Sow farm | Chenzhou Xinhao | Fattening farm |
| Dong'e Xinliu | Sow farm | Nanning Xinhao | Sow farm | Caoxian Xinhao | Sow farm |
| Huanghua Xinhao | Sow farm | Hezhou Xinhao | Sow farm | Shibing Xinliu | Sow farm |
| Hezhou Xinhao | Sow farm | Jiaxiang Xinliu | Sow farm | Hezhou Xinhao | Sow farm |
| Liaoning New Hope | Sow farm | Hezhou Xinhao | Sow farm | Guangdong Xinhao | Sow farm |
| Guigang Xinliu | Sow farm | Shuozhou Xinhao | Sow farm | Hezhou Xinhao | Sow farm |
| Yingcheng Xinhao | Sow farm | Nanning Xinliu | Sow farm | Weinan Xinliu | Sow farm |
| Wuhe Xinhai | Sow farm | Guangdong Xinhao | Sow farm | Bijie Xinliu | Sow farm |
| Zhaoqing Xinhao | Sow farm | Dong'e Xinliu | Sow farm | Guangdong Xinhao | Sow farm |
| Yichun New Hope | Sow farm | Laiyang Xinmu | Sow farm | Yingcheng Xinhao | Sow farm |
| Heyang Xinliu | Sow farm | Liaoning New Hope | Sow farm | Shuozhou Xinhao | Sow farm |
| Zhaoqing Xinhao | Sow farm | Yingcheng Xinhao | Sow farm | Guangdong Xinhao | Sow farm |
| Zhongshan Agriculture and Animal Husbandry | Sow farm | Hezhou Xinhao | Sow farm | Baiyin Nongmu | Sow farm |
| Hezhou Xinhao | Sow farm | Heyang Xinliu | Sow farm | Weinan Xinliu | Sow farm |
| Shuozhou Xinhao | Sow farm | Xingren Xin Liu | Sow farm | Ningming Xinhao | Sow farm |
| Shibing Xinliu | Sow farm | Nanning Xinhao | Sow farm | Dong'a Xinliu | Sow farm |
| Yingcheng Xinhao | Sow farm | Zhaoqing Xinhao | Sow farm | Ningming Xinhao | Sow farm |
| Dong'e Xinliu | Sow farm | Wuhe Xin Hai | Sow farm | Nanning Xinliu | Sow farm |
| Ningming Xinhao | Sow farm | Caoxian Xinhao | Sow farm | Rugao Xinhao | Sow farm |
| Guangdong Xinhao | Sow farm | Gansu Xinhao | Sow farm | Laiyang Xinmu | Sow farm |
| Bijie Xinliu | Sow farm | Dong'e Xin Liu | Sow farm | Ningming Xinhao | Sow farm |
| Hengshui Agriculture and Animal Husbandry | Sow farm | Guangdong Xinhao | Sow farm | Laibin Xinhao | Sow farm |
| Jiaxiang Xinliu | Sow farm | Ningming Xinhao | Sow farm | Taian Xinliu | Sow farm |
| Ruzhou Quansheng | Sow farm | Weinan Xin Liu | Sow farm | Huaiyin New Run | Sow farm |
| Caoxian Xinhao | Sow farm | Shuozhou Xinhao | Sow farm | Juye Xinhao | Sow farm |
| Bijie Xinliu | Sow farm | Nanning Xin Liu | Sow farm | Shuozhou Xinhao | Sow farm |
| Weinan Xinliu | Sow farm | Ningming Xinhao | Sow farm | Xingren Xinliu | Sow farm |
| Gansu Xinhao | Sow farm | Xiangyang Xinhao | Sow farm | Yingtan Xinliu | Sow farm |
| Wuqi Xinliu | Sow farm | Gaotang Xinhao | Sow farm | Juye Xinhao | Sow farm |
| Weinan Xinliu | Sow farm | Yichun Xin Wang | Sow farm | Weinan Xinliu | Sow farm |
| Dong'e Xinliu | Sow farm | Laiyang Xinhao | Sow farm | Laibin Xinhao | Sow farm |
| Laiyang New Agriculture | Sow farm | Guangdong Xinhao | Sow farm | Kangping Xinwang | Sow farm |
| Liaoning Xinwang | Sow farm | Bijie Xin Liu | Sow farm | Longzhou Xinhao | Sow farm |
| Guangdong Xinhao | Sow farm | Wuhe Xin Hai | Sow farm | Nanning Xinliu | Sow farm |
| Ningming Xinhao | Sow farm | Kangping Xin Wang | Sow farm | Wuhe Xinhai | Sow farm |
| Yancheng Zhongtai | Sow farm | Laibin Xinhao | Sow farm | Ningming Xinhao | Sow farm |
| Ningming Xinhao | Sow farm | Weinan Xin Liu | Sow farm | Shibing Xinliu | Sow farm |
| Laiyang Xinhao | Sow farm | Guangdong Xinhao | Sow farm | Taian Xinliu | Sow farm |
| Dong'e Xinliu | Sow farm | Ningming Xinhao | Sow farm | Guangdong Xinhao | Sow farm |
| Gaotang Xinhao | Sow farm | Shibing Xin Liu | Sow farm | Liaoning Xinwang | Sow farm |
| Guangdong Xinhao | Sow farm | Xiangzhou Xinhao | Sow farm | Hainan Xinliu | Sow farm |
| Ningming Xinhao | Sow farm | Dong'e Xin Liu | Sow farm | Yichun Xinwang | Sow farm |
| Weinan Xinliu | Sow farm | Laibin Xinhao | Sow farm | Bijie Xinliu | Sow farm |
| Guangdong Xinhao | Sow farm | Guangdong Xinhao | Sow farm | Wuhe Xinhai | Sow farm |
| Shibing Xinliu | Sow farm | Juye Xinhao | Sow farm | Zhangwu Xinwang | Sow farm |
| Juye Xinhao | Sow farm | Bijie Xin Liu | Sow farm | Liaoning Xinwang | Sow farm |
| Kangping Xinwang | Sow farm | Kangping Xin Wang | Sow farm | Guangdong Xinhao | Sow farm |
| Laibin Xinhao | Sow farm | Juye Xinhao | Sow farm | Laibin Xinhao | Sow farm |
| Nanning Xinliu | Sow farm | Liaoning Xin Wang | Sow farm | Xiangzhou Xinhao | Sow farm |
| Santai Agriculture and Animal Husbandry | Sow farm | Zhangwu Xin Wang | Sow farm | Liaoning Xinwang | Sow farm |
| Hubei Xinhao | Sow farm | Yichun Xin Wang | Sow farm | Hainan Xinliu | Sow farm |
| Guangdong Xinhao | Sow farm | Liaoning Xin Wang | Sow farm | Zhaoqing Xinhao | Sow farm |
| Xiangzhou Xinhao | Sow farm | Hubei Xinhao | Sow farm | Laixi Xinliu | Sow farm |
| Ningming Xinhao | Sow farm | Ningming Xinhao | Sow farm | Linhai Xinliu | Sow farm |
| Laiyang Xinhao | Sow farm | Nanchong Xinhao | Sow farm | Laibin Xinhao | Sow farm |
| Liaoning Xinwang | Sow farm | Hainan Xin Liu | Sow farm | Anyue Xing Xin Xin | Sow farm |
| Shibing Xinliu | Sow farm | Yijun Xin Liu | Sow farm | Ningming Xinhao | Sow farm |
| Xiangzhou Xinhao | Sow farm | Yijun Xin Liu | Sow farm | Yijun Xinliu | Sow farm |
| Longhui Xinhai | Sow farm | Laibin Xinhao | Sow farm | Liaoning Xinwang | Sow farm |
| Yijun Xinliu | Sow farm | Kangping Xin Wang | Sow farm | Liaoning Xinwang | Sow farm |
| Liaoning Xinwang | Sow farm | Ningming Xinhao | Sow farm | Kangping Xinwang | Sow farm |
| Hezhou Xinhao | Sow farm | Laixi Xin Liu | Sow farm | Laibin Xinhao | Sow farm |
| Laibin Xinhao | Sow farm | Santai Nongmu | Sow farm | Yijun Xinliu | Sow farm |
| Shibing Xinliu | Sow farm | Hubei Xinhao | Sow farm | Laiyang Xinmu | Sow farm |
| Yijun Xinliu | Sow farm | Huaiyin Xin Chi | Sow farm | Pengshui Xinliu | Sow farm |
| Hainan Xinliu | Sow farm | Weinan Xin Liu | Sow farm | Qinghua Agriculture and Animal Husbandry | Sow farm |
| Laixi Xinliu | Sow farm | Shibing Xin Liu | Sow farm | Hubei Xinhao | Sow farm |
| Juye Xinhao | Sow farm | Laibin Xinhao | Sow farm | Laiyang Xinhao | Sow farm |
| Kangping Xinwang | Sow farm | Shibing Xin Liu | Sow farm | Laibin Xinhao | Sow farm |
| Kangping Xinwang | Sow farm | Shibing Xin Liu | Sow farm | Kangping Xinwang | Sow farm |
| Zhaoqing Xinhao | Sow farm | Lingao Xin Liu | Sow farm | Yijun Xinliu | Sow farm |
| Dong'e Xinliu | Sow farm | Laibin Xinhao | Sow farm | Shibing Xinliu | Sow farm |
| Yijun Xinliu | Sow farm | Laiyang Xin Mu | Sow farm | Santai Agriculture and Animal Husbandry | Sow farm |
| Liaoning Xinwang | Sow farm | Yijun Xin Liu | Sow farm | Shibing Xinliu | Sow farm |
| Huairou Agriculture and Animal Husbandry | Sow farm | Liaoning Xin Wang | Sow farm | Linhai Xinliu | Sow farm |
| Kangping Xinwang | Sow farm | Nanning Xin Liu | Sow farm | Shibing Xinliu | Sow farm |
| Lingao Xinliu | Sow farm | Qingyuan Xinhao | Sow farm | Laibin Xinhao | Sow farm |
| Laibin Xinhao | Sow farm | Kangping Xin Wang | Sow farm | Heyang Xinliu | Sow farm |
| Laibin Xinhao | Sow farm | Liaoning Xin Wang | Sow farm | Xiangzhou Xinhao | Sow farm |
|  |  | Laibin Xinhao | Sow farm | Leshan Agriculture and Animal Husbandry | Sow farm |
|  |  | Rugao Xinhao | Sow farm | Xiangzhou Xinhao | Sow farm |
|  |  | Liaoning Xin Wang | Sow farm | Yichun Xinwang | Sow farm |
|  |  | Longhui Xin Hai | Sow farm | Liaoning Xinwang | Sow farm |
|  |  | Changge Xin Chi | Sow farm | Hezhou Xinhao | Sow farm |
|  |  | Zhaoqing Xinhao | Sow farm | Guangan Xinhao | Sow farm |
|  |  | Hubei Xinhao | Sow farm | Hubei Xinhao | Sow farm |
|  |  | Lingao Xin Liu | Sow farm | Pingfu Agriculture and Animal Husbandry | Sow farm |
|  |  | Laibin Xinhao | Sow farm | Yan'an Original Source | Sow farm |
|  |  | Xiangzhou Xinhao | Sow farm | Kangping Xinwang | Sow farm |
|  |  | Hainan Xin Liu | Sow farm | Zhangwu Xinwang | Sow farm |
|  |  | Yancheng Zhongtai | Sow farm | Pingfu Agriculture and Animal Husbandry | Sow farm |
|  |  | Xiangzhou Xinhao | Sow farm | Wuqi Xinliu | Sow farm |
|  |  | Sichuan Xinhao | Sow farm | Hubei Xinhao | Sow farm |
|  |  |  |  | Yan'an Original Source | Sow farm |
|  |  |  |  | Pingfu Agriculture and Animal Husbandry | Sow farm |
|  |  |  |  | Zhaoqing Xinhao | Sow farm |
|  |  |  |  | Weinan Xinliu | Sow farm |
|  |  |  |  | Pingfu Agriculture and Animal Husbandry | Sow farm |
|  |  |  |  | Baiyin Agriculture and Animal Husbandry | Sow farm |
| **Jul 2021** | | **Aug 2021** | | **Sep 2021** | |
| **Company (Co., Ltd.)** | **Type of pig farm** | **Company (Co., Ltd.)** | **Type of pig farm** | **Company (Co., Ltd.)** | **Type of pig farm** |
| Nanning Xinhao | Fattening Farm | Guigang Xinliu | Fattening Farm | Laixi Xinliu | Sow farm |
| Laixi Xinliu | Sow farm | Suining Xinliu | Fattening Farm | Suining Xinliu | Sow farm |
| Xianyang Yongxiang | Sow farm | Yingtan Xinliu | Fattening Farm | Tianjin Agriculture and Animal Husbandry | Sow farm |
| Changyi Xinhao | Fattening Farm | Qingfeng Xinliu | Sow farm | Zhangwu Xinwang | Sow farm |
| Tianjin Xinliu | Fattening Farm | Tangshan Xinhao | Sow farm | Gaotang Xinhao | Sow farm |
| Tangshan Xinhao | Sow farm | Lingbao Xinliu | Sow farm | Xiajin Agriculture and Animal Husbandry | Sow farm |
| Fucheng Xinhao | Sow farm | Tangshan Xinhao | Sow farm | Tianjin Xinliu | Fattening farm |
| Laizhou Xinhai | Fattening Farm | Anyang Xinliu | Sow farm | Tongliao Xinhao | Sow farm |
| Tangshan Xinhao | Sow farm | Fucheng Xinhao | Sow farm | Xiajin Agriculture and Animal Husbandry | Sow farm |
| Liaocheng Xinhao | Fattening Farm | Guangdong Xinhao | Sow farm | Xiajin Agriculture and Animal Husbandry | Sow farm |
| Wulian Xinhao | Sow farm | Rugao Xinhao | Fattening Farm | Zhangwu Xinwang | Sow farm |
| Fuxin Xinwang | Sow farm | Lingxin Xinliu | Fattening Farm | Gansu Xinhao | Sow farm |
| Anyang Xinliu | Sow farm | Raoyang Xinhao | Sow farm | Dong'e Xinliu | Fattening farm |
| Anyang Xinliu | Sow farm | Fuxin Xinwang | Sow farm | Xiajin Agriculture and Animal Husbandry | Sow farm |
| Guigang Xinliu | Sow farm | Gaotang Xinhao | Sow farm | Tongliao Xinhao | Sow farm |
| Shanxian Xinhao | Fattening Farm | Danxian Xinliu | Fattening Farm | Gansu Xinhao | Sow farm |
| Tangshan Xinhao | Sow farm | Jiangxi Xinliu | Sow farm | Xiajin Agriculture and Animal Husbandry | Sow farm |
| Yingtan Xinliu | Sow farm | Xiangshui Xinchi | Fattening Farm | Luocheng Xinhao | Sow farm |
| Dongying Xinhao | Fattening Farm | Huanghua Xinhao | Sow farm | Xiajin Agriculture and Animal Husbandry | Sow farm |
| Guangdong Xinhao | Sow farm | Baiyin Agriculture and Animal Husbandry | Sow farm | Nanning Xinhao | Sow farm |
| Laixi Xinliu | Sow farm | Guigang Xinliu | Fattening Farm | Xuanwei Xinliu | Sow farm |
| Shibing Xinliu | Sow farm | Binzhou Xinhai | Fattening Farm | Heishan Xinliu | Sow farm |
| Guizhou Xinliu | Sow farm | Tongliao Xinhao | Sow farm | Laixi Xinliu | Sow farm |
| Lezhi Xinhai | Fattening Farm | Xindongwan Xinliu | Sow farm | Xiajin Agriculture and Animal Husbandry | Sow farm |
| Guigang Xinliu | Sow farm | Huanghua Xinhao | Sow farm | Tongliao Xinhao | Fattening farm |
| Qingyuan Xinhao | Fattening Farm | Chenzhou Xinhao | Fattening Farm | Xiangshui Xincheng | Sow farm |
| Xiajin Nongmu | Sow farm | Liuzhou Xinhao | Fattening Farm | Xiangshui Xincheng | Sow farm |
| Ruzhou Quansheng | Fattening Farm | Raoyang Xinhao | Sow farm | Huanghua Xinhao | Sow farm |
| Laixi Xinliu | Sow farm | Heishan Xinliu | Sow farm | Binzhou Xinhai | Sow farm |
| Heishan Xinliu | Sow farm | Santai Agriculture and Animal Husbandry | Fattening Farm | Heishan Woniu | Sow farm |
| Heishan Xinliu | Sow farm | Nanning Xinhao | Sow farm | Huanghua Xinhao | Sow farm |
| Zhucheng Xinliu | Fattening Farm | Liaocheng Xinhao | Fattening Farm | Guangdong Xinhao | Sow farm |
| Jian Xinchi | Fattening Farm | Laiyang Xinmu | Sow farm | Lingxin Xinliu | Fattening farm |
| Laizhou Xinhai | Sow farm | Jian Xinchi | Sow farm | Rugao Xinhao | Fattening farm |
| Shanxian Xinliu | Sow farm | Tongliao Xinhao | Sow farm | Qingyuan Xinhao | Fattening farm |
| Xiajin Nongmu | Sow farm | Guigang Xinliu | Sow farm | Laibin Xinhao | Sow farm |
| Suining Xinliu | Sow farm | Laizhou Xinhai | Sow farm | Binzhou Xinhai | Sow farm |
| Tongliao Xinhao | Sow farm | Caoxian Xinhao | Fattening Farm | Zhangwu Xinwang | Sow farm |
| Liaoning Xinwang | Sow farm | Xingren Xinliu | Sow farm | Dongying Xinhao | Sow farm |
| Changle Xinhai | Fattening Farm | Tianjin Xinliu | Fattening Farm | Guang'an Xinhao | Sow farm |
| Xiajin Nongmu | Sow farm | Danxian Xinhao | Sow farm | Nanning Xinliu | Sow farm |
| Xiajin Nongmu | Sow farm | Lezhi Xinhai | Fattening Farm | Huanghua Xinhao | Sow farm |
| Shanxian Xinliu | Fattening Farm | Gaotang Xinhao | Fattening Farm | Guigang Xinliu | Sow farm |
| Dongying Xinhao | Sow farm | Zhangwu Xinwang | Sow farm | Tongliao Xinhao | Sow farm |
| Shenze Xinhao | Sow farm | Laibin Xinhao | Sow farm | Raoyang Xinhao | Sow farm |
| Shenze Xinhao | Sow farm | Xiajin Agriculture and Animal Husbandry | Sow farm | Weinan Xinliu | Fattening farm |
| Laixi Xinliu | Sow farm | Beijing Xinliu | Sow farm | Tongliao Xinhao | Sow farm |
| Zhongshan Nongmu | Sow farm | Heishan Xinliu | Sow farm | Heishan Woniu | Sow farm |
| Laiyang Xinmu | Sow farm | Zhangjiakou Xinwang | Sow farm | Zhucheng Xinliu | Fattening farm |
| Laixi Xinliu | Sow farm | Pengshui Xinliu | Fattening Farm | Xingren Xinliu | Sow farm |
| Xingren Xinliu | Sow farm | Xiajin Agriculture and Animal Husbandry | Sow farm | Nanning Xinhao | Sow farm |
| Gaotang Xinhao | Fattening Farm | Lingxin Xinliu | Fattening Farm | Zhucheng Xinliu | Sow farm |
| Shenze Xinhao | Sow farm | Zhangwu Xinwang | Sow farm | Jiangxi Xinliu | Sow farm |
| Shuozhou Xinhao | Sow farm | Laixi Xinliu | Sow farm | Zhangwu Xinwang | Sow farm |
| Zhangwu Xinwang | Sow farm | Tongliao Xinhao | Sow farm | Gaomi Xinliu | Fattening farm |
| Dongying Xinhao | Sow farm | Tongliao Xinhao | Sow farm | Zhangwu Xinwang | Sow farm |
| Zhangwu Xinwang | Sow farm | Hainan Agriculture and Forestry | Sow farm | Tongliao Xinhao | Sow farm |
| Huaiyin Xincheng | Sow farm | Pengshui Xinliu | Fattening Farm | Guigang Xinliu | Sow farm |
| Hainan Nongken | Sow farm | Fucheng Xinhao | Sow farm | Gaotang Xinhao | Fattening farm |
| Liaocheng Xinhao | Fattening Farm | Wuming Xinliu | Sow farm | Zhangjiakou Xinwang | Sow farm |
| Fuxin Xinwang | Sow farm | Henan Agriculture and Animal Husbandry | Fattening Farm | Taian Xinliu | Sow farm |
| Longhui Xinhai | Sow farm | Gansu Xinhao | Fattening Farm | Shangcai Xinliu | Sow farm |
| Kangping Xinwang | Sow farm | Dezhou Xinhao | Sow farm | Anyang Xinliu | Sow farm |
| Beijing Xinliu | Sow farm | Xindongwan Xinliu | Sow farm | Zhangwu Xinwang | Fattening farm |
| Lezhi Xinhai | Fattening Farm | Chongren Xinhai | Fattening Farm | Gaotang Xinhao | Sow farm |
| Raoyang Xinhao | Sow farm | Binzhou Xinhai | Sow farm | Heishan Xinliu | Sow farm |
| Jian Xinchi | Sow farm | Binzhou Xinhai | Sow farm | Lingxin Xinliu | Sow farm |
| Nanning Xinhao | Sow farm | Zhangwu Xinwang | Sow farm | Guang'an Xinhao | Sow farm |
| Xiangshui Xincheng | Fattening Farm | Suining Xinliu | Sow farm | Beijing Xinliu | Sow farm |
| Tangshan Xinhao | Sow farm | Laiyang Xinmu | Sow farm | Lingbao Xinliu | Sow farm |
| Shuyang Xinliu | Sow farm | Fuxin Xinwang | Sow farm | Xiajin Agriculture and Animal Husbandry | Sow farm |
| Heishan Xinliu | Sow farm | Dongying Xinhao | Sow farm | Jian Xinchi | Sow farm |
| Laibin Xinhao | Sow farm | Xiangshui Xinchi | Fattening Farm | Shenze Xinhao | Sow farm |
| Gaotang Xinhao | Sow farm | Taian Xinliu | Fattening Farm | Luding Xinyue | Sow farm |
| Dongying Xinhao | Sow farm | Hebei Xinhao | Sow farm | Xiajin Agriculture and Animal Husbandry | Sow farm |
| Laixi Xinliu | Sow farm | Neiqiu Xinliu | Fattening Farm | Xiajin Agriculture and Animal Husbandry | Sow farm |
| Tangshan Xinhao | Sow farm | Wuhe Xinhai | Sow farm | Xiajin Agriculture and Animal Husbandry | Sow farm |
| Zhangwu Xinwang | Sow farm | Wulian Xinhao | Sow farm | Tianjin Xinliu | Sow farm |
| Longhui Xinhai | Sow farm | Dongying Xinhao | Sow farm | Meishan Xinhai | Fattening farm |
| Tongliao Xinhao | Sow farm | Jian Xinchi | Sow farm | Anyang Xinliu | Sow farm |
| Tongliao Xinhao | Sow farm | Tianxin Xinliu | Sow farm | Guigang Xinliu | Sow farm |
| Hezhou Xinhao | Fattening Farm | Dongying Xinhao | Fattening Farm | Guanling Xinhai | Sow farm |
| Heishan Xinliu | Sow farm | Langzhong Xinliu | Sow farm | Heishan Xinliu | Sow farm |
| Tongliao Xinhao | Fattening Farm | Meishan Xinhai | Sow farm | Liuzhou Xinliu | Sow farm |
| Rugao Xinhao | Fattening Farm | Luocheng Xinhao | Sow farm | Kangping Xinwang | Fattening farm |
| Lezhi Xinhai | Fattening Farm | Hengyang Xinhao | Fattening Farm | Zhejiang Xinhai | Sow farm |
| Laixi Xinliu | Sow farm | Sichuan Xinhao | Sow farm | Zhangwu Xinwang | Sow farm |
| Zhangwu Xinwang | Fattening Farm | Tianxin Xinliu | Sow farm | Raoyang Xinhao | Sow farm |
| Guigang Xinliu | Sow farm | Heishan Xinliu | Sow farm | Sichuan Xinhao | Sow farm |
| Xiajin Nongmu | Sow farm | Qingyuan Xinhao | Fattening Farm | Taian Xinliu | Sow farm |
| Liaocheng Xinhao | Sow farm | Weinan Xinliu | Sow farm | Tongliao Xinhao | Sow farm |
| Laixi Xinliu | Sow farm | Heishan Xinliu | Sow farm | Hainan Agriculture and Reclamation | Sow farm |
| Taian Xinliu | Fattening Farm | Zhuocheng Xinliu | Fattening Farm | Meishan Xinhai | Sow farm |
| Meishan Xinhai | Sow farm | Tianxin Xinliu | Fattening Farm | Heishan Xinliu | Sow farm |
| Fucheng Xinhao | Sow farm | Dong'e Xinliu | Fattening Farm | Guang'an Xinhao | Sow farm |
| Qingfeng Xinliu | Sow farm | Tongliao Xinhao | Sow farm | Dongying Xinhao | Sow farm |
| Xiajin Nongmu | Sow farm | Xinji Xinliu | Sow farm | Hengnan Muyun | Sow farm |
| Pengshui Xinliu | Fattening Farm | Guigang Xinliu | Fattening Farm | Danxian Xinliu | Sow farm |
| Dongying Xinhao | Sow farm | Xinji Xinliu | Sow farm | Tianjin Xinliu | Fattening farm |
| Liuzhou Xinhao | Fattening Farm | Guigang Xinliu | Sow farm | Laizhou Xinhai | Sow farm |
| Qingfeng Xinliu | Sow farm | Xiangshui Xinchi | Fattening Farm | Danxian Xinhao | Sow farm |
| Heishan Xinliu | Sow farm | Shuozhou Xinhao | Fattening Farm | Shenze Xinhao | Sow farm |
| Pengshui Xinliu | Fattening Farm | Xinji Xinliu | Sow farm | Nanchong Xinhao | Sow farm |
| Tianjin Xinliu | Sow farm | Shangcai Xinliu | Fattening Farm | Sichuan Xinhao | Sow farm |
| Lingao Xinliu | Fattening Farm | Xiangshui Xinchi | Fattening Farm | Laizhou Xinhai | Sow farm |
| Dongying Xinhao | Sow farm | Zhuocheng Xinliu | Fattening Farm | Ruzhou Quansheng | Sow farm |
| Laixi Xinliu | Sow farm | Hainan Xinliu | Sow farm | Dongying Xinhao | Sow farm |
| Shanxian Xinhao | Sow farm | Langzhong Xinliu | Sow farm | Huanghua Xinhao | Sow farm |
| Hebei Xinhao | Sow farm | Xinji Xinliu | Sow farm | Longhui Xinhai | Sow farm |
| Suining Xinliu | Sow farm | Laizhou Xinhai | Sow farm | Luocheng Xinhao | Sow farm |
| Binzhou Xinhai | Fattening Farm | Shuozhou Xinhao | Sow farm | Zhangwu Xinwang | Fattening farm |
| Qingfeng Xinliu | Sow farm | Shenze Xinhao | Sow farm | Dongying Xinhao | Sow farm |
| Lingao Xinliu | Fattening Farm | Zhangwu Xinwang | Fattening Farm | Taian Xinliu | Sow farm |
| Nanning Xinliu | Sow farm | Jingxian Xinhao | Sow farm | Danxian Xinliu | Fattening farm |
| Sichuan Xinhao | Sow farm | Fucheng Xinhao | Sow farm | Dongying Xinhao | Sow farm |
| Nantong Xinhai | Sow farm | Sichuan Xinhao | Sow farm | Huaiyin Xincheng | Sow farm |
| Luocheng Xinhao | Sow farm | Guang'an Xinhao | Sow farm | Anyang Xinliu | Sow farm |
| Jiangxi Xinliu | Sow farm | Liaoning Xinwang | Sow farm | Huanghua Xinhao | Sow farm |
| Dongying Xinhao | Sow farm | Sichuan Xinhao | Sow farm | Shenze Xinhao | Sow farm |
| Tianjin Xinliu | Sow farm | Guang'an Xinhao | Fattening Farm | Jiexi Xinliu | Sow farm |
| Laizhou Xinhai | Sow farm | Tongliao Xinhao | Fattening Farm | Luocheng Xinhao | Sow farm |
| Jingxian Xinhao | Sow farm | Guanglü Xinliu | Sow farm | Dongying Xinhao | Sow farm |
| Guigang Xinliu | Sow farm | Laizhou Xinhai | Sow farm | Sichuan Xinhao | Sow farm |
| Meishan Xinhai | Sow farm | Dongying Xinhao | Sow farm | Hainan Xinliu | Sow farm |
| Donghai Xinhao | Sow farm | Ningbo Xinhai | Sow farm | Luocheng Xinhao | Sow farm |
| Qingfeng Xinliu | Sow farm | Shenze Xinhao | Sow farm | Sichuan Xinhao | Sow farm |
| Fuxin Xinwang | Fattening Farm | Laizhou Xinhai | Sow farm | Ruzhou Quansheng | Sow farm |
| Henan Nongmu | Sow farm | Shenze Xinhao | Sow farm | Xindongwan Xinliu | Sow farm |
| Henan Nongmu | Fattening Farm | Xinji Xinliu | Sow farm | Yichun Xinwang | Sow farm |
| Hengyang Xinhao | Fattening Farm | Huaiyin Xinchi | Sow farm | Guang'an Xinhao | Fattening farm |
| Caoxian Xinhao | Fattening Farm | Nanning Xinhao | Sow farm | Dongying Xinhao | Sow farm |
| Gansu Xinhao | Fattening Farm | Danxian Xinliu | Fattening Farm | Wuhe Xinhai | Sow farm |
| Sichuan Xinhao | Sow farm | Laizhou Xinhai | Sow farm | Rongchang Pig Farm | Sow farm |
| Sichuan Xinhao | Sow farm | Laixi Xinliu | Sow farm | Luocheng Xinhao | Sow farm |
| Fuxin Xinwang | Sow farm | Zhangwu Xinwang | Sow farm | Shuozhou Xinhao | Sow farm |
| Wuhe Xinhai | Sow farm | Taian Xinliu | Fattening Farm | Laiyang Xinmu | Sow farm |
| Shangcai Xinliu | Fattening Farm | Sichuan Xinhao | Sow farm | Laizhou Xinhai | Sow farm |
| Dong'e Xinliu | Fattening Farm | Anyue Xing Xin Xin | Fattening Farm | Sichuan Xinhao | Sow farm |
| Hainan Xinliu | Sow farm | Heishan Xinliu | Sow farm | Ruzhou Quansheng | Sow farm |
| Shuozhou Xinhao | Fattening Farm | Guigang Xinliu | Sow farm | Tongliao Xinhao | Fattening farm |
| Guang'an Xinhao | Fattening Farm | Xuanwei Xinliu | Sow farm | Zhenjiang Xinhai | Sow farm |
| Qingyuan Xinhao | Fattening Farm | Taian Xinliu | Fattening Farm | Laixi Xinliu | Sow farm |
| Zhucheng Xinliu | Fattening Farm | Sichuan Xinhao | Sow farm | Zhangwu Xinwang | Sow farm |
| Laizhou Xinhai | Sow farm | Tongliao Xinhao | Sow farm | Heishan Xinliu | Sow farm |
| Taian Xinliu | Fattening Farm | Raoyang Xinhao | Sow farm | Longhui Xinhai | Sow farm |
| Laixi Xinliu | Sow farm | Jian Xinchi | Fattening Farm | Ruzhou Quansheng | Sow farm |
| Xiangshui Xincheng | Fattening Farm | Guigang Xinliu | Sow farm | Jian Xinchi | Fattening farm |
| Lingbao Xinliu | Sow farm | Weinan Xinliu | Sow farm | Wulian Xinhao | Sow farm |
| Shangcai Xinliu | Fattening Farm | Rongchang Pig Farm | Sow farm | Yichun Xinwang | Fattening farm |
| Dingzhou Xinhao | Sow farm | Nanning Xinliu | Sow farm | Tongliao Xinhao | Sow farm |
| Zhucheng Xinliu | Fattening Farm | Heishan Xinliu | Fattening Farm | Suining Xinliu | Sow farm |
| Jingxian Xinhao | Sow farm | Lingbao Xinliu | Sow farm | Shibing Xinliu | Sow farm |
| Wuming Xinliu | Sow farm | Tongliao Xinhao | Fattening Farm | Yantai Xinhao | Sow farm |
| Zhangjiakou Xinwang | Sow farm | Zhangwu Xinwang | Sow farm | Danxian Xinliu | Sow farm |
| Taian Xinliu | Fattening Farm | Donghai Xinhao | Sow farm | Weinan Xin Liu | Sow farm |
| Dongying Xinhao | Sow farm | Gaotang Xinhao | Sow farm | Gaotang Xin Hao | Sow farm |
| Jian Xinchi | Sow farm | Laiyang Xinmu | Sow farm | Linshu Pig Breeding | Sow farm |
| Guigang Xinliu | Sow farm | Gaomi Xinliu | Fattening Farm | Laizhou Xin Hai | Sow farm |
| Fucheng Xinhao | Sow farm | Dingzhou Xinhao | Sow farm | Jian Xin Chi | Sow farm |
| Yantai Xinhao | Sow farm | Anhui Xinliu | Sow farm | Gansu Xin Hao | Sow farm |
| Santai Agriculture and Animal Husbandry | Fattening Farm | Danxian Xinliu | Sow farm | Wuhe Xin Hai | Sow farm |
| Laiyang Xinmu | Sow farm | Guanling Xinhai | Fattening Farm | Laizhou Xin Hai | Sow farm |
| Sichuan Xinhao | Sow farm | Zhangwu Xinwang | Sow farm | Gansu Xin Liu | Sow farm |
| Gaomi Xinliu | Fattening Farm | Zhenjiang Xinhai | Sow farm | Zhangwu Xin Wang | Fattening farm |
| Laizhou Xinhai | Sow farm | Dezhou Xinhao | Sow farm | Longzhou Xin Hao | Fattening farm |
| Sichuan Xinhao | Sow farm | Longhui Xinhai | Sow farm | Shangcai Xin Liu | Fattening farm |
| Xinji Xinliu | Sow farm | Tongliao Xinhao | Sow farm | Shanxian Xin Hao | Sow farm |
| Tongliao Xinhao | Fattening Farm | Yantai Xinhao | Sow farm | Laixi Xin Liu | Sow farm |
| Neiqiu Xinliu | Fattening Farm | Laiyang Xinmu | Fattening Farm | Laizhou Xin Hai | Sow farm |
| Guangan Xinhao | Sow farm | Heishan Xinliu | Fattening Farm | Anhui Xin Liu | Sow farm |
| Suining Xinliu | Sow farm | Yichun Xinwang | Fattening Farm | Laixi Xin Liu | Sow farm |
| Laiyang Xinmu | Fattening Farm | Dongying Xinhao | Sow farm | Yantai Xin Hao | Sow farm |
| Liuzhou Xinliu | Sow farm | Gansu Xinliu | Sow farm | Fuxin Xin Wang | Sow farm |
| Yantai Xinhao | Sow farm | Liaoning Xintao Wang | Sow farm | Lingbao Xin Liu | Sow farm |
| Zhenjiang Xinhai | Sow farm | Zhejiang Xinhai | Sow farm | Wuhe Xin Hai | Sow farm |
| Puyang Xinliu | Fattening Farm | Gaomi Xinliu | Sow farm | Liaoning Xin Tao Wang | Sow farm |
| Linchu Seed Pig | Sow farm | Kangping Xinwang | Fattening Farm | Shanxian Xin Hao | Sow farm |
| Shanxian Xinliu | Sow farm | Liaoning Xintao Wang | Sow farm | Wuqi Xin Liu | Sow farm |
| Zhejiang Xinhai | Sow farm | Hebei Xinhao | Sow farm | Weinan Xin Liu | Sow farm |
| Jian Xinchi | Sow farm | Fuxin Xinwang | Sow farm | Langzhong Xin Liu | Fattening farm |
| Zhejiang Xinhai | Fattening Farm | Dezhou Xinhao | Sow farm | Liaoning Xin Tao Wang | Sow farm |
| Binzhou Xinhai | Sow farm | Dezhou Xinhao | Sow farm | Jian Xin Chi | Sow farm |
| Ningbo Xinhai | Sow farm | Weinan Xinliu | Sow farm | Shanxian Xin Hao | Sow farm |
| Guigang Xinliu | Fattening Farm | Dongying Xinhao | Sow farm | Henan Agriculture and Animal Husbandry | Sow farm |
| Zhangwu Xinwang | Fattening Farm | Shibing Xinliu | Sow farm | Weihui Xin Chi | Sow farm |
| Guanling Xinhai | Fattening Farm | Guangan Xinhao | Sow farm | Dingzhou Xin Hao | Sow farm |
| Dongying Xinhao | Sow farm | Henan Nongmu | Sow farm | Kangping Xin Wang | Sow farm |
| Laixi Xinliu | Sow farm | Binzhou Xinhai | Sow farm | Raoyang Xin Hao | Sow farm |
| Gaomi Xinliu | Sow farm | Luocheng Xinhao | Sow farm | Ruzhou Quansheng | Sow farm |
| Xiangshui Xinchi | Fattening farm | Dongying Xinhao | Sow farm | Shanxian Xin Liu | Sow farm |
| Yantai Xinhao | Sow farm | Jian Xinchi | Sow farm | Gansu Xin Hao | Sow farm |
| Weinan Xinliu | Sow farm | Guangan Xinhao | Sow farm | Anyang Xin Liu | Sow farm |
| Gaotang Xinhao | Sow farm | Laixi Xinliu | Sow farm | Zhangwu Xin Wang | Fattening farm |
| Anhui Xinliu | Sow farm | Tangshan Xinhao | Sow farm | Xindongwan Xin Liu | Sow farm |
| Caoxian Xinhao | Sow farm | Laizhou Xinhai | Sow farm | Kangping Xin Wang | Sow farm |
| Xiangyang Xinhao | Sow farm | Dongying Xinhao | Sow farm | Laizhou Xin Hai | Sow farm |
| Rongchang Pig Farm | Sow farm | Shangxian Xinliu | Sow farm | Gaomi Xin Liu | Sow farm |
| Yantai Xinhao | Sow farm | Tongliao Xinhao | Fattening farm | Shuozhou Xin Hao | Sow farm |
| Liaoning Xintao Wang | Sow farm | Tangshan Xinhao | Sow farm | Leshan Agriculture and Animal Husbandry | Sow farm |
| Yingtan Xinliu | Fattening farm | Hengshui Nongmu | Sow farm | Yinbao Breeding | Sow farm |
| Dongying Xinhao | Sow farm | Luding Xinyue | Fattening farm | Laizhou Xin Hai | Sow farm |
| Dezhou Xinhao | Sow farm | Luocheng Xinhao | Sow farm | Bijie Xin Liu | Fattening farm |
| Zhangwu Xinwang | Sow farm | Wuhe Xinhai | Sow farm | Pengshui Xin Liu | Fattening farm |
| Dezhou Xinhao | Sow farm | Liaocheng Xinhao | Sow farm | Laizhou Xin Hai | Sow farm |
| Yantai Xinhao | Sow farm | Kangping Xinwang | Sow farm | Shanxian Xin Hao | Sow farm |
| Liaoning Xintao Wang | Sow farm | Yantai Xinhao | Sow farm | Laizhou Xin Hai | Sow farm |
| Heishan Xinliu | Fattening farm | Luocheng Xinhao | Sow farm | Langzhong Xin Liu | Sow farm |
| Laizhou Xinhai | Sow farm | Jingxian Xinhao | Sow farm | Jiaxiang Xin Liu | Sow farm |
| Wuqi Xinliu | Sow farm | Shangxian Xinhao | Sow farm | Ruyuan Xin Hao | Fattening farm |
| Donghai Xinhao | Sow farm | Jiangxi Xinliu | Sow farm | Xiangyang Xin Hao | Sow farm |
| Guangan Xinhao | Sow farm | Donghai Xinhao | Sow farm | Liaoning Xin Tao Wang | Sow farm |
| Jiexi Xinliu | Fattening farm | Luding Xinyue | Fattening farm | Gansu Xin Hao | Sow farm |
| Gansu Xinliu | Sow farm | Yantai Xinhao | Sow farm | Taian Xin Liu | Sow farm |
| Shanxian Xinhao | Sow farm | Wuqi Xinliu | Sow farm | Yantai Xin Hao | Sow farm |
| Dongying Xinhao | Sow farm | Yantai Xinhao | Sow farm | Gansu Xin Hao | Sow farm |
| Shanxian Xinhao | Sow farm | Shangxian Xinhao | Sow farm | Liaoning Xin Wang | Sow farm |
| Chongren Xinhai | Fattening farm | Luocheng Xinhao | Sow farm | Yantai Xin Hao | Sow farm |
| Binzhou Xinhai | Sow farm | Jian Xinchi | Sow farm | Guigang Xin Liu | Sow farm |
| Dongying Xinhao | Sow farm | Shangxian Xinliu | Sow farm | Baiyin Agriculture and Animal Husbandry | Sow farm |
| Xinji Xinliu | Sow farm | Dongying Xinhao | Sow farm | Gaotang Xin Hao | Sow farm |
| Laizhou Xinhai | Sow farm | Dongying Xinhao | Sow farm | Liaocheng Xin Hao | Sow farm |
| Heishan Xinliu | Sow farm | Tongliao Xinhao | Sow farm | Shuozhou Xin Hao | Sow farm |
| Pingfu Agriculture and Animal Husbandry | Sow farm | Tongliao Xinhao | Sow farm | Qinghua Agriculture and Animal Husbandry | Sow farm |
| Luo Cheng Xinhao | Sow farm | Laizhou Xinhai | Sow farm | Zhejiang Xin Hai | Sow farm |
| Shibing Xinliu | Sow farm | Tongliao Xinhao | Sow farm | Dongying Xin Hao | Sow farm |
| Fucheng Xinhao | Sow farm | Jiaxiang Xinliu | Sow farm | Dongying Xin Hao | Sow farm |
| Yantai Xinhao | Sow farm | Guangan Xinhao | Sow farm | Juye Xin Hao | Sow farm |
| Kangping Xinwang | Sow farm | Laizhou Xinhai | Sow farm | Dongying Xin Hao | Sow farm |
| Jiangxi Xinliu | Sow farm | Shangxian Xinhao | Sow farm | Weinan Xin Liu | Sow farm |
| Ruyuan Xinhao | Fattening farm | Liaoning Xintao Wang | Sow farm | Dongying Xin Hao | Sow farm |
| Nanning Xinhao | Sow farm | Tongliao Xinhao | Sow farm | Dongying Xin Hao | Sow farm |
| Laizhou Xinhai | Sow farm | Tongliao Xinhao | Sow farm | Laiyang Xin Hao | Sow farm |
| Xiangyang Xinhao | Sow farm | Weinan Xinliu | Sow farm | Liaoning Xin Wang | Sow farm |
| Lezhi Xinhai | Fattening farm | Tongliao Xinhao | Sow farm | Jingxian Xin Hao | Sow farm |
| Laizhou Xinhai | Sow farm | Laizhou Xinhai | Sow farm | Tongliao Xin Hao | Sow farm |
| Laizhou Xinhai | Sow farm | Tongliao Xinhao | Sow farm | Longzhou Xin Hao | Sow farm |
| Xinji Xinliu | Sow farm | Dongying Xinhao | Sow farm | Dongying Xin Hao | Sow farm |
| Dongying Xinhao | Sow farm | Liaocheng Xinhao | Sow farm | Pengshui Xin Liu | Fattening farm |
| Liaoning Xintao Wang | Sow farm | Yantai Xinhao | Sow farm | Shibing Xin Liu | Sow farm |
| Luo Cheng Xinhao | Sow farm | Dongying Xinhao | Sow farm | Jiexi Xin Liu | Fattening farm |
| Jian Xinchi | Sow farm | Laizhou Xinhai | Sow farm | Pingfu Agriculture and Animal Husbandry | Sow farm |
| Luo Cheng Xinhao | Sow farm | Yinbao Breeding | Sow farm | Guigang Xin Liu | Sow farm |
| Dongying Xinhao | Sow farm | Tongliao Xinhao | Sow farm | Yantai Xin Hao | Sow farm |
| Yantai Xinhao | Sow farm | Yantai Xinhao | Sow farm | Santai Agriculture and Animal Husbandry | Sow farm |
| Guangan Xinhao | Sow farm | Dongying Xinhao | Sow farm | Yantai Xin Hao | Sow farm |
| Tongliao Xinhao | Sow farm | Zhejiang Xinhai | Fattening farm | Yantai Xin Hao | Sow farm |
| Luo Cheng Xinhao | Sow farm | Jian Xinchi | Sow farm | Zhucheng Xin Liu | Sow farm |
| Xuanwei Xinliu | Sow farm | Dongying Xinhao | Sow farm | Laixi Xin Liu | Sow farm |
| Luding Xinyue | Fattening farm | Ruzhou Quansheng | Sow farm | Caoxian Xin Hao | Sow farm |
| Guangan Xinhao | Sow farm | Shangxian Xinhao | Sow farm | Dongying Xin Hao | Sow farm |
| Dongying Xinhao | Sow farm | Longhui Xinhai | Sow farm | Hubei Xin Hao | Sow farm |
| Liaocheng Xinhao | Sow farm | Dongying Xinhao | Sow farm | Longzhou Xin Hao | Sow farm |
| Shanxian Xinhao | Sow farm | Suning Xinliu | Sow farm | Yantai Xin Hao | Sow farm |
| Guigang Xinliu | Fattening farm | Xiangyang Xinhao | Sow farm | Liangshan Xin Liu | Sow farm |
| Wuhe Xinhai | Sow farm | Leshan Nongmu | Sow farm | Yantai Xin Hao | Sow farm |
| Dongying Xinhao | Sow farm | Dongying Xinhao | Sow farm | Yan'an Benyuan | Sow farm |
| Dongying Xinhao | Sow farm | Binzhou Xinhai | Sow farm | Pucheng Xin Liu | Sow farm |
| Guanglv Xinliu | Sow farm | Jiangxi Xinliu | Sow farm | Longzhou Xin Hao | Sow farm |
| Zhenyuan Xinhai | Sow farm | Ruzhou Quansheng | Sow farm | Guangyuan Xin Hao | Sow farm |
| Dongying Xinhao | Sow farm | Tongliao Xinhao | Sow farm | Longzhou Xin Hao | Sow farm |
| Linyi Seed Pig | Sow farm | Shibing Xinliu | Sow farm | Nanchong Xin Hao | Fattening farm |
| Shanxian Xinliu | Sow farm | Yantai Xinhao | Sow farm | Linyi Pig Breeding | Sow farm |
| Laizhou Xinhai | Sow farm | Juye Xinhao | Sow farm | Dongying Xin Hao | Sow farm |
| Yinbao Breeding | Sow farm | Caoxian Xinhao | Sow farm | Longzhou Xin Hao | Sow farm |
| Jingxian Xinhao | Sow farm | Linyi Seed Pig | Sow farm | Longzhou Xin Hao | Sow farm |
| Longzhou Xinhao | Fattening farm | Gaotang Xinhao | Sow farm | Zhenjiang Xin Hai | Sow farm |
| Ruzhou Quansheng | Sow farm | Wuhe Xinhai | Sow farm | Weinan Xin Liu | Sow farm |
| Zhangwu Xinwang | Sow farm | Xiangyang Xinhao | Sow farm | Dongying Xin Hao | Fattening farm |
| Weinan Xinliu | Sow farm | Ruzhou Quansheng | Sow farm | Longzhou Xin Hao | Sow farm |
| Weinan Xinliu | Sow farm | Yantai Xinhao | Sow farm | Guang'an Xin Hao | Fattening farm |
| Dongying Xinhao | Sow farm | Yantai Xinhao | Sow farm | Kangping Xin Wang | Sow farm |
| Zhucheng Xinliu | Sow farm | Wuhe Xinhai | Sow farm | Zhongshan Agriculture and Animal Husbandry | Sow farm |
| Yantai Xinhao | Sow farm | Ruyuan Xinhao | Fattening farm | Fuxin Xin Wang | Sow farm |
| Juye Xinhao | Sow farm | Guangan Xinhao | Sow farm | Qingyuan Xin Hao | Fattening farm |
| Guangan Xinhao | Sow farm | Shangcai Xinliu | Fattening farm | Wuhe Xin Hai | Sow farm |
| Shanxian Xinhao | Sow farm | Laiyang Xinhao | Sow farm | Jian Xin Chi | Sow farm |
| Wulian Xinhao | Sow farm | Suning Xinliu | Sow farm | Heishan Xin Liu | Sow farm |
| Jiaxiang Xinliu | Sow farm | Bijie Xinliu | Fattening farm | Jian Xin Chi | Sow farm |
| Gansu Xinhao | Sow farm | Zhucheng Xinliu | Sow farm | Guangdong Xin Hao | Sow farm |
| Zhangwu Xinwang | Sow farm | Yichun Xinwang | Sow farm | Wuhe Xin Hai | Sow farm |
| Anyue Xing Xinxin | Fattening farm | Heishan Xinliu | Sow farm | Yingcheng Xin Hao | Sow farm |
| Bijie Xin Liu | Fattening farm | Dongying Xinhao | Sow farm | Hezhou Xinhao | Sow farm |
| Kangping Xin Wang | Sow farm | Longzhou Xinhao | Sow farm | Zhenjiang Xinhai | Sow farm |
| Xiangshui Xin Chi | Fattening farm | Hengnan Muyun | Sow farm | Shanxian Xinliu | Sow farm |
| Laiyang Xin Hao | Sow farm | Longzhou Xinhao | Fattening farm | Langzhong Xinliu | Sow farm |
| Tianjin Xin Liu | Fattening farm | Qinghua Nongmu | Sow farm | Jiangxi Xinliu | Sow farm |
| Liaoning Xin Tao Wang | Sow farm | Pingfu Nongmu | Sow farm | Jingxian Xinhao | Sow farm |
| Xinji Xin Liu | Sow farm | Pucheng Xinliu | Sow farm | HeishanWoniu | Sow farm |
| Qingyuan Xin Hao | Fattening farm | Santai Nongmu | Sow farm | Jiangyou Pig Farm | Sow farm |
| Longzhou Xin Hao | Sow farm | Heishan Xinliu | Sow farm | Wuhe Xinhai | Sow farm |
| Dongying Xin Hao | Sow farm | Longzhou Xinhao | Sow farm | Jiangxi Xinliu | Sow farm |
| Tongliao Xin Hao | Sow farm | Longzhou Xinhao | Sow farm | Xiangyang Xinhao | Sow farm |
| Leshan Nongmu | Sow farm | Longzhou Xinhao | Sow farm | Qingfeng Xinliu | Sow farm |
| Weinan Xin Liu | Sow farm | Yan'an Benyuan | Sow farm | Ruyuan Xinhao | Sow farm |
| Xinji Xin Liu | Sow farm | Longzhou Xinhao | Sow farm | Donghai Xinhao | Sow farm |
| Gaotang Xin Hao | Sow farm | Hubei Xinhao | Sow farm | Liaoning New Taowang | Sow farm |
| Longzhou Xin Hao | Sow farm | Pengshui Xinliu | Fattening farm | Shuozhou Xinhao | Sow farm |
| Yantai Xin Hao | Sow farm | Gansu Xinhao | Sow farm | Guangan Xinhao | Fattening farm |
| Pengshui Xin Liu | Fattening farm | Liangshan Xinliu | Sow farm | Tongliao Xinhao | Sow farm |
| Longzhou Xin Hao | Sow farm | Liaoning Xinwang | Sow farm | Chenzhou Xinhao | Sow farm |
| Tongliao Xin Hao | Fattening farm | Longzhou Xinhao | Sow farm | Dongying Xinhao | Sow farm |
| Linyi Xin Hao | Sow farm | Shuozhou Xinhao | Sow farm | Fuxin Xinwang | Sow farm |
| Liaocheng Xin Hao | Sow farm | Gansu Xinhao | Sow farm | Liaoning New Taowang | Sow farm |
| Gansu Xin Hao | Sow farm | Kangping Xinwang | Sow farm | Longhui Xinhai | Fattening farm |
| Longzhou Xin Hao | Sow farm | Gansu Xinhao | Sow farm | Qingyuan Xinhao | Fattening farm |
| Santai Nongmu | Sow farm | Liaoning Xintao Wang | Sow farm | Zhangjiakou Xinwang | Sow farm |
| Linyi Xin Hao | Sow farm | Ruzhou Quansheng | Sow farm | Dongying Xinhao | Sow farm |
| Ruzhou Quansheng | Sow farm | Weinan Xinliu | Sow farm | Liaoning New Taowang | Sow farm |
| Kangping Xin Wang | Fattening farm | Langzhong Xinliu | Fattening farm | Gaotang Xinhao | Sow farm |
| Longzhou Xin Hao | Sow farm | Tongliao Xinhao | Sow farm | Liaoning New Taowang | Sow farm |
| Hubei Xin Hao | Sow farm | Longzhou Xinhao | Sow farm | Jiangxi Xinliu | Sow farm |
| Zhangjiakou Xin Wang | Sow farm | Tangshan Xinhao | Sow farm | Heishan Xinliu | Sow farm |
| Dezhou Xin Hao | Sow farm | Tangshan Xinhao | Sow farm | Liaoning Xinwang | Sow farm |
| Liangshan Xin Liu | Sow farm | Liaoning Xintao Wang | Sow farm | Guangan Xinhao | Fattening farm |
| Dongying Xin Hao | Sow farm | Fuxin Xinwang | Sow farm | Leshan Agriculture and Animal Husbandry | Sow farm |
| Qinghua Nongmu | Sow farm | Jiangxi Xinliu | Sow farm | Liaoning New Taowang | Sow farm |
| Pucheng Xin Liu | Sow farm | Jiexi Xinliu | Fattening farm | Liaocheng Xinhao | Sow farm |
| Wuhe Xin Hai | Sow farm | Changyi Xinhao | Sow farm | Hubei Xinhao | Sow farm |
| Pingfu Nongmu | Sow farm | Nanning Xinliu | Sow farm | Gansu Xinhao | Sow farm |
| Zhenjiang Xin Hai | Sow farm | Gansu Xinhao | Sow farm | Suining Xinliu | Sow farm |
| Baiyin Nongmu | Sow farm | Zhongshan Nongmu | Sow farm | Ji'an New Chi | Sow farm |
| Gansu Xin Hao | Sow farm | Heishan Woniou | Sow farm | Hainan State Farms | Sow farm |
| Yan'an Benyuan | Sow farm | Gansu Xinhao | Sow farm | Nanning Xinliu | Sow farm |
| Heishan Woniou | Sow farm | Taian Xinliu | Sow farm | Guangan Xinhao | Sow farm |
| Longzhou Xin Hao | Sow farm | Dezhou Xinhao | Sow farm | Lingbao Xinliu | Sow farm |
| Luding Xinyue | Fattening farm | Ruzhou Quansheng | Sow farm | Fuxin Xinwang | Sow farm |
| Shuozhou Xin Hao | Sow farm | Qingfeng Xinliu | Sow farm | Liaoning Xinwang | Sow farm |
| Yichun Xin Wang | Fattening farm | Weinan Xinliu | Sow farm | Juye Xinhao | Sow farm |
| Gaomi Xin Liu | Sow farm | Zhangjiakou Xinwang | Sow farm | Gansu Xinhao | Sow farm |
| Liaoning Xin Tao Wang | Sow farm | Jian Xinchi | Sow farm | Juye Xinhao | Sow farm |
| Tongliao Xin Hao | Sow farm | Yingcheng Xinhao | Sow farm | Gansu Xinhao | Sow farm |
| Dezhou Xin Hao | Sow farm | Jiangyou Pig Farm | Sow farm | Wulian Xinhao | Sow farm |
| Tongliao Xin Hao | Sow farm | Liaoning Xintao Wang | Sow farm | Gansu Xinhao | Sow farm |
| Jiangxi Xin Liu | Sow farm | Lingxian Xinliu | Sow farm | Liaoning Xinwang | Sow farm |
| Tongliao Xin Hao | Sow farm | Dongying Xinhao | Sow farm | Xingren Xinliu | Sow farm |
| Hebei Xin Hao | Sow farm | Jiexi Xinliu | Fattening farm | Changge New Chi | Sow farm |
| Gansu Xin Hao | Sow farm | Gaotang Xinhao | Sow farm | Guigang Xinliu | Sow farm |
| Changyi Xin Hao | Sow farm | Gansu Xinhao | Sow farm | Fuxin Xinwang | Sow farm |
| Liaoning Xin Wang | Sow farm | Zhangwu Xinwang | Fattening farm | Xiangyang Xinhao | Sow farm |
| Weinan Xin Liu | Sow farm | Ruyuan Xinhao | Sow farm | Gansu Xinhao | Sow farm |
| Ruzhou Quansheng | Sow farm | Nanchong Xinhao | Sow farm | Gansu Xinhao | Sow farm |
| Wulian Xin Hao | Sow farm | Liaoning Xinwang | Sow farm | Liaoning Xinwang | Sow farm |
| Kangping Xin Wang | Sow farm | Weihui Xincheng | Sow farm | Liaoning Xinwang | Sow farm |
| Guangyuan Xin Hao | Sow farm | Tangshan Xinhao | Sow farm | Ningbo Xinhai | Sow farm |
| Laiyang Xinmu | Sow farm | Liaoning Xintao Wang | Sow farm | Hainan State Farms | Sow farm |
| Xuanwei Xin Liu | Sow farm | Liaoning Xintao Wang | Sow farm | Changyi Xinhao | Sow farm |
| Wuhe Xin Hai | Sow farm | Gaomi Xinliu | Sow farm | Wulian Xinhao | Sow farm |
| Zhongshan Nongmu | Sow farm | Qingyuan Xinhao | Fattening farm | Juye Xinhao | Sow farm |
| Tongliao Xin Hao | Sow farm | Hainan Nongken | Sow farm | Baiyin Agriculture and Animal Husbandry | Sow farm |
| Tongliao Xin Hao | Sow farm | Linyi Xinhao | Sow farm | Zhejiang Xinhai | Sow farm |
| Liaocheng Xin Hao | Sow farm | Guangdong Xinhao | Sow farm | Laixi Xinliu | Sow farm |
| Heishan Xin Liu | Sow farm | Juye Xinhao | Sow farm | Xingren Xinliu | Sow farm |
| Ruzhou Quansheng | Sow farm | Gansu Xinhao | Sow farm | Xiangyang Xinhao | Sow farm |
| Gansu Xin Hao | Sow farm | Linyi Seed Pig | Sow farm | Suining Xinliu | Sow farm |
| Liaoning Xin Tao Wang | Sow farm | Gansu Xinhao | Sow farm | Chenzhou Xinhao | Sow farm |
| Laixi Xin Liu | Sow farm | Meishan Xinhai | Fattening farm | Gaomi Xinliu | Sow farm |
| Weinan Xin Liu | Sow farm | Gaomi Xinliu | Sow farm | Nanning Xinhao | Sow farm |
| Dezhou Xin Hao | Sow farm | Liaocheng Xinhao | Sow farm | Gaomi Xinliu | Sow farm |
| Pucheng Xin Liu | Sow farm | Zhangwu Xinwang | Sow farm | Gansu Xinhao | Sow farm |
| Jiangyou Zhuchang | Sow farm | Laixi Xinliu | Sow farm | Jingxian Xinhao | Sow farm |
| Tongliao Xin Hao | Sow farm | Wulian Xinhao | Sow farm | Yingcheng Xinhao | Sow farm |
| Ruyuan Xin Hao | Sow farm | Laixi Xinliu | Sow farm | Fuxin Xinwang | Sow farm |
| Longhui Xin Hai | Sow farm | Hubei Xinhao | Sow farm | Guangyuan Xinhao | Sow farm |
| Gansu Xin Hao | Sow farm | Jingxian Xinhao | Sow farm | Taian Xinliu | Sow farm |
| Gansu Xin Hao | Sow farm | Gaomi Xinliu | Sow farm | Jiangxi Xinliu | Sow farm |
| Ruzhou Quansheng | Sow farm | Changyi Xinhao | Sow farm | Guigang Xinliu | Sow farm |
| Fuxin Xin Wang | Sow farm | Guangyuan Xinliu | Sow farm | Laixi Xinliu | Sow farm |
| Hengnan Muyun | Sow farm | Linyi Xinhao | Sow farm | Guigang Xinliu | Sow farm |
| Yichun Xin Wang | Sow farm | Tangshan Xinhao | Sow farm | Liaoning Xinwang | Sow farm |
| Gaotang Xin Hao | Sow farm | Gansu Xinhao | Sow farm | Tongliao Xinhao | Sow farm |
| Juye Xin Hao | Sow farm | Tangshan Xinhao | Sow farm | Gansu Xinhao | Sow farm |
| Jiangxi Xin Liu | Sow farm | Anyang Xinliu | Sow farm | Shuozhou Xinhao | Sow farm |
| Liaoning Xin Tao Wang | Sow farm | Liuzhou Xinliu | Sow farm | Gansu Xinliu | Sow farm |
| Yucheng Xin Liu | Sow farm | Wuhe Xinhai | Sow farm | Tongliao Xinhao | Sow farm |
| Hainan Nongken | Sow farm | Liaoning Xinwang | Sow farm | Guangan Xinhao | Sow farm |
| Jian Xin Chi | Fattening farm | Lingbao Xinliu | Sow farm | Linyi Xinhao | Sow farm |
| Laixi Xin Liu | Sow farm | Juye Xinhao | Sow farm | Donghai Xinhao | Sow farm |
| Guangyuan Xin Hao | Sow farm | Yucheng Xinliu | Sow farm | Gansu Xinhao | Sow farm |
| Liaoning Xin Tao Wang | Sow farm | Zhenjiang Xinhai | Sow farm | Gansu Xinhao | Sow farm |
| Changyi Xin Hao | Sow farm | Guangan Xinhao | Fattening farm | Chenzhou Xinhao | Sow farm |
| Nanning Xin Liu | Sow farm | Gaomi Xinliu | Sow farm | Yingtan Xinliu | Sow farm |
| Gaomi Xin Liu | Sow farm | Wulian Xinhao | Sow farm | Anhui Xinliu | Sow farm |
| Pucheng Xin Liu | Sow farm | Changyi Xinhao | Sow farm | Gaomi Xinliu | Sow farm |
| Changyi Xin Hao | Sow farm | Gansu Xinhao | Sow farm | Liaoning Xinwang | Sow farm |
| Lingxin Xin Liu | Sow farm | Liaocheng Xinhao | Sow farm | Hubei Xinhao | Sow farm |
| Liaocheng Xin Hao | Sow farm | Hengnan Muyun | Sow farm | Nanning Xinhao | Sow farm |
| Juye Xin Hao | Sow farm | Tangshan Xinhao | Sow farm | Lingao Xinliu | Sow farm |
| Tongliao Xin Hao | Sow farm | Nanning Xinhao | Sow farm | Taian Xinliu | Sow farm |
| Heishan Xin Liu | Sow farm | Meishan Xinhai | Sow farm | Laixi Xinliu | Sow farm |
| Taipeng Xin Liu | Sow farm | Guangan Xinhao | Fattening farm | Laiyang Xinmu | Sow farm |
| Gaomi Xin Liu | Sow farm | Anhui Xinliu | Sow farm | Ningbo Xinhai | Sow farm |
| Hubei Xin Hao | Sow farm | Donghai Xinhao | Sow farm | Laiyang Xinhao | Sow farm |
| Juye Xin Hao | Sow farm | Liaoning Xinwang | Sow farm | Changyi Xinhao | Sow farm |
| Wuhe Xin Hai | Sow farm | Jiangxi Xinliu | Sow farm | Hubei Xinhao | Sow farm |
| Zhucheng Xin Liu | Sow farm | Gansu Xinliu | Sow farm | Liaocheng Xinhao | Sow farm |
| Kangping Xin Wang | Sow farm | Heishan Xinliu | Sow farm | Chenzhou Xinhao | Sow farm |
| Chenzhou Xin Hao | Sow farm | Laixi Xinliu | Sow farm | Pucheng Xinliu | Sow farm |
| Lingbao Xin Liu | Sow farm | Xiangyang Xinhao | Sow farm | Liaocheng Xinhao | Sow farm |
| Binzhou Xin Hai | Sow farm | Guigang Xinliu | Sow farm | Hengnan Pastoral Farming | Sow farm |
| Caoxian Xin Hao | Sow farm | Juye Xinhao | Sow farm | Baiyin Agriculture and Animal Husbandry | Sow farm |
| Gansu Xin Hao | Sow farm | Guangyuan Xinliu | Sow farm | Linyi Xinhao | Sow farm |
| Anyang Xin Liu | Sow farm | Fuxin Xinwang | Sow farm | Yucheng Xinliu | Sow farm |
| Gansu Xin Hao | Sow farm | Liaocheng Xinhao | Sow farm | Heishan Xinliu | Sow farm |
| Kangping Xin Wang | Sow farm | Zhejiang Xinhai | Sow farm | Santai Agriculture and Animal Husbandry | Sow farm |
| Fuxin Xin Wang | Sow farm | Wuhe Xinhai | Sow farm | Ruyuan Xinhao | Sow farm |
| Ruyuan Xin Hao | Sow farm | Guangyuan Xinliu | Sow farm | Hengnan Pastoral Farming | Sow farm |
| Zhucheng Xin Liu | Sow farm | Zhangjiakou Xinwang | Sow farm | Nanchong Xinhao | Sow farm |
| Binzhou Xin Hai | Sow farm | Heishan Xinliu | Sow farm | Gaomi Xinliu | Sow farm |
| Gaomi Xin Liu | Sow farm | Xiangyang Xinhao | Sow farm | Heishan Xinliu | Sow farm |
| Guigang Xin Liu | Fattening farm | Zhejiang Xinhai | Sow farm | Yingcheng Xinhao | Sow farm |
| Liaoning Xin Wang | Sow farm | Taian Xinliu | Sow farm | Liaocheng Xinhao | Sow farm |
| Gansu Xin Liu | Sow farm | Meishan Xinhai | Sow farm | Donghai Xinhao | Sow farm |
| Yingcheng Xin Hao | Sow farm | Leshan Nongmu | Sow farm | Zhu Cheng Xinliu | Sow farm |
| Nanning Xin Hao | Sow farm | Nanning Xinhao | Sow farm | Meishan Xinhai | Sow farm |
| Zhucheng Xin Liu | Sow farm | Pucheng Xinliu | Sow farm | Liaoning Xinwang | Sow farm |
| Xiangyang Xin Hao | Sow farm | Hengnan Muyun | Sow farm | Guigang Xinliu | Sow farm |
| Heishan Xin Liu | Sow farm | Kangping Xinwang | Sow farm | Taian Xinliu | Sow farm |
| Shuozhou Xin Hao | Sow farm | Hainan Nongken | Sow farm | Zhangjiakou Xinwang | Sow farm |
| Hebei Xin Hao | Sow farm | Fuxin Xinwang | Sow farm | Shuozhou Xinhao | Sow farm |
| Ruyuan Xin Hao | Sow farm | Liaoning Xinwang | Sow farm | Taian Xinliu | Sow farm |
| Tangshan Xin Hao | Sow farm | Laixi Xinliu | Sow farm | Heyang Xinliu | Sow farm |
| Meishan Xin Hai | Sow farm | Chenzhou Xinhao | Sow farm | Xiangyang Xinhao | Sow farm |
| Laixi Xin Liu | Sow farm | Guigang Xinliu | Sow farm | Gansu Xinhao | Sow farm |
| Hubei Xin Hao | Sow farm | Laixi Xinliu | Sow farm | Yucheng Xinliu | Sow farm |
| Tangshan Xin Hao | Sow farm | Kangping Xinwang | Sow farm | Heishan Xinliu | Sow farm |
| Tangshan Xin Hao | Sow farm | Guangyuan Xinliu | Sow farm | Juye Xinhao | Sow farm |
| Zhejiang Xin Hai | Sow farm | Ningbo Xinhai | Sow farm | Laixi Xinliu | Sow farm |
| Guigang Xin Liu | Sow farm | Gansu Xinhao | Sow farm | Shuozhou Xinhao | Sow farm |
| Zhangjiakou Xin Wang | Sow farm | Yingcheng Xinhao | Sow farm | Shuozhou Xinhao | Sow farm |
| Yingcheng Xin Hao | Sow farm | Chenzhou Xinhao | Sow farm | Hubei Xinhao | Sow farm |
| Anhui Xin Liu | Sow farm | Meishan Xinhai | Sow farm | Bijie Xinliu | Sow farm |
| Meishan Xin Hai | Sow farm | Gansu Xinhao | Sow farm | Meishan Xinhai | Sow farm |
| Liaoning Xin Wang | Sow farm | Chenzhou Xinhao | Sow farm | Baiyin Agriculture and Animal Husbandry | Sow farm |
| Baiyin Nongmu | Sow farm | Hubei Xinhao | Sow farm | Donghai Xinhao | Sow farm |
| Zhangjiakou Xin Wang | Sow farm | Xingren Xinliu | Sow farm | Hengnan Pastoral Farming | Sow farm |
| Guigang Xin Liu | Sow farm | Gansu Xinhao | Sow farm | Shuozhou Xinhao | Sow farm |
| Caoxian Xinhao | Sow farm | Nanchong Xinhao | Sow farm | Meishan Xinhai | Sow farm |
| Longzhou Xinhao | Sow farm | Zhu Cheng Xinliu | Sow farm | Anyue Xing Xin Xin | Sow farm |
| Meishan Xinhai | Fattening farm | Guigang Xinliu | Sow farm | Pucheng Xinliu | Sow farm |
| Liaoning New Hope | Sow farm | Laixi Xinliu | Sow farm | Hubei Xinhao | Sow farm |
| Chenzhou Xinhao | Sow farm | Nanchong Xinhao | Fattening farm | Juye Xinhao | Sow farm |
| Hainan Agriculture | Sow farm | Zhu Cheng Xinliu | Sow farm | Laibin Xinhao | Sow farm |
| Heishan Xinliu | Sow farm | Hengnan Muyun | Sow farm | Hezhou Xinhao | Sow farm |
| Weinan Xinliu | Sow farm | Shuozhou Xinhao | Sow farm | Guangyuan Xinhao | Sow farm |
| Guangdong Xinhao | Sow farm | Hubei Xinhao | Sow farm | Guangyuan Xinhao | Sow farm |
| Liaoning New Hope | Sow farm | Kangping New Hope | Sow farm | Yingcheng Xinhao | Sow farm |
| Guangan Xinhao | Fattening farm | Laixi Xinliu | Sow farm | Yingcheng Xinhao | Sow farm |
| Langzhong Xinliu | Fattening farm | Heishan Xinliu | Sow farm | Nanning Xinhao | Sow farm |
| Gansu Xinliu | Sow farm | Donghai Xinhao | Sow farm | Changyi Xinhao | Sow farm |
| Jiexi Xinliu | Fattening farm | Laiyang Xinhao | Sow farm | Hubei Xinhao | Sow farm |
| Taian Xinliu | Sow farm | Weinan Xinliu | Sow farm | Hubei Xinhao | Sow farm |
| Hengnan Muyun | Sow farm | Taian Xinliu | Sow farm | Hainan Xinliu | Sow farm |
| Guangyuan Xinhao | Sow farm | Wuhe Xinhai | Sow farm | Pucheng Xinliu | Sow farm |
| Dong'e Xinliu | Sow farm | Dezhou Xinhao | Sow farm | Ruyuan Xinhao | Sow farm |
| Chenzhou Xinhao | Fattening farm | Liaoning New Hope | Sow farm | Dong'e Xinliu | Sow farm |
| Laixi Xinliu | Sow farm | Gansu Xinhao | Sow farm | Yichun New Hope | Sow farm |
| Nanchong Xinhao | Sow farm | Shuozhou Xinhao | Sow farm | Qinghua Agriculture and Animal Husbandry | Sow farm |
| Hengnan Muyun | Sow farm | Pucheng Xinliu | Sow farm | Pucheng Xinliu | Sow farm |
| Laixi Xinliu | Sow farm | Yingcheng Xinhao | Sow farm | Guanling Xinhai | Sow farm |
| Shuozhou Xinhao | Sow farm | Caoxian Xinhao | Sow farm | Guangdong Xinhao | Sow farm |
| Hubei Xinhao | Sow farm | Baiyin Agriculture and Animal Husbandry | Sow farm | Nanning Xinliu | Sow farm |
| Kangping New Hope | Sow farm | Fuxin New Hope | Fattening farm | Ningming Xinhao | Sow farm |
| Gansu Xinhao | Sow farm | Zhangwu New Hope | Fattening farm | Laixi Xinliu | Sow farm |
| Yucheng Xinliu | Sow farm | Pucheng Xinliu | Sow farm | Laibin Xinhao | Sow farm |
| Xiangyang Xinhao | Sow farm | Weinan Xinliu | Sow farm | Yingcheng Xinhao | Sow farm |
| Zhuocheng Xinliu | Sow farm | Hubei Xinhao | Sow farm | Laibin Xinhao | Sow farm |
| Gansu Xinhao | Sow farm | Meishan Xinhai | Sow farm | Guangdong Xinhao | Sow farm |
| Yingcheng Xinhao | Sow farm | Taian Xinliu | Sow farm | Zhu Cheng Xinliu | Sow farm |
| Pucheng Xinliu | Sow farm | Ningming Xinhao | Sow farm | Laibin Xinhao | Sow farm |
| Laibin Xinhao | Sow farm | Guigang Xinliu | Sow farm | Lingxin Xinliu | Sow farm |
| Guangyuan Xinhao | Sow farm | Yucheng Xinliu | Sow farm | Dong'e Xinliu | Sow farm |
| Guang'an Xinhao | Fattening farm | Yingcheng Xinhao | Sow farm | Kangping New Hope | Sow farm |
| Gansu Xinhao | Sow farm | Laixi Xinliu | Sow farm | Liaoning New Hope | Sow farm |
| Guigang Xinliu | Sow farm | Pingfu Agriculture and Animal Husbandry | Sow farm | Shuozhou Xinhao | Sow farm |
| Nanchong Xinhao | Sow farm | Kangping New Hope | Sow farm | Liaoning New Hope | Sow farm |
| Wuhe Xinhai | Sow farm | Laibin Xinhao | Sow farm | Caoxian Xinhao | Sow farm |
| Zhangjiakou New Hope | Sow farm | Caoxian Xinhao | Sow farm | Dong'e Xinliu | Sow farm |
| Liaoning New Hope | Sow farm | Linhai Xinliu | Sow farm | Guanling Xinhai | Sow farm |
| Hengnan Muyun | Sow farm | Bijie Xinliu | Sow farm | Liaoning New Hope | Sow farm |
| Weinan Xinliu | Sow farm | Liaoning New Hope | Sow farm | Dong'e Xinliu | Sow farm |
| Weihui Xinchi | Sow farm | Shuozhou Xinhao | Sow farm | Wuqi Xinliu | Sow farm |
| Gansu Xinhao | Sow farm | Yingtan Xinliu | Sow farm | Hezhou Xinhao | Sow farm |
| Shuozhou Xinhao | Sow farm | Chenzhou Xinhao | Sow farm | Sichuan Xinhao | Sow farm |
| Meishan Xinhai | Sow farm | Hainan Xinliu | Sow farm | Guangdong Xinhao | Sow farm |
| Heyang Xinliu | Sow farm | Pucheng Xinliu | Sow farm | Zhu Cheng Xinliu | Sow farm |
| Chenzhou Xinhao | Sow farm | Xingren Xinliu | Sow farm | Meishan Xinhai | Sow farm |
| Baiyin Agriculture | Sow farm | Dezhou Xinhao | Sow farm | Kangping New Hope | Sow farm |
| Laixi Xinliu | Sow farm | Ruyuan Xinhao | Sow farm | Guangdong Xinhao | Sow farm |
| Hubei Xinhao | Sow farm | Hubei Xinhao | Sow farm | Yijun Xinliu | Sow farm |
| Wuhe Xinhai | Sow farm | Taian Xinliu | Sow farm | Guangdong Xinhao | Sow farm |
| Tangshan Xinhao | Sow farm | Nanning Xinhao | Sow farm | Kangping New Hope | Sow farm |
| Hubei Xinhao | Sow farm | Dong'e Xinliu | Sow farm | Laibin Xinhao | Sow farm |
| Nanning Xinhao | Sow farm | Zhu Cheng Xinliu | Sow farm | Weinan Xinliu | Sow farm |
| Kangping New Hope | Sow farm | Hubei Xinhao | Sow farm | Bijie Xinliu | Sow farm |
| Heishan Xinliu | Sow farm | Laibin Xinhao | Sow farm | Laibin Xinhao | Sow farm |
| Jiaxiang Xinliu | Sow farm | Wuqi Xinliu | Sow farm | Baiyin Agriculture and Animal Husbandry | Sow farm |
| Laixi Xinliu | Sow farm | Heyang Xinliu | Sow farm | Nanning Xinhao | Sow farm |
| Dong'e Xinliu | Sow farm | Jiaxiang Xinliu | Sow farm | Hezhou Xinhao | Sow farm |
| Xingren Xinliu | Sow farm | Zhangjiakou New Hope | Sow farm | Hubei Xinhao | Sow farm |
| Ningming Xinhao | Sow farm | Guanling Xinhai | Sow farm | Laibin Xinhao | Sow farm |
| Guanling Xinhai | Sow farm | Shuozhou Xinhao | Sow farm | Gansu Xinliu | Sow farm |
| Nanning Xinhao | Sow farm | Santai Agriculture and Animal Husbandry | Sow farm | Wuqi Xinliu | Sow farm |
| Yingcheng Xinhao | Sow farm | Dong'e Xinliu | Sow farm | Nanning Xinliu | Sow farm |
| Pucheng Xinliu | Sow farm | Liaoning New Hope | Sow farm | Kangping New Hope | Sow farm |
| Gansu Xinhao | Sow farm | Zhu Cheng Xinliu | Sow farm | Hezhou Xinhao | Sow farm |
| Xiangyang Xinhao | Sow farm | Yingcheng Xinhao | Sow farm | Hubei Xinhao | Sow farm |
| Shuozhou Xinhao | Sow farm | Dong'e Xinliu | Sow farm | Hubei Xinhao | Sow farm |
| Tangshan Xinhao | Sow farm | Pengshui Xinliu | Fattening farm | Ningming Xinhao | Sow farm |
| Zhejiang Xinhai | Sow farm | Hezhou Xinhao | Sow farm | Weinan Xinliu | Sow farm |
| Leshan Agriculture | Sow farm | Xiangyang Xinhao | Sow farm | Kangping New Hope | Sow farm |
| Wuqi Xinliu | Sow farm | Wuhe Xinhai | Sow farm | Yijun Xinliu | Sow farm |
| Laiyang Xinhao | Sow farm | Hezhou Xinhao | Sow farm | Laiyang Xinhao | Sow farm |
| Dong'e Xinliu | Sow farm | Baiyin Agriculture and Animal Husbandry | Sow farm | Kangping New Hope | Sow farm |
| Caoxian Xinhao | Sow farm | Qinghua Agriculture and Animal Husbandry | Sow farm | Nanning Xinhao | Sow farm |
| Guangdong Xinhao | Sow farm | Gansu Xinhao | Sow farm | Kangping New Hope | Sow farm |
| Xingren Xinliu | Sow farm | Laibin Xinhao | Sow farm | Baiyin Agriculture and Animal Husbandry | Sow farm |
| Yingcheng Xinhao | Sow farm | Guang'an Xinhao | Fattening farm | Heyang Xinliu | Sow farm |
| Dong'e Xinliu | Sow farm | Dong'e Xinliu | Sow farm | Dong'e Xinliu | Sow farm |
| Baiyin Agriculture | Sow farm | Liaoning New Hope | Sow farm | Dong'e Xinliu | Sow farm |
| Taian Xinliu | Sow farm | Shuozhou Xinhao | Sow farm | Yijun Xinliu | Sow farm |
| Baiyin Agriculture | Sow farm | Laibin Xinhao | Sow farm | Ningming Xinhao | Sow farm |
| Yingtan Xinliu | Sow farm | Laibin Xinhao | Sow farm | Laibin Xinhao | Sow farm |
| Dong'e Xinliu | Sow farm | Juye Xinhao | Sow farm | Weinan Xinliu | Sow farm |
| Hezhou Xinhao | Sow farm | Guangdong Xinhao | Sow farm | Wuhe Xinhai | Sow farm |
| Liaoning New Hope | Sow farm | Juye Xinhao | Sow farm | Zhangjiakou New Hope | Sow farm |
| Hezhou Xinhao | Sow farm | Yichun New Hope | Sow farm | Liaoning New Hope | Sow farm |
| Nanning Xinhao | Sow farm | Dong'e Xinliu | Sow farm | Heishan Xinliu | Sow farm |
| Caoxian Xinhao | Sow farm | Zhangwu New Hope | Fattening farm | Baiyin Agriculture and Animal Husbandry | Sow farm |
| Laibin Xinhao | Sow farm | Laibin Xinhao | Sow farm | Jiaxiang Xinliu | Sow farm |
| Hezhou Xinhao | Sow farm | Yingcheng Xinhao | Sow farm | Zhaoqing Xinhao | Sow farm |
| Shibing Xinliu | Sow farm | Zhangjiakou New Hope | Sow farm | Zhangjiakou New Hope | Sow farm |
| Shuozhou Xinhao | Sow farm | Gansu Xinliu | Sow farm | Caoxian Xinhao | Sow farm |
| Dong'e Xinliu | Sow farm | Laibin Xinhao | Sow farm | Hainan Xinliu | Sow farm |
| Juye Xinhao | Sow farm | Kangping New Hope | Sow farm | Yingcheng Xinhao | Sow farm |
| Tangshan Xinhao | Sow farm | Caoxian Xinhao | Sow farm | Liaoning New Hope | Sow farm |
| Dezhou Xinhao | Sow farm | Liaoning New Hope | Sow farm | Ningming Xinhao | Sow farm |
| Guangdong Xinhao | Sow farm | Nanning Xinhao | Sow farm | Xiangzhou Xinhao | Sow farm |
| Yingcheng Xinhao | Sow farm | Guangdong Xinhao | Sow farm | Wuhe Xinhai | Sow farm |
| Tangshan Xinhao | Sow farm | Hezhou Xinhao | Sow farm | Jiaxiang Xinliu | Sow farm |
| Nanchong Xinhao | Fattening farm | Shuozhou Xinhao | Sow farm | Kangping New Hope | Sow farm |
| Juye Xinhao | Sow farm | Caoxian Xinhao | Sow farm | Zhaoqing Xinhao | Sow farm |
| Hubei Xinhao | Sow farm | Zhenjiang Xinhai | Sow farm | Ningming Xinhao | Sow farm |
| Zhangwu New Hope | Fattening farm | Hezhou Xinhao | Sow farm | Zhu Cheng Xinliu | Sow farm |
| Guangdong Xinhao | Sow farm | Ningming Xinhao | Sow farm | Shuozhou Xinhao | Sow farm |
| Shuozhou Xinhao | Sow farm | Hezhou Xinhao | Sow farm | Guigang Xinliu | Sow farm |
| Nanning Xinhao | Sow farm | Guangdong Xinhao | Sow farm | Dong'e Xinliu | Sow farm |
| Yingcheng Xinhao | Sow farm | Shuozhou Xinhao | Sow farm | Xiangzhou Xinhao | Sow farm |
| Laibin Xinhao | Sow farm | Bijie Xinliu | Sow farm | Wuhe Xinhai | Sow farm |
| Guangdong Xinhao | Sow farm | Baiyin Agriculture and Animal Husbandry | Sow farm | Shibing Xinliu | Sow farm |
| Baiyin Agriculture | Sow farm | Nanning Xinhao | Sow farm | Caoxian Xinhao | Sow farm |
| Santai Agriculture | Sow farm | Guangdong Xinhao | Sow farm | Wuhe Xinhai | Sow farm |
| Bijie Xinliu | Sow farm | Heyang Xinliu | Sow farm | Ningming Xinhao | Sow farm |
| Hezhou Xinhao | Sow farm | Dong'e Xinliu | Sow farm | Caoxian Xinhao | Sow farm |
| Hezhou Xinhao | Sow farm | Ruyuan Xinhao | Sow farm | Yichun Xinwang | Sow farm |
| Leshan Agriculture and Animal Husbandry | Sow farm | Leshan Nongmu | Sow farm | Weinan Xinliu | Sow farm |
| Hengshui Agriculture and Animal Husbandry | Sow farm | Wuhe Xinha | Sow farm | Pingfu Nongmu | Sow farm |
| Laibin Xinhao | Sow farm | Heishan Xinliu | Sow farm | Pingfu Nongmu | Sow farm |
| Kangping Xinwang | Sow farm | Wuqi Xinliu | Sow farm | Zhaoqing Xinhao | Sow farm |
| Qinghua Agriculture and Animal Husbandry | Sow farm | Lingxin Gaoxinliu | Sow farm | Shibing Xinliu | Sow farm |
| Taian Xinliu | Sow farm | Yijun Xinliu | Sow farm | Xiangzhou Xinhao | Sow farm |
| Guangdong Xinhao | Sow farm | Nanning Xinliu | Sow farm | Yan'an Benyuan | Sow farm |
| Ningming Xinhao | Sow farm | Shuozhou Xinhao | Sow farm | Shibing Xinliu | Sow farm |
| Hainan Xinliu | Sow farm | Xiangzhou Xinhao | Sow farm | Santai Nongmu | Sow farm |
| Xiangzhou Xinhao | Sow farm | Ningming Xinhao | Sow farm | Zhaoqing Xinhao | Sow farm |
| Lingao Xinliu | Sow farm | Baiyin Nongmu | Sow farm | Pingfu Nongmu | Sow farm |
| Yichun Xinwang | Sow farm | Guangdong Xinhao | Sow farm | Shibing Xinliu | Sow farm |
| Liaoning Xinwang | Sow farm | Baiyin Nongmu | Sow farm | Xiangzhou Xinhao | Sow farm |
| Ningming Xinhao | Sow farm | Wuhe Xinha | Sow farm | Shibing Xinliu | Sow farm |
| Liaoning Xinwang | Sow farm | Yingcheng Xinha | Sow farm | Leshan Nongmu | Sow farm |
| Laibin Xinhao | Sow farm | Liaoning Xinwang | Sow farm | Xiajiang Xinliu | Sow farm |
| Ningming Xinhao | Sow farm | Ningming Xinhao | Sow farm | Pingfu Nongmu | Sow farm |
| Weinan Xinliu | Sow farm | Liaoning Xinwang | Sow farm | Yan'an Benyuan | Sow farm |
| Hainan Xinliu | Sow farm | Kangping Xinwang | Sow farm | Yan'an Benyuan | Sow farm |
| Laibin Xinhao | Sow farm | Kangping Xinwang | Sow farm | Hezhou Xinhao | Sow farm |
| Shuozhou Xinhao | Sow farm | Hainan Xinliu | Sow farm | Leshan Nongmu | Sow farm |
| Nanning Xinliu | Sow farm | Yijun Xinliu | Sow farm | Hubei Xinhao | Sow farm |
| Shibing Xinliu | Sow farm | Liaoning Xinwang | Sow farm | Yanting Xinhao | Sow farm |
| Laibin Xinhao | Sow farm | Laibin Xinhao | Sow farm | Zhejiang Xinhai | Sow farm |
| Liaoning Xinwang | Sow farm | Dong'e Xinliu | Sow farm | Yanting Xinhao | Sow farm |
| Zhangwu Xinwang | Sow farm | Ningming Xinhao | Sow farm | Laiyang Xinmu | Sow farm |
| Liaoning Xinwang | Sow farm | Guanling Xinha | Sow farm | Qinghua Nongmu | Sow farm |
| Chenzhou Xinhao | Sow farm | Nanning Xinliu | Sow farm | Ningming Xinhao | Sow farm |
| Xiangzhou Xinhao | Sow farm | Yichun Xinwang | Sow farm | Yanting Xinhao | Sow farm |
| Wuhe Xinhai | Sow farm | Weinan Xinliu | Sow farm | Yingtan Xinliu | Sow farm |
| Weinan Xinliu | Sow farm | Laiyang Xinha | Sow farm | Laiyang Xinmu | Sow farm |
| Heyang Xinliu | Sow farm | Yijun Xinliu | Sow farm |  |  |
| Nanning Xinliu | Sow farm | Ningming Xinhao | Sow farm |  |  |
| Bijie Xinliu | Sow farm | Hubei Xinhao | Sow farm |  |  |
| Tangshan Xinhao | Sow farm | Kangping Xinwang | Sow farm |  |  |
| Taian Xinliu | Sow farm | Weinan Xinliu | Sow farm |  |  |
| Guangan Xinhao | Sow farm | Shibing Xinliu | Sow farm |  |  |
| Liaoning Xinwang | Sow farm | Xiangzhou Xinhao | Sow farm |  |  |
| Yijun Xinliu | Sow farm | Hezhou Xinhao | Sow farm |  |  |
| Lingao Xinliu | Sow farm | Baiyin Nongmu | Sow farm |  |  |
| Wuhe Xinhai | Sow farm | Changge Xincheng | Sow farm |  |  |
| Dong'e Xinliu | Sow farm | Shibing Xinliu | Sow farm |  |  |
| Wuqi Xinliu | Sow farm | Zhaoqing Xinhao | Sow farm |  |  |
| Ningming Xinhao | Sow farm | Hubei Xinhao | Sow farm |  |  |
| Yijun Xinliu | Sow farm | Hubei Xinhao | Sow farm |  |  |
| Laibin Xinhao | Sow farm | Shibing Xinliu | Sow farm |  |  |
| Jiaxiang Xinliu | Sow farm | Liaoning Xinwang | Sow farm |  |  |
| Kangping Xinwang | Sow farm | Xiangzhou Xinhao | Sow farm |  |  |
| Kangping Xinwang | Sow farm | Jiaxiang Xinliu | Sow farm |  |  |
| Liaoning Xinwang | Sow farm | Zhaoqing Xinhao | Sow farm |  |  |
| Shibing Xinliu | Sow farm | Shibing Xinliu | Sow farm |  |  |
| Hubei Xinhao | Sow farm | Kangping Xinwang | Sow farm |  |  |
| Baiyin Agriculture and Animal Husbandry | Sow farm | Yan'an Benyuan | Sow farm |  |  |
| Yichun Xinwang | Sow farm | Pingfu Nongmu | Sow farm |  |  |
| Yijun Xinliu | Sow farm | Anyue Xinxinxin | Sow farm |  |  |
| Laiyang Xinhao | Sow farm |  |  |  |  |
| Pengshui Xinliu | Sow farm |  |  |  |  |
| Ningming Xinhao | Sow farm |  |  |  |  |
| Kangping Xinwang | Sow farm |  |  |  |  |
| Shibing Xinliu | Sow farm |  |  |  |  |
| Hubei Xinhao | Sow farm |  |  |  |  |
| Liaoning Xinwang | Sow farm |  |  |  |  |
| Hubei Xinhao | Sow farm |  |  |  |  |
| Laiyang Xinmu | Sow farm |  |  |  |  |
| Shibing Xinliu | Sow farm |  |  |  |  |
| Pingfu Agriculture and Animal Husbandry | Sow farm |  |  |  |  |
| Santai Agriculture and Animal Husbandry | Sow farm |  |  |  |  |
| Zhaoqing Xinhao | Sow farm |  |  |  |  |
| Zhaoqing Xinhao | Sow farm |  |  |  |  |
| Laiyang Xinmu | Sow farm |  |  |  |  |
| Xiangzhou Xinhao | Sow farm |  |  |  |  |
| Xiangzhou Xinhao | Sow farm |  |  |  |  |
| Zhaoqing Xinhao | Sow farm |  |  |  |  |
| Yan'an Benyuan | Sow farm |  |  |  |  |
| Zhenjiang Xinhai | Sow farm |  |  |  |  |
| Pingfu Agriculture and Animal Husbandry | Sow farm |  |  |  |  |
| Hubei Xinhao | Sow farm |  |  |  |  |
| Laixi Xinliu | Sow farm |  |  |  |  |
| Xiangzhou Xinhao | Sow farm |  |  |  |  |
| Pingfu Agriculture and Animal Husbandry | Sow farm |  |  |  |  |
| Yan'an Benyuan | Sow farm |  |  |  |  |
| Zhangwu Xinwang | Sow farm |  |  |  |  |
| Huaiyin Xinchi | Sow farm |  |  |  |  |
| **Oct 2021** | | **Nov 2021** | | **Dec 2021** | |
| **Company (Co., Ltd.)** | **Type of pig farm** | **Company (Co., Ltd.)** | **Type of pig farm** | **Company (Co., Ltd.)** | **Type of pig farm** |
| Luocheng Xin Hao | Sow farm | Tongliao Xinhao | Sow farm | Guangan Xinhao | Fattening Farm |
| Gaomi Xinliu | Fattening farm | Laixi Xinliu | Fattening farm | Changyi Xinhao | Fattening Farm |
| Taian Xin Liu | Fattening farm | Gansu Xinhao | Sow farm | Changge Xincheng | Sow farm |
| Lingxin Xin Liu | Fattening farm | Longhui Xinhai | Sow farm | Guangan Xinhao | Fattening Farm |
| Taian Xin Liu | Fattening farm | Longhui Xinhai | Sow farm | Ningbo Xinhai | Sow farm |
| Tianjin Agriculture and Animal Husbandry | Sow farm | Guangdong Xinhao | Sow farm | Puyang Xinliu | Fattening Farm |
| Lingxin Xin Liu | Sow farm | Taian Xinliu | Fattening farm | Taian Xinliu | Fattening Farm |
| Nanning Xin Hao | Sow farm | Shuozhou Xinhao | Fattening farm | Wuming Xinliu | Sow farm |
| Tongliao Xin Hao | Sow farm | Sichuan Xinhao | Sow farm | Gansu Xinhao | Sow farm |
| Langzhong Xin Liu | Fattening farm | Shanxian Xinhao | Sow farm | Gansu Xinhao | Sow farm |
| Taian Xin Liu | Fattening farm | Guangan Xinhao | Fattening farm | Laiyang Xinmu | Fattening Farm |
| Tianjin Agriculture and Animal Husbandry | Sow farm | Xiajiang Xinliu | Sow farm | Guigang Xinliu | Fattening Farm |
| Rugao Xin Hao | Fattening farm | Tongliao Xinhao | Sow farm | Gaotang Xinhao | Sow farm |
| Pingfu Agriculture and Animal Husbandry | Sow farm | Sichuan Xinhao | Sow farm | Sichuan Xinhao | Sow farm |
| Dongying Xin Hao | Sow farm | Guangan Xinhao | Fattening farm | Tongliao Xinhao | Sow farm |
| Xingren Xin Liu | Sow farm | Gaotang Xinhao | Sow farm | Shanxian Xinliu | Fattening Farm |
| Jian Xin Chi | Sow farm | Jian New Run | Sow farm | Dong'e Xinliu | Fattening Farm |
| Guanling Xin Hai | Sow farm | Fuxin New Hope | Fattening farm | Xiajiang Xinliu | Sow farm |
| Ningbo Xin Hai | Sow farm | HeishanWoniu | Sow farm | Sichuan Xinhao | Sow farm |
| Dongying Xin Hao | Sow farm | Pingfu Livestock and Agriculture | Sow farm | Shuozhou Xinhao | Sow farm |
| Dongying Xin Hao | Sow farm | Beijing Xinliu | Sow farm | Tongliao Xinhao | Sow farm |
| Anyang Xin Liu | Sow farm | Ningbo Xinhai | Sow farm | Tongliao Xinhao | Sow farm |
| Longzhou Xin Hao | Fattening farm | HeishanWoniu | Sow farm | Yingtan Xinliu | Sow farm |
| Guangdong Xin Hao | Sow farm | Tongliao Xinhao | Sow farm | Heishan Woniu | Sow farm |
| Gaotang Xin Hao | Sow farm | Guangan Xinhao | Fattening farm | Dong'e Xinliu | Sow farm |
| Dongying Xin Hao | Sow farm | Shenze Xinhao | Sow farm | Beijing Xinliu | Sow farm |
| Gansu Xin Hao | Sow farm | Guanling Xinhai | Sow farm | Jian Xinchi | Sow farm |
| Liaoning Xin Wang | Sow farm | Beijing Xinliu | Sow farm | Heishan Woniu | Sow farm |
| Shanxian Xin Liu | Fattening farm | Tongliao Xinhao | Sow farm | Guangan Xinhao | Fattening Farm |
| Dingzhou Xin Hao | Sow farm | Shenze Xinhao | Sow farm | Hengnan Muyun | Sow farm |
| Wulian Xin Hao | Sow farm | Heishan Xinliu | Sow farm | Pingfu Nongmu | Sow farm |
| Jian Xin Chi | Fattening farm | Shenze Xinhao | Sow farm | Tongliao Xinhao | Sow farm |
| Pingfu Agriculture and Animal Husbandry | Sow farm | Gansu Xinhao | Sow farm | Guanling Xinhai | Sow farm |
| Zhenjiang Xin Hai | Sow farm | Guangan Xinhao | Fattening farm | Shenze Xinhao | Sow farm |
| Zhangjiakou Xin Wang | Sow farm | Sichuan Xinhao | Sow farm | Heishan Xinliu | Sow farm |
| Guigang Xin Liu | Sow farm | Hengnan Animal Husbandry | Sow farm | Guigang Xinliu | Sow farm |
| Anyang Xin Liu | Sow farm | Gansu Xinhao | Sow farm | Dongying Xinhao | Fattening Farm |
| Tongliao Xin Hao | Fattening farm | Zhangjiakou New Hope | Sow farm | Shenze Xinhao | Sow farm |
| Tianjin Xin Liu | Fattening farm | Laiyang Xinmu | Fattening farm | Sichuan Xinhao | Sow farm |
| Heishan Woniou | Sow farm | Shuozhou Xinhao | Sow farm | Gaotang Xinhao | Sow farm |
| Linyi Breeding Pig | Sow farm | Shenze Xinhao | Sow farm | Beijing Xinliu | Sow farm |
| Luocheng Xin Hao | Sow farm | Tongliao Xinhao | Sow farm | Hainan Nongken | Sow farm |
| Luocheng Xin Hao | Sow farm | Dongying Xinhao | Fattening farm | Anyang Xinliu | Sow farm |
| Nanchong Xin Hao | Fattening farm | Yingtan Xinliu | Sow farm | Guangan Xinhao | Sow farm |
| Pengshui Xin Liu | Fattening farm | Langzhong Xinliu | Sow farm | Tianjin Nongmu | Sow farm |
| Shibing Xin Liu | Sow farm | Raoyang Xinhao | Sow farm | Shenze Xinhao | Sow farm |
| Linyi Breeding Pig | Sow farm | Guangan Xinhao | Sow farm | Shanxian Xinliu | Sow farm |
| Sichuan Xin Hao | Sow farm | Hainan Agriculture and Reclamation | Sow farm | Hainan Xinliu | Sow farm |
| Shenze Xin Hao | Sow farm | Shanxian Xinliu | Sow farm | Anhui Xinliu | Sow farm |
| Heishan Xin Liu | Sow farm | Xindongwan Xinliu | Sow farm | Shenze Xinhao | Sow farm |
| Beijing Xin Liu | Sow farm | Shenze Xinhao | Sow farm | Laixi Xinliu | Sow farm |
| Shanxian Xin Liu | Fattening farm | Laiyang Xinmu | Sow farm | Shuozhou Xinhao | Sow farm |
| Jiexi Xin Liu | Fattening farm | Hainan Xinliu | Sow farm | Gaotang Xinhao | Sow farm |
| Heishan Woniou | Sow farm | Pingfu Livestock and Agriculture | Sow farm | Zhejiang Xinhai | Sow farm |
| Longhui Xin Hai | Sow farm | Sichuan Xinhao | Sow farm | Xiangyang Xinhao | Sow farm |
| Tongliao Xin Hao | Sow farm | Sichuan Xinhao | Sow farm | Wuqi Xinliu | Sow farm |
| Langzhong Xin Liu | Sow farm | Laixi Xinliu | Sow farm | Raoyang Xinhao | Sow farm |
| Luocheng Xin Hao | Sow farm | Laizhou Xinhai | Sow farm | Langzhong Xinliu | Sow farm |
| Shanxian Xin Hao | Sow farm | Zhejiang Xinhai | Sow farm | Laizhou Xinhai | Sow farm |
| Weinan Xin Liu | Sow farm | Wuqi Xinliu | Sow farm | Guangan Xinhao | Fattening Farm |
| Tianjin Agriculture and Animal Husbandry | Sow farm | Gaotang Xinhao | Sow farm | Gansu Xinliu | Sow farm |
| Gaotang Xin Hao | Sow farm | Gansu Xinliu | Sow farm | Laizhou Xinhai | Sow farm |
| Weinan Xin Liu | Sow farm | Xiangyang Xinhao | Sow farm | Huaiyin Xincheng | Sow farm |
| Raoyang Xin Hao | Sow farm | Huaiyin New Run | Sow farm | Zhangjiakou Xinwang | Sow farm |
| Sichuan Xin Hao | Sow farm | Anyang Xinliu | Sow farm | Shenze Xinhao | Sow farm |
| Longhui Xin Hai | Sow farm | Laizhou Xinhai | Sow farm | Dongying Xinhao | Sow farm |
| Raoyang Xin Hao | Sow farm | Dongying Xinhao | Sow farm | Suining Xinliu | Sow farm |
| Guigang Xin Liu | Sow farm | Laixi Xinliu | Sow farm | Yingtan Xinliu | Sow farm |
| Zhenjiang Xin Hai | Sow farm | Shenze Xinhao | Sow farm | Dongying Xinhao | Sow farm |
| Luocheng Xin Hao | Sow farm | Rongchang Pig Farm | Sow farm | Jian Xinchi | Sow farm |
| Bijie Xin Liu | Fattening farm | Shanxian Xinhao | Sow farm | Huanghua Xinhao | Sow farm |
| Xiajin Agriculture and Animal Husbandry | Sow farm | Weinan Xinliu | Sow farm | Meishan Xinhai | Sow farm |
| Xiajin Agriculture and Animal Husbandry | Sow farm | Suining Xinliu | Sow farm | Guangan Xinhao | Sow farm |
| Qingyuan Xin Hao | Fattening farm | Jian New Run | Sow farm | Rongchang Pig Farm | Sow farm |
| Xiajin Agriculture and Animal Husbandry | Sow farm | Meishan Xinhai | Sow farm | Xindongwan Xinliu | Sow farm |
| Tongliao Xin Hao | Sow farm | Wuhe Xinhai | Sow farm | Jiangxi Xinliu | Sow farm |
| Sichuan Xin Hao | Sow farm | Dongying Xinhao | Sow farm | Liaoning Xinwang | Sow farm |
| Shenze Xin Hao | Sow farm | Changge New Run | Sow farm | Xiangyang Xinhao | Sow farm |
| Henan Agriculture and Animal Husbandry | Sow farm | Jiangxi Xinliu | Sow farm | Laiyang Xinhao | Sow farm |
| Nanchong Xin Hao | Sow farm | Yingtan Xinliu | Sow farm | Xiangyang Xinhao | Sow farm |
| Xiajin Agriculture and Animal Husbandry | Sow farm | Laiyang Xinmu | Sow farm | Shanxian Xinhao | Sow farm |
| Shanxian Xin Hao | Sow farm | Guigang Xinliu | Fattening farm | Wuhe Xinhai | Sow farm |
| Xiajin Agriculture and Animal Husbandry | Sow farm | Lingbao Xinliu | Sow farm | Meishan Xinhai | Sow farm |
| Guang'an Xin Hao | Sow farm | Puyang Xinliu | Fattening farm | Laixi Xinliu | Sow farm |
| Zhenjiang Xin Hai | Sow farm | Raoyang Xinhao | Sow farm | Shanxian Xinhao | Sow farm |
| Xiajin Agriculture and Animal Husbandry | Sow farm | Shanxian Xinhao | Sow farm | Shenze Xinhao | Sow farm |
| Xiajin Agriculture and Animal Husbandry | Sow farm | Shanxian Xinhao | Sow farm | Liaoning Xintao Wang | Sow farm |
| Guang'an Xin Hao | Fattening farm | Tianjin Agriculture and Animal Husbandry | Sow farm | Xiangyang Xinhao | Sow farm |
| Shuozhou Xin Hao | Fattening farm | Linyi Breeding Pig | Sow farm | Weinan Xinliu | Sow farm |
| Hengnan Muyun | Sow farm | Anhui Xinliu | Sow farm | Lingbao Xinliu | Sow farm |
| Laixi Xin Liu | Sow farm | Tianjin Agriculture and Animal Husbandry | Sow farm | Huanghua Xinhao | Sow farm |
| Shanxian Xin Hao | Sow farm | Huanghua Xinhao | Sow farm | Laizhou Xinhai | Sow farm |
| Qingfeng Xin Liu | Sow farm | Huanghua Xinhao | Sow farm | Tianjin Nongmu | Sow farm |
| Laiyang Xinmu | Fattening farm | Xiajin Agriculture and Animal Husbandry | Sow farm | Shanxian Xinhao | Sow farm |
| Shenze Xin Hao | Sow farm | Tianjin Agriculture and Animal Husbandry | Sow farm | Tianjin Nongmu | Sow farm |
| Meishan Xin Hai | Sow farm | Xiangyang Xinhao | Sow farm | Yichun Xinwang | Sow farm |
| Huanghua Xin Hao | Sow farm | Xiajin Agriculture and Animal Husbandry | Sow farm | Yinbao Breeding | Sow farm |
| Huanghua Xin Hao | Sow farm | Xiajin Agriculture and Animal Husbandry | Sow farm | Tongliao Xinhao | Sow farm |
| Lingbao Xin Liu | Sow farm | Xiajin Agriculture and Animal Husbandry | Sow farm | Kangping Xinwang | Sow farm |
| Sichuan Xin Hao | Sow farm | Tianjin Agriculture and Animal Husbandry | Sow farm | Tongliao Xinhao | Sow farm |
| Laizhou Xin Hai | Sow farm | Xiangyang Xinhao | Sow farm | Longzhou Xinhao | Sow farm |
| Guang'an Xin Hao | Fattening farm | Xiajin Agriculture and Animal Husbandry | Sow farm | Tianjin Nongmu | Sow farm |
| Huanghua Xin Hao | Sow farm | Raoyang Xinhao | Sow farm | Laizhou Xinhai | Sow farm |
| Huanghua Xin Hao | Sow farm | Xiajin Agriculture and Animal Husbandry | Sow farm | Kangping Xinwang | Sow farm |
| Weinan Xin Liu | Sow farm | Xiajin Agriculture and Animal Husbandry | Sow farm | Taian Xinliu | Sow farm |
| Hainan Xin Liu | Sow farm | Xiangyang Xinhao | Sow farm | Heishan Xinliu | Sow farm |
| Gansu Xin Hao | Sow farm | Liaoning New Taowang | Sow farm | Jian Xinchi | Sow farm |
| Xindongwan Xin Liu | Sow farm | Jian New Run | Sow farm | Laizhou Xinhai | Sow farm |
| Longhui Xin Hai | Fattening farm | Huanghua Xinhao | Sow farm | Jian Xinchi | Sow farm |
| Hainan Agriculture and Reclamation | Sow farm | Huanghua Xinhao | Sow farm | Liaoning Xintao Wang | Sow farm |
| Sichuan Xin Hao | Sow farm | Jian New Run | Sow farm | Dongying Xinhao | Sow farm |
| Huaiyin Xin Chi | Sow farm | Huanghua Xinhao | Sow farm | Laizhou Xinhai | Sow farm |
| Laixi Xin Liu | Sow farm | Tianjin Agriculture and Animal Husbandry | Sow farm | Donghai Xinhao | Sow farm |
| Gaotang Xin Hao | Sow farm | Shanxian Xinhao | Sow farm | Santai Nongmu | Sow farm |
| Shuozhou Xin Hao | Sow farm | Yinbao Breeding | Sow farm | Raoyang Xinhao | Sow farm |
| Rongchang Pig Farm | Sow farm | Tongliao Xinhao | Sow farm | Raoyang Xinhao | Sow farm |
| Guang'an Xin Hao | Fattening farm | Liaoning New Taowang | Sow farm | Yantai Xinhao | Sow farm |
| Tongliao Xin Hao | Sow farm | Yichun New Hope | Sow farm | Fuxin Xinwang | Sow farm |
| Gansu Xin Liu | Sow farm | Tongliao Xinhao | Sow farm | Laizhou Xinhai | Sow farm |
| Huanghua Xin Hao | Sow farm | Taian Xinliu | Sow farm | Pingfu Nongmu | Sow farm |
| Wuhe Xin Hai | Sow farm | Tongliao Xinhao | Sow farm | Yantai Xinhao | Sow farm |
| Weinan Xin Liu | Sow farm | Kangping New Hope | Sow farm | Yantai Xinhao | Sow farm |
| Laizhou Xin Hai | Sow farm | Xindongwan Xinliu | Sow farm | Shanxian Xinhao | Sow farm |
| Dongying Xin Hao | Sow farm | Laizhou Xinhai | Sow farm | Laizhou Xinhai | Sow farm |
| Xiajin Agriculture and Animal Husbandry | Sow farm | Laizhou Xinhai | Sow farm | Hubei Xinhao | Sow farm |
| Xiajin Agriculture and Animal Husbandry | Sow farm | Laizhou Xinhai | Sow farm | Wuhe Xinhai | Sow farm |
| Zhejiang Xin Hai | Sow farm | Jian New Run | Sow farm | Tianjin Nongmu | Sow farm |
| Shanxian Xin Hao | Sow farm | Laizhou Xinhai | Sow farm | Gao Mi Xinliu | Sow farm |
| Raoyang Xin Hao | Sow farm | Laizhou Xinhai | Sow farm | Longzhou Xinhao | Sow farm |
| Wuqi Xin Liu | Sow farm | Fuxin New Hope | Sow farm | Leshan Nongmu | Sow farm |
| Dongying Xin Hao | Sow farm | Longzhou Xinhao | Sow farm | Laizhou Xinhai | Sow farm |
| Shanxian Xin Liu | Sow farm | Kangping New Hope | Sow farm | Longzhou Xinhao | Sow farm |
| Suining Xin Liu | Sow farm | Gaomi Xinliu | Sow farm | Gansu Xinhao | Sow farm |
| Jian Xin Chi | Sow farm | Laizhou Xinhai | Sow farm | Liaocheng Xinhao | Sow farm |
| Xiajin Agriculture and Animal Husbandry | Sow farm | Xiajin Agriculture and Animal Husbandry | Sow farm | Longzhou Xinhao | Sow farm |
| Xiajin Agriculture and Animal Husbandry | Sow farm | Jiaxiang Xinliu | Sow farm | Sichuan Xinhao | Sow farm |
| Guang'an Xin Hao | Fattening farm | Xiajin Agriculture and Animal Husbandry | Sow farm | Shibing Xinliu | Sow farm |
| Shanxian Xin Hao | Sow farm | Qinghua Agriculture and Animal Husbandry | Sow farm | Huanghua Xinhao | Sow farm |
| Tianjin Agriculture and Animal Husbandry | Sow farm | Laiyang Xinhao | Sow farm | Liangshan Xinliu | Sow farm |
| Xiangyang Xin Hao | Sow farm | Hubei Xinhao | Sow farm | Yantai Xinhao | Sow farm |
| Tianjin Agriculture and Animal Husbandry | Sow farm | Xiajin Agriculture and Animal Husbandry | Sow farm | Qinghua Nongmu | Sow farm |
| Xingren Xin Liu | Sow farm | Liaocheng Xinhao | Sow farm | Longzhou Xinhao | Sow farm |
| Jiangxi Xin Liu | Sow farm | Gansu Xinhao | Sow farm | Dongying Xinhao | Sow farm |
| Gansu Xin Hao | Sow farm | Liangshan Xinliu | Sow farm | Yantai Xinhao | Sow farm |
| Anhui Xin Liu | Sow farm | Shibing Xinliu | Sow farm | Guangdong Xinhao | Sow farm |
| Guigang Xin Liu | Fattening farm | Yantai Xinhao | Sow farm | Dongying Xinhao | Sow farm |
| Beijing Xin Liu | Sow farm | Laizhou Xinhai | Sow farm | Dongying Xinhao | Sow farm |
| Jian Xin Chi | Sow farm | Xiajin Agriculture and Animal Husbandry | Sow farm | Longzhou Xinhao | Sow farm |
| Liaoning Xintao Wang | Sow farm | Santai Agriculture and Animal Husbandry | Sow farm | Pingfu Nongmu | Sow farm |
| Wuhe Xin Hai | Sow farm | Yantai Xinhao | Sow farm | Jiaxiang Xinliu | Sow farm |
| Laizhou Xin Hai | Sow farm | Pingfu Livestock and Agriculture | Sow farm | Laizhou Xinhai | Sow farm |
| Leshan Agriculture and Animal Husbandry | Sow farm | Yantai Xinhao | Sow farm | Hezhou Xinhao | Sow farm |
| Yichun Xintao Wang | Sow farm | Yantai Xinhao | Sow farm | Yan'an Benyuan | Sow farm |
| Liaoning Xintao Wang | Sow farm | Yantai Xinhao | Sow farm | Caoxian Xinhao | Sow farm |
| Jian Xin Chi | Sow farm | Dongying Xinhao | Sow farm | Yantai Xinhao | Sow farm |
| Wuhe Xin Hai | Sow farm | Laizhou Xinhai | Sow farm | Xiangyang Xinhao | Sow farm |
| Wuhe Xin Hai | Sow farm | Wuhe Xinhai | Sow farm | Longzhou Xinhao | Sow farm |
| Wuhe Xin Hai | Sow farm | Longzhou Xinhao | Sow farm | Yantai Xinhao | Sow farm |
| Fuxin Xintao Wang | Fattening farm | Caoxian Xinhao | Sow farm | Dongying Xinhao | Sow farm |
| Taian Xin Liu | Sow farm | Longzhou Xinhao | Sow farm | Yantai Xinhao | Sow farm |
| Xingren Xin Liu | Sow farm | Guangan Xinhao | Sow farm | Dongying Xinhao | Sow farm |
| Xiangyang New Hope | Sow farm | Dongying Xinhao | Sow farm | Yantai Xinhao | Sow farm |
| Yinbao Breeding | Sow farm | Yantai Xinhao | Sow farm | Dongying Xinhao | Sow farm |
| Kangping Xinwang | Sow farm | Longzhou Xinhao | Sow farm | Xiajin Agro-Pastoral | Sow farm |
| Fuxin Xinwang | Sow farm | Wuhe Xinhai | Sow farm | Ruyuan Xinhao | Sow farm |
| Anyang Xinliu | Sow farm | Longzhou Xinhao | Sow farm | Xiajin Agro-Pastoral | Sow farm |
| Laizhou Xinhai | Sow farm | Yantai Xinhao | Sow farm | Xiajin Agro-Pastoral | Sow farm |
| Laizhou Xinhai | Sow farm | Dongying Xinhao | Sow farm | Zhongshan Agro-Pastoral | Sow farm |
| Gaomi Xinliu | Sow farm | Yantai Xinhao | Sow farm | Guangyuan Xinhao | Sow farm |
| Xiangyang New Hope | Sow farm | Pucheng Xinliu | Sow farm | Nanning Xinhao | Sow farm |
| Laizhou Xinhai | Sow farm | Longzhou Xinhao | Sow farm | Xiajin Agro-Pastoral | Sow farm |
| Laizhou Xinhai | Sow farm | Hezhou Xinhao | Sow farm | Guigang Xinliu | Sow farm |
| Longzhou New Hope | Sow farm | Dongying Xinhao | Sow farm | Tongliao Xinhao | Sow farm |
| Liaocheng New Hope | Sow farm | Dongying Xinhao | Sow farm | Xiajin Agro-Pastoral | Sow farm |
| Gansu New Hope | Sow farm | Yantai Xinhao | Sow farm | Pucheng Xinliu | Sow farm |
| Laizhou Xinhai | Sow farm | Leshan Agriculture and Animal Husbandry | Sow farm | Dongying Xinhao | Sow farm |
| Laizhou Xinhai | Sow farm | Longzhou Xinhao | Sow farm | Xiajin Agro-Pastoral | Sow farm |
| Yantai New Hope | Sow farm | Xiangyang Xinhao | Sow farm | Xiajin Agro-Pastoral | Sow farm |
| Qinghua Agriculture and Animal Husbandry | Sow farm | Wuhe Xinhai | Sow farm | Xiajin Agro-Pastoral | Sow farm |
| Laiyang New Hope | Sow farm | Wuhe Xinhai | Sow farm | Taian Xinliu | Sow farm |
| Leshan Agriculture and Animal Husbandry | Sow farm | Yan'an Benyuan | Sow farm | Nanning Xinliu | Sow farm |
| Shibing Xinliu | Sow farm | Dongying Xinhao | Sow farm | Huanghua Xinhao | Sow farm |
| Yantai New Hope | Sow farm | Guangdong Xinhao | Sow farm | Zhuocheng Xinliu | Sow farm |
| Yantai New Hope | Sow farm | Zhongshan Agriculture and Animal Husbandry | Sow farm | Xindongwan Xinliu | Sow farm |
| Anyue Xinxin | Sow farm | Dongying Xinhao | Sow farm | Juye Xinhao | Sow farm |
| Yantai New Hope | Sow farm | Wuhe Xinhai | Sow farm | Huanghua Xinhao | Sow farm |
| Santai Agriculture and Animal Husbandry | Sow farm | Juye Xinhao | Sow farm | Jiangyou Pig Farm | Sow farm |
| Yantai New Hope | Sow farm | Zhou Xinliu | Sow farm | Santai Agro-Pastoral | Sow farm |
| Liangshan Xinliu | Sow farm | Yingtan Xinliu | Sow farm | Chenzhou Xinhao | Sow farm |
| Juye New Hope | Sow farm | Liaoning New Taowang | Sow farm | Linyi Breeding Pig | Sow farm |
| Pingfu Agriculture and Animal Husbandry | Sow farm | Chenzhou Xinhao | Sow farm | Liaoning Xintao Wang | Sow farm |
| Yantai New Hope | Sow farm | Nanning Xinhao | Sow farm | Jingxian Xinhao | Sow farm |
| Xiangyang New Hope | Sow farm | Nanning Xinliu | Sow farm | Heishan Xinliu | Sow farm |
| Sichuan New Hope | Sow farm | Ruyuan Xinhao | Sow farm | Anyang Xinliu | Sow farm |
| Caoxian New Hope | Sow farm | Meishan Xinhai | Sow farm | Dong'e Xinliu | Sow farm |
| Longzhou New Hope | Sow farm | Shuozhou Xinhao | Sow farm | Laixi Xinliu | Sow farm |
| Xindongwan Xinliu | Sow farm | Heishan Xinliu | Sow farm | Dong'e Xinliu | Sow farm |
| Kangping Xinwang | Sow farm | Baiyin Agriculture and Animal Husbandry | Sow farm | Liaoning Xinwang | Sow farm |
| Jiaxiang Xinliu | Sow farm | Jingxian Xinhao | Sow farm | Yingtan Xinliu | Sow farm |
| Hubei New Hope | Sow farm | Jiangyou Pig Farm | Sow farm | Xiajin Agro-Pastoral | Sow farm |
| Longzhou New Hope | Sow farm | Heishan Xinliu | Sow farm | Wuhe Xinhai | Sow farm |
| Yantai New Hope | Sow farm | Dongying Xinhao | Sow farm | Heishan Xinliu | Sow farm |
| Yantai New Hope | Sow farm | Heishan Xinliu | Sow farm | Wuhe Xinhai | Sow farm |
| Yan'an Benyuan | Sow farm | Dong'a Xinliu | Sow farm | Xiajin Agro-Pastoral | Sow farm |
| Longzhou New Hope | Sow farm | Anyang Xinliu | Sow farm | Wuhe Xinhai | Sow farm |
| Dongying New Hope | Sow farm | Yingtan Xinliu | Sow farm | Wuhe Xinhai | Sow farm |
| Anyang Xinliu | Sow farm | Santai Agriculture and Animal Husbandry | Sow farm | Laixi Xinliu | Sow farm |
| Longzhou New Hope | Sow farm | Guangyuan Xinhao | Sow farm | Donghai Xinhao | Sow farm |
| Zhuocheng Xinliu | Sow farm | Heishan Xinliu | Sow farm | Liaoning Xinwang | Sow farm |
| Longzhou New Hope | Sow farm | Anyang Xinliu | Sow farm | Heishan Xinliu | Sow farm |
| Laizhou Xinhai | Sow farm | Liaoning New Taowang | Sow farm | Donghai Xinhao | Sow farm |
| Yantai New Hope | Sow farm | Dong'a Xinliu | Sow farm | Xiajin Agro-Pastoral | Sow farm |
| Dongying New Hope | Sow farm | Liaoning New Taowang | Sow farm | Juye Xinhao | Sow farm |
| Longzhou New Hope | Sow farm | Liaoning New Taowang | Sow farm | Laixi Xinliu | Sow farm |
| Pucheng Xinliu | Sow farm | Liaoning New Taowang | Sow farm | Fuxin Xinwang | Sow farm |
| Guigang Xinliu | Sow farm | Gansu Xinhao | Sow farm | Donghai Xinhao | Sow farm |
| Tongliao New Hope | Sow farm | Guigang Xinliu | Sow farm | Linyi Xinhao | Sow farm |
| Dongying New Hope | Sow farm | Liaoning Xinwang | Sow farm | Meishan Xinhai | Sow farm |
| Dongying New Hope | Sow farm | Fuxin Xinwang | Sow farm | Liaoning Xintao Wang | Sow farm |
| Liaoning Xintao Wang | Sow farm | Fuxin Xinwang | Sow farm | Meishan Xinhai | Sow farm |
| Tongliao New Hope | Sow farm | Guigang Xinliu | Sow farm | Taian Xinliu | Sow farm |
| Jingxian New Hope | Sow farm | Heishan Wolniu | Sow farm | Liaoning Xinwang | Sow farm |
| Dongying New Hope | Sow farm | Meishan Xinhai | Sow farm | Liaoning Xintao Wang | Sow farm |
| Shuozhou New Hope | Sow farm | Meishan Xinhai | Sow farm | Liaoning Xintao Wang | Sow farm |
| Hezhou New Hope | Sow farm | Hainan Agriculture and Forestry Reclamation | Sow farm | Liaoning Xinwang | Sow farm |
| Heishan Xinliu | Sow farm | Lingbao Xinliu | Sow farm | Liaoning Xinwang | Sow farm |
| Guang'an New Hope | Sow farm | Anyang Xinliu | Sow farm | Anyang Xinliu | Sow farm |
| Heishan Xinliu | Sow farm | Hainan Agriculture and Forestry Reclamation | Sow farm | Baiyin Agro-Pastoral | Sow farm |
| Zhongshan Agriculture and Animal Husbandry | Sow farm | Liaoning New Taowang | Sow farm | Liaoning Xinwang | Sow farm |
| Dongying New Hope | Sow farm | Guangan Xinhao | Sow farm | Fuxin Xinwang | Sow farm |
| Dongying New Hope | Sow farm | Liaoning Xinwang | Sow farm | Langzhong Xinliu | Sow farm |
| Jiangyou Pig Farm | Sow farm | Langzhong Xinliu | Sow farm | Liaoning Xinwang | Sow farm |
| Chenzhou New Hope | Sow farm | Heishan Xinliu | Sow farm | Shuozhou Xinhao | Sow farm |
| Ruyuan New Hope | Sow farm | Meishan Xinhai | Sow farm | Fuxin Xinwang | Sow farm |
| Dongying New Hope | Fattening farm | Lingao Xinliu | Sow farm | Linyi Xinhao | Sow farm |
| Anyang Xinliu | Sow farm | Liaoning Xinwang | Sow farm | Guangan Xinhao | Sow farm |
| Wuhe Xinhai | Sow farm | Shuozhou Xinhao | Sow farm | Suining Xinliu | Sow farm |
| Nanning New Hope | Sow farm | Dong'a Xinliu | Sow farm | Meishan Xinhai | Sow farm |
| Baiyin Agriculture and Animal Husbandry | Sow farm | Taopian Xinliu | Sow farm | Liaoning Xinwang | Sow farm |
| Laiyang Xinmu | Sow farm | Chenzhou Xinhao | Sow farm | Gansu Xinliu | Sow farm |
| Jian New Chi | Sow farm | Liaoning Xinwang | Sow farm | Liaoning Xintao Wang | Sow farm |
| Lingbao Xinliu | Sow farm | Juye Xinhao | Sow farm | Jiangxi Xinliu | Sow farm |
| Guangdong New Hope | Sow farm | Langzhong Xinliu | Sow farm | Liaoning Xinwang | Sow farm |
| Nanning Xinliu | Sow farm | Guigang Xinliu | Sow farm | Anyang Xinliu | Sow farm |
| Dongying New Hope | Sow farm | Nanning Xinhao | Sow farm | Dong'e Xinliu | Sow farm |
| Gansu New Hope | Sow farm | Jiangxi Xinliu | Sow farm | Jiangxi Xinliu | Sow farm |
| Guangyuan New Hope | Sow farm | Yingcheng Xinhao | Sow farm | Kangping Xinwang | Sow farm |
| Tongliao New Hope | Sow farm | Gansu Xinhao | Sow farm | Guigang Xinliu | Sow farm |
| Liaoning Xintao Wang | Sow farm | Fuxin Xinwang | Sow farm | Hainan State Farms | Sow farm |
| Heishan Woniux | Sow farm | Taopian Xinliu | Sow farm | Hubei Xinhao | Sow farm |
| Xiangyang New Hope | Sow farm | Ningming Xinhao | Sow farm | Heishan Xinliu | Sow farm |
| Liaoning Xintao Wang | Sow farm | Chenzhou Xinhao | Sow farm | Hainan State Farms | Sow farm |
| Changge New Chi | Sow farm | Jiangxi Xinliu | Sow farm | Yingtan Xinliu | Sow farm |
| Liaoning Xintao Wang | Sow farm | Liaoning Xinwang | Sow farm | Dong'e Xinliu | Sow farm |
| Dongying New Hope | Sow farm | Yucheng Xinliu | Sow farm | Liaoning Xinwang | Sow farm |
| Tongliao New Hope | Sow farm | Liaoning Xinwang | Sow farm | Leshan Agro-Pastoral | Sow farm |
| Liaoning Xintao Wang | Sow farm | Nanning Xinhao | Sow farm | Guangan Xinhao | Sow farm |
| Dongying New Hope | Sow farm | Ningming Xinhao | Sow farm | Yucheng Xinliu | Sow farm |
| Hainan State Farms | Sow farm | Liaoning Xinwang | Sow farm | Lingao Xinliu | Sow farm |
| Hainan State Farms | Sow farm | Hubei Xinhao | Sow farm | Baiyin Agro-Pastoral | Sow farm |
| Jian New Chi | Sow farm | Liaoning Xinwang | Sow farm | Liaoning Xinwang | Sow farm |
| Liaoning Xintao Wang | Sow farm | Shuozhou Xinhao | Sow farm | Jiangxi Xinliu | Sow farm |
| Langzhong Xinliu | Sow farm | Jiangxi Xinliu | Sow farm | Yingcheng Xinhao | Sow farm |
| Liaoning Xinwang | Sow farm | Liaoning Xinwang | Sow farm | Liaoning Xintao Wang | Sow farm |
| Santai Agriculture and Animal Husbandry | Sow farm | Nanning Xinhao | Sow farm | Heishan Woniu | Sow farm |
| Shuozhou New Hope | Sow farm | Heyang Xinliu | Sow farm | Lingao Xinliu | Sow farm |
| Danxian Xinliu | Sow farm | Hubei Xinhao | Sow farm | Hubei Xin Hao | Sow farm |
| Taian Xinliu | Sow farm | Chenzhou Xinhao | Sow farm | Gaotang Xinhao | Sow farm |
| Guang'an New Hope | Sow farm | Hubei Xinhao | Sow farm | Yingcheng Xinhao | Sow farm |
| Jiangxi Xinliu | Sow farm | Yucheng Xinliu | Sow farm | Laixi Xinliu | Sow farm |
| Heishan Xinliu | Sow farm | Ningming Xinhao | Sow farm | Xiangzhou Xinhao | Sow farm |
| Hubei New Hope | Sow farm | Taopian Xinliu | Sow farm | Juye Xinhao | Sow farm |
| Guigang Xinliu | Sow farm | Shuozhou Xinhao | Sow farm | Taian Xinliu | Sow farm |
| Chenzhou New Hope | Sow farm | Dongying Xinhao | Sow farm | Wuhe Xinhai | Sow farm |
| Lingbao Xinliu | Sow farm | Liaoning Xinwang | Sow farm | Hubei Xin Hao | Sow farm |
| Guang'an New Hope | Sow farm | Kangping Xinwang | Sow farm | Wulian Xin Hao | Sow farm |
| Heishan Xinliu | Sow farm | Jian New Chi | Sow farm | Suining Xinliu | Sow farm |
| Meishan Xinhai | Sow farm | Anhui Xinliu | Sow farm | Dong'e Xinliu | Sow farm |
| Meishan Xinhai | Sow farm | Gansu Xinliu | Sow farm | Liaocheng Xin Hao | Sow farm |
| Guangyuan New Hope | Sow farm | Pucheng Xinliu | Sow farm | Hubei Xin Hao | Sow farm |
| Yingcheng Xinhao | Sow farm | Hubei Xinhao | Sow farm | Chenzhou Xinhao | Sow farm |
| Taian Xinliu | Sow farm | Ningming Xinhao | Sow farm | Yichun Xinwang | Sow farm |
| Chenzhou Xinhao | Sow farm | Guangyuan Xinhao | Sow farm | Shuozhou Xinhao | Sow farm |
| Baiyin Agriculture and Animal Husbandry | Sow farm | Juye Xinhao | Sow farm | Heyang Xinliu | Sow farm |
| Nanning Xinhao | Sow farm | Guangdong Xinhao | Sow farm | Langzhong Xinliu | Sow farm |
| Meishan Xinhai | Sow farm | Baiyin Agriculture and Animal Husbandry | Sow farm | Nanning Xinhao | Sow farm |
| Taian Xinliu | Sow farm | Leshan Agriculture and Animal Husbandry | Sow farm | Nanning Xinhao | Sow farm |
| Wulian Xinhao | Sow farm | Lingxin New No. 6 | Sow farm | Yichun Xinwang | Sow farm |
| Fuxin Xinwang | Sow farm | Liaocheng Xinhao | Sow farm | Wuhe Xinhai | Sow farm |
| Lingxian Xinliu | Sow farm | Yichun Xinwang | Sow farm | Ningming Xinhao | Sow farm |
| Liaoning Xinwang | Sow farm | Liaoning Xinwang | Sow farm | Ningming Xinhao | Sow farm |
| Liaocheng Xinhao | Sow farm | Dong'e New No. 6 | Sow farm | Juye Xinhao | Sow farm |
| Yingtan Xinliu | Sow farm | Liaocheng Xinhao | Sow farm | Chenzhou Xinhao | Sow farm |
| Langzhong Xinliu | Sow farm | Gansu New No. 6 | Sow farm | Anhui Xinliu | Sow farm |
| Suining Xinliu | Sow farm | Shuozhou Xinhao | Sow farm | Hengnan Muyun | Sow farm |
| Liaoning Xinwang | Sow farm | Laixi New No. 6 | Sow farm | Wuhe Xinhai | Sow farm |
| Liaoning Xinwang | Sow farm | Yingcheng Xinhao | Sow farm | Gansu Xinhao | Sow farm |
| Pucheng Xinliu | Sow farm | Dong'e New No. 6 | Sow farm | Yucheng Xinliu | Sow farm |
| Laixi Xinliu | Sow farm | Kangping Xinwang | Sow farm | Gansu Xinhao | Sow farm |
| Yucheng Xinliu | Sow farm | Laixi New No. 6 | Sow farm | Guigang Xinliu | Sow farm |
| Gansu Xinhao | Sow farm | Juye Xinhao | Sow farm | Hengnan Muyun | Sow farm |
| Gansu Xinhao | Sow farm | Linyi Xinhao | Sow farm | Taian Xinliu | Sow farm |
| Jingxian Xinhao | Sow farm | Laibin Xinhao | Sow farm | Shuozhou Xinhao | Sow farm |
| Yucheng Xinliu | Sow farm | Guigang New No. 6 | Sow farm | Yingcheng Xinhao | Sow farm |
| Liaoning Xinwang | Sow farm | Suining New No. 6 | Sow farm | Wuqi Xinliu | Sow farm |
| Hubei Xinhao | Sow farm | Shuozhou Xinhao | Sow farm | Chenzhou Xinhao | Sow farm |
| Nanning Xinhao | Sow farm | Laibin Xinhao | Sow farm | Yingcheng Xinhao | Sow farm |
| Chenzhou Xinhao | Sow farm | Yingcheng Xinhao | Sow farm | Gansu Xinhao | Sow farm |
| Heishan Xinliu | Sow farm | Laibin Xinhao | Sow farm | Changyi Xinhao | Sow farm |
| Donghai Xinhao | Sow farm | Donghai Xinhao | Sow farm | Dongying Xinhao | Sow farm |
| Taian Xinliu | Sow farm | Linyi Xinhao | Sow farm | Hezhou Xinhao | Sow farm |
| Gansu Xinhao | Sow farm | Laiyang Xinhao | Sow farm | Hubei Xinhao | Sow farm |
| Gansu Xinhao | Sow farm | Hubei Xinhao | Sow farm | Juye Xinhao | Sow farm |
| Danxian Xinliu | Sow farm | Hezhou Xinhao | Sow farm | Ningming Xinhao | Sow farm |
| Gansu Xinhao | Sow farm | Suining New No. 6 | Sow farm | Laibin Xinhao | Sow farm |
| Pucheng Xinliu | Sow farm | Wuhe Xinhai | Sow farm | Ba Yinongmu | Sow farm |
| Hezhou Xinhao | Sow farm | Nanning Xinhao | Sow farm | Changyi Xinhao | Sow farm |
| Liaocheng Xinhao | Sow farm | Ningming Xinhao | Sow farm | Gansu Xinliu | Sow farm |
| Liaocheng Xinhao | Sow farm | Hezhou Xinhao | Sow farm | Hubei Xinhao | Sow farm |
| Gansu Xinliu | Sow farm | Liaocheng Xinhao | Sow farm | Laiyang Xinhao | Sow farm |
| Hubei Xinhao | Sow farm | Hubei Xinhao | Sow farm | Guangyuan Xinhao | Sow farm |
| Ningming Xinhao | Sow farm | Jingxian Xinhao | Sow farm | Laibin Xinhao | Sow farm |
| Gansu Xinhao | Sow farm | Kangping Xinwang | Sow farm | Hubei Xinhao | Sow farm |
| Guangdong Xinhao | Sow farm | Guangdong Xinhao | Sow farm | Laibin Xinhao | Sow farm |
| Nanning Xinhao | Sow farm | Ningming Xinhao | Sow farm | Guigang Xinliu | Sow farm |
| Jiangxi Xinliu | Sow farm | Baiyin Agriculture and Animal Husbandry | Sow farm | Yingcheng Xinhao | Sow farm |
| Hengnan Muyun | Sow farm | Hengnan Pastoral Cultivation | Sow farm | Heishan Xinliu | Sow farm |
| Ningming Xinhao | Sow farm | Hengnan Pastoral Cultivation | Sow farm | Hezhou Xinhao | Sow farm |
| Linyi Xinhao | Sow farm | Wulian Xinhao | Sow farm | Ningming Xinhao | Sow farm |
| Pucheng Xinliu | Sow farm | Yingcheng Xinhao | Sow farm | Nanning Xinhao | Sow farm |
| Shuozhou Xinhao | Sow farm | Yingcheng Xinhao | Sow farm | Laiyang Xinmu | Sow farm |
| Meishan Xinhai | Sow farm | Laiyang New Pastoral | Sow farm | Liaocheng Xinhao | Sow farm |
| Shuozhou Xinhao | Sow farm | Gansu Xinhao | Sow farm | Bai Yinongmu | Sow farm |
| Guangdong Xinhao | Sow farm | Hubei Xinhao | Sow farm | Liaocheng Xinhao | Sow farm |
| Jiangxi Xinliu | Sow farm | Wuqi New No. 6 | Sow farm | Guangdong Xinhao | Sow farm |
| Ningming Xinhao | Sow farm | Juye Xinhao | Sow farm | Lingbao Xinliu | Sow farm |
| Juye Xinhao | Sow farm | Hengnan Pastoral Cultivation | Sow farm | Hengnan Muyun | Sow farm |
| Yingcheng Xinhao | Sow farm | Guigang New No. 6 | Sow farm | Santai Nongmu | Sow farm |
| Shuozhou Xinliu | Sow farm | Hubei Xinhao | Sow farm | Ningming Xinhao | Sow farm |
| Suining Xinliu | Sow farm | Gansu Xinhao | Sow farm | Hubei Xinhao | Sow farm |
| Baiyin Agriculture and Animal Husbandry | Sow farm | Laibin Xinhao | Sow farm | Sichuan Xinhao | Sow farm |
| Yingcheng Xinhao | Sow farm | Laibin Xinhao | Sow farm | Hubei Xinhao | Sow farm |
| Anhui Xinliu | Sow farm | Yan'an Benyuan | Sow farm | Yingcheng Xinhao | Sow farm |
| Guangyuan Xinhao | Sow farm | Guangdong Xinhao | Sow farm | Hezhou Xinhao | Sow farm |
| Laixi Xinliu | Sow farm | Pucheng New No. 6 | Sow farm | Hezhou Xinhao | Sow farm |
| Laiyang Xinhao | Sow farm | Yichun Xinwang | Sow farm | Guigang Xinliu | Sow farm |
| Hubei Xinhao | Sow farm | Pucheng New No. 6 | Sow farm | Shuozhou Xinhao | Sow farm |
| Shuozhou Xinliu | Sow farm | Kangping Xinwang | Sow farm | Zhejiang Xinhai | Sow farm |
| Gaomi Xinliu | Sow farm | Lingbao New No. 6 | Sow farm | Pucheng Xinliu | Sow farm |
| Dong'e Xinliu | Sow farm | Ningming Xinhao | Sow farm | Pucheng Xinliu | Sow farm |
| Ningming Xinhao | Sow farm | Hezhou Xinhao | Sow farm | Yijun Xinliu | Sow farm |
| Hubei Xinhao | Sow farm | Gansu Xinhao | Sow farm | Laibin Xinhao | Sow farm |
| Gansu Xinhao | Sow farm | Baiyin Agriculture and Animal Husbandry | Sow farm | Liaoning Xinwang | Sow farm |
| Liaoning Xinwang | Sow farm | Fuxin Xinwang | Sow farm | Changyi Xinhao | Sow farm |
| Guigang Xinliu | Sow farm | Liaoning Xinwang | Sow farm | Dong'e Xinliu | Sow farm |
| Guangdong Xinhao | Sow farm | Hubei Xinhao | Sow farm | Yijun Xinliu | Sow farm |
| Jiangxi Xinliu | Sow farm | Gansu Xinhao | Sow farm | Yingcheng Xinhao | Sow farm |
| Gaomi Xinliu | Sow farm | Yijun New No. 6 | Sow farm | Liaoning Xinwang | Sow farm |
| Hainan Xinliu | Sow farm | Pucheng New No. 6 | Sow farm | Xiangzhou Xinhao | Sow farm |
| Yingcheng Xinhao | Sow farm | Hubei Xinhao | Sow farm | Laibin Xinhao | Sow farm |
| Gansu Xinhao | Sow farm | Ningbo Xinhai | Sow farm | Hubei Xinhao | Sow farm |
| Nanning Xinhao | Sow farm | Jiangxi New No. 6 | Sow farm | Gaomi Xinliu | Sow farm |
| Guangdong Xinhao | Sow farm | Wuhe Xinhai | Sow farm | Gansu Xinhao | Sow farm |
| Yichun Xinwang | Sow farm | Hezhou Xinhao | Sow farm | Wuhe Xinhai | Sow farm |
| Guangyuan Xinhao | Sow farm | Laibin Xinhao | Sow farm | Ningming Xinhao | Sow farm |
| Pucheng Xinliu | Sow farm | Yingcheng Xinhao | Sow farm | Guigang Xinliu | Sow farm |
| Juye Xinhao | Sow farm | Yan'an Benyuan | Sow farm | Kangping Xinwang | Sow farm |
| Hengnan Muyun | Sow farm | Hubei Xinhao | Sow farm | Yan'an Benyuan | Sow farm |
| Linyi Xinhao | Sow farm | Zhangjiakou Xinwang | Sow farm | Fuxin Xinwang | Sow farm |
| Zhucheng Xinliu | Sow farm | Yijun New No. 6 | Sow farm | Hubei Xinhao | Sow farm |
| Yijun Xinliu | Sow farm | Gansu Xinhao | Sow farm | Kangping Xinwang | Sow farm |
| Ningming Xinhao | Sow farm | Wuhe Xinhai | Sow farm | Wuqi Xinliu | Sow farm |
| Juye Xinhao | Sow farm | Guangyuan Xinhao | Sow farm | Guangyuan Xinhao | Sow farm |
| Laibin Xinhao | Sow farm | Guangdong Xinhao | Sow farm | Yijun Xinliu | Sow farm |
| Laibin Xinhao | Sow farm | Laixi New No. 6 | Sow farm | Zhangjiakou Xinwang | Sow farm |
| Jingxian Xinhao | Sow farm | Zhejiang Xinhai | Sow farm | Kangping Xinwang | Sow farm |
| Shuozhou Xinliu | Sow farm | Xiangzhou Xinhao | Sow farm | Laibin Xinhao | Sow farm |
| Yingcheng Xinhao | Sow farm | Liaoning Xinwang | Sow farm | Yan'an Benyuan | Sow farm |
| Donghai Xinhao | Sow farm | Guangan Xinhao | Sow farm | Hezhou Xinhao | Sow farm |
| Dong'e Xinliu | Sow farm | Guangdong Xinhao | Sow farm | Guangdong Xinhao | Sow farm |
| Hengnan Muyun | Sow farm | Laiyang Xinhao | Sow farm | Xiangzhou Xinhao | Sow farm |
| Laiyang Xinhao | Sow farm | Changyi Xinhao | Sow farm | Guangdong Xinhao | Sow farm |
| Baiyin Agriculture and Animal Husbandry | Sow farm | Kangping Xinwang | Sow farm | Bai Yinongmu | Sow farm |
| Juye Xinhao | Sow farm | Gaomi New No. 6 | Sow farm | Nanning Xinhao | Sow farm |
| Liaoning Xinwang | Sow farm | Gansu Xinhao | Sow farm | Jiaxiang Xinliu | Sow farm |
| Liaoning Xinwang | Sow farm | Yingcheng Xinhao | Sow farm | Lingbao Xinliu | Sow farm |
| Liaoning Xinwang | Sow farm | Santai Agriculture and Animal Husbandry | Sow farm | Ningming Xinhao | Sow farm |
| Shuozhou Xinliu | Sow farm | Laibin Xinhao | Sow farm | Laixi Xinliu | Sow farm |
| Wuqi Xinliu | Sow farm | Shuozhou Xinhao | Sow farm | Shuozhou Xinhao | Sow farm |
| Hubei Xinhao | Sow farm | Donghai Xinhao | Sow farm | Zhejiang Xinhai | Sow farm |
| Fuxin Xinwang | Sow farm | Guigang New No. 6 | Sow farm | Gaomi Xinliu | Sow farm |
| Guangdong Xinhao | Sow farm | Jiaxiang New No. 6 | Sow farm | Guangdong Xinhao | Sow farm |
| Laibin Xinhao | Sow farm | Dong'e New No. 6 | Sow farm | Kangping Xinwang | Sow farm |
| Dong'e Xinliu | Sow farm | Qinghua Agriculture and Animal Husbandry | Sow farm | Laiyang Xinhao | Sow farm |
| Hezhou Xinhao | Sow farm | Dongying Xinhao | Sow farm | Zhucheng Xinliu | Sow farm |
| Hubei Xinhao | Sow farm | Zhaoqing Xinhao | Sow farm | Zhangjiakou Xinwang | Sow farm |
| Kangping Xinwang | Sow farm | Hezhou Xinhao | Sow farm | Kangping Xinwang | Sow farm |
| Dong'e Xinliu | Sow farm | Xiangzhou Xinhao | Sow farm | Guangdong Xinhao | Sow farm |
| Kangping Xinwang | Sow farm | Wuhe Xinhai | Sow farm | Guanling Xinhai | Sow farm |
| Nanning Xinliu | Sow farm | Gaomi New No. 6 | Sow farm | Shuozhou Xinhao | Sow farm |
| Lingxian Xinliu | Sow farm | Dong'e New No. 6 | Sow farm | Danxian Xinliu | Sow farm |
| Hezhou Xinhao | Sow farm | Changyi Xinhao | Sow farm | Gansu Xinhao | Sow farm |
| Kangping Xinwang | Sow farm | Heishan New No. 6 | Sow farm | Dongying Xinhao | Sow farm |
| Ningming Xinhao | Sow farm | Zhuocheng New No. 6 | Sow farm | Laibin Xinhao | Sow farm |
| Hubei Xinhao | Sow farm | Kangping Xinwang | Sow farm | Zhucheng Xinliu | Sow farm |
| Liaoning Xinwang | Sow farm | Taian New No. 6 | Sow farm | Zhangjiakou Xinwang | Sow farm |
| Zhaoqing Xinhao | Sow farm | Yijun New No. 6 | Sow farm | Zhaoqing Xinhao | Sow farm |
| Hezhou Xinhao | Sow farm | Zhaoqing Xinhao | Sow farm | Kangping Xinwang | Sow farm |
| Zhangjiakou Xinwang | Sow farm | Baiyin Agriculture and Animal Husbandry | Sow farm | Qinghua Nongmu | Sow farm |
| Qinghua Agriculture and Animal Husbandry | Sow farm | Zhuocheng New No. 6 | Sow farm | Gansu Xinhao | Sow farm |
| Nanning Xinliu | Sow farm | Zhangjiakou Xinwang | Sow farm | Bai Yinongmu | Sow farm |
| Laibin Xinhao | Sow farm | Donghai Xinhao | Sow farm | Shibing Xinliu | Sow farm |
| Gaomi Xinliu | Sow farm | Yan'an Benyuan | Sow farm | Guanling Xinhai | Sow farm |
| Guigang Xinliu | Sow farm | Gansu Xinhao | Sow farm | Yan'an Benyuan | Sow farm |
| Dong'e Xinliu | Sow farm | Shuozhou Xinhao | Sow farm | Nanning Xinliu | Sow farm |
| Fuxin Xinwang | Sow farm | Baiyin Agriculture and Animal Husbandry | Sow farm | Liaocheng Xinhao | Sow farm |
| Hubei Xinhao | Sow farm | Jiaxiang New No. 6 | Sow farm | Gansu Xinhao | Sow farm |
| Zhangjiakou Xinwang | Sow farm | Xiangzhou Xinhao | Sow farm | Shuozhou Xinhao | Sow farm |
| Fuxin Xinwang | Sow farm | Heyang New No. 6 | Sow farm | Pucheng Xinliu | Sow farm |
| Yijun Xinliu | Sow farm | Nanning New No. 6 | Sow farm | Gansu Xinhao | Sow farm |
| Liaoning Xinwang | Sow farm | Zhangjiakou Xinwang | Sow farm | Nanning Xinliu | Sow farm |
| Gansu Xinhao | Sow farm | Hainan New No. 6 | Sow farm | Pingfu Nongmu | Sow farm |
| Kangping Xinwang | Sow farm | Ruyuan Xinhao | Sow farm | Kangping Xinwang | Sow farm |
| Yijun Xinliu | Sow farm | Kangping Xinwang | Sow farm | Gansu Xinhao | Sow farm |
| Laibin Xinhao | Sow farm | Zhangjiakou Xinwang | Sow farm | Heyang Xinliu | Sow farm |
| Laibin Xinhao | Sow farm | Guangyuan Xinhao | Sow farm | Zhaoqing Xinhao | Sow farm |
| Laibin Xinhao | Sow farm | Nanning New No. 6 | Sow farm | Laibin Xinhao | Sow farm |
| Hubei Xinhao | Sow farm | Kangping New Hope | Sow farm | Leshan Nongmu | Sow farm |
| Juye Xinhao | Sow farm | Juye Xinhao | Sow farm | Pingfu Agriculture and Animal Husbandry | Sow farm |
| Zhu Cheng Xinliu | Sow farm | Zhu Cheng Xinliu | Sow farm | Shibing Xinliu | Sow farm |
| Baiyin Agriculture and Animal Husbandry | Sow farm | Qinghua Agriculture and Animal Husbandry | Sow farm | Gansu Xinhao | Sow farm |
| Dong'e Xinliu | Sow farm | Weinan Xinliu | Sow farm | Zhaoqing Xinhao | Sow farm |
| Hubei Xinhao | Sow farm | Gansu Xinhao | Sow farm | Ningbo Xinhai | Sow farm |
| Zhaoqing Xinhao | Sow farm | Zhejiang Xinhai | Sow farm | Shibing Xinliu | Sow farm |
| Yingcheng Xinhao | Sow farm | Liaoning Xinwang | Sow farm | Huairou Agriculture and Animal Husbandry | Sow farm |
| Changyi Xinhao | Sow farm | Shibing Xinliu | Sow farm | Qinghua Agriculture and Animal Husbandry | Sow farm |
| Yan'an Benyuan | Sow farm | Wuqi Xinliu | Sow farm | Zhu Cheng Xinliu | Sow farm |
| Changyi Xinhao | Sow farm | Zhu Cheng Xinliu | Sow farm | Jingxian Xinhao | Sow farm |
| Wuhe Xinhai | Sow farm | Gansu Xinhao | Sow farm | Juye Xinhao | Sow farm |
| Kangping Xinwang | Sow farm | Ruyuan Xinhao | Sow farm | Zhangjiakou Xinwang | Sow farm |
| Gansu Xinhao | Sow farm | Gaomi Xinliu | Sow farm | Shibing Xinliu | Sow farm |
| Kangping Xinwang | Sow farm | Jingxian Xinhao | Sow farm | Shibing Xinliu | Sow farm |
| Wuhe Xinhai | Sow farm | Xiangzhou Xinhao | Sow farm | Pingfu Agriculture and Animal Husbandry | Sow farm |
| Zhangjiakou Xinwang | Sow farm | Shibing Xinliu | Sow farm | Ningbo Xinhai | Sow farm |
| Liaoning Xinwang | Sow farm | Pingfu Agriculture and Animal Husbandry | Sow farm | Bijie Xinliu | Sow farm |
| Kangping Xinwang | Sow farm | Laibin Xinhao | Sow farm | Weinan Xinliu | Sow farm |
| Ruyuan Xinhao | Sow farm | Donghai Xinhao | Sow farm | Jingxian Xinhao | Sow farm |
| Guigang Xinliu | Sow farm | Pingfu Agriculture and Animal Husbandry | Sow farm | Xiangzhou Xinhao | Sow farm |
| Gansu Xinliu | Sow farm | Zhaoqing Xinhao | Sow farm | Laixi Xinliu | Sow farm |
| Yingcheng Xinhao | Sow farm | Shuozhou Xinhao | Sow farm | Caoxian Xinhao | Sow farm |
| Dong'e Xinliu | Sow farm | Leshan Agriculture and Animal Husbandry | Sow farm | Pingfu Agriculture and Animal Husbandry | Sow farm |
| Zhu Cheng Xinliu | Sow farm | Pingfu Agriculture and Animal Husbandry | Sow farm | Gansu Xinhao | Sow farm |
| Qinghua Agriculture and Animal Husbandry | Sow farm | Guanling Xinhai | Sow farm | Ruyuan Xinhao | Sow farm |
| Kangping Xinwang | Sow farm | Shibing Xinliu | Sow farm | Gaomi Xinliu | Sow farm |
| Yichun Xinwang | Sow farm | Weinan Xinliu | Sow farm | Danxian Xinliu | Sow farm |
| Ningming Xinhao | Sow farm | Weinan Xinliu | Sow farm | Hainan Xinliu | Sow farm |
| Laixi Xinliu | Sow farm | Shibing Xinliu | Sow farm | Weinan Xinliu | Sow farm |
| Zhangjiakou Xinwang | Sow farm | Shanxian Xinliu | Sow farm | Heishan Xinliu | Sow farm |
| Gaotang Xinhao | Sow farm | Baiyin Agriculture and Animal Husbandry | Sow farm | Zhu Cheng Xinliu | Sow farm |
| Leshan Agriculture and Animal Husbandry | Sow farm | Shibing Xinliu | Sow farm | Pucheng Xinliu | Sow farm |
| Heyang Xinliu | Sow farm | Ningbo Xinhai | Sow farm | Shuozhou Xinhao | Sow farm |
| Zhaoqing Xinhao | Sow farm | Shanxian Xinliu | Sow farm | Zhaoqing Xinhao | Sow farm |
| Weinan Xinliu | Sow farm | Heishan Xinliu | Sow farm | Weinan Xinliu | Sow farm |
| Yan'an Benyuan | Sow farm | Zhaoqing Xinhao | Sow farm | Caoxian Xinhao | Sow farm |
| Heyang Xinliu | Sow farm | Laixi Xinliu | Sow farm | Weinan Xinliu | Sow farm |
| Xiangzhou Xinhao | Sow farm | Caoxian Xinhao | Sow farm | Jiaxiang Xinliu | Sow farm |
| Liaoning Xinwang | Sow farm | Pingfu Agriculture and Animal Husbandry | Sow farm | Caoxian Xinhao | Sow farm |
| Baiyin Agriculture and Animal Husbandry | Sow farm | Weinan Xinliu | Sow farm | Guangyuan Xinhao | Sow farm |
| Xiangzhou Xinhao | Sow farm | Hainan Xinliu | Sow farm | Baiyin Agriculture and Animal Husbandry | Sow farm |
| Weinan Xinliu | Sow farm | Gaotang Xinhao | Sow farm | Ruyuan Xinhao | Sow farm |
| Liaoning Xinwang | Sow farm | Liaocheng Xinhao | Sow farm | Hainan Xinliu | Sow farm |
| Xiangzhou Xinhao | Sow farm | Guanling Xinhai | Sow farm | Yanting Xinhao | Sow farm |
| Pingfu Agriculture and Animal Husbandry | Sow farm | Caoxian Xinhao | Sow farm | Wulian Xinhao | Sow farm |
| Wuqi Xinliu | Sow farm | Wulian Xinhao | Sow farm | Jiangxi Xinliu | Sow farm |
| Shuozhou Xinhao | Sow farm | Caoxian Xinhao | Sow farm | Caoxian Xinhao | Sow farm |
| Heishan Xinliu | Sow farm | Yanting Xinhao | Sow farm | Yanting Xinhao | Sow farm |
| Yan'an Benyuan | Sow farm | Bijie Xinliu | Sow farm | Yanting Xinhao | Sow farm |
| Zhaoqing Xinhao | Sow farm | Changyi Xinhao | Sow farm | Bijie Xinliu | Sow farm |
| Shibing Xinliu | Sow farm | Xiangzhou Xinhao | Sow farm | Gaomi Xinliu | Sow farm |
| Laibin Xinhao | Sow farm | Caoxian Xinhao | Sow farm | Yanting Xinhao | Sow farm |
| Wuhe Xinhai | Sow farm | Yanting Xinhao | Sow farm | Huaiyin Xincheng | Sow farm |
| Zhu Cheng Xinliu | Sow farm | Yanting Xinhao | Sow farm |  |  |
| Santai Agriculture and Animal Husbandry | Sow farm | Yanting Xinhao | Sow farm |  |  |
| Shibing Xinliu | Sow farm | Xiajiang Xinliu | Sow farm |  |  |
| Liaocheng Xinhao | Sow farm | Bijie Xinliu | Sow farm |  |  |
| Guigang Xinliu | Sow farm | Laixi Xinliu | Sow farm |  |  |
| Jiaxiang Xinliu | Sow farm | Laixi Xinliu | Sow farm |  |  |
| Baiyin Agriculture and Animal Husbandry | Sow farm | Gaomi Xinliu | Sow farm |  |  |
| Ningbo Xinhai | Sow farm |  |  |  |  |
| Hainan Xinliu | Sow farm |  |  |  |  |
| Weinan Xinliu | Sow farm |  |  |  |  |
| Pingfu Agriculture and Animal Husbandry | Sow farm |  |  |  |  |
| Xiangzhou Xinhao | Sow farm |  |  |  |  |
| Donghai Xinhao | Sow farm |  |  |  |  |
| Caoxian Xinhao | Sow farm |  |  |  |  |
| Shibing Xinliu | Sow farm |  |  |  |  |
| Shibing Xinliu | Sow farm |  |  |  |  |
| Ruyuan Xinhao | Sow farm |  |  |  |  |
| Guanling Xinhai | Sow farm |  |  |  |  |
| Jiaxiang Xinliu | Sow farm |  |  |  |  |
| Wuhe Xinhai | Sow farm |  |  |  |  |
| Pingfu Agriculture and Animal Husbandry | Sow farm |  |  |  |  |
| Shibing Xinliu | Sow farm |  |  |  |  |
| Weinan Xinliu | Sow farm |  |  |  |  |
| Laixi Xinliu | Sow farm |  |  |  |  |
| Changyi Xinhao | Sow farm |  |  |  |  |
| Pingfu Agriculture and Animal Husbandry | Sow farm |  |  |  |  |
| Laixi Xinliu | Sow farm |  |  |  |  |
| Ningbo Xinhai | Sow farm |  |  |  |  |
| Leshan Agriculture and Animal Husbandry | Sow farm |  |  |  |  |
| Caoxian Xinhao | Sow farm |  |  |  |  |
| Bijie Xinliu | Sow farm |  |  |  |  |
| Heishan Xinliu | Sow farm |  |  |  |  |
| Zhejiang Xinhai | Sow farm |  |  |  |  |
| Caoxian Xinhao | Sow farm |  |  |  |  |
| Bijie Xinliu | Sow farm |  |  |  |  |
| Yingtan Xinliu | Sow farm |  |  |  |  |
| Caoxian Xinhao | Sow farm |  |  |  |  |
| Donghai Xinhao | Sow farm |  |  |  |  |
| Gaomi Xinliu | Sow farm |  |  |  |  |
| Yanting Xinhao | Sow farm |  |  |  |  |
| Laixi Xinliu | Sow farm |  |  |  |  |
| Wulian Xinhao | Sow farm |  |  |  |  |
| Zhejiang Xinhai | Sow farm |  |  |  |  |
| Hezhou Xinhao | Sow farm |  |  |  |  |
| Yanting Xinhao | Sow farm |  |  |  |  |
| Yanting Xinhao | Sow farm |  |  |  |  |
| Xiajiang Xinliu | Sow farm |  |  |  |  |
| Xiangzhou Xinhao | Sow farm |  |  |  |  |
| Laiyang Xinmu | Sow farm |  |  |  |  |
| Yanting Xinhao | Sow farm |  |  |  |  |
| Laiyang Xinmu | Sow farm |  |  |  |  |
| Guanling Xinhai | Sow farm |  |  |  |  |
| Huairou Agriculture and Animal Husbandry | Sow farm |  |  |  |  |
| **Jan 2022** | | **Feb 2022** | | **Mar 2022** | |
| **Company (Co., Ltd.)** | **Type of pig farm** | **Company (Co., Ltd.)** | **Type of pig farm** | **Company (Co., Ltd.)** | **Type of pig farm** |
| Yingtan Xinliu | Sow farm | Dong'e Xinliu | Sow farm | Tianjin Nongmu | Sow farm |
| Jian Xinchi | Sow farm | Laizhou Xinhai | Sow farm | Dongying Xinhao | Fattening Farm |
| Shuozhou Xinhao | Sow farm | Laiyang Xinmu | Fattening farm | Liaoning Xinwang | Sow farm |
| Changyi Xinhao | Fattening farm | Changyi Xinhao | Fattening farm | Gaotang Xinhao | Fattening Farm |
| Shanxian Xinhao | Sow farm | Shuozhou Xinhao | Sow farm | Zhenjiang Xinhai | Sow farm |
| Laiyang Xinmu | Fattening farm | Guangan Xinhao | Sow farm | Laiyang Xinmu | Fattening Farm |
| Taian Xinliu | Fattening farm | Tongliao Xinhao | Sow farm | Tianjin Nongmu | Sow farm |
| Ningbo Xinhai | Sow farm | Guigang Xinliu | Sow farm | Shenze Xinhao | Sow farm |
| Shuozhou Xinhao | Sow farm | Taian Xinliu | Fattening farm | Taian Xinliu | Fattening Farm |
| Guangan Xinhao | Fattening farm | Dongying Xinhao | Fattening farm | Taian Xinliu | Sow farm |
| Dong'e Xinliu | Sow farm | Beijing Xinliu | Sow farm | Shenze Xinhao | Sow farm |
| Guangan Xinhao | Fattening farm | Shanxian Xinhao | Sow farm | Xiajin Nongmu | Sow farm |
| Shanxian Xinliu | Fattening farm | Meishan Xinhai | Sow farm | Gaomi Xinliu | Sow farm |
| Sichuan Xinhao | Sow farm | Laixi Xinliu | Sow farm | Xiangyang Xinhao | Sow farm |
| Jiangxi Xinliu | Sow farm | Fuxin Xinwang | Sow farm | Huanghua Xinhao | Sow farm |
| Wuming Xinliu | Sow farm | Hengnan Muyun | Sow farm | Laizhou Xinhai | Sow farm |
| Xiangyang Xinhao | Sow farm | Xiangyang Xinhao | Sow farm | Tianjin Nongmu | Sow farm |
| Laiyang Xinhao | Sow farm | Raoyang Xinhao | Sow farm | Huanghua Xinhao | Sow farm |
| Heishan Woniou | Sow farm | Ningbo Xinhai | Sow farm | Shenze Xinhao | Sow farm |
| Xiajiang Xinliu | Sow farm | Heishan Woniu | Sow farm | Xindongwan Xinliu | Sow farm |
| Beijing Xinliu | Sow farm | Guangan Xinhao | Sow farm | Gaomi Xinliu | Sow farm |
| Shenze Xinhao | Sow farm | Tianjin Nongmu | Sow farm | Tongliao Xinhao | Sow farm |
| Tianjin Nongmu | Sow farm | Tianjin Nongmu | Sow farm | Guigang Xinliu | Sow farm |
| Heishan Woniou | Sow farm | Beijing Xinliu | Sow farm | Heishan Xinliu | Sow farm |
| Sichuan Xinhao | Sow farm | Xiajiang Xinliu | Sow farm | Heishan Woniu | Sow farm |
| Dongying Xinhao | Fattening farm | Xiajin Nongmu | Sow farm | Shanxian Xinliu | Sow farm |
| Anhui Xinliu | Sow farm | Zhenjiang Xinhai | Sow farm | Zhangjiakou Xinwang | Sow farm |
| Dong'e Xinliu | Sow farm | Liaoning Xinwang | Sow farm | Sichuan Xinhao | Sow farm |
| Beijing Xinliu | Sow farm | Raoyang Xinhao | Sow farm | Shanxian Xinhao | Sow farm |
| Guanling Xinhai | Sow farm | Laizhou Xinhai | Sow farm | Anhui Xinliu | Sow farm |
| Laixi Xinliu | Sow farm | Heishan Woniu | Sow farm | Shuozhou Nongmu | Sow farm |
| Heishan Xinliu | Sow farm | Sichuan Xinhao | Sow farm | Hengnan Muyun | Sow farm |
| Guangan Xinhao | Sow farm | Suining Xinliu | Sow farm | Shenze Xinhao | Sow farm |
| Hainan Nongken | Sow farm | Shenze Xinhao | Sow farm | Tianjin Nongmu | Sow farm |
| Sichuan Xinhao | Sow farm | Anhui Xinliu | Sow farm | Beijing Xinliu | Sow farm |
| Shenze Xinhao | Sow farm | Taian Xinliu | Sow farm | Jingxian Xinhao | Sow farm |
| Laixi Xinliu | Sow farm | Xiangyang Xinhao | Sow farm | Laixi Xinliu | Sow farm |
| Gaotang Xinhao | Sow farm | Raoyang Xinhao | Sow farm | Ningbo Xinhai | Sow farm |
| Shanxian Xinliu | Sow farm | Shanxian Xinliu | Sow farm | Huanghua Xinhao | Sow farm |
| Guangan Xinhao | Sow farm | Shanxian Xinliu | Sow farm | Heishan Xinliu | Sow farm |
| Wuqi Xinliu | Sow farm | Kangping Xinwang | Sow farm | Shenze Xinhao | Sow farm |
| Zhejiang Xinhai | Sow farm | Shenze Xinhao | Sow farm | Zhuocheng Xinliu | Sow farm |
| Laizhou Xinhai | Sow farm | Linyi Xinhao | Sow farm | Xiajiang Xinliu | Sow farm |
| Hainan Xinliu | Sow farm | Xiangyang Xinhao | Sow farm | Gaomi Xinliu | Sow farm |
| Meishan Xinhai | Sow farm | Guanling Xinhai | Sow farm | Heishan Woniu | Sow farm |
| Raoyang Xinhao | Sow farm | Heishan Xinliu | Sow farm | Huanghua Xinhao | Sow farm |
| Langzhong Xinliu | Sow farm | Guangan Xinhao | Sow farm | Shanxian Xinliu | Sow farm |
| Guanglin Nongmu | Sow farm | Sichuan Xinhao | Sow farm | Suining Xinliu | Sow farm |
| Laizhou Xinhai | Sow farm | Gaotang Xinhao | Sow farm | Liaoning Xinwang | Sow farm |
| Suining Xinliu | Sow farm | Jiangxi Xinliu | Sow farm | Liaoning Xinwang | Sow farm |
| Yingtan Xinliu | Sow farm | Laixi Xinliu | Sow farm | Laizhou Xinhai | Sow farm |
| Gansu Xinliu | Sow farm | Suining Xinliu | Sow farm | Liaoning Xinwang | Sow farm |
| Laizhou Xinhai | Sow farm | Huanghua Xinhao | Sow farm | Kangping Xinwang | Sow farm |
| Anyang Xinliu | Sow farm | Wuqi Xinliu | Sow farm | Liaoning Xinwang | Sow farm |
| Tianjin Nongmu | Sow farm | Laizhou Xinhai | Sow farm | Liaoning Xinwang | Sow farm |
| Huaiyin Xinchi | Sow farm | Langzhong Xinliu | Sow farm | Xiangyang Xinhao | Sow farm |
| Xiangyang Xinhao | Sow farm | Laixi Xinliu | Sow farm | Liaoning Xinwang | Sow farm |
| Huairou Nongmu | Sow farm | Fuxin Xinwang | Sow farm | Liaoning Xinwang | Sow farm |
| Xiangyang Xinhao | Sow farm | Zhejiang Xinhai | Sow farm | Huanghua Xinhao | Sow farm |
| Dongying Xinhao | Sow farm | Hainan Nongken | Sow farm | Jian Xinchi | Sow farm |
| Laixi Xinliu | Sow farm | Xindongwan Xinliu | Sow farm | Laixi Xinliu | Sow farm |
| Raoyang Xinhao | Sow farm | Dongying Xinhao | Sow farm | Guang'an Xinhao | Sow farm |
| Dongying Xinhao | Sow farm | Yingtan Xinliu | Sow farm | Tongliao Xinhao | Sow farm |
| Shenze Xinhao | Sow farm | Tianjin Nongmu | Sow farm | Tianjin Nongmu | Sow farm |
| Jiangxi Xinliu | Sow farm | Taian Xinliu | Sow farm | Wannian Xinhai | Sow farm |
| Shenze Xinhao | Sow farm | Yichun Xinwang | Sow farm | Laixi Xinliu | Sow farm |
| Tianjin Nongmu | Sow farm | Gansu Xinliu | Sow farm | Gaotang Xinhao | Sow farm |
| Guangan Xinhao | Sow farm | Hainan Xinliu | Sow farm | Gansu Xinliu | Sow farm |
| Xiangyang Xinhao | Sow farm | Laizhou Xinhai | Sow farm | Guanling Xinhai | Sow farm |
| Liaoning Xinwang | Sow farm | Huaiyin Xincheng | Sow farm | Wuqi Xinliu | Sow farm |
| Huanghua Xinhao | Sow farm | Jian Xinchi | Sow farm | Laizhou Xinhai | Sow farm |
| Tianjin Nongmu | Sow farm | Shenze Xinhao | Sow farm | Gaomi Xinliu | Sow farm |
| Rongchang Pig Farm | Sow farm | Tianjin Nongmu | Sow farm | Sichuan Xinhao | Sow farm |
| Jian Xinchi | Sow farm | Zhu Cheng Xinliu | Sow farm | Langzhong Xinliu | Sow farm |
| Xiangyang Xinhao | Sow farm | Laixi Xinliu | Sow farm | Yichun Xinwang | Sow farm |
| Yantai Xinhao | Sow farm | Gaomi Xinliu | Sow farm | Hainan Nongken | Sow farm |
| Fuxin Xinwang | Sow farm | Laixi Xinliu | Sow farm | Pingfu Nongmu | Sow farm |
| Zhangjiakou Xinwang | Sow farm | Zhu Cheng Xinliu | Sow farm | Yucheng Xinliu | Sow farm |
| Yichun Xinwang | Sow farm | Jian Xinchi | Sow farm | Zhejiang Xinhai | Sow farm |
| Xindongwan Xinliu | Sow farm | Shenze Xinhao | Sow farm | Zhuocheng Xinliu | Sow farm |
| Jian Xinchi | Sow farm | Dongying Xinhao | Sow farm | Laizhou Xinhai | Sow farm |
| Gaotang Xinhao | Sow farm | Pingfu Nongmu | Sow farm | Hainan Xinliu | Sow farm |
| Tongliao Xinhao | Sow farm | Huanghua Xinhao | Sow farm | Huaiyin Xinchi | Sow farm |
| Huanghua Xinhao | Sow farm | Benxiang Liji | Sow farm | Liaoning Xinwang | Sow farm |
| Liaoning Xintao Wang | Sow farm | Zhu Cheng Xinliu | Sow farm | Yingtan Xinliu | Sow farm |
| Meishan Xinhai | Sow farm | Huanghua Xinhao | Sow farm | Heishan Xinliu | Sow farm |
| Shanxian Xinhao | Sow farm | Anyang Xinliu | Sow farm | Dongying Xinhao | Sow farm |
| Heishan Xinliu | Sow farm | Rongchang Pig Farm | Sow farm | Dongying Xinhao | Sow farm |
| Shenze Xinhao | Sow farm | Liaoning Xinwang | Sow farm | Zhuocheng Xinliu | Sow farm |
| Laixi Xinliu | Sow farm | Jiangxi Xinliu | Sow farm | Yantai Xinhao | Sow farm |
| Shanxian Xinhao | Sow farm | Heishan Xinliu | Sow farm | Linyi Xinhao | Sow farm |
| Yinbao Breeding | Sow farm | Yantai Xinhao | Sow farm | Laixi Xinliu | Sow farm |
| Taian Xinliu | Sow farm | Liaoning Xinwang | Sow farm | Xiajin Nongmu | Sow farm |
| Taian Xinliu | Sow farm | Liaoning Xinwang | Sow farm | Jingxian Xinhao | Sow farm |
| Jian Xinchi | Sow farm | Huanghua Xinhao | Sow farm | Jian Xinchi | Sow farm |
| Yantai Xinhao | Sow farm | Meishan Xinhai | Sow farm | Heishan Xinliu | Sow farm |
| Longzhou Xinhao | Sow farm | Liaoning Xinwang | Sow farm | Suining Xinliu | Sow farm |
| Gaomi Xinliu | Sow farm | Yucheng Xinliu | Sow farm | Meishan Xinhai | Sow farm |
| Kangping Xinwang | Sow farm | Liaoning Xinwang | Sow farm | Dongying Xinhao | Sow farm |
| Raoyang Xinhao | Sow farm | Laixi Xinliu | Sow farm | Dongying Xinhao | Sow farm |
| Laizhou Xinhai | Sow farm | Tianjin Nongmu | Sow farm | Anyang Xinliu | Sow farm |
| Dongying Xinhao | Sow farm | Heishan Xinliu | Sow farm | Dongying Xinhao | Sow farm |
| Huanghua Xinhao | Sow farm | Liaoning Xintao Wang | Sow farm | Fuxin Xinwang | Sow farm |
| Fuxin Xinwang | Sow farm | Gaomi Xinliu | Sow farm | Rongchang Pig Farm | Sow farm |
| Laizhou Xinhai | Sow farm | Laizhou Xinhai | Sow farm | Changyi Xinhao | Sow farm |
| Weinan Xinliu | Sow farm | Shanxian Xinhao | Sow farm | Laizhou Xinhai | Sow farm |
| Wuhe Xinhai | Sow farm | Gaotang Xinhao | Sow farm | Dongying Xinhao | Sow farm |
| Laizhou Xinhai | Sow farm | Liaoning Xinwang | Sow farm | Dongying Xinhao | Sow farm |
| Liaoning Xintao Wang | Sow farm | Dongying Xinhao | Sow farm | Dongying Xinhao | Sow farm |
| Shanxian Xinhao | Sow farm | Fuxin Xinwang | Sow farm | Wuhe Xinhai | Sow farm |
| Tianjin Nongmu | Sow farm | Dongying Xinhao | Sow farm | Xiajin Nongmu | Sow farm |
| Guigang Xinliu | Sow farm | Heishan Xinliu | Sow farm | Jiangxi Xinliu | Sow farm |
| Santai Nongmu | Sow farm | Dongying Xinhao | Sow farm | Laizhou Xinhai | Sow farm |
| Zhucheng Xinliu | Sow farm | Liaoning Xinwang | Sow farm | Liaoning Xintao Wang | Sow farm |
| Kangping Xinwang | Sow farm | Shanxian Xinliu | Sow farm | Laizhou Xinhai | Sow farm |
| Laixi Xinliu | Sow farm | Jian Xinchi | Sow farm | Xiajin Nongmu | Sow farm |
| Guangyuan Xinhao | Sow farm | Shanxian Xinhao | Sow farm | Jian Xinchi | Sow farm |
| Laixi Xinliu | Sow farm | Yinbao Breeding | Sow farm | Shanxian Xinhao | Sow farm |
| Yantai Xinhao | Sow farm | Linyi Xinhao | Sow farm | Kangping Xinwang | Sow farm |
| Yantai Xinhao | Sow farm | Longzhou Xinhao | Sow farm | Anyang Xinliu | Sow farm |
| Tongliao Xinhao | Sow farm | Wuhe Xinhai | Sow farm | Liaoning Xinwang | Sow farm |
| Longzhou Xinhao | Sow farm | Zhenjiang Xinhai | Sow farm | Kangping Xinwang | Sow farm |
| Lingbao Xinliu | Sow farm | Yantai Xinhao | Sow farm | Baiyin Nongmu | Sow farm |
| Laizhou Xinhai | Sow farm | Zhangjiakou Xinwang | Sow farm | Gaotang Xinhao | Sow farm |
| Longzhou Xinhao | Sow farm | Dongying Xinhao | Sow farm | Shanxian Xinliu | Sow farm |
| Dongying Xinhao | Sow farm | Dongying Xinhao | Sow farm | Shanxian Xinhao | Sow farm |
| Hubei Xinhao | Sow farm | Shanxian Xinhao | Sow farm | Yantai Xinhao | Sow farm |
| Shanxian Xinhao | Sow farm | Laizhou Xinhai | Sow farm | Linyi Pig Breeding | Sow farm |
| Leshan Nongmu | Sow farm | Laizhou Xinhai | Sow farm | Yantai Xinhao | Sow farm |
| Laizhou Xinhai | Sow farm | Zhu Cheng Xinliu | Sow farm | Dongying Xinhao | Sow farm |
| Laizhou Xinhai | Sow farm | Kangping Xinwang | Sow farm | Laixi Xinliu | Sow farm |
| Liaocheng Xinhao | Sow farm | Shanxian Xinhao | Sow farm | Yinbao Breeding | Sow farm |
| Juye Xinhao | Sow farm | Santai Nongmu | Sow farm | Kangping Xinwang | Sow farm |
| Dongying Xinhao | Sow farm | Liaoning Xintao Wang | Sow farm | Yantai Xinhao | Sow farm |
| Dongying Xinhao | Sow farm | Kangping Xinwang | Sow farm | Shanxian Xinhao | Sow farm |
| Dongying Xinhao | Sow farm | Dongying Xinhao | Sow farm | Shanxian Xinhao | Sow farm |
| Liangshan Xinliu | Sow farm | Longzhou Xinhao | Sow farm | Yantai Xinhao | Sow farm |
| Longzhou Xinhao | Sow farm | Longzhou Xinhao | Sow farm | Dong'e Xinliu | Sow farm |
| Dongying Xinhao | Sow farm | Gaomi Xinliu | Sow farm | Laizhou Xinhai | Sow farm |
| Gansu Xinhao | Sow farm | Dongying Xinhao | Sow farm | Jingxian Xinhao | Sow farm |
| Pingfu Nongmu | Sow farm | Yantai Xinhao | Sow farm | Heishan Xinliu | Sow farm |
| Guangdong Xinhao | Sow farm | Laizhou Xinhai | Sow farm | Laizhou Xinhai | Sow farm |
| Yantai Xinhao | Sow farm | Hubei Xinhao | Sow farm | Dongying Xinhao | Sow farm |
| Yan'an Benyuan | Sow farm | Guangyuan Xinhao | Sow farm | Yantai Xinhao | Sow farm |
| Yantai Xinhao | Sow farm | Laizhou Xinhai | Sow farm | Liaoning Xintao Wang | Sow farm |
| Shibing Xinliu | Sow farm | Yantai Xinhao | Sow farm | Laizhou Xinhai | Sow farm |
| Longzhou Xinhao | Sow farm | Dong'e Xinliu | Sow farm | Hubei Xinhao | Sow farm |
| Longzhou Xinhao | Sow farm | Laizhou Xinhai | Sow farm | Longzhou Xinhao | Sow farm |
| Qinghua Nongmu | Sow farm | Liaocheng Xinhao | Sow farm | Gaotang Xinhao | Sow farm |
| Hezhou Xinhao | Sow farm | Leshan Nongmu | Sow farm | Dong'e Xinliu | Sow farm |
| Laizhou Xinhai | Sow farm | Dongying Xinhao | Sow farm | Jiaxiang Xinliu | Sow farm |
| Yantai Xinhao | Sow farm | Liangshan Xinliu | Sow farm | Santai Nongmu | Sow farm |
| Heishan Xinliu | Sow farm | Lingbao Xinliu | Sow farm | Juye Xinhao | Sow farm |
| Santai Nongmu | Sow farm | Xiajin Nongmu | Sow farm | Longzhou Xinhao | Sow farm |
| Longzhou Xinhao | Sow farm | Yantai Xinhao | Sow farm | Lingbao Xinliu | Sow farm |
| Linyi Xinhao | Sow farm | Juye Xinhao | Sow farm | Longzhou Xinhao | Sow farm |
| Yantai Xinhao | Sow farm | Wuhe Xinhai | Sow farm | Yan'an Benyuan | Sow farm |
| Yantai Xinhao | Sow farm | Longzhou New Hope | Sow farm | Guangyuan Xinhao | Sow farm |
| Yucheng Xinliu | Sow farm | Shenze New Hope | Sow farm | Longzhou Xinhao | Sow farm |
| Wuhe Xinhai | Sow farm | Kangping New Outlook | Sow farm | Leshan Agriculture and Animal Husbandry | Sow farm |
| Caoxian Xinhao | Sow farm | Yan'an Benyuan | Sow farm | Yantai Xinhao | Sow farm |
| Tongliao Xinhao | Sow farm | Juye New Hope | Sow farm | Yantai Xinhao | Sow farm |
| Dongying Xinhao | Sow farm | Heishan Xinliu | Sow farm | Longzhou Xinhao | Sow farm |
| Dongying Xinhao | Sow farm | Yantai New Hope | Sow farm | Juye Xinhao | Sow farm |
| Ruyuan Xinhao | Sow farm | Longzhou New Hope | Sow farm | Wuhe Xinhai | Sow farm |
| Xiajin Nongmu | Sow farm | Pingfu Animal Husbandry | Sow farm | Laizhou Xinhai | Sow farm |
| Liaoning Xinwang | Sow farm | Shibing Xinliu | Sow farm | Shibing Xinliu | Sow farm |
| Taian Xinliu | Sow farm | Rugao New Hope | Sow farm | Longzhou Xinhao | Sow farm |
| Shenze Xinhao | Sow farm | Yantai New Hope | Sow farm | Qinghua Agriculture and Animal Husbandry | Sow farm |
| Nanning Xinliu | Sow farm | Gaotang New Hope | Sow farm | Jiangxi Xinliu | Sow farm |
| Nanning Xinhao | Sow farm | Longzhou New Hope | Sow farm | Nanning Xinliu | Sow farm |
| Jiaxiang Xinliu | Sow farm | Gansu New Hope | Sow farm | Zhangjiakou New Hope | Sow farm |
| Zhongshan Nongmu | Sow farm | Guangdong New Hope | Sow farm | Caoxian Xinhao | Sow farm |
| Shanxian Xinliu | Sow farm | Hezhou New Hope | Sow farm | Yantai Xinhao | Sow farm |
| Liaoning Xinwang | Sow farm | Santai Animal Husbandry | Sow farm | Hezhou Xinhao | Sow farm |
| Liaoning Xinwang | Sow farm | Laizhou Xinhai | Sow farm | Longzhou Xinhao | Sow farm |
| Liaoning Xinwang | Sow farm | Qinghua Animal Husbandry | Sow farm | Kangping New Hope | Sow farm |
| Jiangyou Pig Farm | Sow farm | Longzhou New Hope | Sow farm | Guangdong Xinhao | Sow farm |
| Gaotang Xinhao | Sow farm | Kangping New Outlook | Sow farm | Santai Agriculture and Animal Husbandry | Sow farm |
| Anyang Xinliu | Sow farm | Guigang Xinliu | Sow farm | Liangshan Xinliu | Sow farm |
| Liaoning Xintao Wang | Sow farm | Yantai New Hope | Sow farm | Gansu Xinhao | Sow farm |
| Xindongwan Xinliu | Sow farm | Xiajin Animal Husbandry | Sow farm | Pingfu Agriculture and Animal Husbandry | Sow farm |
| Chenzhou Xinhao | Sow farm | Dong'e Xinliu | Sow farm | Liaocheng Xinhao | Sow farm |
| Linyi Xinhao | Sow farm | Yantai New Hope | Sow farm | Laixi Xinliu | Sow farm |
| Jingxian Xinhao | Sow farm | Zhangjiakou New Outlook | Sow farm | Weinan Xinliu | Sow farm |
| Huanghua Xinhao | Sow farm | Nanning Xinliu | Sow farm | Ruyuan Xinhao | Sow farm |
| Pucheng Xinliu | Sow farm | Linyi Breeding Pig | Sow farm | Benxiang Lijia | Sow farm |
| Xiajin Nongmu | Sow farm | Caoxian New Hope | Sow farm | Tongliao Xinhao | Sow farm |
| Dong'e Xinliu | Sow farm | Suining Xinliu | Sow farm | Chenzhou Xinhao | Sow farm |
| Heishan Xinliu | Sow farm | Ruyuan New Hope | Sow farm | Tongliao Xinhao | Sow farm |
| Liaoning Xinwang | Sow farm | Zhu Cheng Xinliu | Sow farm | Zhongshan Agriculture and Animal Husbandry | Sow farm |
| Liaoning Xinwang | Sow farm | Tongliao New Hope | Sow farm | Nanning Xinhao | Sow farm |
| Kangping Xinwang | Sow farm | Jingxian New Hope | Sow farm | Anyang Xinliu | Sow farm |
| Dong'e Xinliu | Sow farm | Jiaxiang Xinliu | Sow farm | Dong'e Xinliu | Sow farm |
| Liaoning Xinwang | Sow farm | Tongliao New Hope | Sow farm | Tongliao Xinhao | Sow farm |
| Tongliao Xinhao | Sow farm | Shenze New Hope | Sow farm | Fuxin New Hope | Sow farm |
| Suining Xinliu | Sow farm | Zhangjiakou New Outlook | Sow farm | Liaoning New Tao望 | Sow farm |
| Fuxin Xinwang | Sow farm | Kangping New Outlook | Sow farm | Baiyin Agriculture and Animal Husbandry | Sow farm |
| Fuxin Xinwang | Sow farm | Weinan Xinliu | Sow farm | Yucheng Xinliu | Sow farm |
| Heishan Xinliu | Sow farm | Xiangyang New Hope | Sow farm | Jiangyou Pig Farm | Sow farm |
| Pingfu Nongmu | Sow farm | Nanning New Hope | Sow farm | Liaoning New Hope | Sow farm |
| Huanghua Xinhao | Sow farm | Zhongshan Animal Husbandry | Sow farm | Pucheng Xinliu | Sow farm |
| Juye Xinhao | Sow farm | Dong'e Xinliu | Sow farm | Tongliao Xinhao | Sow farm |
| Meishan Xinhai | Sow farm | Tongliao New Hope | Sow farm | Fuxin New Hope | Sow farm |
| Jingxian Xinhao | Sow farm | Chenzhou New Hope | Sow farm | Guigang Xinliu | Sow farm |
| Fuxin Xinwang | Sow farm | Liaoning Xintao Outlook | Sow farm | Guigang Xinliu | Sow farm |
| Suining Xinliu | Sow farm | Jiangyou Pig Farm | Sow farm | Liaoning New Hope | Sow farm |
| Xiajin Nongmu | Sow farm | Fuxin New Outlook | Sow farm | Wulian Xinhao | Sow farm |
| Dong'e Xinliu | Sow farm | Huanghua New Hope | Sow farm | Xiangyang Xinhao | Sow farm |
| Yucheng Xinliu | Sow farm | Xindongwan Xinliu | Sow farm | Yichun New Hope | Sow farm |
| Liaoning Xinwang | Sow farm | Jingxian New Hope | Sow farm | Changyi Xinhao | Sow farm |
| Juye Xinhao | Sow farm | Changyi New Hope | Sow farm | Anyang Xinliu | Sow farm |
| Xiajin Nongmu | Sow farm | Gaomi Xinliu | Sow farm | Dongying Xinhao | Sow farm |
| Xiajin Nongmu | Sow farm | Tongliao New Hope | Sow farm | Heishan Xinliu | Sow farm |
| Xiajin Nongmu | Sow farm | Baiyin Animal Husbandry | Sow farm | Liaoning New Hope | Sow farm |
| Meishan Xinhai | Sow farm | Pucheng Xinliu | Sow farm | Langzhong Xinliu | Sow farm |
| Liaoning Xintao Wang | Sow farm | Anyang Xinliu | Sow farm | Liaoning New Tao望 | Sow farm |
| Anyang Xinliu | Sow farm | Laixi Xinliu | Sow farm | Liaoning New Tao望 | Sow farm |
| Liaoning Xintao Wang | Sow farm | Fuxin New Outlook | Sow farm | Wulian Xinhao | Sow farm |
| Laixi Xinliu | Sow farm | Liaoning New Outlook | Sow farm | Liaoning New Tao望 | Sow farm |
| Liaoning Xintao Wang | Sow farm | Laixi Xinliu | Sow farm | Langzhong Xinliu | Sow farm |
| Xiajin Nongmu | Sow farm | Anyang Xinliu | Sow farm | Taipeng Xinliu | Sow farm |
| Liaoning Xinwang | Sow farm | Anyang Xinliu | Sow farm | Yingcheng Xinhao | Sow farm |
| Yingtan Xinliu | Sow farm | Guigang Xinliu | Sow farm | Changyi Xinhao | Sow farm |
| Liaoning Xinwang | Sow farm | Juye New Hope | Sow farm | Wuhe Xinhai | Sow farm |
| Xiajin Nongmu | Sow farm | Baiyin Animal Husbandry | Sow farm | Xiajin Agriculture and Animal Husbandry | Sow farm |
| Wuhe Xinhai | Sow farm | Liaoning New Outlook | Sow farm | Zhejiang Xinhai | Sow farm |
| Xiajin Nongmu | Sow farm | Yucheng Xinliu | Sow farm | Juye Xinhao | Sow farm |
| Linyi Pig Breeding | Sow farm | Meishan Xinhai | Sow farm | Meishan Xinhai | Sow farm |
| Langzhong Xinliu | Sow farm | Dongying New Hope | Sow farm | Shuozhou Xinhao | Sow farm |
| Baiyin Nongmu | Sow farm | Liaoning Xintao Outlook | Sow farm | Jiangxi Xinliu | Sow farm |
| Wulian Xinhao | Sow farm | Liaoning Xintao Outlook | Sow farm | Yingcheng Xinhao | Sow farm |
| Kangping Xinwang | Sow farm | Liaoning Xintao Outlook | Sow farm | Dong'e Xinliu | Sow farm |
| Wuhe Xinhai | Sow farm | Wulian New Hope | Sow farm | Huaiyin New Chi | Sow farm |
| Kangping Xinwang | Sow farm | Liaoning New Outlook | Sow farm | Wuhe Xinhai | Sow farm |
| Meishan Xinhai | Sow farm | Dong'e Xinliu | Sow farm | Anhui Xinliu | Sow farm |
| Hubei Xinhao | Sow farm | Gaomi Xinliu | Sow farm | Liaoning New Tao望 | Sow farm |
| Anyang Xinliu | Sow farm | Yichun New Outlook | Sow farm | Jiangxi Xinliu | Sow farm |
| Xiajin Nongmu | Sow farm | Langzhong Xinliu | Sow farm | Wuhe Xinhai | Sow farm |
| Dong'e Xinliu | Sow farm | Shuozhou New Hope | Sow farm | Meishan Xinhai | Sow farm |
| Guangan Xinhao | Sow farm | Taian Xinliu | Sow farm | Shuozhou Xinhao | Sow farm |
| Gaomi Xinliu | Sow farm | Meishan Xinhai | Sow farm | Wuhe Xinhai | Sow farm |
| Jiangxi Xinliu | Sow farm | Yingtan Xinliu | Sow farm | Baiyin Agriculture and Animal Husbandry | Sow farm |
| Wuhe Xinhai | Sow farm | Xiajin Animal Husbandry | Sow farm | Gansu Xinhao | Sow farm |
| Zhu Cheng Xinliu | Sow farm | Xiajin Animal Husbandry | Sow farm | Hengnan Muyun | Sow farm |
| Dongying Xinhao | Sow farm | Yingcheng New Hope | Sow farm | Jiangxi Xinliu | Sow farm |
| Gansu Xinliu | Sow farm | Liaoning Xintao Outlook | Sow farm | Taipeng Xinliu | Sow farm |
| Taian Xinliu | Sow farm | Heishan Xinliu | Sow farm | Lingbao Xinliu | Sow farm |
| Laixi Xinliu | Sow farm | Wulian New Hope | Sow farm | Xindongwan Xinliu | Sow farm |
| Wuhe Xinhai | Sow farm | Heyang Xinliu | Sow farm | Yingcheng Xinhao | Sow farm |
| Guigang Xinliu | Sow farm | Meishan Xinhai | Sow farm | Hengnan Muyun | Sow farm |
| Liaoning Xintao Wang | Sow farm | Gansu New Hope | Sow farm | Heishan Xinliu | Sow farm |
| Guangan Xinhao | Sow farm | Taian Xinliu | Sow farm | Xiajin Agriculture and Animal Husbandry | Sow farm |
| Jiangxi Xinliu | Sow farm | Wuhe Xinhai | Sow farm | Heyang Xinliu | Sow farm |
| Heishan Xinliu | Sow farm | Wuhe Xinhai | Sow farm | Jiangxi Xinliu | Sow farm |
| Liaoning Xinwang | Sow farm | Taian Xinliu | Sow farm | Yingtan Xinliu | Sow farm |
| Dong'e Xinliu | Sow farm | Anhui Xinliu | Sow farm | Wuhe Xinhai | Sow farm |
| Yingcheng Xinhao | Sow farm | Wuhe Xinhai | Sow farm | Xiajin Agriculture and Animal Husbandry | Sow farm |
| Baiyin Nongmu | Sow farm | Baiyin Animal Husbandry | Sow farm | Dong'e Xinliu | Sow farm |
| Jiangxi Xinliu | Sow farm | Liaoning New Outlook | Sow farm | Juye Xinhao | Sow farm |
| Wuhe Xinhai | Sow farm | Jiangxi Xinliu | Sow farm | Wuhe Xinhai | Sow farm |
| Leshan Nongmu | Sow farm | Wuhe Xinhai | Sow farm | Taipeng Xinliu | Sow farm |
| Xiajin Nongmu | Sow farm | Hengnan Muyun | Sow farm | Guangan Xinhao | Sow farm |
| Yingtan Xinliu | Sow farm | Gansu Xinliu | Sow farm | Juye Xinhao | Sow farm |
| Shuozhou Xinhao | Sow farm | Jiangxi Xinliu | Sow farm | Lingbao Xinliu | Sow farm |
| Wuhe Xinhai | Sow farm | Zhejiang Xinhai | Sow farm | Wuhe Xinhai | Sow farm |
| Hengnan Muyun | Sow farm | Hengnan Muyun | Sow farm | Xiajin Agriculture and Animal Husbandry | Sow farm |
| Yichun Xinwang | Sow farm | Shuozhou New Hope | Sow farm | Yingcheng Xinhao | Sow farm |
| Heyang Xinliu | Sow farm | Jiangxi Xinliu | Sow farm | Shuozhou Xinhao | Sow farm |
| Liaocheng Xinhao | Sow farm | Shuozhou New Hope | Sow farm | Weinan Xinliu | Sow farm |
| Yichun Xinwang | Sow farm | Wuhe Xinhai | Sow farm | Nanning Xinliu | Sow farm |
| Hengnan Muyun | Sow farm | Wuhe Xinhai | Sow farm | Liaoning New Tao望 | Sow farm |
| Yingcheng Xinhao | Sow farm | Gansu New Hope | Sow farm | Zhejiang Xinhai | Sow farm |
| Liaoning Xintao Wang | Sow farm | Lingbao Xinliu | Sow farm | Guangyuan Xinhao | Sow farm |
| Taian Xinliu | Sow farm | Zhejiang Xinhai | Sow farm | Yichun New Hope | Sow farm |
| Hubei Xinhao | Sow farm | Changyi New Hope | Sow farm | Hainan Agriculture and Forestry | Sow farm |
| Juye Xinhao | Sow farm | Xiajin Animal Husbandry | Sow farm | Xiajin Agriculture and Animal Husbandry | Sow farm |
| Heishan Woniou | Sow farm | Wuhe Xinhai | Sow farm | Pingfu Agriculture and Animal Husbandry | Sow farm |
| Hainan State Farms | Sow farm | Xiajin Animal Husbandry | Sow farm | Liaocheng Xinhao | Sow farm |
| Anhui Xinliu | Sow farm | Yingtan Xinliu | Sow farm | Shenze Xinhao | Sow farm |
| Guangyuan Xinhao | Sow farm | Changyi New Hope | Sow farm | Heishan Woniou | Sow farm |
| Zhejiang Xinhai | Sow farm | Xiajin Agriculture and Animal Husbandry | Sow farm | Guangan Xinhao | Sow farm |
| Juye Xinhao | Sow farm | Hainan State Farms | Sow farm | Caoxian Xinhao | Sow farm |
| Shuozhou Xinhao | Sow farm | Langzhong Xinliu | Sow farm | Guanling Xinhai | Sow farm |
| Changyi Xinhao | Sow farm | Xiajin Agriculture and Animal Husbandry | Sow farm | Hubei Xinhao | Sow farm |
| Changyi Xinhao | Sow farm | Yichun Xinwang | Sow farm | Guigang Xinliu | Sow farm |
| Hubei Xinhao | Sow farm | Hubei Xinhao | Sow farm | Xiajin Nongmu | Sow farm |
| Hubei Xinhao | Sow farm | Dong'e Xinliu | Sow farm | Santai Nongmu | Sow farm |
| Wuhe Xinhai | Sow farm | Guangyuan Xinhao | Sow farm | Guigang Xinliu | Sow farm |
| Zhou Cheng Xinliu | Sow farm | Yingcheng Xinhao | Sow farm | Yingtan Xinliu | Sow farm |
| Hainan State Farms | Sow farm | Xiajin Agriculture and Animal Husbandry | Sow farm | Wuqi Xinliu | Sow farm |
| Yingcheng Xinhao | Sow farm | Liaoning New Tao Wang | Sow farm | Wuqi Xinliu | Sow farm |
| Hubei Xinhao | Sow farm | Guigang Xinliu | Sow farm | Kangping Xinwang | Sow farm |
| Wuqi Xinliu | Sow farm | Liaocheng Xinhao | Sow farm | Hubei Xinhao | Sow farm |
| Heishan Xinliu | Sow farm | Heishan Xinliu | Sow farm | Pingfu Nongmu | Sow farm |
| Zhou Cheng Xinliu | Sow farm | Yingcheng Xinhao | Sow farm | Kangping Xinwang | Sow farm |
| Yingcheng Xinhao | Sow farm | Hubei Xinhao | Sow farm | Hubei Xinhao | Sow farm |
| Yingcheng Xinhao | Sow farm | Xiajin Agriculture and Animal Husbandry | Sow farm | Dongying Xinhao | Sow farm |
| Yingcheng Xinhao | Sow farm | Heishan Woniu | Sow farm | Yingcheng Xinhao | Sow farm |
| Shuozhou Xinhao | Sow farm | Yingcheng Xinhao | Sow farm | Shuozhou Xinhao | Sow farm |
| Santai Agriculture and Animal Husbandry | Sow farm | Pingfu Agriculture and Animal Husbandry | Sow farm | Laibin Xinhao | Sow farm |
| Guigang Xinliu | Sow farm | Hengnan Muyun | Sow farm | Caoxian Xinhao | Sow farm |
| Zhejiang Xinhai | Sow farm | Pingfu Agriculture and Animal Husbandry | Sow farm | Xiajin Nongmu | Sow farm |
| Ningming Xinliu | Sow farm | Guangan Xinhao | Sow farm | Hezhou Xinhao | Sow farm |
| Gansu Xinhao | Sow farm | Juye Xinhao | Sow farm | Hainan Nongken | Sow farm |
| Ningming Xinliu | Sow farm | Santai Agriculture and Animal Husbandry | Sow farm | Yingcheng Xinhao | Sow farm |
| Gansu Xinhao | Sow farm | Hainan State Farms | Sow farm | Shuozhou Xinhao | Sow farm |
| Lingbao Xinliu | Sow farm | Yingcheng Xinhao | Sow farm | Lingao Xinliu | Sow farm |
| Chenzhou Xinhao | Sow farm | Guigang Xinliu | Sow farm | Guanling Xinhai | Sow farm |
| Hengnan Muyun | Sow farm | Guanling Xinhai | Sow farm | Pucheng Xinliu | Sow farm |
| Kangping Xinwang | Sow farm | Guangan Xinhao | Sow farm | Shuozhou Xinhao | Sow farm |
| Baiyin Agriculture and Animal Husbandry | Sow farm | Juye Xinhao | Sow farm | Kangping Xinwang | Sow farm |
| Nanning Xinhao | Sow farm | Wuqi Xinliu | Sow farm | Jian Xinchi | Sow farm |
| Langzhong Xinliu | Sow farm | Lingao Xinliu | Sow farm | Shuozhou Xinhao | Sow farm |
| Gaomi Xinliu | Sow farm | Guigang Xinliu | Sow farm | Meishan Xinhai | Sow farm |
| Ningming Xinliu | Sow farm | Ningming Xinhao | Sow farm | Hengnan Muyun | Sow farm |
| Yingcheng Xinhao | Sow farm | Weinan Xinliu | Sow farm | Hubei Xinhao | Sow farm |
| Nanning Xinhao | Sow farm | Caoxian Xinhao | Sow farm | Laiyang Xinhao | Sow farm |
| Guigang Xinliu | Sow farm | Baiyin Agriculture and Animal Husbandry | Sow farm | Pucheng Xinliu | Sow farm |
| Guanling Xinhai | Sow farm | Hubei Xinhao | Sow farm | Hubei Xinhao | Sow farm |
| Liaocheng Xinhao | Sow farm | Jiangxi Xinliu | Sow farm | Ningming Xinhao | Sow farm |
| Chenzhou Xinhao | Sow farm | Gansu Xinhao | Sow farm | Juye Xinhao | Sow farm |
| Lingao Xinliu | Sow farm | Lingbao Xinliu | Sow farm | Chenzhou Xinhao | Sow farm |
| Guigang Xinliu | Sow farm | Baiyin Agriculture and Animal Husbandry | Sow farm | Baiyin Nongmu | Sow farm |
| Hubei Xinhao | Sow farm | Ningming Xinhao | Sow farm | Jiaxiang Xinliu | Sow farm |
| Chenzhou Xinhao | Sow farm | Hezhou Xinhao | Sow farm | Laibin Xinhao | Sow farm |
| Changyi Xinhao | Sow farm | Ningming Xinhao | Sow farm | Hezhou Xinhao | Sow farm |
| Gansu Xinhao | Sow farm | Shuozhou Xinhao | Sow farm | Hubei Xinhao | Sow farm |
| Caoxian Xinhao | Sow farm | Guigang Xinliu | Sow farm | Chenzhou Xinhao | Sow farm |
| Baiyin Agriculture and Animal Husbandry | Sow farm | Leshan Agriculture and Animal Husbandry | Sow farm | Gansu Xinhao | Sow farm |
| Laibin Xinhao | Sow farm | Pucheng Xinliu | Sow farm | Hubei Xinhao | Sow farm |
| Hezhou Xinhao | Sow farm | Gansu Xinhao | Sow farm | Yijun Xinliu | Sow farm |
| Liaocheng Xinhao | Sow farm | Laibin Xinhao | Sow farm | Liaocheng Xinhao | Sow farm |
| Hubei Xinhao | Sow farm | Pingfu Agriculture and Animal Husbandry | Sow farm | Yan'an Benyuan | Sow farm |
| Pingfu Agriculture and Animal Husbandry | Sow farm | Pucheng Xinliu | Sow farm | Hubei Xinhao | Sow farm |
| Hubei Xinhao | Sow farm | Chenzhou Xinhao | Sow farm | Xiangzhou Xinhao | Sow farm |
| Wulian Xinhao | Sow farm | Kangping Xinwang | Sow farm | Chenzhou Xinhao | Sow farm |
| Hezhou Xinhao | Sow farm | Hubei Xinhao | Sow farm | Yingcheng Xinhao | Sow farm |
| Hubei Xinhao | Sow farm | Hezhou Xinhao | Sow farm | Hezhou Xinhao | Sow farm |
| Caoxian Xinhao | Sow farm | Hubei Xinhao | Sow farm | Xiangzhou Xinhao | Sow farm |
| Yijun Xinliu | Sow farm | Kangping Xinwang | Sow farm | Guangdong Xinhao | Sow farm |
| Guangyuan Xinhao | Sow farm | Yingcheng Xinhao | Sow farm | Heyang Xinliu | Sow farm |
| Pucheng Xinliu | Sow farm | Shuozhou Xinhao | Sow farm | Hubei Xinhao | Sow farm |
| Liaoning Xinwang | Sow farm | Hubei Xinhao | Sow farm | Liaocheng Xinhao | Sow farm |
| Guangdong Xinhao | Sow farm | Shuozhou Xinhao | Sow farm | Pingfu Nongmu | Sow farm |
| Baiyin Agriculture and Animal Husbandry | Sow farm | Chenzhou Xinhao | Sow farm | Hezhou Xinhao | Sow farm |
| Hezhou Xinhao | Sow farm | Nanning Xinhao | Sow farm | Gansu Xinhao | Sow farm |
| Gaomi Xinliu | Sow farm | Hezhou Xinhao | Sow farm | Zhangjiakou Xinwang | Sow farm |
| Pucheng Xinliu | Sow farm | Shuozhou Xinhao | Sow farm | Baiyin Nongmu | Sow farm |
| Shuozhou Xinhao | Sow farm | Hubei Xinhao | Sow farm | Hezhou Xinhao | Sow farm |
| Danxian Xinliu | Sow farm | Nanning Xinhao | Sow farm | Guigang Xinliu | Sow farm |
| Zhou Cheng Xinliu | Sow farm | Gansu Xinhao | Sow farm | Kangping Xinwang | Sow farm |
| Laibin Xinhao | Sow farm | Guangdong Xinhao | Sow farm | Guigang Xinliu | Sow farm |
| Liaocheng Xinhao | Sow farm | Wuqi Xinliu | Sow farm | Gansu Xinliu | Sow farm |
| Guigang Xinliu | Sow farm | Hezhou Xinhao | Sow farm | Nanning Xinhao | Sow farm |
| Shuozhou Xinhao | Sow farm | Yijun Xinliu | Sow farm | Pucheng Xinliu | Sow farm |
| Hezhou Xinhao | Sow farm | Hezhou Xinhao | Sow farm | Yijun Xinliu | Sow farm |
| Laibin Xinhao | Sow farm | Laiyang Xinhao | Sow farm | Leshan Nongmu | Sow farm |
| Pingfu Agriculture and Animal Husbandry | Sow farm | Liaocheng Xinhao | Sow farm | Guangyuan Xinhao | Sow farm |
| Nanning Xinhao | Sow farm | Chenzhou Xinhao | Sow farm | Laibin Xinhao | Sow farm |
| Wuqi Xinliu | Sow farm | Xiangzhou Xinhao | Sow farm | Baiyin Nongmu | Sow farm |
| Gansu Xinhao | Sow farm | Nanning Xinhao | Sow farm | Nanning Xinhao | Sow farm |
| Gaomi Xinliu | Sow farm | Laibin Xinhao | Sow farm | Bijie Xinliu | Sow farm |
| Laibin Xinhao | Sow farm | Caoxian Xinhao | Sow farm | Laibin Xinhao | Sow farm |
| Jiaxiang Xinliu | Sow farm | Pingfu Agriculture and Animal Husbandry | Sow farm | Gansu Xinhao | Sow farm |
| Lingbao Xinliu | Sow farm | Hubei Xinhao | Sow farm | Caoxian Xinhao | Sow farm |
| Yijun Xinliu | Sow farm | Laibin Xinhao | Sow farm | Gansu Xinhao | Sow farm |
| Gansu Xinhao | Sow farm | Laibin Xinhao | Sow farm | Laibin Xinhao | Sow farm |
| Laiyang Xinhao | Sow farm | Laibin Xinhao | Sow farm | Pingfu Nongmu | Sow farm |
| Laibin Xinhao | Sow farm | Yijun Xinliu | Sow farm | Shibing Xinliu | Sow farm |
| Ningming Xinhao | Sow farm | Shuozhou Xinhao | Sow farm | Gansu Xinhao | Sow farm |
| Yijun Xinliu | Sow farm | Guanling Xinhai | Sow farm | Ningming Xinhao | Sow farm |
| Yan'an Benyuan | Sow farm | Yingcheng Xinhao | Sow farm | Gansu Xinliu | Sow farm |
| Ningming Xinhao | Sow farm | Heishan Xinliu | Sow farm | Hubei Xinhao | Sow farm |
| Laibin Xinhao | Sow farm | Kangping Xinwang | Sow farm | Yan'an Benyuan | Sow farm |
| Kangping Xinwang | Sow farm | Baiyin Agriculture and Animal Husbandry | Sow farm | Nanning Xinhao | Sow farm |
| Gansu Xinliu | Sow farm | Shibing Xinliu | Sow farm | Baiyin Nongmu | Sow farm |
| Kangping Xinwang | Sow farm | Hubei Xinhao | Sow farm | Pingfu Nongmu | Sow farm |
| Leshan Agriculture and Animal Husbandry | Sow farm | Liaocheng Xinhao | Sow farm | Caoxian Xinhao | Sow farm |
| Shuozhou Xinhao | Sow farm | Liaocheng Xinhao | Sow farm | Shibing Xinliu | Sow farm |
| Laiyang Ximu | Sow farm | Pucheng Xinliu | Sow farm | Heishan Xinliu | Sow farm |
| Nanning Xinliu | Sow farm | Gansu Xinhao | Sow farm | Shibing Xinliu | Sow farm |
| Shibing Xinliu | Sow farm | Yijun Xinliu | Sow farm | Gansu Xinhao | Sow farm |
| Pingfu Agriculture and Animal Husbandry | Sow farm | Huaiyin New Chi | Sow farm | Laiyang Xinhao | Sow farm |
| Shuozhou Xinhao | Sow farm | Jiaxiang Xinliu | Sow farm | Zhangjiakou Xinwang | Sow farm |
| Guanling Xinhai | Sow farm | Yan'an Benyuan | Sow farm | Laibin Xinhao | Sow farm |
| Hezhou Xinhao | Sow farm | Jingxian Xinhao | Sow farm | Yan'an Benyuan | Sow farm |
| Kangping Xinwang | Sow farm | Dongying Xinhao | Sow farm | Leshan Nongmu | Sow farm |
| Juye Xinhao | Sow farm | Juye Xinhao | Sow farm | Weinan Xinliu | Sow farm |
| Hubei Xinhao | Sow farm | Laibin Xinhao | Sow farm | Nanning Xinliu | Sow farm |
| Xiangzhou Xinhao | Sow farm | Gansu Xinhao | Sow farm | Shibing Xinliu | Sow farm |
| Shibing Xinliu | Sow farm | Hubei Xinhao | Sow farm | Yijun Xinliu | Sow farm |
| Lingao Xinliu | Sow farm | Guangyuan Xinhao | Sow farm | Xiangzhou Xinhao | Sow farm |
| Yan'an Benyuan | Sow farm | Laibin Xinhao | Sow farm | Gansu Xinhao | Sow farm |
| Ningming Xinhao | Sow farm | Leshan Agriculture and Animal Husbandry | Sow farm | Guangyuan Xinhao | Sow farm |
| Laibin Xinhao | Sow farm | Ningming Xinhao | Sow farm | Yanting Xinhou | Sow farm |
| Dongying Xinhao | Sow farm | Shibing Xinliu | Sow farm | Shibing Xinliu | Sow farm |
| Zhangjiakou Xinwang | Sow farm | Laiyang Xinmu | Sow farm | Gansu Xinhao | Sow farm |
| Gansu Xinhao | Sow farm | Kangping Xinwang | Sow farm | Laibin Xinhao | Sow farm |
| Pucheng Xinliu | Sow farm | Hubei Xinhao | Sow farm | Bijie Xinliu | Sow farm |
| Guangdong Xinhao | Sow farm | Xiangzhou Xinhao | Sow farm | Zhangjiakou Xinwang | Sow farm |
| Baiyin Agriculture and Animal Husbandry | Sow farm | Gansu Xinhao | Sow farm | Guangdong Xinhao | Sow farm |
| Nanning Xinhao | Sow farm | Nanning Xinliu | Sow farm | Hubei Xinhao | Sow farm |
| Guangdong Xinhao | Sow farm | Heyang Xinliu | Sow farm | Xiangzhou Xinhao | Sow farm |
| Bijie Xinliu | Sow farm | Gansu Xinliu | Sow farm | Liaocheng Xinhao | Sow farm |
| Heishan Xinliu | Sow farm | Xiangzhou Xinhao | Sow farm | Laibin Xinhao | Sow farm |
| Yan'an Benyuan | Sow farm | Shibing Xinliu | Sow farm | Guangdong Xinhao | Sow farm |
| Shibing Xinliu | Sow farm | Gansu Xinhao | Sow farm | Ningming Xinhao | Sow farm |
| Zhangjiakou Xinwang | Sow farm | Baiyin Agriculture and Animal Husbandry | Sow farm | Guangdong Xinhao | Sow farm |
| Yanting Xinhao | Sow farm | Ningming Xinhao | Sow farm | Pucheng Xinliu | Sow farm |
| Guangdong Xinhao | Sow farm | Yan'an Benyuan | Sow farm | Ningming Xinhao | Sow farm |
| Gansu Xinhao | Sow farm | Ningming Xinhao | Sow farm | Jiaxiang Xinliu | Sow farm |
| Shibing Xinliu | Sow farm | Ningming Xinhao | Sow farm | Ningming Xinhao | Sow farm |
| Weinan Xinliu | Sow farm | Nanning Xinhao | Sow farm | Lingao Xinliu | Sow farm |
| Heyang Xinliu | Sow farm | Gansu Xinhao | Sow farm | Ningming Xinhao | Sow farm |
| Pingfu Agriculture and Animal Husbandry | Sow farm | Bijie Xinliu | Sow farm | Yanting Xinhou | Sow farm |
| Nanning Xinliu | Sow farm | Laibin Xinhao | Sow farm | Weinan Xinliu | Sow farm |
| Guangyuan Xinhao | Sow farm | Zhangjiakou Xinwang | Sow farm | Gansu Xinhao | Sow farm |
| Gansu Xinhao | Sow farm | Guangdong Xinhao | Sow farm | Pingfu Nongmu | Sow farm |
| Ningming Xinhao | Sow farm | Shibing Xinliu | Sow farm | Yanting Xinhou | Sow farm |
| Wuhe Xinhai | Sow farm | Zhangjiakou Xinwang | Sow farm | Ningming Xinhao | Sow farm |
| Shibing Xinliu | Sow farm | Pingfu Agriculture and Animal Husbandry | Sow farm | Nanning Xinhao | Sow farm |
| Laiyang Xinhao | Sow farm | Xiangzhou Xinhao | Sow farm | Zhaoqing Xinhao | Sow farm |
| Kangping New Hope | Sow farm | Caoxian Xinhao | Sow farm | Wuhe Xinhai | Sow farm |
| Xiangzhou Xinhao | Sow farm | Guangdong Xinhao | Sow farm | Guangdong Xinhao | Sow farm |
| Zhangjiakou New Hope | Sow farm | Guangdong Xinhao | Sow farm | Xiangzhou Xinhao | Sow farm |
| Gansu Xinhao | Sow farm | Zhangjiakou Xinwang | Sow farm | Laiyang Xinmu | Sow farm |
| Guangdong Xinhao | Sow farm | Shibing Xinliu | Sow farm | Zhaoqing Xinhao | Sow farm |
| Zhangjiakou New Hope | Sow farm | Yanting Xinhao | Sow farm | Zhaoqing Xinhao | Sow farm |
| Zhaoqing Xinhao | Sow farm | Laiyang Xinhao | Sow farm | Qinghua Nongmu | Sow farm |
| Laibin Xinhao | Sow farm | Xiangzhou Xinhao | Sow farm | Gansu Xinhao | Sow farm |
| Caoxian Xinhao | Sow farm | Weinan Xinliu | Sow farm | Yanting Xinhao | Sow farm |
| Zhaoqing Xinhao | Sow farm | Bijie Xinliu | Sow farm | Zhaoqing Xinhao | Sow farm |
| Weinan Xinliu | Sow farm | Yan'an Benyuan | Sow farm | Ningbo Xinhai | Sow farm |
| Gansu Xinhao | Sow farm | Zhaoqing Xinhao | Sow farm | Weinan Xinliu | Sow farm |
| Gansu Xinhao | Sow farm | Guangdong Xinhao | Sow farm | Hainan Xinliu | Sow farm |
| Jiangxi Xinliu | Sow farm | Nanning Xinliu | Sow farm | Zhenjiang Xinhai | Sow farm |
| Xiangzhou Xinhao | Sow farm | Zhaoqing Xinhao | Sow farm | Ruyuan Xinhao | Sow farm |
| Xiangzhou Xinhao | Sow farm | Guangyuan Xinhao | Sow farm | Ruyuan Xinhao | Sow farm |
| Qinghua Agriculture and Animal Husbandry | Sow farm | Zhaoqing Xinhao | Sow farm | Hainan Xinliu | Sow farm |
| Zhaoqing Xinhao | Sow farm | Pingfu Animal Husbandry | Sow farm | Ningbo Xinhai | Sow farm |
| Xiangzhou Xinhao | Sow farm | Zhaoqing Xinhao | Sow farm | Qinghua Nongmu | Sow farm |
| Jingxian Xinhao | Sow farm | Pucheng Xinliu | Sow farm | Laiyang Xinmu | Sow farm |
| Ningbo Xinhai | Sow farm | Yanting Xinhao | Sow farm | Xiajiang Xinliu | Sow farm |
| Zhaoqing Xinhao | Sow farm | Gansu Xinhao | Sow farm | Xianyang Yongxiang | Sow farm |
| Weinan Xinliu | Sow farm | Wuhe Xinhai | Sow farm | Yan'an Benyuan | Sow farm |
| Baiyin Agriculture and Animal Husbandry | Sow farm | Yanting Xinhao | Sow farm | Laiyang Xinmu | Sow farm |
| Bijie Xinliu | Sow farm | Lingao Xinliu | Sow farm | Yancheng Zhongtai | Sow farm |
| Shuozhou Xinhao | Sow farm | Qinghua Animal Husbandry | Sow farm | Huanghua Xinhao | Sow farm |
| Ruyuan Xinhao | Sow farm | Weinan Xinliu | Sow farm | Huairou Nongmu | Sow farm |
| Weinan Xinliu | Sow farm | Weinan Xinliu | Sow farm | Laiyang Xinhao | Sow farm |
| Jiaxiang Xinliu | Sow farm | Caoxian Xinhao | Sow farm | Laibin Xinhao | Sow farm |
| Ningbo Xinhai | Sow farm | Hainan Xinliu | Sow farm | Yan'an Benyuan | Sow farm |
| Qinghua Agriculture and Animal Husbandry | Sow farm | Jiaxiang Xinliu | Sow farm | Laibin Xinhao | Sow farm |
| Pucheng Xinliu | Sow farm | Ruyuan Xinhao | Sow farm | Laiyang Xinhao | Sow farm |
| Yanting Xinhao | Sow farm | Yanting Xinhao | Sow farm | Hebei Xinhao | Sow farm |
| Yanting Xinhao | Sow farm | Ningbo Xinhai | Sow farm | Hubei Xinhao | Sow farm |
| Yanting Xinhao | Sow farm | Qinghua Animal Husbandry | Sow farm | Dong'e Xinliu | Sow farm |
| Caoxian Xinhao | Sow farm | Ruyuan Xinhao | Sow farm | Laibin Xinhao | Sow farm |
| Hainan Xinliu | Sow farm | Hainan Xinliu | Sow farm | Caoxian Xinhao | Sow farm |
| Ruyuan Xinhao | Sow farm | Ningbo Xinhai | Sow farm |  |  |
| Hainan Xinliu | Sow farm | Xiajiang Xinliu | Sow farm |  |  |
| Huaiyin New Chi | Sow farm | Jian Xinchi | Sow farm |  |  |
| Xiajiang Xinliu | Sow farm | Laiyang Xinmu | Sow farm |  |  |
| Benxiang Li Family | Sow farm | Xianyang Yongxiang | Sow farm |  |  |
| Jian New Chi | Sow farm | Yan'an Benyuan | Sow farm |  |  |
| Rugao Xinhao | Sow farm | Laiyang Xinmu | Sow farm |  |  |
| Hubei Xinhao | Sow farm | Yancheng Zhongtai | Sow farm |  |  |
| Laiyang Xinmu | Sow farm | Jiaxiang Xinliu | Sow farm |  |  |
| Pingfu Agriculture and Animal Husbandry | Sow farm | Liaocheng Xinhao | Sow farm |  |  |
| Xianyang Yongxiang | Sow farm | Dong'e Xinliu | Sow farm |  |  |
| Yan'an Benyuan | Sow farm |  |  |  |  |
| Laiyang Xinmu | Sow farm |  |  |  |  |
| Hebei Xinhao | Sow farm |  |  |  |  |
| Huanghua Xinhao | Sow farm |  |  |  |  |
| Xiangzhou Xinhao | Sow farm |  |  |  |  |
| Dong'e Xinliu | Sow farm |  |  |  |  |
| Yan'an Benyuan | Sow farm |  |  |  |  |
| Kangping New Hope | Sow farm |  |  |  |  |
| Fucheng Xinhao | Sow farm |  |  |  |  |
| Jiaxiang Xinliu | Sow farm |  |  |  |  |
| Liaocheng Xinhao | Sow farm |  |  |  |  |
| Dong'e Xinliu | Sow farm |  |  |  |  |
| Zhangwu New Hope | Sow farm |  |  |  |  |
| Hebei Xinhao | Sow farm |  |  |  |  |
| Juye Xinhao | Sow farm |  |  |  |  |
| **Apr 2022** | | **May 2022** | | **Jun 2022** | |
| **Company (Co., Ltd.)** | **Type of pig farm** | **Company (Co., Ltd.)** | **Type of pig farm** | **Company (Co., Ltd.)** | **Type of pig farm** |
| Ji'an Xincheng | Sow farm | Gaotang Xinhao | Fattening farm | Laixi Xinliu | Sow farm |
| Tianjin Nongmu | Sow farm | Yantai Xinhao | Fattening farm | Heishan Xinliu | Sow farm |
| Laiyang Xinmu | Fattening farm | Suining Xin Liu | Sow farm | Tianjin Agriculture and Animal Husbandry | Sow farm |
| Beijing Xinliu | Sow farm | Baiyin Agriculture and Animal Husbandry | Sow farm | Tianjin Agriculture and Animal Husbandry | Sow farm |
| Dong'e Xinliu | Sow farm | Kangping Xinwang | Sow farm | Kangping Xinwang | Sow farm |
| Nanning Xinliu | Sow farm | Beijing Xin Liu | Sow farm | Kangping Xinwang | Sow farm |
| Laizhou Xinhai | Sow farm | Baiyin Agriculture and Animal Husbandry | Sow farm | Guanling Xinhai | Sow farm |
| Zhuocheng Xinliu | Sow farm | Zhenjiang Xinhai | Sow farm | Suining Xinliu | Sow farm |
| Taian Xinliu | Sow farm | Baiyin Agriculture and Animal Husbandry | Sow farm | Zhu Cheng Xinliu | Sow farm |
| Gaotang Xinhao | Fattening farm | Heishan Woniou | Sow farm | Hengnan Muyun | Sow farm |
| Anhui Xinliu | Sow farm | Baiyin Agriculture and Animal Husbandry | Sow farm | Beijing Xinliu | Sow farm |
| Dong'e Xinliu | Sow farm | Shanxian Xin Liu | Sow farm | Zhu Cheng Xinliu | Sow farm |
| Shanxian Xinliu | Sow farm | Baiyin Agriculture and Animal Husbandry | Sow farm | Shuozhou Xinhao | Sow farm |
| Ji'an Xincheng | Sow farm | Dongying Xinhao | Sow farm | Tongliao Xinhao | Sow farm |
| Dingzhou Xinhao | Sow farm | Shanxian Xin Liu | Fattening farm | Suining Xinliu | Fattening farm |
| Zhuocheng Xinliu | Sow farm | Shanxian Xin Liu | Sow farm | Dingzhou Xinhao | Sow farm |
| Heishan Woniou | Sow farm | Changyi Xinhao | Sow farm | Anyang Xinliu | Sow farm |
| Xindongwan Xinliu | Sow farm | Baiyin Agriculture and Animal Husbandry | Sow farm | Xiajin Agriculture and Animal Husbandry | Sow farm |
| Xiangyang Xinhao | Sow farm | Dongying Xinhao | Sow farm | Xiajin Agriculture and Animal Husbandry | Sow farm |
| Laixi Xinliu | Sow farm | Heishan Woniou | Sow farm | Zhenjiang Xinhai | Sow farm |
| Guigang Xinliu | Sow farm | Guigang Xin Liu | Sow farm | Zhangjiakou Xinwang | Sow farm |
| Beijing Xinliu | Sow farm | Beijing Xin Liu | Sow farm | Zhangxin Wangchang | Sow farm |
| Ji'an Xincheng | Sow farm | Raoyang Xinhao | Sow farm | Beijing Xinliu | Sow farm |
| Shuozhou Xinhao | Sow farm | Suining Xin Liu | Sow farm | Kangping Xinwang | Sow farm |
| Tongliao Xinhao | Sow farm | Shuozhou Xinhao | Sow farm | Fuxin Xinwang | Sow farm |
| Kangping Xinwang | Sow farm | Xindongwan Xin Liu | Sow farm | Zhangxin Wangchang | Sow farm |
| Tongliao Xinhao | Sow farm | Xiajiang Xin Liu | Sow farm | Zhangxin Wangchang | Sow farm |
| Suining Xinliu | Sow farm | Nanning Xin Liu | Sow farm | Dongying Xinhao | Sow farm |
| Shanxian Xinliu | Sow farm | Fuping Xin Liu | Sow farm | Suining Xinliu | Sow farm |
| Liaoning Xinwang | Sow farm | Suining Xin Liu | Fattening farm | Kangping Xinwang | Sow farm |
| Fuping Xinliu | Sow farm | Taian Xin Liu | Sow farm | Xiajiang Xinliu | Sow farm |
| Ji'an Xincheng | Sow farm | Laixi Xin Liu | Sow farm | Huaiyin Xincheng | Sow farm |
| Xiajiang Xinliu | Sow farm | Dingzhou Xinhao | Sow farm | Wuhe Xinhai | Sow farm |
| Zhenjiang Xinhai | Sow farm | Zhucheng Xin Liu | Sow farm | Guang'an Xinhao | Sow farm |
| Heishan Woniou | Sow farm | Suining Xin Liu | Sow farm | Shenze Xinhao | Sow farm |
| Laixi Xinliu | Sow farm | Tongliao Xinhao | Sow farm | Fuxin Xinwang | Sow farm |
| Ningbo Xinhai | Sow farm | Langzhong Xin Liu | Sow farm | Guanling Xinhai | Sow farm |
| Baiyin Nongmu | Sow farm | Laizhou Xinhai | Sow farm | Langzhong Xinliu | Sow farm |
| Zhuocheng Xinliu | Sow farm | Guang'an Xinhao | Sow farm | Gansu Xinliu | Sow farm |
| Ji'an Xincheng | Sow farm | Gansu Xin Liu | Sow farm | Wuqi Xinliu | Sow farm |
| Tianjin Nongmu | Sow farm | Zhucheng Xin Liu | Sow farm | Laixi Xinliu | Sow farm |
| Dongying Xinhao | Sow farm | Wuqi Xin Liu | Sow farm | Gansu Xinhao | Sow farm |
| Guang'an Xinhao | Sow farm | Fuxin Xinwang | Sow farm | Laizhou Xinhai | Sow farm |
| Gansu Xinhao | Sow farm | Zhenjiang Xinhai | Sow farm | Raoyang Xinhao | Sow farm |
| Gansu Xinliu | Sow farm | Guanling Xinhai | Sow farm | Tianjin Agriculture and Animal Husbandry | Sow farm |
| Laizhou Xinhai | Sow farm | Ningbo Xinhai | Sow farm | Dongying Xinhao | Sow farm |
| Laixi Xinliu | Sow farm | Huaiyin Xincheng | Sow farm | Dongying Xinhao | Sow farm |
| Wuqi Xinliu | Sow farm | Dongying Xinhao | Sow farm | Anyang Xinliu | Sow farm |
| Zhenjiang Xinhai | Sow farm | Tianjin Agriculture and Animal Husbandry | Sow farm | Laizhou Xinhai | Sow farm |
| Guanling Xinhai | Sow farm | Zhejiang Xinhai | Sow farm | Juye Xinhao | Sow farm |
| Langzhong Xinliu | Sow farm | Dongying Xinhao | Sow farm | Pingfu Agriculture and Animal Husbandry | Sow farm |
| Yantai Xinhao | Sow farm | Laizhou Xinhai | Sow farm | Zhejiang Xinhai | Sow farm |
| Laixi Xinliu | Sow farm | Heishan Woniou | Sow farm | Ningbo Xinhai | Sow farm |
| Yichun Xinwang | Sow farm | Pingfu Agriculture and Animal Husbandry | Sow farm | Meishan Xinhai | Sow farm |
| Zhuocheng Xinliu | Sow farm | Hengnan Muyun | Sow farm | Hainan Agriculture and Reclamation | Sow farm |
| Gaotang Xinhao | Sow farm | Dongying Xinhao | Sow farm | Xindongwan Xinliu | Sow farm |
| Pingfu Nongmu | Sow farm | Hainan Agriculture and Forestry | Sow farm | Nanning Xinliu | Sow farm |
| Laizhou Xinhai | Sow farm | Yantai Xinhao | Sow farm | Hainan Xinliu | Sow farm |
| Suining Xinliu | Sow farm | Dong'e Xin Liu | Sow farm | Yichun Xinwang | Sow farm |
| Laizhou Xinhai | Sow farm | Yichun Xinwang | Sow farm | Xiajin Agriculture and Animal Husbandry | Sow farm |
| Zhejiang Xinhai | Sow farm | Wuhe Xin Hai | Sow farm | Yingtan Xinliu | Sow farm |
| Hainan Nongken | Sow farm | Hainan Xin Liu | Sow farm | Kangping Xinwang | Sow farm |
| Laizhou Xinhai | Sow farm | Dongying Xinhao | Sow farm | Anyang Xinliu | Sow farm |
| Huaiyin Xincheng | Sow farm | Yingtan Xin Liu | Sow farm | Taian Xinliu | Sow farm |
| Liaoning Xintao Wang | Sow farm | Xiajin Agriculture and Animal Husbandry | Sow farm | Liaoning Xinwang | Sow farm |
| Hainan Xinliu | Sow farm | Fuxin Xinwang | Sow farm | Zhenjiang Xinhai | Sow farm |
| Dongying Xinhao | Sow farm | Zhenjiang Xinhai | Sow farm | Changyi Xinhao | Sow farm |
| Dongying Xinhao | Sow farm | Dong'e Xin Liu | Sow farm | Dongying Xinhao | Sow farm |
| Hengnan Muyun | Sow farm | Sichuan Xinhao | Sow farm | Dongying Xinhao | Sow farm |
| Laixi Xinliu | Sow farm | Gansu Xinhao | Sow farm | Yantai Xinhao | Sow farm |
| Yingtan Xinliu | Sow farm | Tongliao Xinhao | Sow farm | Tongliao Xinhao | Sow farm |
| Fuxin Xinwang | Sow farm | Dongying Xinhao | Sow farm | Laizhou Xinhai | Sow farm |
| Sichuan Xinhao | Sow farm | Dongying Xinhao | Sow farm | Liaoning Xintao Wang | Sow farm |
| Gansu Xinhao | Sow farm | Anyang Xin Liu | Sow farm | Rongchang Pig Farm | Sow farm |
| Tongliao Xinhao | Sow farm | Laizhou Xinhai | Sow farm | Anyang Xinliu | Sow farm |
| Xiangyang Xinhao | Sow farm | Gaotang Xinhao | Sow farm | Tongliao Xinhao | Sow farm |
| Shanxian Xinliu | Sow farm | Dongying Xinhao | Sow farm | Yantai Xinhao | Sow farm |
| Dongying Xinhao | Sow farm | Rongchang Pig Farm | Sow farm | Laizhou Xinhai | Sow farm |
| Xiajin Nongmu | Sow farm | Laixi Xin Liu | Sow farm | Zhenjiang Xinhai | Sow farm |
| Changyi Xinhao | Sow farm | Linyi Breeding Pig | Sow farm | Yantai Xinhao | Sow farm |
| Yucheng Xinliu | Sow farm | Anyang Xin Liu | Sow farm | Yantai Xinhao | Sow farm |
| Heishan Xinliu | Sow farm | Laizhou Xinhai | Sow farm | Dongying Xinhao | Sow farm |
| Dongying Xinhao | Sow farm | Yucheng Xin Liu | Sow farm | Linyi Breeding Pig | Sow farm |
| Wuhe Xinhai | Sow farm | Yantai Xinhao | Sow farm | Dong'e Xinliu | Sow farm |
| Rongchang Pig Farm | Sow farm | Yantai Xinhao | Sow farm | Raoyang Xinhao | Sow farm |
| Dongying Xinhao | Sow farm | Laizhou Xinhai | Sow farm | Dongying Xinhao | Sow farm |
| Dongying Xinhao | Sow farm | Meishan Xinhai | Sow farm | Yinbao Breeding | Sow farm |
| Yucheng Xinliu | Sow farm | Kangping Xinwang | Sow farm | Wuhe Xinhai | Sow farm |
| Linyi Zhongzhu | Sow farm | Yantai Xinhao | Sow farm | Dongying Xinhao | Sow farm |
| Dongying Xinhao | Sow farm | Dongying Xinhao | Sow farm | Laizhou Xinhai | Sow farm |
| Fuxin Xinwang | Sow farm | Wuhe Xin Hai | Sow farm | Danxian Xinhao | Sow farm |
| Yantai Xinhao | Sow farm | Danxian Xinhao | Sow farm | Laizhou Xinhai | Sow farm |
| Huaiyin Xincheng | Sow farm | Yinbao Breeding | Sow farm | Yantai Xinhao | Sow farm |
| Wuhe Xinhai | Sow farm | Laizhou Xinhai | Sow farm | Gaotang Xinhao | Sow farm |
| Meishan Xinhai | Sow farm | Laizhou Xinhai | Sow farm | Laizhou Xinhai | Sow farm |
| Anyang Xinliu | Sow farm | Yantai Xinhao | Sow farm | Jiangxi Xinliu | Sow farm |
| Yantai Xinhao | Sow farm | Yantai Xinhao | Sow farm | Danxian Xinliu | Sow farm |
| Yantai Xinhao | Sow farm | Dongying Xinhao | Sow farm | Gansu Xinhao | Sow farm |
| Liaoning Xinwang | Sow farm | Kangping Xinwang | Sow farm | Laizhou Xinhai | Sow farm |
| Laizhou Xinhai | Sow farm | Yantai Xinhao | Sow farm | Yantai Xinhao | Sow farm |
| Dongying Xinhao | Sow farm | Danxian Xinhao | Sow farm | Shuozhou Xinliu | Sow farm |
| Zhenjiang Xinhai | Sow farm | Laizhou Xinhai | Sow farm | Kangping Xinwang | Sow farm |
| Laizhou Xinhai | Sow farm | Weinan Xin Liu | Sow farm | Laizhou Xinhai | Sow farm |
| Taian Xinliu | Sow farm | Dong'e Xin Liu | Sow farm | Yantai Xinhao | Sow farm |
| Zhenjiang Xinhai | Sow farm | Jiaxiang Xin Liu | Sow farm | Danxian Xinliu | Sow farm |
| Yantai Xinhao | Sow farm | Dong'e Xin Liu | Sow farm | Jiaxiang Xinliu | Sow farm |
| Laizhou Xinhai | Sow farm | Kangping Xinwang | Sow farm | Yantai Xinhao | Sow farm |
| Dong'e Xinliu | Sow farm | Laizhou Xinhai | Sow farm | Suining Xinliu | Sow farm |
| Anyang Xinliu | Sow farm | Danxian Xinhao | Sow farm | Qinghua Agriculture and Animal Husbandry | Sow farm |
| Kangping Xinwang | Sow farm | Danxian Xinhao | Sow farm | Laizhou Xinhai | Sow farm |
| Yinbao Breeding | Sow farm | Zhenjiang Xinhai | Sow farm | Weinan Xinliu | Sow farm |
| Yantai Xinhao | Sow farm | Dongying Xinhao | Sow farm | Zhenjiang Xinhai | Sow farm |
| Yantai Xinhao | Sow farm | Longzhou Xinhao | Sow farm | Santai Agriculture and Animal Husbandry | Sow farm |
| Dong'e Xinliu | Sow farm | Yantai Xinhao | Sow farm | Lingbao Xinliu | Sow farm |
| Dongying Xinhao | Sow farm | Santai Agriculture and Animal Husbandry | Sow farm | Heishan Gaojia | Sow farm |
| Kangping Xinwang | Sow farm | Qinghua Agriculture and Animal Husbandry | Sow farm | Longzhou Xinhao | Sow farm |
| Laizhou Xinhai | Sow farm | Yantai Xinhao | Sow farm | Danxian Xinliu | Sow farm |
| Shanxian Xinhao | Sow farm | Longzhou Xinhao | Sow farm | Longzhou Xinhao | Sow farm |
| Laizhou Xinhai | Sow farm | Juye Xinhao | Sow farm | Leshan Agriculture and Animal Husbandry | Sow farm |
| Heishan Xinliu | Sow farm | Heishan Xin Liu | Sow farm | Longzhou Xinhao | Sow farm |
| Jiangxi Xinliu | Sow farm | Nanning Xin Liu | Sow farm | Nanning Xinliu | Sow farm |
| Baiyin Nongmu | Sow farm | Heishan Xin Liu | Sow farm | Juye Xinhao | Sow farm |
| Shanxian Xinhao | Sow farm | Longzhou Xinhao | Sow farm | Hezhou Xinhao | Sow farm |
| Shanxian Xinhao | Sow farm | Hubei Xinhao | Sow farm | Shibing Xinliu | Sow farm |
| Shanxian Xinhao | Sow farm | Laizhou Xinhai | Sow farm | Longzhou Xinhao | Sow farm |
| Jiangxi Xinliu | Sow farm | Guangyuan Xinhao | Sow farm | Yantai Xinhao | Sow farm |
| Hubei Xinhao | Sow farm | Longzhou Xinhao | Sow farm | Hubei Xinhao | Sow farm |
| Juye Xinhao | Sow farm | Leshan Agriculture and Animal Husbandry | Sow farm | Zhangjiakou Xinwang | Sow farm |
| Guangyuan Xinhao | Sow farm | Hezhou Xinhao | Sow farm | Yan'an Benyuan | Sow farm |
| Longzhou Xinhao | Sow farm | Anyang Xin Liu | Sow farm | Heishan Gaojia | Sow farm |
| Yantai Xinhao | Sow farm | Yan'an Benyuan | Sow farm | Dong'e Xinliu | Sow farm |
| Longzhou Xinhao | Sow farm | Longzhou Xinhao | Sow farm | Guangyuan Xinhao | Sow farm |
| Yan'an Benyuan | Sow farm | Lingbao Xin Liu | Sow farm | Longzhou Xinhao | Sow farm |
| Longzhou Xinhao | Sow farm | Caoxian Xinhao | Sow farm | Liangshan Xinliu | Sow farm |
| Longzhou Xinhao | Sow farm | Ruyuan Xinhao | Sow farm | Zhenjiang Xinhai | Sow farm |
| Jiaxiang Xinliu | Sow farm | Longzhou Xinhao | Sow farm | Longzhou Xinhao | Sow farm |
| Yantai Xinhao | Sow farm | Shibing Xin Liu | Sow farm | Ruyuan Xinhao | Sow farm |
| Juye Xinhao | Sow farm | Liaoning Xintao Wang | Sow farm | Juye Xinhao | Sow farm |
| Longzhou Xinhao | Sow farm | Zhangjiakou Xinwang | Sow farm | Juye Xinhao | Sow farm |
| Qinghua Nongmu | Sow farm | Gansu Xinhao | Sow farm | Jiangxi Xinliu | Sow farm |
| Lingbao Xinliu | Sow farm | Yucheng Xin Liu | Sow farm | Longzhou Xinhao | Sow farm |
| Caoxian Xinhao | Sow farm | Longzhou Xinhao | Sow farm | Guangdong Xinhao | Sow farm |
| Laizhou Xinhai | Sow farm | Liangshan Xin Liu | Sow farm | Chenzhou Xinhao | Sow farm |
| Nanning Xinliu | Sow farm | Jiangxi Xin Liu | Sow farm | Liaocheng Xinhao | Sow farm |
| Leshan Nongmu | Sow farm | Chenzhou Xinhao | Sow farm | Zhangxin Wangchang | Sow farm |
| Zhangjiakou Xinwang | Sow farm | Guangdong Xinhao | Sow farm | Juye Xinhao | Sow farm |
| Santai Nongmu | Sow farm | Liaocheng Xinhao | Sow farm | Caoxian Xinhao | Sow farm |
| Shibing Xinliu | Sow farm | Zhenjiang Xinhai | Sow farm | Pucheng Xinliu | Sow farm |
| Baiyin Nongmu | Sow farm | Gansu Xinhao | Sow farm | Gansu Xinhao | Sow farm |
| Longzhou Xinhao | Sow farm | Liaoning Xinwang | Sow farm | Pingfu Agriculture and Animal Husbandry | Sow farm |
| Liaoning Xintao Wang | Sow farm | Santai Agriculture and Animal Husbandry | Sow farm | Santai Agriculture and Animal Husbandry | Sow farm |
| Kangping Xinwang | Sow farm | Pingfu Agriculture and Animal Husbandry | Sow farm | Dong'e Xinliu | Sow farm |
| Hezhou Xinhao | Sow farm | Jiangxi Xin Liu | Sow farm | Raoyang Xinhao | Sow farm |
| Santai Nongmu | Sow farm | Nanning Xinhao | Sow farm | Nanning Xinhao | Sow farm |
| Longzhou Xinhao | Sow farm | Pucheng Xin Liu | Sow farm | Zhongshan Agriculture and Animal Husbandry | Sow farm |
| Guangdong Xinhao | Sow farm | Meishan Xinhai | Sow farm | Yucheng Xin Liu | Sow farm |
| Gaotang Xinhao | Sow farm | Zhongshan Agriculture and Animal Husbandry | Sow farm | Dong'e Xin Liu | Sow farm |
| Kangping New Hope | Sow farm | Anyang Xin Liu | Sow farm | Heishan Gaojia | Sow farm |
| Ruyuan Xinhao | Sow farm | Gaotang Xin Hao | Sow farm | Heishan Gaojia | Sow farm |
| Pingfu Agriculture and Animal Husbandry | Sow farm | Liaoning Xin Wang | Sow farm | Changyi Xin Hao | Sow farm |
| Gansu Xinhao | Sow farm | Juye Xin Hao | Sow farm | Jiangyou Pig Farm | Sow farm |
| Weinan Xinliu | Sow farm | Jiangyou Pig Farm | Sow farm | Gaotang Xin Hao | Sow farm |
| Fuxin New Hope | Sow farm | Gaotang Xin Hao | Sow farm | Meishan Xin Hai | Sow farm |
| Baiyin Agriculture and Animal Husbandry | Sow farm | Heishan Xin Liu | Sow farm | Juye Xin Hao | Sow farm |
| Liaocheng Xinhao | Sow farm | Liaoning Xintao Wang | Sow farm | Liaoning Xin Wang | Sow farm |
| Liangshan Xinliu | Sow farm | Zhangjiakou Xin Wang | Sow farm | Wuhe Xin Hai | Sow farm |
| Baiyin Agriculture and Animal Husbandry | Sow farm | Wuhe Xin Hai | Sow farm | Wulian Xin Hao | Sow farm |
| Gaotang Xinhao | Sow farm | Shuozhou Xin Hao | Sow farm | Yucheng Xin Liu | Sow farm |
| Chenzhou Xinhao | Sow farm | Changyi Xin Hao | Sow farm | Guigang Xin Liu | Sow farm |
| Baiyin Agriculture and Animal Husbandry | Sow farm | Liaoning Xin Wang | Sow farm | Shuozhou Xin Hao | Sow farm |
| Nanning Xinhao | Sow farm | Liaoning Xin Wang | Sow farm | Wuhe Xin Hai | Sow farm |
| Anyang Xinliu | Sow farm | Guigang Xin Liu | Sow farm | Gaotang Xin Hao | Sow farm |
| Zhongshan Agriculture and Animal Husbandry | Sow farm | Heishan Xin Liu | Sow farm | Nanning Xin Liu | Sow farm |
| Jiangyou Pig Farm | Sow farm | Xiajin Nongmu | Sow farm | Xindongwan Xin Liu | Sow farm |
| Baiyin Agriculture and Animal Husbandry | Sow farm | Kangping Xin Wang | Sow farm | Xiajin Agro-Pastoral | Sow farm |
| Pucheng Xinliu | Sow farm | Wuhe Xin Hai | Sow farm | Wulian Xin Hao | Sow farm |
| Dong'e Xinliu | Sow farm | Wulian Xin Hao | Sow farm | Liaoning Xin Wang | Sow farm |
| Liaoning New Hope | Sow farm | Wulian Xin Hao | Sow farm | Liaoning Xin Wang | Sow farm |
| Heishan Xinliu | Sow farm | Yancheng Zhongtai | Sow farm | Liaoning Xin Wang | Sow farm |
| Tongliao Xinhao | Sow farm | Nanning Xin Liu | Sow farm | Tongliao Xin Hao | Sow farm |
| Taian Xinliu | Sow farm | Langzhong Xin Liu | Sow farm | Wuhe Xin Hai | Sow farm |
| Tongliao Xinhao | Sow farm | Liaoning Xin Wang | Sow farm | Tongliao Xin Hao | Sow farm |
| Wulian Xinhao | Sow farm | Xindongwan Xin Liu | Sow farm | Tongliao Xin Hao | Sow farm |
| Wuhe Xinhai | Sow farm | Shuozhou Xin Hao | Sow farm | Tongliao Xin Hao | Sow farm |
| Liaoning New Tao望 | Sow farm | Wuhe Xin Hai | Sow farm | Changyi Xin Hao | Sow farm |
| Wannian Xinhai | Sow farm | Dong'e Xin Liu | Sow farm | Liaoning Xintao Wang | Sow farm |
| Langzhong Xinliu | Sow farm | Dong'e Xin Liu | Sow farm | Zhangxin Wang Field | Sow farm |
| Changyi Xinhao | Sow farm | Langzhong Xin Liu | Sow farm | Langzhong Xin Liu | Sow farm |
| Dongying Xinhao | Sow farm | Taian Xin Liu | Sow farm | Xiajin Agro-Pastoral | Sow farm |
| Liaoning New Hope | Sow farm | Taian Xin Liu | Sow farm | Langzhong Xin Liu | Sow farm |
| Wuhe Xinhai | Sow farm | Wuhe Xin Hai | Sow farm | Shuozhou Xin Hao | Sow farm |
| Nanning Xinliu | Sow farm | Zhejiang Xin Hai | Sow farm | Taian Xin Liu | Sow farm |
| Shuozhou Xinhao | Sow farm | Taian Xin Liu | Sow farm | Wuhe Xin Hai | Sow farm |
| Wulian Xinhao | Sow farm | Tongliao Xin Hao | Sow farm | Dong'e Xin Liu | Sow farm |
| Changyi Xinhao | Sow farm | Tongliao Xin Hao | Sow farm | Dong'e Xin Liu | Sow farm |
| Liaoning New Hope | Sow farm | Xiajin Nongmu | Sow farm | Taian Xin Liu | Sow farm |
| Tongliao Xinhao | Sow farm | Tongliao Xin Hao | Sow farm | Taian Xin Liu | Sow farm |
| Yichun New Hope | Sow farm | Yingcheng Xin Hao | Sow farm | Shuozhou Xin Hao | Sow farm |
| Heishan Xinliu | Sow farm | Meishan Xin Hai | Sow farm | Meishan Xin Hai | Sow farm |
| Tongliao Xinhao | Sow farm | Tongliao Xin Hao | Sow farm | Jiangxi Xin Liu | Sow farm |
| Zhejiang Xinhai | Sow farm | Jiangxi Xin Liu | Sow farm | Heishan Gaojia | Sow farm |
| Langzhong Xinliu | Sow farm | Jiangxi Xin Liu | Sow farm | Meishan Xin Hai | Sow farm |
| Anyang Xinliu | Sow farm | Hengnan Muyun | Sow farm | Xiajin Agro-Pastoral | Sow farm |
| Wuhe Xinhai | Sow farm | Wuhe Xin Hai | Sow farm | Jiangxi Xin Liu | Sow farm |
| Guigang Xinliu | Sow farm | Wuhe Xin Hai | Sow farm | Zhangjiakou Xin Wang | Sow farm |
| Taian Xinliu | Sow farm | Xiajin Nongmu | Sow farm | Xiajin Agro-Pastoral | Sow farm |
| Shuozhou Xinhao | Sow farm | Shuozhou Xin Hao | Sow farm | Laixi Xin Liu | Sow farm |
| Guigang Xinliu | Sow farm | Laixi Xin Liu | Sow farm | Liaocheng Xin Hao | Sow farm |
| Meishan Xinhai | Sow farm | Meishan Xin Hai | Sow farm | Shuozhou Xin Hao | Sow farm |
| Meishan Xinhai | Sow farm | Lingbao Xin Liu | Sow farm | Wannian Xin Hai | Sow farm |
| Benxiang Li Jia | Sow farm | Xiajin Nongmu | Sow farm | Liaocheng Xin Hao | Sow farm |
| Liaoning New Tao望 | Sow farm | Wannian Xin Hai | Sow farm | Anhui Xin Liu | Sow farm |
| Taian Xinliu | Sow farm | Heishan Xin Liu | Sow farm | Kangping Xin Wang | Sow farm |
| Liaoning New Tao望 | Sow farm | Lingbao Xin Liu | Sow farm | Jiangxi Xin Liu | Sow farm |
| Juye Xinhao | Sow farm | Juye Xin Hao | Sow farm | Xiajin Agro-Pastoral | Sow farm |
| Laixi Xinliu | Sow farm | Juye Xin Hao | Sow farm | Shuozhou Xin Hao | Sow farm |
| Wannian Xinhai | Sow farm | Anhui Xin Liu | Sow farm | Zhejiang Xin Hai | Sow farm |
| Liaoning New Tao望 | Sow farm | Shuozhou Xin Hao | Sow farm | Wannian Xin Hai | Sow farm |
| Yingcheng Xinhao | Sow farm | Liaocheng Xin Hao | Sow farm | Shuozhou Xin Hao | Sow farm |
| Wuhe Xinhai | Sow farm | Jiangxi Xin Liu | Sow farm | Weinan Xin Liu | Sow farm |
| Dong'e Xinliu | Sow farm | Hengnan Muyun | Sow farm | Guigang Xin Liu | Sow farm |
| Hengnan Muyun | Sow farm | Yingcheng Xin Hao | Sow farm | Wuhe Xin Hai | Sow farm |
| Anhui Xinliu | Sow farm | Zhejiang Xin Hai | Sow farm | Wuhe Xin Hai | Sow farm |
| Lingbao Xinliu | Sow farm | Xiajin Nongmu | Sow farm | Laibin Xin Hao | Sow farm |
| Shuozhou Xinhao | Sow farm | Liaoning Xintao Wang | Sow farm | Lingbao Xin Liu | Sow farm |
| Yingcheng Xinhao | Sow farm | Weinan Xin Liu | Sow farm | Hengnan Muyun | Sow farm |
| Xiajin Agriculture and Animal Husbandry | Sow farm | Liaoning Xintao Wang | Sow farm | Wuqi Xin Liu | Sow farm |
| Hengnan Muyun | Sow farm | Changyi Xin Hao | Sow farm | Lingbao Xin Liu | Sow farm |
| Heishan Xinliu | Sow farm | Laixi Xin Liu | Sow farm | Liaoning Xintao Wang | Sow farm |
| Xiajin Agriculture and Animal Husbandry | Sow farm | Guigang Xin Liu | Sow farm | Zhangjiakou Xin Wang | Sow farm |
| Jiangxi Xinliu | Sow farm | Kangping Xin Wang | Sow farm | Jiangxi Xin Liu | Sow farm |
| Wuhe Xinhai | Sow farm | Shuozhou Xin Hao | Sow farm | Xiajin Agro-Pastoral | Sow farm |
| Jiangxi Xinliu | Sow farm | Shuozhou Xin Hao | Sow farm | Liaoning Xintao Wang | Sow farm |
| Lingbao Xinliu | Sow farm | Yichun Xin Wang | Sow farm | Santai Agro-Pastoral | Sow farm |
| Liaoning New Tao望 | Sow farm | Juye Xin Hao | Sow farm | Liaoning Xintao Wang | Sow farm |
| Juye Xinhao | Sow farm | Juye Xin Hao | Sow farm | Guangdong Xin Hao | Sow farm |
| Wuhe Xinhai | Sow farm | Liaoning Xintao Wang | Sow farm | Wuhe Xin Hai | Sow farm |
| Meishan Xinhai | Sow farm | Liaoning Xintao Wang | Sow farm | Zhejiang Xin Hai | Sow farm |
| Dongying Xinhao | Sow farm | Jiangxi Xin Liu | Sow farm | Wuqi Xin Liu | Sow farm |
| Zhejiang Xinhai | Sow farm | Taian Xin Liu | Sow farm | Laibin Xin Hao | Sow farm |
| Xiajin Agriculture and Animal Husbandry | Sow farm | Liaocheng Xin Hao | Sow farm | Guanling Xin Hai | Sow farm |
| Laiyang New Livestock | Sow farm | Guang'an Xin Hao | Sow farm | Yingcheng Xin Hao | Sow farm |
| Juye Xinhao | Sow farm | Wuhe Xin Hai | Sow farm | Yichun Xin Wang | Sow farm |
| Jiangxi Xinliu | Sow farm | Wuqi Xin Liu | Sow farm | Yanting Xin Hao | Sow farm |
| Xiajin Agriculture and Animal Husbandry | Sow farm | Kangping Xin Wang | Sow farm | Guangan Xin Hao | Sow farm |
| Wuhe Xinhai | Sow farm | Santai Nongmu | Sow farm | Laibin Xin Hao | Sow farm |
| Liaocheng Xinhao | Sow farm | Shuozhou Xin Hao | Sow farm | Guangyuan Xin Hao | Sow farm |
| Weinan Xinliu | Sow farm | Guangdong Xin Hao | Sow farm | Gansu Xin Hao | Sow farm |
| Jiangxi Xinliu | Sow farm | Wuqi Xin Liu | Sow farm | Yanting Xin Hao | Sow farm |
| Xiajin Agriculture and Animal Husbandry | Sow farm | Yichun Xin Wang | Sow farm | Ningming Xin Hao | Sow farm |
| Dong'e Xinliu | Sow farm | Laibin Xin Hao | Sow farm | Laibin Xin Hao | Sow farm |
| Guang'an Xinhao | Sow farm | Caoxian Xin Hao | Sow farm | Guanling Xin Hai | Sow farm |
| Shuozhou Xinhao | Sow farm | Heyang Xin Liu | Sow farm | Kangping Xin Wang | Sow farm |
| Xindongwan Xinliu | Sow farm | Guangyuan Xin Hao | Sow farm | Laibin Xin Hao | Sow farm |
| Heyang Xinliu | Sow farm | Liaocheng Xin Hao | Sow farm | Hubei Xin Hao | Sow farm |
| Pingfu Agriculture and Animal Husbandry | Sow farm | Xiajin Nongmu | Sow farm | Chenzhou Xin Hao | Sow farm |
| Heishan Wolniu | Sow farm | Dong'e Xin Liu | Sow farm | Taian Xin Liu | Sow farm |
| Liaocheng Xinhao | Sow farm | Bijie Xin Liu | Sow farm | Pingfu Agro-Pastoral | Sow farm |
| Shuozhou Xinhao | Sow farm | Yanting Xin Hao | Sow farm | Hubei Xin Hao | Sow farm |
| Juye Xinhao | Sow farm | Hengnan Muyun | Sow farm | Laixi Xin Liu | Sow farm |
| Kangping New Hope | Sow farm | Hubei Xin Hao | Sow farm | Laibin Xin Hao | Sow farm |
| Guigang Xinliu | Sow farm | Kangping Xin Wang | Sow farm | Dong'e Xin Liu | Sow farm |
| Guang'an Xinhao | Sow farm | Chenzhou Xin Hao | Sow farm | Hubei Xin Hao | Sow farm |
| Kangping New Hope | Sow farm | Hezhou Xin Hao | Sow farm | Chenzhou Xin Hao | Sow farm |
| Wuqi Xinliu | Sow farm | Guanling Xin Hai | Sow farm | Yan'an Benyuan | Sow farm |
| Shuozhou Xinhao | Sow farm | Guang'an Xin Hao | Sow farm | Hezhou Xin Hao | Sow farm |
| Guigang Xinliu | Sow farm | Caoxian Xin Hao | Sow farm | Leshan Agro-Pastoral | Sow farm |
| Guangyuan Xinhao | Sow farm | Hubei Xin Hao | Sow farm | Kangping Xin Wang | Sow farm |
| Caoxian Xinhao | Sow farm | Zhangjiakou Xin Wang | Sow farm | Yanting Xin Hao | Sow farm |
| Wuqi Xinliu | Sow farm | Guanling Xin Hai | Sow farm | Leshan Agro-Pastoral | Sow farm |
| Guigang Xinliu | Sow farm | Chenzhou Xin Hao | Sow farm | Hubei Xin Hao | Sow farm |
| Liaoning New Tao望 | Sow farm | Hubei Xin Hao | Sow farm | Hezhou Xin Hao | Sow farm |
| Hubei Xinhao | Sow farm | Laiyang Xin Hao | Sow farm | Yingcheng Xin Hao | Sow farm |
| Laibin Xinhao | Sow farm | Yanting Xin Hao | Sow farm | Hezhou Xin Hao | Sow farm |
| Caoxian Xinhao | Sow farm | Hubei Xin Hao | Sow farm | Hezhou Xin Hao | Sow farm |
| Santai Agriculture and Animal Husbandry | Sow farm | Chenzhou Xin Hao | Sow farm | Liaocheng Xin Hao | Sow farm |
| Hubei Xinhao | Sow farm | Laibin Xin Hao | Sow farm | Weinan Xin Liu | Sow farm |
| Shuozhou Xinhao | Sow farm | Ningming Xin Hao | Sow farm | Chenzhou Xin Hao | Sow farm |
| Laiyang Xinhao | Sow farm | Jiaxiang Xin Liu | Sow farm | Hubei Xin Hao | Sow farm |
| Caoxian Xinhao | Sow farm | Hezhou Xin Hao | Sow farm | Zhangjiakou Xin Wang | Sow farm |
| Hengnan Muyun | Sow farm | Laibin Xin Hao | Sow farm | Laibin Xinhao | Sow farm |
| Liaocheng Xin Hao | Sow farm | Laibin Xinhao | Sow farm | Hezhou Xinhao | Sow farm |
| Laibin Xin Hao | Sow farm | Bijie Xinliu | Sow farm | Hubei Xinhao | Sow farm |
| Zhangjiakou Xin Wang | Sow farm | Yingtan Xinliu | Sow farm | Bijie Xinliu | Sow farm |
| Pucheng Xin Liu | Sow farm | Yijun Xinliu | Sow farm | Jiaxiang Xinliu | Sow farm |
| Jiaxiang Xin Liu | Sow farm | Pingfu Agriculture and Animal Husbandry | Sow farm | Laibin Xinhao | Sow farm |
| Hezhou Xin Hao | Sow farm | Wannian Xinhai | Sow farm | Pucheng Xinliu | Sow farm |
| Yingcheng Xin Hao | Sow farm | Gansu Xinhao | Sow farm | Caoxian Xinhao | Sow farm |
| Yan'an Ben Yuan | Sow farm | Laibin Xinhao | Sow farm | Shibing Xinliu | Sow farm |
| Guangdong Xin Hao | Sow farm | Hezhou Xinhao | Sow farm | Ningming Xinhao | Sow farm |
| Hainan Nongken | Sow farm | Yan'an Benyuan | Sow farm | Liaoning New Taowang | Sow farm |
| Chenzhou Xin Hao | Sow farm | Caoxian Xinhao | Sow farm | Bijie Xinliu | Sow farm |
| Xiajin Nongmu | Sow farm | Laibin Xinhao | Sow farm | Yijun Xinliu | Sow farm |
| Guanling Xin Hai | Sow farm | Yanting Xinhao | Sow farm | Hubei Xinhao | Sow farm |
| Ningming Xin Hao | Sow farm | Hubei Xinhao | Sow farm | Hubei Xinhao | Sow farm |
| Hubei Xin Hao | Sow farm | Guangyuan Xinhao | Sow farm | Yingcheng Xinhao | Sow farm |
| Yingtan Xin Liu | Sow farm | Leshan Agriculture and Animal Husbandry | Sow farm | Yijun Xinliu | Sow farm |
| Gansu Xin Hao | Sow farm | Yanting Xinhao | Sow farm | Kangping Xinwang | Sow farm |
| Yingtan Xin Liu | Sow farm | Laibin Xinhao | Sow farm | Shibing Xinliu | Sow farm |
| Yijun Xin Liu | Sow farm | Hubei Xinhao | Sow farm | Shibing Xinliu | Sow farm |
| Hubei Xin Hao | Sow farm | Yijun Xinliu | Sow farm | Shibing Xinliu | Sow farm |
| Kangping Xin Wang | Sow farm | Leshan Agriculture and Animal Husbandry | Sow farm | Shibing Xinliu | Sow farm |
| Lingao Xin Liu | Sow farm | Ningming Xinhao | Sow farm | Caoxian Xinhao | Sow farm |
| Yingcheng Xin Hao | Sow farm | Pucheng Xinliu | Sow farm | Pingfu Agriculture and Animal Husbandry | Sow farm |
| Pingfu Nongmu | Sow farm | Hezhou Xinhao | Sow farm | Yanting Xinhao | Sow farm |
| Hezhou Xin Hao | Sow farm | Zhangjiakou Xinwang | Sow farm | Yingcheng Xinhao | Sow farm |
| Hubei Xin Hao | Sow farm | Hezhou Xinhao | Sow farm | Weinan Xinliu | Sow farm |
| Yichun Xin Wang | Sow farm | Guigang Xinliu | Sow farm | Yichun Xinwang | Sow farm |
| Chenzhou Xin Hao | Sow farm | Pingfu Agriculture and Animal Husbandry | Sow farm | Guigang Xinliu | Sow farm |
| Chenzhou Xin Hao | Sow farm | Hubei Xinhao | Sow farm | Pucheng Xinliu | Sow farm |
| Guangyuan Xin Hao | Sow farm | Shibing Xinliu | Sow farm | Guangan Xinhao | Sow farm |
| Zhangjiakou Xin Wang | Sow farm | Liaoning Xintao Wang | Sow farm | Hubei Xinhao | Sow farm |
| Laiyang Xin Hao | Sow farm | Yingcheng Xinhao | Sow farm | Hengnan Agriculture and Animal Husbandry | Sow farm |
| Hubei Xin Hao | Sow farm | Hubei Xinhao | Sow farm | Hubei Xinhao | Sow farm |
| Hubei Xin Hao | Sow farm | Pingfu Agriculture and Animal Husbandry | Sow farm | Jiaxiang Xinliu | Sow farm |
| Guanling Xin Hai | Sow farm | Gansu Xinhao | Sow farm | Yingtan Xinliu | Sow farm |
| Hubei Xin Hao | Sow farm | Xiangzhou Xinhao | Sow farm | Gansu Xinhao | Sow farm |
| Bijie Xin Liu | Sow farm | Weinan Xinliu | Sow farm | Pucheng Xinliu | Sow farm |
| Heyang Xin Liu | Sow farm | Weinan Xinliu | Sow farm | Xiangzhou Xinhao | Sow farm |
| Pucheng Xin Liu | Sow farm | Hainan Agriculture and Reclamation | Sow farm | Liaoning New Taowang | Sow farm |
| Kangping Xin Wang | Sow farm | Laiyang Xinhao | Sow farm | Laiyang Xinhao | Sow farm |
| Yijun Xin Liu | Sow farm | Ningming Xinhao | Sow farm | Wuhe Xinhai | Sow farm |
| Hezhou Xin Hao | Sow farm | Shibing Xinliu | Sow farm | Laiyang Xinhao | Sow farm |
| Hubei Xin Hao | Sow farm | Shibing Xinliu | Sow farm | Guangyuan Xinhao | Sow farm |
| Xiangzhou Xin Hao | Sow farm | Guigang Xinliu | Sow farm | Caoxian Xinhao | Sow farm |
| Hezhou Xin Hao | Sow farm | Kangping Xinwang | Sow farm | Pucheng Xinliu | Sow farm |
| Guigang Xin Liu | Sow farm | Hubei Xinhao | Sow farm | Gansu Xinliu | Sow farm |
| Leshan Nongmu | Sow farm | Guigang Xinliu | Sow farm | Guigang Xinliu | Sow farm |
| Laibin Xin Hao | Sow farm | Laibin Xinhao | Sow farm | Ningming Xinhao | Sow farm |
| Leshan Nongmu | Sow farm | Pucheng Xinliu | Sow farm | Ningming Xinhao | Sow farm |
| Laibin Xin Hao | Sow farm | Ningming Xinhao | Sow farm | Gansu Xinhao | Sow farm |
| Laibin Xin Hao | Sow farm | Xiangzhou Xinhao | Sow farm | Ningming Xinhao | Sow farm |
| Caoxian Xin Hao | Sow farm | Pucheng Xinliu | Sow farm | Nanning Xinhao | Sow farm |
| Yan'an Ben Yuan | Sow farm | Yan'an Benyuan | Sow farm | Yijun Xinliu | Sow farm |
| Yanting Xin Hao | Sow farm | Shibing Xinliu | Sow farm | Hengnan Agriculture and Animal Husbandry | Sow farm |
| Shibing Xin Liu | Sow farm | Nanning Xinhao | Sow farm | Heyang Xinliu | Sow farm |
| Hezhou Xin Hao | Sow farm | Pucheng Xinliu | Sow farm | Nanning Xinhao | Sow farm |
| Pingfu Nongmu | Sow farm | Weinan Xinliu | Sow farm | Guangdong Xinhao | Sow farm |
| Pucheng Xin Liu | Sow farm | Yingcheng Xinhao | Sow farm | Lingxin Xinliu | Sow farm |
| Yan'an Ben Yuan | Sow farm | Heyang Xinliu | Sow farm | Nanning Xinhao | Sow farm |
| Hainan Nongken | Sow farm | Nanning Xinhao | Sow farm | Yingcheng Xinhao | Sow farm |
| Yingcheng Xin Hao | Sow farm | Nanning Xinhao | Sow farm | Weinan Xinliu | Sow farm |
| Ningming Xin Hao | Sow farm | Hubei Xinhao | Sow farm | Yan'an Benyuan | Sow farm |
| Pingfu Nongmu | Sow farm | Jiaxiang Xinliu | Sow farm | Pingfu Agriculture and Animal Husbandry | Sow farm |
| Yingcheng Xin Hao | Sow farm | Guangyuan Xinhao | Sow farm | Guangdong Xinhao | Sow farm |
| Shibing Xin Liu | Sow farm | Yingcheng Xinhao | Sow farm | Guangyuan Xinhao | Sow farm |
| Shibing Xin Liu | Sow farm | Shibing Xinliu | Sow farm | Gansu Xinhao | Sow farm |
| Xiangzhou Xin Hao | Sow farm | Ningming Xinhao | Sow farm | Ningming Xinhao | Sow farm |
| Yanting Xin Hao | Sow farm | Nanning Xinliu | Sow farm | Qinghua Agriculture and Animal Husbandry | Sow farm |
| Laibin Xin Hao | Sow farm | Gansu Xinhao | Sow farm | Guangdong Xinhao | Sow farm |
| Nanning Xin Hao | Sow farm | Guangdong Xinhao | Sow farm | Pingfu Agriculture and Animal Husbandry | Sow farm |
| Gansu Xin Hao | Sow farm | Ningming Xinhao | Sow farm | Guigang Xinliu | Sow farm |
| Shibing Xin Liu | Sow farm | Guangdong Xinhao | Sow farm | Yingcheng Xinhao | Sow farm |
| Gansu Xin Liu | Sow farm | Guangdong Xinhao | Sow farm | Hainan Agriculture and Forestry | Sow farm |
| Guangyuan Xin Hao | Sow farm | Gansu Xinliu | Sow farm | Nanning Xinliu | Sow farm |
| Shibing Xin Liu | Sow farm | Yan'an Benyuan | Sow farm | Guangdong Xinhao | Sow farm |
| Yanting Xin Hao | Sow farm | Hainan Agriculture and Reclamation | Sow farm | Yan'an Benyuan | Sow farm |
| Gansu Xin Hao | Sow farm | Yingtan Xinliu | Sow farm | Ningming Xinhao | Sow farm |
| Zhangjiakou Xin Wang | Sow farm | Liaocheng Xinhao | Sow farm | Xiangzhou Xinhao | Sow farm |
| Weinan Xin Liu | Sow farm | Caoxian Xinhao | Sow farm | Gansu Xinhao | Sow farm |
| Laibin Xin Hao | Sow farm | Wuhe Xinhai | Sow farm | Gansu Xinhao | Sow farm |
| Bijie Xin Liu | Sow farm | Gansu Xinhao | Sow farm | Pingfu Agriculture and Animal Husbandry | Sow farm |
| Nanning Xin Liu | Sow farm | Yingcheng Xinhao | Sow farm | Xiangzhou Xinhao | Sow farm |
| Weinan Xin Liu | Sow farm | Lingao Xinliu | Sow farm | Ruyuan Xinhao | Sow farm |
| Ningming Xin Hao | Sow farm | Ruyuan Xinhao | Sow farm | Qinghua Agriculture and Animal Husbandry | Sow farm |
| Xiangzhou Xin Hao | Sow farm | Ningming Xinhao | Sow farm | Gansu Xinhao | Sow farm |
| Guangdong Xin Hao | Sow farm | Qinghua Agriculture and Animal Husbandry | Sow farm | Guigang Xinliu | Sow farm |
| Gansu Xin Hao | Sow farm | Yijun Xinliu | Sow farm | Gansu Xinhao | Sow farm |
| Ningming Xin Hao | Sow farm | Guigang Xinliu | Sow farm | Pingfu Agriculture and Animal Husbandry | Sow farm |
| Nanning Xin Hao | Sow farm | Guangdong Xinhao | Sow farm | Nanning Xinhao | Sow farm |
| Guangdong Xin Hao | Sow farm | Pingfu Agriculture and Animal Husbandry | Sow farm | Hainan Agriculture and Forestry | Sow farm |
| Liaocheng Xin Hao | Sow farm | Hainan Xinliu | Sow farm | Zhaoqing Xinhao | Sow farm |
| Gansu Xin Hao | Sow farm | Xiangzhou Xinhao | Sow farm | Liaocheng Xinhao | Sow farm |
| Pucheng Xin Liu | Sow farm | Lingao Xinliu | Sow farm | Caoxian Xinhao | Sow farm |
| Hubei Xin Hao | Sow farm | Pingfu Agriculture and Animal Husbandry | Sow farm | Gansu Xinhao | Sow farm |
| Yanting Xin Hao | Sow farm | Ruyuan Xinhao | Sow farm | Gansu Xinhao | Sow farm |
| Xiangzhou Xin Hao | Sow farm | Gansu Xinliu | Sow farm | Yingtan Xinliu | Sow farm |
| Nanning Xin Hao | Sow farm | Nanning Xinhao | Sow farm | Yingcheng Xinhao | Sow farm |
| Gansu Xin Hao | Sow farm | Gansu Xinhao | Sow farm | Heyang Xinliu | Sow farm |
| Laibin Xin Hao | Sow farm | Xiangzhou Xinhao | Sow farm | Zhaoqing Xinhao | Sow farm |
| Lingao Xin Liu | Sow farm | Qinghua Agriculture and Animal Husbandry | Sow farm | Xiangzhou Xinhao | Sow farm |
| Pingfu Nongmu | Sow farm | Xiangzhou Xinhao | Sow farm | Hainan Xinliu | Sow farm |
| Wuhe Xin Hai | Sow farm | Hainan Xinliu | Sow farm | Ruyuan Xinhao | Sow farm |
| Yijun Xin Liu | Sow farm | Gansu Xinhao | Sow farm | Gansu Xinhao | Sow farm |
| Gansu Xin Hao | Sow farm | Pingfu Agriculture and Animal Husbandry | Sow farm | Hainan Xinliu | Sow farm |
| Ningming Xin Hao | Sow farm | Zhaoqing Xinhao | Sow farm | Ningbo Xinhai | Sow farm |
| Guangdong Xin Hao | Sow farm | Benxiang Lijia | Sow farm | Ningbo Xinhai | Sow farm |
| Pingfu Nongmu | Sow farm | Laiyang Xintu | Sow farm | Xiangzhou Xinhao | Sow farm |
| Ningming Xin Hao | Sow farm | Ningbo Xinhai | Sow farm | Lingxin Xinliu | Sow farm |
| Weinan Xin Liu | Sow farm | Gansu Xinhao | Sow farm | Zhaoqing Xinhao | Sow farm |
| Gansu Xin Hao | Sow farm | Ningbo Xinhai | Sow farm | Zhaoqing Xinhao | Sow farm |
| Jiaxiang Xin Liu | Sow farm | Gansu Xinhao | Sow farm |  |  |
| Gansu Xin Hao | Sow farm | Yingcheng Xinhao | Sow farm |  |  |
| Shenze Xin Hao | Sow farm | Zhaoqing Xinhao | Sow farm |  |  |
| Qinghua Nongmu | Sow farm | Zhaoqing Xinhao | Sow farm |  |  |
| Xiangzhou Xin Hao | Sow farm | Zhaoqing Xinhao | Sow farm |  |  |
| Ningming Xin Hao | Sow farm | Gansu Xinhao | Sow farm |  |  |
| Gansu Xin Hao | Sow farm | Gansu Xinhao | Sow farm |  |  |
| Yingcheng Xin Hao | Sow farm | Xiajiang Xinliu | Sow farm |  |  |
| Hainan Xin Liu | Sow farm | Gansu Xinhao | Sow farm |  |  |
| Guangdong Xin Hao | Sow farm | Shenze Xinhao | Sow farm |  |  |
| Gansu Xin Liu | Sow farm | Laiyang Xintu | Sow farm |  |  |
| Ruyuan Xin Hao | Sow farm | Hubei Xinhao | Sow farm |  |  |
| Nanning Xin Hao | Sow farm | Xianyang Yongxiang | Sow farm |  |  |
| Ruyuan Xin Hao | Sow farm | Laiyang Xintu | Sow farm |  |  |
| Ningbo Xin Hai | Sow farm | Yan'an Benyuan | Sow farm |  |  |
| Zhaoqing Xin Hao | Sow farm | Liaoning Xintao Wang | Sow farm |  |  |
| Ningbo Xin Hai | Sow farm | Linyi Zhongzhu | Sow farm |  |  |
| Qinghua Nongmu | Sow farm | Benxiang Lijia | Sow farm |  |  |
| Zhaoqing Xin Hao | Sow farm |  |  |  |  |
| Zhaoqing Xin Hao | Sow farm |  |  |  |  |
| Hainan Xin Liu | Sow farm |  |  |  |  |
| Zhaoqing Xin Hao | Sow farm |  |  |  |  |
| Hubei Xin Hao | Sow farm |  |  |  |  |
| Xiajiang Xin Liu | Sow farm |  |  |  |  |
| Gansu Xin Hao | Sow farm |  |  |  |  |
| Laiyang Xin Mu | Sow farm |  |  |  |  |
| Xianyang Yongxiang | Sow farm |  |  |  |  |
| Laiyang Xin Mu | Sow farm |  |  |  |  |
| Yan'an Ben Yuan | Sow farm |  |  |  |  |
| Yancheng Zhongtai | Sow farm |  |  |  |  |
| Huanghua Xin Hao | Sow farm |  |  |  |  |
| Huairou Nongmu | Sow farm |  |  |  |  |
| Laiyang Xin Hao | Sow farm |  |  |  |  |
| Laibin Xin Hao | Sow farm |  |  |  |  |
| Yan'an Ben Yuan | Sow farm |  |  |  |  |
| Laibin Xin Hao | Sow farm |  |  |  |  |
| Laiyang Xin Hao | Sow farm |  |  |  |  |
| Hebei Xin Hao | Sow farm |  |  |  |  |
| Hubei Xin Hao | Sow farm |  |  |  |  |
| Dong'e Xin Liu | Sow farm |  |  |  |  |
| Laibin Xin Hao | Sow farm |  |  |  |  |
| Caoxian Xin Hao | Sow farm |  |  |  |  |
| Huaiyin Xin Chi | Sow farm |  |  |  |  |
| Xiajin Nongmu | Sow farm |  |  |  |  |
| Weinan Xin Liu | Sow farm |  |  |  |  |
| Jiaxiang Xin Liu | Sow farm |  |  |  |  |
| Liaocheng Xin Hao | Sow farm |  |  |  |  |
| Zhangwu Xin Wang | Sow farm |  |  |  |  |
| Hebei Xin Hao | Sow farm |  |  |  |  |
| Juye Xin Hao | Sow farm |  |  |  |  |
| **Jul 2022** | | **Aug 2022** | | **Sep 2022** | |
| **Company (Co., Ltd.)** | **Type of pig farm** | **Company (Co., Ltd.)** | **Type of pig farm** | **Company (Co., Ltd.)** | **Type of pig farm** |
| Fuxin Xinwang | Fattening Farm | Laibin Breeding | Sow farm | Dongying Xinhao | Sow farm |
| Xinjin Xinhao | Sow farm | Meishan Xinhai | Sow farm | Tongliao Xinhao | Sow farm |
| Wannian Xinhai | Sow farm | Chenzhou Xinhao | Sow farm | Jiangxi Xinliu | Sow farm |
| Beijing Xinliu | Sow farm | Guanling Xinhai | Sow farm | Laibin Breeding | Sow farm |
| Anyang Xinliu | Sow farm | Beijing Xinliu | Sow farm | Chenzhou Xinhao | Sow farm |
| Guanling Xinhai | Sow farm | Laizhou Xinhai | Sow farm | Anyang Xinliu | Sow farm |
| Shuozhou Xinhao | Sow farm | Fuxin New Hope | Fattening farm | Beijing Xinliu | Sow farm |
| Zhangxin Wangchang | Sow farm | Dingzhou Xinhao | Sow farm | Fuxin Xinwang | Fattening farm |
| Tianjin Nongmu | Sow farm | Anyang Xinliu | Sow farm | Xingren Xinliu | Sow farm |
| Dingzhou Xinhao | Sow farm | Anyang Xinliu | Sow farm | Xindongwan Xinliu | Sow farm |
| Juye Xinhao | Sow farm | Dongying Xinhao | Sow farm | Xingren Xinliu | Sow farm |
| Meishan Xinhai | Sow farm | Xindongwan Xinliu | Sow farm | Gansu Xinhao | Sow farm |
| Tianjin Nongmu | Sow farm | Gansu Xinhao | Sow farm | Gansu Xinhao | Sow farm |
| Anyang Xinliu | Sow farm | Dongying Xinhao | Sow farm | Weinan Xinliu | Sow farm |
| Dongying Xinhao | Sow farm | Dongying Xinhao | Sow farm | Juye Xinhao | Sow farm |
| Anyang Xinliu | Sow farm | Wannian Xinhai | Sow farm | Juye Xinhao | Sow farm |
| Rugao Xinhao | Sow farm | Beijing Xinliu | Sow farm | Liuzhou Xinliu | Sow farm |
| Beijing Xinliu | Sow farm | Gansu Xinhao | Sow farm | Weinan Xinliu | Sow farm |
| Tianjin Nongmu | Sow farm | Tongliao Xinhao | Sow farm | Laibin Breeding | Sow farm |
| Dongying Xinhao | Sow farm | Tongliao Xinhao | Sow farm | Laizhou Xinhai | Sow farm |
| Zhenjiang Xinhai | Sow farm | Jiangxi Xinliu | Sow farm | Shuozhou Xinhao | Sow farm |
| Fuxin Xinwang | Sow farm | Xinjin Xinhao | Sow farm | Liuzhou Xinliu | Sow farm |
| Tongliao Xinhao | Sow farm | Weinan Xinliu | Sow farm | Tongliao Xinhao | Sow farm |
| Gansu Xinhao | Sow farm | Xiajiang Xinliu | Sow farm | Suining Xinliu | Sow farm |
| Xiajiang Xinliu | Sow farm | Juye Xinhao | Sow farm | Liuzhou Xinliu | Sow farm |
| Fuxin Xinwang | Sow farm | Zhangjiakou New Hope | Sow farm | Beijing Xinliu | Sow farm |
| Zhangjiakou Xinwang | Sow farm | Guanling Xinhai | Sow farm | Laibin Xinhao | Sow farm |
| Juye Xinhao | Sow farm | Zhenjiang Xinhai | Sow farm | Chenzhou Xinhao | Sow farm |
| Tianjin Nongmu | Sow farm | Juye Xinhao | Sow farm | Lezhi Xinhai | Sow farm |
| Laibin Yuzhong | Sow farm | Guangan Xinhao | Sow farm | Chenzhou Xinhao | Sow farm |
| Suining Xinliu | Sow farm | Langzhong Xinliu | Sow farm | Laiyang Xinmu | Fattening farm |
| Dongying Xinhao | Sow farm | Gansu Xinliu | Sow farm | Chenzhou Xinhao | Sow farm |
| Guanling Xinhai | Sow farm | Tianjin Agriculture and Animal Husbandry | Sow farm | Wannian Xinhai | Sow farm |
| Jiangxi Xinliu | Sow farm | Tianjin Agriculture and Animal Husbandry | Sow farm | Xiajiang Xinliu | Sow farm |
| Guangan Xinhao | Sow farm | Laiyang New Livestock | Fattening farm | Lezhi Xinhai | Sow farm |
| Langzhong Xinliu | Sow farm | Laizhou Xinhai | Sow farm | Xindongwan Xinliu | Sow farm |
| Wuqi Xinliu | Sow farm | Dongying Xinhao | Sow farm | Nanning Xinliu | Sow farm |
| Shuozhou Xinhao | Sow farm | Suining Xinliu | Sow farm | Zhenjiang Xinhai | Sow farm |
| Dongying Xinhao | Sow farm | Wuqi Xinliu | Sow farm | Langzhong Xinliu | Sow farm |
| Laizhou Xinhai | Sow farm | Tianjin Agriculture and Animal Husbandry | Sow farm | Guanling Xinhai | Sow farm |
| Tianjin Nongmu | Sow farm | Laizhou Xinhai | Sow farm | Guang'an Xinhao | Sow farm |
| Wuhe Xinhai | Sow farm | Fuxin New Hope | Sow farm | Gansu Xinliu | Sow farm |
| Gansu Xinliu | Sow farm | Hainan Agriculture and Reclamation | Sow farm | Laizhou Xinhai | Sow farm |
| Laixi Xinliu | Sow farm | Pingfu Agriculture and Animal Husbandry | Sow farm | Dongying Xinhao | Sow farm |
| Laizhou Xinhai | Sow farm | Zhejiang Xinhai | Sow farm | Jingxian Xinhao | Sow farm |
| Tongliao Xinhao | Sow farm | Yichun New Hope | Sow farm | Xinjin Xinhao | Sow farm |
| Pingfu Nongmu | Sow farm | Ningbo Xinhai | Sow farm | Lezhi Xinhai | Sow farm |
| Hainan Nongken | Sow farm | Hainan Xinliu | Sow farm | Wuqi Xinliu | Sow farm |
| Raoyang Xinhao | Sow farm | Raoyang Xinhao | Sow farm | Laizhou Xinhai | Sow farm |
| Hainan Xinliu | Sow farm | Laixi Xinliu | Sow farm | Hainan State Farms | Sow farm |
| Zhejiang Xinhai | Sow farm | Yingtan Xinliu | Sow farm | Zhejiang Xinhai | Sow farm |
| Juye Xinhao | Sow farm | Nanning Xinliu | Sow farm | Pingfu Nongmu | Sow farm |
| Ningbo Xinhai | Sow farm | Xindongwan Xinliu | Sow farm | Ningbo Xinhai | Sow farm |
| Xiajin Nongmu | Sow farm | Dong'e Xinliu | Sow farm | Jingxian Xinhao | Sow farm |
| Xiajin Nongmu | Sow farm | Taian Xinliu | Sow farm | Yichun Xinwang | Sow farm |
| Yichun Xinwang | Sow farm | Tianjin Agriculture and Animal Husbandry | Sow farm | Suining Xinliu | Sow farm |
| Yingtan Xinliu | Sow farm | Wuhe Xinhai | Sow farm | Hainan Xinliu | Sow farm |
| Taian Xinliu | Sow farm | Shuozhou Xinhao | Sow farm | Yingtan Xinliu | Sow farm |
| Xindongwan Xinliu | Sow farm | Rongchang Pig Farm | Sow farm | Jingxian Xinhao | Sow farm |
| Nanning Xinliu | Sow farm | Tianjin Agriculture and Animal Husbandry | Sow farm | Laixi Xinliu | Sow farm |
| Gansu Xinhao | Sow farm | Fuxin New Hope | Sow farm | Raoyang Xinhao | Sow farm |
| Zhangxin Wangchang | Sow farm | Juye Xinhao | Sow farm | Dong'e Xinliu | Sow farm |
| Tongliao Xinhao | Sow farm | Tongliao Xinhao | Sow farm | Tianjin Nongmu | Sow farm |
| Zhangxin Wangchang | Sow farm | Laizhou Xinhai | Sow farm | Shuozhou Xinhao | Sow farm |
| Dongjiang Xinliu | Sow farm | Rugao Xinhao | Sow farm | Tianjin Nongmu | Sow farm |
| Rongchang Zhuchang | Sow farm | Laibin Breeding | Sow farm | Juye Xinhao | Sow farm |
| Xiajin Nongmu | Sow farm | Yantai Xinhao | Sow farm | Rongchang Pig Farm | Sow farm |
| Changyi Xinhao | Sow farm | Laizhou Xinhai | Sow farm | Meishan Xinhai | Sow farm |
| Dongying Xinhao | Sow farm | Yinbao Breeding | Sow farm | Zhangjiakou Xinwang | Sow farm |
| Yantai Xinhao | Sow farm | Yantai Xinhao | Sow farm | Changyi Xinhao | Sow farm |
| Yantai Xinhao | Sow farm | Laizhou Xinhai | Sow farm | Shuozhou Xinhao | Sow farm |
| Laizhou Xinhai | Sow farm | Laizhou Xinhai | Sow farm | Laizhou Xinhai | Sow farm |
| Yantai Xinhao | Sow farm | Laizhou Xinhai | Sow farm | Tianjin Nongmu | Sow farm |
| Zhenjiang Xinhai | Sow farm | Laizhou Xinhai | Sow farm | Fuxin Xinwang | Sow farm |
| Liaoning Xinwang | Sow farm | Yantai Xinhao | Sow farm | Laizhou Xinhai | Sow farm |
| Juye Xinhao | Sow farm | Zhenjiang Xinhai | Sow farm | Taian Xinliu | Sow farm |
| Fuxin Xinwang | Sow farm | Laizhou Xinhai | Sow farm | Tianjin Nongmu | Sow farm |
| Yantai Xinhao | Sow farm | Juye Xinhao | Sow farm | Laizhou Xinhai | Sow farm |
| Laizhou Xinhai | Sow farm | Wuhe Xinhai | Sow farm | Yinbao Breeding | Sow farm |
| Laizhou Xinhai | Sow farm | Zhenjiang Xinhai | Sow farm | Wuhe Xinhai | Sow farm |
| Laizhou Xinhai | Sow farm | Shuozhou Xinhao | Sow farm | Laizhou Xinhai | Sow farm |
| Xindongwan Xinliu | Sow farm | Dongying Xinhao | Sow farm | Juye Xinhao | Sow farm |
| Laizhou Xinhai | Sow farm | Zhenjiang Xinhai | Sow farm | Laizhou Xinhai | Sow farm |
| Yinbao Breeding | Sow farm | Yantai Xinhao | Sow farm | Laizhou Xinhai | Sow farm |
| Laizhou Xinhai | Sow farm | Zhenjiang Xinhai | Sow farm | Yantai Xinhao | Sow farm |
| Zhenjiang Xinhai | Sow farm | Laizhou Xinhai | Sow farm | Laizhou Xinhai | Sow farm |
| Zhenjiang Xinhai | Sow farm | Ruyuan Xinhao | Sow farm | Ruyuan Xinhao | Sow farm |
| Zhenjiang Xinhai | Sow farm | Yantai Xinhao | Sow farm | Dongying Xinhao | Sow farm |
| Yantai Xinhao | Sow farm | Liaoning New Hope | Sow farm | Zhangjiakou Xinwang | Sow farm |
| Wuhe Xinhai | Sow farm | Dongying Xinhao | Sow farm | Wuhe Xinhai | Sow farm |
| Dongying Xinhao | Sow farm | Xiajin Agriculture and Animal Husbandry | Sow farm | Laibin Breeding | Sow farm |
| Juye Xinhao | Sow farm | Yantai Xinhao | Sow farm | Laizhou Xinhai | Sow farm |
| Laizhou Xinhai | Sow farm | Qinghua Agriculture and Animal Husbandry | Sow farm | Dongying Xinhao | Sow farm |
| Dongying Xinhao | Sow farm | Changyi Xinhao | Sow farm | Yantai Xinhao | Sow farm |
| Yantai Xinhao | Sow farm | Leshan Agriculture and Animal Husbandry | Sow farm | Yantai Xinhao | Sow farm |
| Qinghua Nongmu | Sow farm | Xiajin Agriculture and Animal Husbandry | Sow farm | Zhenjiang Xinhai | Sow farm |
| Lingbao Xinliu | Sow farm | Lingbao Xinliu | Sow farm | Hubei Xinhao | Sow farm |
| Heishan Gaojia | Sow farm | Danxian Xinhao | Sow farm | Weinan Xinliu | Sow farm |
| Jiaxiang Xinliu | Sow farm | Xiajin Agriculture and Animal Husbandry | Sow farm | Santai Nongmu | Sow farm |
| Shanxian Xinhao | Sow farm | Zhangjiakou New Hope | Sow farm | Lingbao Xinliu | Sow farm |
| Laizhou Xinhai | Sow farm | Heishan Gaojia | Sow farm | Shanxian Xinhao | Sow farm |
| Shanxian Xinhao | Sow farm | Hubei Xinhao | Sow farm | Shibing Xinliu | Sow farm |
| Zhangjiakou Xinwang | Sow farm | Danxian Xinhao | Sow farm | Yantai Xinhao | Sow farm |
| Kangping Xinwang | Sow farm | Danxian Xinhao | Sow farm | Heishan Gaojia | Sow farm |
| Leshan Nongmu | Sow farm | Dongying Xinhao | Sow farm | Dongying Xinhao | Sow farm |
| Hubei Xinhao | Sow farm | Kangping New Hope | Sow farm | Leshan Nongmu | Sow farm |
| Yantai Xinhao | Sow farm | Shibing Xinliu | Sow farm | Tongliao Xinhao | Sow farm |
| Shanxian Xinhao | Sow farm | Santai Agriculture and Animal Husbandry | Sow farm | Qinghua Nongmu | Sow farm |
| Shibing Xinliu | Sow farm | Yantai Xinhao | Sow farm | Zhenjiang Xinhai | Sow farm |
| Longzhou Xinhao | Sow farm | Jiaxiang Xinliu | Sow farm | Chenzhou Xinhao | Sow farm |
| Weinan Xinliu | Sow farm | Pucheng Xinliu | Sow farm | Guigang Xinliu | Sow farm |
| Dongying Xinhao | Sow farm | Chenzhou Xinhao | Sow farm | Shanxian Xinhao | Sow farm |
| Longzhou Xinhao | Sow farm | Guangdong Xinhao | Sow farm | Zhenjiang Xinhai | Sow farm |
| Yantai Xinhao | Sow farm | Longzhou Xinhao | Sow farm | Jiaxiang Xinliu | Sow farm |
| Hezhou Xinhao | Sow farm | Dongying Xinhao | Sow farm | Longzhou Xinhao | Sow farm |
| Yantai Xinhao | Sow farm | Yantai Xinhao | Sow farm | Guangdong Xinhao | Sow farm |
| Longzhou Xinhao | Sow farm | Weinan Xinliu | Sow farm | Shanxian Xinhao | Sow farm |
| Santai Nongmu | Sow farm | Hezhou Xinhao | Sow farm | Pucheng Xinliu | Sow farm |
| Guangyuan Xinhao | Sow farm | Guangyuan Xinhao | Sow farm | Kangping Xinwang | Sow farm |
| Longzhou Xinhao | Sow farm | Liangshan Xinliu | Sow farm | Yantai Xinhao | Sow farm |
| Chenzhou Xinhao | Sow farm | Longzhou Xinhao | Sow farm | Yantai Xinhao | Sow farm |
| Ruyuan Xinhao | Sow farm | Pingfu Agriculture and Animal Husbandry | Sow farm | Zhenjiang Xinhai | Sow farm |
| Pucheng Xinliu | Sow farm | Gansu Xinhao | Sow farm | Hezhou Xinhao | Sow farm |
| Longzhou Xinhao | Sow farm | Longzhou Xinhao | Sow farm | Nanning Xinliu | Sow farm |
| Nanning Xinliu | Sow farm | Longzhou Xinhao | Sow farm | Guangyuan Xinhao | Sow farm |
| Longzhou Xinhao | Sow farm | Longzhou Xinhao | Sow farm | Pingfu Nongmu | Sow farm |
| Gaotang Xinhao | Sow farm | Yantai Xinhao | Sow farm | Liangshan Xinliu | Sow farm |
| Yan'an Benyuan | Sow farm | Yan'an Benyuan | Sow farm | Yantai Xinhao | Sow farm |
| Liangshan Xinliu | Sow farm | Longzhou Xinhao | Sow farm | Longzhou Xinhao | Sow farm |
| Shanxian Xinhao | Sow farm | Danxian Xinhao | Sow farm | Jiangxi Xinliu | Sow farm |
| Guangdong Xinhao | Sow farm | Nanning Xinliu | Sow farm | Longzhou Xinhao | Sow farm |
| Jiangxi Xinliu | Sow farm | Caoxian Xinhao | Sow farm | Gansu Xinhao | Sow farm |
| Pingfu Nongmu | Sow farm | Longzhou Xinhao | Sow farm | Longzhou Xinhao | Sow farm |
| Longzhou Xinhao | Sow farm | Jiangxi Xinliu | Sow farm | Yantai Xinhao | Sow farm |
| Gansu Xinhao | Sow farm | Zhangxin Wangchang | Sow farm | Yan'an Benyuan | Sow farm |
| Caoxian Xinhao | Sow farm | Santai Agriculture and Animal Husbandry | Sow farm | Yantai Xinhao | Sow farm |
| Liaocheng Xinhao | Sow farm | Gaotang Xinhao | Sow farm | Longzhou Xinhao | Sow farm |
| Nanning Xinhao | Sow farm | Meishan Xinhai | Sow farm | Caoxian Xinhao | Sow farm |
| Santai Nongmu | Sow farm | Nanning Xinhao | Sow farm | Longzhou Xinhao | Sow farm |
| Meishan Xinhai | Sow farm | Liaocheng Xinhao | Sow farm | Shanxian Xinhao | Sow farm |
| Raoyang Xinhao | Sow farm | Zhangxin Wangchang | Sow farm | Longzhou Xinhao | Sow farm |
| Zhongshan Nongmu | Sow farm | Zhongshan Agriculture and Animal Husbandry | Sow farm | Dongying Xinhao | Sow farm |
| Heishan Gaojia | Sow farm | Raoyang Xinhao | Sow farm | Nanning Xinhao | Sow farm |
| Changyi Xinhao | Sow farm | Fuxin New Hope | Sow farm | Santai Nongmu | Sow farm |
| Heishan Gaojia | Sow farm | Guigang Xinliu | Sow farm | Tianjin Nongmu | Sow farm |
| Jiangyou Zhuchang | Sow farm | Jiangyou Pig Farm | Sow farm | Liaocheng Xinhao | Sow farm |
| Dong'a Xinliu | Sow farm | Heishan Gaojia | Sow farm | Fuxin Xinwang | Sow farm |
| Dong'a Xinliu | Sow farm | Suining Xinliu | Sow farm | Gaotang Xinhao | Sow farm |
| Suining Xinliu | Sow farm | Changyi Xinhao | Sow farm | Xiajin Nongmu | Sow farm |
| Heishan Gaojia | Sow farm | Shuozhou Xinhao | Sow farm | Zhongshan Nongmu | Sow farm |
| Guigang Xinliu | Sow farm | Changyi Xinhao | Sow farm | Shuozhou Xinhao | Sow farm |
| Wuhe Xinhai | Sow farm | Heishan Gaojia | Sow farm | Liaoning Xinwang | Sow farm |
| Shuozhou Xinhao | Sow farm | Wuhe Xinhai | Sow farm | Jiangyou Pig Farm | Sow farm |
| Gaotang Xinhao | Sow farm | Shuozhou Xinhao | Sow farm | Shuozhou Xinhao | Sow farm |
| Shuozhou Xinhao | Sow farm | Heishan Gaojia | Sow farm | Weinan Xinliu | Sow farm |
| Linyi Zhongzhu | Sow farm | Shuozhou Xinhao | Sow farm | Xiajin Nongmu | Sow farm |
| Wulian Xinhao | Sow farm | Wulian Xinhao | Sow farm | Shuozhou Xinhao | Sow farm |
| Raoyang Xinhao | Sow farm | Gaotang Xinhao | Sow farm | Raoyang Xinhao | Sow farm |
| Wulian Xinhao | Sow farm | Wuhe Xinhai | Sow farm | Xiajin Agriculture and Animal Husbandry | Sow farm |
| Yucheng Xinliu | Sow farm | Raoyang Xinhao | Sow farm | Xiajin Agriculture and Animal Husbandry | Sow farm |
| Wuhe Xinhai | Sow farm | Wulian Xinhao | Sow farm | Heishan Gaojia | Sow farm |
| Nanning Xinliu | Sow farm | Wuhe Xinhai | Sow farm | Meishan Xinhai | Sow farm |
| Changyi Xinhao | Sow farm | Laibin Xinhao | Sow farm | Changyi Xinhao | Sow farm |
| Liaoning New Hope | Sow farm | Liaoning Xinwang | Sow farm | Fuxin Xinwang | Sow farm |
| Tongliao Xinhao | Sow farm | Shuozhou Xinhao | Sow farm | Zhangxin Prospect Field | Sow farm |
| Gaotang Xinhao | Sow farm | Tongliao Xinhao | Sow farm | Rugao Xinhao | Sow farm |
| Shuozhou Xinhao | Sow farm | Tongliao Xinhao | Sow farm | Raoyang Xinhao | Sow farm |
| Tongliao Xinhao | Sow farm | Shuozhou Xinhao | Sow farm | Zhangxin Prospect Field | Sow farm |
| Wuhe Xinhai | Sow farm | Dong'e Xinliu | Sow farm | Meishan Xinhai | Sow farm |
| Liaoning New Hope | Sow farm | Liaoning Xinwang | Sow farm | Changyi Xinhao | Sow farm |
| Xiajin Agriculture and Animal Husbandry | Sow farm | Liaoning Xinwang | Sow farm | Shuozhou Xinhao | Sow farm |
| Liaoning New Hope | Sow farm | Liaocheng Xinhao | Sow farm | Shuozhou Xinhao | Sow farm |
| Tongliao Xinhao | Sow farm | Xiajin Nongmu | Sow farm | Laibin Xinhao | Sow farm |
| Liaoning New Hope | Sow farm | Anhui Xinliu | Sow farm | Wulian Xinhao | Sow farm |
| Shuozhou Xinhao | Sow farm | Dong'e Xinliu | Sow farm | Heishan Gaojia | Sow farm |
| Dong'e Xinliu | Sow farm | Liaocheng Xinhao | Sow farm | Wulian Xinhao | Sow farm |
| Tongliao Xinhao | Sow farm | Zhangxinwangchang | Sow farm | Wuhe Xinhai | Sow farm |
| Yucheng Xinliu | Sow farm | Liaoning Xinwang | Sow farm | Wuhe Xinhai | Sow farm |
| Zhangxin Wangchang | Sow farm | Tongliao Xinhao | Sow farm | Heishan Gaojia | Sow farm |
| Shuozhou Xinhao | Sow farm | Gaotang Xinhao | Sow farm | Liaocheng Xinhao | Sow farm |
| Shuozhou Xinhao | Sow farm | Laibin Xinhao | Sow farm | Gaotang Xinhao | Sow farm |
| Heishan Gaojia | Sow farm | Heishan Gaojia | Sow farm | Liaocheng Xinhao | Sow farm |
| Liaocheng Xinhao | Sow farm | Tongliao Xinhao | Sow farm | Anhui Xinliu | Sow farm |
| Anhui Xinliu | Sow farm | Meishan Xinhai | Sow farm | Xiajin Agriculture and Animal Husbandry | Sow farm |
| Xiajin Agriculture and Animal Husbandry | Sow farm | Wuhe Xinhai | Sow farm | Wuhe Xinhai | Sow farm |
| Laibin Xinhao | Sow farm | Dong'e Xinliu | Sow farm | Laixi Xinliu | Sow farm |
| Wuhe Xinhai | Sow farm | Taian Xinliu | Sow farm | Liaoning Xinwang | Sow farm |
| Liaocheng Xinhao | Sow farm | Lingao Xinliu | Sow farm | Liaoning Xinwang | Sow farm |
| Wannian Xinhai | Sow farm | Nanning Xinliu | Sow farm | Liaoning Xinwang | Sow farm |
| Taian Xinliu | Sow farm | Xiajin Nongmu | Sow farm | Tongliao Xinhao | Sow farm |
| Laixi Xinliu | Sow farm | Yucheng Xinliu | Sow farm | Taian Xinliu | Sow farm |
| Xiajin Agriculture and Animal Husbandry | Sow farm | Xiajin Nongmu | Sow farm | Laibin Xinhao | Sow farm |
| Zhangjiakou New Hope | Sow farm | Taian Xinliu | Sow farm | Taian Xinliu | Sow farm |
| Wannian Xinhai | Sow farm | Liaoning Xintao Wang | Sow farm | Taian Xinliu | Sow farm |
| Guigang Xinliu | Sow farm | Kangping Xinwang | Sow farm | Zhangxin Prospect Field | Sow farm |
| Xiajin Agriculture and Animal Husbandry | Sow farm | Wannian Xinhai | Sow farm | Linqing Xinliu | Sow farm |
| Laibin Xinhao | Sow farm | Yucheng Xinliu | Sow farm | Liaoning Xinwang | Sow farm |
| Laibin Xinhao | Sow farm | Laibin Xinhao | Sow farm | Tongliao Xinhao | Sow farm |
| Liaoning Xintao Wang | Sow farm | Laibin Xinhao | Sow farm | Santai Agriculture and Animal Husbandry | Sow farm |
| Xiajin Agriculture and Animal Husbandry | Sow farm | Santai Nongmu | Sow farm | Liaoning New Tao Prospect | Sow farm |
| Wuhe Xinhai | Sow farm | Taian Xinliu | Sow farm | Tongliao Xinhao | Sow farm |
| Guanling Xinhai | Sow farm | Wuhe Xinhai | Sow farm | Gansu Xinhao | Sow farm |
| Wuhe Xinhai | Sow farm | Chenzhou Xinhao | Sow farm | Xiajin Agriculture and Animal Husbandry | Sow farm |
| Taian Xinliu | Sow farm | Laibin Xinhao | Sow farm | Yingcheng Xinhao | Sow farm |
| Jiangxi Xinliu | Sow farm | Hezhou Xinhao | Sow farm | Laibin Xinhao | Sow farm |
| Wuhe Xinhai | Sow farm | Hengnan Muyun | Sow farm | Chenzhou Xinhao | Sow farm |
| Langzhong Xinliu | Sow farm | Laixi Xinliu | Sow farm | Xiajin Agriculture and Animal Husbandry | Sow farm |
| Gansu Xinhao | Sow farm | Guangling Xinhai | Sow farm | Hubei Xinhao | Sow farm |
| Kangping New Hope | Sow farm | Xiajin Nongmu | Sow farm | Heishan Gaojia | Sow farm |
| Jiangxi Xinliu | Sow farm | Guangling Xinhai | Sow farm | Kangping Xinwang | Sow farm |
| Hengnan Muyun | Sow farm | Gansu Xinhao | Sow farm | Yingcheng Xinhao | Sow farm |
| Hubei Xinhao | Sow farm | Yingcheng Xinhao | Sow farm | Hezhou Xinhao | Sow farm |
| Santai Agriculture and Animal Husbandry | Sow farm | Meishan Xinliu | Sow farm | Hubei Xinhao | Sow farm |
| Hubei Xinhao | Sow farm | Wuhe Xinhai | Sow farm | Wuhe Xinhai | Sow farm |
| Guanling Xinhai | Sow farm | Gansu Xinhao | Sow farm | Hengnan Pastoral Farming | Sow farm |
| Wuqi Xinliu | Sow farm | Wuqi Xinliu | Sow farm | Laibin Xinhao | Sow farm |
| Linhai Xinliu | Sow farm | Liaocheng Xinhao | Sow farm | Guanling Xinhai | Sow farm |
| Laibin Xinhao | Sow farm | Hezhou Xinhao | Sow farm | Dong'e Xinliu | Sow farm |
| Zhangjiakou New Hope | Sow farm | Guigang Xinliu | Sow farm | Hubei Xinhao | Sow farm |
| Ningming Xinhao | Sow farm | Hubei Xinhao | Sow farm | Gansu Xinhao | Sow farm |
| Jiangxi Xinliu | Sow farm | Xiajin Nongmu | Sow farm | Tongliao Xinhao | Sow farm |
| Chenzhou Xinhao | Sow farm | Laixi Xinliu | Sow farm | Kangping Xinwang | Sow farm |
| Taian Xinliu | Sow farm | Yingcheng Xinhao | Sow farm | Hezhou Xinhao | Sow farm |
| Pingfu Agriculture and Animal Husbandry | Sow farm | Jiangxi Xinliu | Sow farm | Liaocheng Xinhao | Sow farm |
| Laixi Xinliu | Sow farm | Bijie Xinliu | Sow farm | Guanling Xinhai | Sow farm |
| Hezhou Xinhao | Sow farm | Jiangxi Xinliu | Sow farm | Hubei Xinhao | Sow farm |
| Weinan Xinliu | Sow farm | Hubei Xinhao | Sow farm | Xiajin Agriculture and Animal Husbandry | Sow farm |
| Wuqi Xinliu | Sow farm | Wuhe Xinhai | Sow farm | Yingcheng Xinhao | Sow farm |
| Langzhong Xinliu | Sow farm | Ningming Xinhao | Sow farm | Gansu Xinhao | Sow farm |
| Yanting Xinhao | Sow farm | Chenzhou Xinhao | Sow farm | Laibin Xinhao | Sow farm |
| Guangdong Xinhao | Sow farm | Yingcheng Xinhao | Sow farm | Gaotang Xinhao | Sow farm |
| Taian Xinliu | Sow farm | Hubei Xinhao | Sow farm | Hezhou Xinhao | Sow farm |
| Chenzhou Xinhao | Sow farm | Hubei Xinhao | Sow farm | Wuhe Xinhai | Sow farm |
| Hubei Xinhao | Sow farm | Hezhou Xinhao | Sow farm | Hubei Xinhao | Sow farm |
| Chenzhou Xinhao | Sow farm | Jiangxi Xinliu | Sow farm | Hubei Xinhao | Sow farm |
| Laibin Xinhao | Sow farm | Pingfu Nongmu | Sow farm | Laibin Xinhao | Sow farm |
| Mianyang Xinhai | Sow farm | Pingfu Nongmu | Sow farm | Wuhe Xinhai | Sow farm |
| Yanting Xinhao | Sow farm | Yanting Xinhao | Sow farm | Yingcheng Xinhao | Sow farm |
| Hezhou Xinhao | Sow farm | Wuqi Xinliu | Sow farm | Ningming Xinhao | Sow farm |
| Guangyuan Xinhao | Sow farm | Kangping Xinwang | Sow farm | Chenzhou Xinhao | Sow farm |
| Laibin Xinhao | Sow farm | Chenzhou Xinhao | Sow farm | Wuqi Xinliu | Sow farm |
| Yingcheng Xinhao | Sow farm | Guangan Xinhao | Sow farm | Taian Xinliu | Sow farm |
| Xiajin Agriculture and Animal Husbandry | Sow farm | Hezhou Xinhao | Sow farm | Chenzhou Xinhao | Sow farm |
| Hezhou Xinhao | Sow farm | Hengnan Muyun | Sow farm | Pingfu Agriculture and Animal Husbandry | Sow farm |
| Bijie Xinliu | Sow farm | Hubei Xinhao | Sow farm | Liaoning New Tao Prospect | Sow farm |
| Dong'e Xinliu | Sow farm | Zhangjiakou Xinwang | Sow farm | Laibin Xinhao | Sow farm |
| Yingcheng Xinhao | Sow farm | Liaoning Xintao Wang | Sow farm | Gansu Xinhao | Sow farm |
| Zhangjiakou New Hope | Sow farm | Guangyuan Xinhao | Sow farm | Hezhou Xinhao | Sow farm |
| Dong'e Xinliu | Sow farm | Yingcheng Xinhao | Sow farm | Shibing Xinliu | Sow farm |
| Hezhou Xinhao | Sow farm | Wannian Xinhai | Sow farm | Liaocheng Xinhao | Sow farm |
| Guangan Xinhao | Sow farm | Hubei Xinhao | Sow farm | Pingfu Agriculture and Animal Husbandry | Sow farm |
| Hubei Xinhao | Sow farm | Guangan Xinhao | Sow farm | Bijie Xinliu | Sow farm |
| Yan'an Benyuan | Sow farm | Gansu Xinhao | Sow farm | Guangyuan Xinhao | Sow farm |
| Guangan Xinhao | Sow farm | Guangdong Xinhao | Sow farm | Guangan Xinhao | Sow farm |
| Hubei Xinhao | Sow farm | Taian Xinliu | Sow farm | Hengnan Pastoral Farming | Sow farm |
| Yingcheng Xinhao | Sow farm | Shibing Xinliu | Sow farm | Gansu Xinhao | Sow farm |
| Yichun New Hope | Sow farm | Guigang Xinliu | Sow farm | Weinan Xinliu | Sow farm |
| Shibing Xinliu | Sow farm | Gansu Xinhao | Sow farm | Zhejiang Xinhai | Sow farm |
| Laibin Xinhao | Sow farm | Xiajin Nongmu | Sow farm | Laixi Xinliu | Sow farm |
| Liaocheng Xinhao | Sow farm | Hezhou Xinhao | Sow farm | Shibing Xinliu | Sow farm |
| Kangping New Hope | Sow farm | Weinan Xinliu | Sow farm | Wuqi Xinliu | Sow farm |
| Hezhou Xinhao | Sow farm | Gansu Xinhao | Sow farm | Dong'e Xinliu | Sow farm |
| Hubei Xinhao | Sow farm | Zhangjiakou Xinwang | Sow farm | Guigang Xinliu | Sow farm |
| Hubei Xinhao | Sow farm | Bijie Xinliu | Sow farm | Zhangjiakou Xinwang | Sow farm |
| Yingcheng Xinhao | Sow farm | Laibin Xinhao | Sow farm | Zhejiang Xinhai | Sow farm |
| Yanting Xinhao | Sow farm | Laiyang Xinhao | Sow farm | Langzhong Xinliu | Sow farm |
| Jiaxiang Xinliu | Sow farm | Jiangxi Xinliu | Sow farm | Guangan Xinhao | Sow farm |
| Gansu Xinhao | Sow farm | Langzhong Xinliu | Sow farm | Gansu Xinhao | Sow farm |
| Hubei Xinhao | Sow farm | Gansu Xinhao | Sow farm | Gansu Xinhao | Sow farm |
| Bijie Xinliu | Sow farm | Hengnan Muyun | Sow farm | Xiajin Agriculture and Animal Husbandry | Sow farm |
| Hubei Xinhao | Sow farm | Qinghua Nongmu | Sow farm | Hezhou Xinhao | Sow farm |
| Leshan Agriculture and Animal Husbandry | Sow farm | Liaoning Xintao Wang | Sow farm | Weinan Xinliu | Sow farm |
| Mianyang Xinhai | Sow farm | Laibin Xinhao | Sow farm | Qinghua Agriculture and Animal Husbandry | Sow farm |
| Jiangxi Xinliu | Sow farm | Jiaxiang Xinliu | Sow farm | Hubei Xinhao | Sow farm |
| Dong'e Xinliu | Sow farm | Shibing Xinliu | Sow farm | Hubei Xinhao | Sow farm |
| Kangping New Hope | Sow farm | Leshan Nongmu | Sow farm | Laibin Xinhao | Sow farm |
| Pingfu Agriculture and Animal Husbandry | Sow farm | Kangping Xinwang | Sow farm | Guangdong Xinhao | Sow farm |
| Guigang Xinliu | Sow farm | Liaocheng Xinhao | Sow farm | Hubei Xinhao | Sow farm |
| Ningming Xinhao | Sow farm | Yijun Xinliu | Sow farm | Leshan Agriculture and Animal Husbandry | Sow farm |
| Gansu Xinhao | Sow farm | Lingbao Xinliu | Sow farm | Jiangxi Xinliu | Sow farm |
| Gansu Xinhao | Sow farm | Kangping Xinwang | Sow farm | Lingbao Xinliu | Sow farm |
| Kangping New Hope | Sow farm | Jiaxiang Xin Liu | Sow farm | Laiyang Xinhao | Sow farm |
| Jiaxiang Xinliu | Sow farm | Gansu Xin Hao | Sow farm | Dong'e Xinliu | Sow farm |
| Leshan Agriculture and Animal Husbandry | Sow farm | Laiyang Xin Hao | Sow farm | Jiangxi Xinliu | Sow farm |
| Pingfu Agriculture and Animal Husbandry | Sow farm | Yan'an Benyuan | Sow farm | Bijie Xinliu | Sow farm |
| Hengnan Muyun | Sow farm | Yanting Xin Hao | Sow farm | Wuhe Xinhai | Sow farm |
| Lingbao Xinliu | Sow farm | Lingbao Xin Liu | Sow farm | Liaoning Xintao Wang | Sow farm |
| Yijun Xinliu | Sow farm | Guigang Xin Liu | Sow farm | Nanning Xinliu | Sow farm |
| Xiangzhou Xinhao | Sow farm | Zhangjiakou Xinwang | Sow farm | Yanting Xinhao | Sow farm |
| Shibing Xinliu | Sow farm | Hubei Xin Hao | Sow farm | Jiangxi Xinliu | Sow farm |
| Weinan Xinliu | Sow farm | Ningming Xin Hao | Sow farm | Lingbao Xinliu | Sow farm |
| Shibing Xinliu | Sow farm | Dong'a Xin Liu | Sow farm | Shibing Xinliu | Sow farm |
| Wuhe Xinhai | Sow farm | Guangyuan Xin Hao | Sow farm | Ningming Xinhao | Sow farm |
| Liaoning New Taowang | Sow farm | Hubei Xin Hao | Sow farm | Langzhou Xinliu | Sow farm |
| Guigang Xinliu | Sow farm | Weinan Xin Liu | Sow farm | Xiangzhou Xinhao | Sow farm |
| Caoxian Xinhao | Sow farm | Xiangzhou Xin Hao | Sow farm | Ningming Xinhao | Sow farm |
| Pucheng Xinliu | Sow farm | Gansu Xin Hao | Sow farm | Yijun Xinliu | Sow farm |
| Gansu Xinhao | Sow farm | Hubei Xin Hao | Sow farm | Kangping Xinwang | Sow farm |
| Yanting Xinhao | Sow farm | Caoxian Xin Hao | Sow farm | Hubei Xinhao | Sow farm |
| Hengnan Muyun | Sow farm | Hubei Xin Hao | Sow farm | Laiyang Xinhao | Sow farm |
| Guangyuan Xinhao | Sow farm | Langxin Xin Liu | Sow farm | Leshan Nongmu | Sow farm |
| Laibin Xinhao | Sow farm | Shibing Xin Liu | Sow farm | Yingcheng Xinhao | Sow farm |
| Pucheng Xinliu | Sow farm | Ningming Xin Hao | Sow farm | Kangping Xinwang | Sow farm |
| Ningming Xinhao | Sow farm | Yijun Xin Liu | Sow farm | Hubei Xinhao | Sow farm |
| Yingcheng Xinhao | Sow farm | Leshan Nongmu | Sow farm | Yucheng Xinliu | Sow farm |
| Shibing Xinliu | Sow farm | Gansu Xin Hao | Sow farm | Yanting Xinhao | Sow farm |
| Pucheng Xinliu | Sow farm | Wuhe Xinhai | Sow farm | Zhangjiakou Xinwang | Sow farm |
| Guangdong Xinhao | Sow farm | Laibin Xin Hao | Sow farm | Hainan Nongken | Sow farm |
| Liaoning New Taowang | Sow farm | Guigang Xin Liu | Sow farm | Pucheng Xinliu | Sow farm |
| Laiyang Xinhao | Sow farm | Dong'a Xin Liu | Sow farm | Guigang Xinliu | Sow farm |
| Nanning Xinhao | Sow farm | Yingcheng Xin Hao | Sow farm | Caoxian Xinhao | Sow farm |
| Weinan Xinliu | Sow farm | Yingcheng Xin Hao | Sow farm | Shibing Xinliu | Sow farm |
| Pucheng Xinliu | Sow farm | Ningming Xin Hao | Sow farm | Yingcheng Xinhao | Sow farm |
| Caoxian Xinhao | Sow farm | Yijun Xin Liu | Sow farm | Ningming Xinhao | Sow farm |
| Liaoning New Taowang | Sow farm | Shibing Xin Liu | Sow farm | Ningming Xinhao | Sow farm |
| Shibing Xinliu | Sow farm | Dong'a Xin Liu | Sow farm | Gansu Xinhao | Sow farm |
| Guangdong Xinhao | Sow farm | Hainan Nongken | Sow farm | Hengnan Muyun | Sow farm |
| Ningming Xinhao | Sow farm | Qinghua Nongmu | Sow farm | Caoxian Xinhao | Sow farm |
| Yijun Xinliu | Sow farm | Yingcheng Xin Hao | Sow farm | Xiangzhou Xinhao | Sow farm |
| Guigang Xinliu | Sow farm | Nanning Xin Hao | Sow farm | Guangyuan Xinhao | Sow farm |
| Hubei Xinhao | Sow farm | Pingfu Nongmu | Sow farm | Gansu Xinhao | Sow farm |
| Gansu Xinhao | Sow farm | Hainan Nongken | Sow farm | Yijun Xinliu | Sow farm |
| Qinghua Agriculture and Animal Husbandry | Sow farm | Guangdong Xin Hao | Sow farm | Ningming Xinhao | Sow farm |
| Zhejiang Xinhai | Sow farm | Zhejiang Xinhai | Sow farm | Yingcheng Xinhao | Sow farm |
| Ningming Xinhao | Sow farm | Pucheng Xin Liu | Sow farm | Guigang Xinliu | Sow farm |
| Laiyang Xinhao | Sow farm | Zhangxin Wangchang | Sow farm | Pingfu Nongmu | Sow farm |
| Yijun Xinliu | Sow farm | Ningming Xin Hao | Sow farm | Liaoning Xintao Wang | Sow farm |
| Pingfu Agriculture and Animal Husbandry | Sow farm | Pingfu Nongmu | Sow farm | Ningming Xinhao | Sow farm |
| Zhejiang Xinhai | Sow farm | Liaoning Xintao Wang | Sow farm | Jiaxiang Xinliu | Sow farm |
| Xiangzhou Xinhao | Sow farm | Ningming Xin Hao | Sow farm | Dong'e Xinliu | Sow farm |
| Yingcheng Xinhao | Sow farm | Xiangzhou Xin Hao | Sow farm | Yijun Xinliu | Sow farm |
| Ningming Xinhao | Sow farm | Pucheng Xin Liu | Sow farm | Zhangxin Wangchang | Sow farm |
| Nanning Xinhao | Sow farm | Caoxian Xin Hao | Sow farm | Hainan Nongken | Sow farm |
| Yichun Xinwang | Sow farm | Ningming Xin Hao | Sow farm | Xiangzhou Xinhao | Sow farm |
| Gansu Xinhao | Sow farm | Gansu Xin Hao | Sow farm | Guangdong Xinhao | Sow farm |
| Guangdong Xinhao | Sow farm | Zhejiang Xinhai | Sow farm | Wuhe Xinhai | Sow farm |
| Gansu Xinliu | Sow farm | Nanning Xin Hao | Sow farm | Dong'e Xinliu | Sow farm |
| Gansu Xinhao | Sow farm | Shibing Xin Liu | Sow farm | Ningbo Xinhai | Sow farm |
| Lingbao Xinliu | Sow farm | Yan'an Benyuan | Sow farm | Nanning Xinhao | Sow farm |
| Weinan Xinliu | Sow farm | Yanting Xin Hao | Sow farm | Pucheng Xinliu | Sow farm |
| Pingfu Agriculture and Animal Husbandry | Sow farm | Guangdong Xin Hao | Sow farm | Yucheng Xinliu | Sow farm |
| Guangdong Xinhao | Sow farm | Caoxian Xin Hao | Sow farm | Shibing Xinliu | Sow farm |
| Xiangzhou Xinhao | Sow farm | Xiangzhou Xin Hao | Sow farm | Pingfu Nongmu | Sow farm |
| Ningming Xinhao | Sow farm | Weinan Xin Liu | Sow farm | Guigang Xinliu | Sow farm |
| Guangyuan Xinhao | Sow farm | Guangdong Xin Hao | Sow farm | Gansu Xinhao | Sow farm |
| Gansu Xinhao | Sow farm | Gansu Xin Liu | Sow farm | Pingfu Nongmu | Sow farm |
| Nanning Xinhao | Sow farm | Yichun Xinwang | Sow farm | Pucheng Xinliu | Sow farm |
| Yingcheng Xinhao | Sow farm | Ningbo Xinhai | Sow farm | Caoxian Xinhao | Sow farm |
| Caoxian Xinhao | Sow farm | Pingfu Nongmu | Sow farm | Qinghua Nongmu | Sow farm |
| Pingfu Agriculture and Animal Husbandry | Sow farm | Pucheng Xin Liu | Sow farm | Yan'an Benyuan | Sow farm |
| Guigang Xinliu | Sow farm | Pingfu Nongmu | Sow farm | Guangdong Xinhao | Sow farm |
| Qinghua Agriculture and Animal Husbandry | Sow farm | Gansu Xin Hao | Sow farm | Wannian Xinhai | Sow farm |
| Zhangxinwangchang | Sow farm | Nanning Xin Hao | Sow farm | Nanning Xinhao | Sow farm |
| Liaocheng Xinhao | Sow farm | Guangyuan Xin Hao | Sow farm | Nanning Xinhao | Sow farm |
| Hainan State Farms | Sow farm | Yanting Xin Hao | Sow farm | Jiangxi Xinliu | Sow farm |
| Yan'an Benyuan | Sow farm | Yichun Xinwang | Sow farm | Nanning Xinhao | Sow farm |
| Gansu Xinhao | Sow farm | Nanning Xin Hao | Sow farm | Gansu Xinhao | Sow farm |
| Gansu Xinhao | Sow farm | Guigang Xin Liu | Sow farm | Yanting Xinhao | Sow farm |
| Nanning Xinhao | Sow farm | Nanning Xin Liu | Sow farm | Guangdong Xinhao | Sow farm |
| Yingtan Xinliu | Sow farm | Linyi Zhongzhu | Sow farm | Guangyuan Xinhao | Sow farm |
| Liaoning New Taowang | Sow farm | Weinan Xin Liu | Sow farm | Yan'an Benyuan | Sow farm |
| Yan'an Benyuan | Sow farm | Caoxian Xin Hao | Sow farm | Liaoning Xintao Wang | Sow farm |
| Ruyuan Xinhao | Sow farm | Guangdong Xin Hao | Sow farm | Zhangjiakou Xinwang | Sow farm |
| Nanning Xinliu | Sow farm | Ruyuan Xin Hao | Sow farm | Pucheng Xinliu | Sow farm |
| Zhaoqing Xinhao | Sow farm | Liaoning Xintao Wang | Sow farm | Weinan Xinliu | Sow farm |
| Heyang Xinliu | Sow farm | Pucheng Xin Liu | Sow farm | Pingfu Nongmu | Sow farm |
| Caoxian Xinhao | Sow farm | Ruyuan Xin Hao | Sow farm | Nanning Xinliu | Sow farm |
| Ruyuan Xinhao | Sow farm | Lingao Xin Liu | Sow farm | Dong'e Xinliu | Sow farm |
| Zhaoqing Xinhao | Sow farm | Yingtan Xin Liu | Sow farm | Gansu Xinliu | Sow farm |
| Gansu Xinliu | Sow farm | Xiangzhou Xin Hao | Sow farm | Yichun Xinwang | Sow farm |
| Xiangzhou Xinhao | Sow farm | Yan'an Benyuan | Sow farm | Lingao Xinliu | Sow farm |
| Yingtan Xinliu | Sow farm | Hubei Xin Hao | Sow farm | Weinan Xinliu | Sow farm |
| Hainan State Farms | Sow farm | Ningbo Xinhai | Sow farm | Caoxian Xinhao | Sow farm |
| Ningbo Xinhai | Sow farm | Gansu Xin Liu | Sow farm | Jiaxiang Xinliu | Sow farm |
| Ningbo Xinhai | Sow farm | Heyang Xin Liu | Sow farm | Xiangzhou Xinhao | Sow farm |
| Xiangzhou Xinhao | Sow farm | Zhaoqing Xin Hao | Sow farm | Yan'an Benyuan | Sow farm |
| Hainan Xinliu | Sow farm | Xiangzhou Xin Hao | Sow farm | Wannian Xinhai | Sow farm |
| Zhaoqing Xinhao | Sow farm | Zhaoqing Xin Hao | Sow farm | Ningbo Xinhai | Sow farm |
| Heyang Xinliu | Sow farm | Zhaoqing Xin Hao | Sow farm | Yingtan Xinliu | Sow farm |
| Hainan Xinliu | Sow farm | Xiajiang Xin Liu | Sow farm | Yichun Xinwang | Sow farm |
| Zhaoqing Xinhao | Sow farm | Yingtan Xin Liu | Sow farm | Yanting Xinhao | Sow farm |
| Lingao Xinliu | Sow farm | Hainan Xin Liu | Sow farm | Heyang Xinliu | Sow farm |
| Xiajiang Xinliu | Sow farm | Hainan Xin Liu | Sow farm | Guigang Xinliu | Sow farm |
| Liaoning New Taowang | Sow farm | Zhaoqing Xin Hao | Sow farm | Guangdong Xinhao | Sow farm |
| Hubei Xinhao | Sow farm | Heyang Xin Liu | Sow farm | Yingtan Xinliu | Sow farm |
| Laiyang Xinmu | Sow farm | Liaoning Xintao Wang | Sow farm | Ruyuan Xinhao | Sow farm |
|  |  | Laiyang Xinmu | Sow farm | Ruyuan Xinhao | Sow farm |
|  |  | Laiyang Xinmu | Sow farm | Xiangzhou Xinliu | Sow farm |
|  |  |  |  | Hainan Xinliu | Sow farm |
|  |  |  |  | Zhaoqing Xinhao | Sow farm |
|  |  |  |  | Zhaoqing Xinhao | Sow farm |
|  |  |  |  | Zhaoqing Xinhao | Sow farm |
|  |  |  |  | Hainan Xinliu | Sow farm |
|  |  |  |  | Gansu Xinliu | Sow farm |
|  |  |  |  | Heyang Xinliu | Sow farm |
| **Oct 2022** | | **Nov 2022** | | **Dec 2022** | |
| **Company (Co., Ltd.)** | **Type of pig farm** | **Company (Co., Ltd.)** | **Type of pig farm** | **Company (Co., Ltd.)** | **Type of pig farm** |
| Dongying Xinhao | Sow farm | Rongxian Xinhai | Sow farm | Lezhi Xinhai | Sow farm |
| Laibin Breeding | Sow farm | Meishan Xinhai | Sow farm | Beijing Xinliu | Sow farm |
| Beijing Xinliu | Sow farm | Meishan Xinha | Sow farm | Lezhi Xinhai | Sow farm |
| Shanxian Xinhao | Sow farm | Rongxian Xinha | Sow farm | Tongliao Xinhao | Sow farm |
| Zibo Breeding | Sow farm | Beijing Xinliu | Sow farm | Laiyang Xinhao | Sow farm |
| HeishanWoniu | Sow farm | Jiangxi Xinliu | Sow farm | Lezhi Xinhai | Sow farm |
| Laiyang Xinmu | Fattening farm | Hengnan Muyun | Sow farm | Rongxian Xinhai | Sow farm |
| Fuxin Xinwang | Fattening farm | Dongying Xinhao | Sow farm | Shuozhou Xinhao | Sow farm |
| Zhangxin Prospect Field | Sow farm | Liaoning Xintao Wang | Sow farm | Zibo Breeding | Sow farm |
| Heishan | Sow farm | Yancheng Zhongtai | Sow farm | Hengnan Muyun | Sow farm |
| Yanting Xinhao | Sow farm | Fuxin Xinwang | Fattening farm | Beijing Xinliu | Sow farm |
| Nanning Xinliu | Sow farm | Laibin Yuzhong | Sow farm | Laibin Breeding | Sow farm |
| Heishan | Sow farm | Chenzhou Xinhao | Sow farm | Hebei Xinhao | Sow farm |
| Anyang Xinliu | Sow farm | Laiyang Xinhao | Sow farm | Heishan Woniou | Sow farm |
| Weinan Xinliu | Sow farm | Yanting Xinhao | Sow farm | Shuozhou Xinhao | Sow farm |
| Shuozhou Xinhao | Sow farm | Zibo Yuzhong | Sow farm | Shuozhou Xinhao | Sow farm |
| Weinan Xinliu | Sow farm | Dongying Xinhao | Sow farm | Xiajiang Xinliu | Sow farm |
| Beijing Xinliu | Sow farm | Hebei Xinhao | Sow farm | Rongxian Xinhai | Sow farm |
| Liuzhou Xinliu | Sow farm | Heishan Woniou | Sow farm | Laiyang Xinhao | Sow farm |
| Meishan Xinhai | Sow farm | Yanting Xinhao | Sow farm | Shuozhou Xinhao | Sow farm |
| Tongliao Xinhao | Sow farm | Beijing Xinliu | Sow farm | Hebei Xinhao | Sow farm |
| Fuxin Xinwang | Sow farm | Laixi Xinliu | Sow farm | Rongxian Xinhai | Sow farm |
| Xiajiang Xinliu | Sow farm | Liaoning Xintao Wang | Sow farm | Yanting Xinhao | Sow farm |
| Lezhi Xinhai | Sow farm | Laixi Xinliu | Sow farm | Dongying Xinhao | Sow farm |
| Xingren Xinliu | Sow farm | Longhai Xinhai | Sow farm | Liaoning Xintao Wang | Sow farm |
| Xingren Xinliu | Sow farm | Xiajiang Xinliu | Sow farm | Zhenjiang Xinhai | Sow farm |
| Chenzhou Xinhao | Sow farm | Shuozhou Xinhao | Sow farm | Gansu Xinliu | Sow farm |
| Laibin Breeding | Sow farm | Shuozhou Xinhao | Sow farm | Liuzhou Xinliu | Sow farm |
| Langzhong Xinliu | Sow farm | Shanxian Xinhao | Sow farm | Dongying Xinhao | Sow farm |
| Zhenjiang Xinhai | Sow farm | Shuozhou Xinhao | Sow farm | Wuqi Xinliu | Sow farm |
| Guang'an Xinhao | Sow farm | Liuzhou Xinliu | Sow farm | Shuozhou Xinhao | Sow farm |
| Gansu Xinliu | Sow farm | Zhangxin Wangchang | Sow farm | Langzhong Xinliu | Sow farm |
| Guanling Xinhai | Sow farm | Hebei Xinhao | Sow farm | Guang'an Xinhao | Sow farm |
| Dongying Xinhao | Sow farm | Zhenjiang Xinhai | Sow farm | Guangling Xinhai | Sow farm |
| Wuqi Xinliu | Sow farm | Fuxin Xinwang | Sow farm | Danxian Xinhao | Sow farm |
| Chenzhou Xinhao | Sow farm | Gansu Xinliu | Sow farm | Dongying Xinhao | Sow farm |
| Laizhou Xinhai | Sow farm | Guang'an Xinhao | Sow farm | Lezhi Xinhai | Sow farm |
| Meishan Xinhai | Sow farm | Langzhong Xinliu | Sow farm | Laizhou Xinhai | Sow farm |
| Wannian Xinhai | Sow farm | Wuqi Xinliu | Sow farm | Laizhou Xinhai | Sow farm |
| Weinan Xinliu | Sow farm | Guanling Xinhai | Sow farm | Zhejiang Xinhai | Sow farm |
| Laizhou Xinhai | Sow farm | Dongying Xinhao | Sow farm | Hainan Nongken | Sow farm |
| Meishan Xinhai | Sow farm | Laizhou Xinhai | Sow farm | Yichun Xintai | Sow farm |
| Zhejiang Xinhai | Sow farm | Letong Xinhai | Sow farm | Pingfu Nongmu | Sow farm |
| Hainan Agriculture | Sow farm | Laizhou Xinhai | Sow farm | Hainan Xinliu | Sow farm |
| Pingfu Farming and Animal Husbandry | Sow farm | Hebei Xinhao | Sow farm | Ningbo Xinhai | Sow farm |
| Ningbo Xinhai | Sow farm | Heishan Woniou | Sow farm | Chenzhou Xinhao | Sow farm |
| Yichun Xinwang | Sow farm | Shuozhou Xinhao | Sow farm | Yingtan Xinliu | Sow farm |
| Hainan Xinliu | Sow farm | Zhejiang Xinhai | Sow farm | Wannian Xinhai | Sow farm |
| Laixi Xinliu | Sow farm | Hainan Nongken | Sow farm | Zhangxinwangchang | Sow farm |
| Yingtan Xinliu | Sow farm | Yanting Xinhao | Sow farm | Hebei Xinhao | Sow farm |
| Shuozhou Xinhao | Sow farm | Pingfu Nongmu | Sow farm | Shuozhou Xinhao | Sow farm |
| Liuzhou Xinliu | Sow farm | Ningbo Xinhai | Sow farm | Fuxin Xintai | Sow farm |
| Shuozhou Xinhao | Sow farm | Heishan Woniou | Sow farm | Shuozhou Xinhao | Sow farm |
| Dong'e Xinliu | Sow farm | Tongliao Xinhao | Sow farm | Yanting Xinhao | Sow farm |
| Liaocheng Xinhao | Sow farm | Yichun Xinwang | Sow farm | Jingxian Xinhao | Sow farm |
| Changyi Xinhao | Sow farm | Hainan Xinliu | Sow farm | Shuozhou Xinhao | Sow farm |
| Shuozhou Xinhao | Sow farm | Wannian Xinhai | Sow farm | Liaoning Xintao Wang | Sow farm |
| Juye Xinhao | Sow farm | Yingtan Xinliu | Sow farm | Yancheng Zhongtai | Sow farm |
| Xinjin Xinhao | Sow farm | Laibin Yuzhong | Sow farm | Rongchang Pig Farm | Sow farm |
| Jingxian Xinhao | Sow farm | Chenzhou Xinhao | Sow farm | Tongliao Xinhao | Sow farm |
| Chenzhou Xinhao | Sow farm | Jingxian Xinhao | Sow farm | Juye Xinhao | Sow farm |
| Rongchang Pig Farm | Sow farm | Shuozhou Xinhao | Sow farm | Hebei Xinhao | Sow farm |
| Raoyang Xinhao | Sow farm | Rongchang Zhuchang | Sow farm | Longhai Xinhai | Sow farm |
| Tianjin Agriculture and Animal Husbandry | Sow farm | Laixi Xinliu | Sow farm | Laibin Breeding | Sow farm |
| Liuzhou Xinliu | Sow farm | Weinan Xinliu | Sow farm | Chenzhou Xinhao | Sow farm |
| Chenzhou Xinhao | Sow farm | Juye Xinhao | Sow farm | Juye Xinhao | Sow farm |
| Laizhou Xinhai | Sow farm | Xingren Xinliu | Sow farm | Raoyang Xinhao | Sow farm |
| Juye Xinhao | Sow farm | Xingren Xinliu | Sow farm | Raoyang Xinhao | Sow farm |
| Yinbao Breeding | Sow farm | Raoyang Xinhao | Sow farm | Hebei Xinhao | Sow farm |
| Laizhou Xinhai | Sow farm | Weinan Xinliu | Sow farm | Yinbao Breeding | Sow farm |
| Shuozhou Xinhao | Sow farm | Juye Xinhao | Sow farm | Heishan Woniou | Sow farm |
| Laizhou Xinhai | Sow farm | Changyi Xinhao | Sow farm | Raoyang Xinhao | Sow farm |
| Jingxian Xinhao | Sow farm | Chenzhou Xinhao | Sow farm | Yanting Xinhao | Sow farm |
| Dongying Xinhao | Sow farm | Weinan Xinliu | Sow farm | Tianjin Nongmu | Sow farm |
| Laizhou Xinhai | Sow farm | Shuozhou Xinhao | Sow farm | Hubei Xinhao | Sow farm |
| Laizhou Xinhai | Sow farm | Dong'e Xinliu | Sow farm | Yantai Xinhao | Sow farm |
| Weinan Xinliu | Sow farm | Yinbao Yuzheng | Sow farm | Weinan Xinliu | Sow farm |
| Weinan Xinliu | Sow farm | Tianjin Nongmu | Sow farm | Jingxian Xinhao | Sow farm |
| Heishan Gaojia | Sow farm | Laizhou Xinhai | Sow farm | Laizhou Xinhai | Sow farm |
| Laibin Breeding | Sow farm | Hubei Xinhao | Sow farm | Laizhou Xinhai | Sow farm |
| Dongying Xinhao | Sow farm | Laizhou Xinhai | Sow farm | Yantai Xinhao | Sow farm |
| Ruyuan Xinhao | Sow farm | Weinan Xinliu | Sow farm | Laizhou Xinhai | Sow farm |
| Laizhou Xinhai | Sow farm | Laizhou Xinhai | Sow farm | Heishan Woniou | Sow farm |
| Yantai Xinhao | Sow farm | Xinjin Xinhao | Sow farm | Heishan Gaojia | Sow farm |
| Taipeng Xinliu | Sow farm | Laibin Yuzhong | Sow farm | Lingbao Xinliu | Sow farm |
| Laizhou Xinhai | Sow farm | Dongying Xinhao | Sow farm | Zhenjiang Xinhao | Sow farm |
| Zhangjiakou Xinwang | Sow farm | Heishan Gaojia | Sow farm | Zhenjiang Xinhao | Sow farm |
| Santai Agriculture and Animal Husbandry | Sow farm | Laizhou Xinhai | Sow farm | Danxian Xinhao | Sow farm |
| Hubei Xinhao | Sow farm | Shuozhou Xinhao | Sow farm | Laizhou Xinhai | Sow farm |
| Tianjin Agriculture and Animal Husbandry | Sow farm | Yantai Xinhao | Sow farm | Leshan Nongmu | Sow farm |
| Laizhou Xinhai | Sow farm | Laizhou Xinhai | Sow farm | Weinan Xinliu | Sow farm |
| Yan'an Original Source | Sow farm | Yantai Xinhao | Sow farm | Taian Xinliu | Sow farm |
| Leshan Agriculture and Animal Husbandry | Sow farm | Yantai Xinhao | Sow farm | Danxian Xinhao | Sow farm |
| Tongliao Xinhao | Sow farm | Liuzhou Xinliu | Sow farm | Zhenjiang Xinhao | Sow farm |
| Lingbao Xinliu | Sow farm | Taian Xinliu | Sow farm | Yantai Xinhao | Sow farm |
| Yantai Xinhao | Sow farm | Jingxian Xinhao | Sow farm | Santai Nongmu | Sow farm |
| Yantai Xinhao | Sow farm | Laizhou Xinhai | Sow farm | Zhenjiang Xinhao | Sow farm |
| Kangping Xinwang | Sow farm | Tongliao Xinhao | Sow farm | Danxian Xinhao | Sow farm |
| Zhenjiang Xinhai | Sow farm | Leshan Nongmu | Sow farm | Danxian Xinhao | Sow farm |
| Tianjin Agriculture and Animal Husbandry | Sow farm | Lingbao Xinliu | Sow farm | Laizhou Xinhai | Sow farm |
| Zhenjiang Xinhai | Sow farm | Santai Nongmu | Sow farm | Guangdong Xinhao | Sow farm |
| Qinghua Agriculture and Animal Husbandry | Sow farm | Shuozhou Xinhao | Sow farm | Shibing Xinliu | Sow farm |
| Guigang Xinliu | Sow farm | Zhenjiang Xinhai | Sow farm | Chenzhou Xinhao | Sow farm |
| Zhenjiang Xinhai | Sow farm | Zhenjiang Xinhai | Sow farm | Laizhou Xinhai | Sow farm |
| Yantai Xinhao | Sow farm | Laizhou Xinhai | Sow farm | Yan'an Benyuan | Sow farm |
| Hezhou Xinhao | Sow farm | Zhenjiang Xinhai | Sow farm | Laizhou Xinhai | Sow farm |
| Guangdong Xinhao | Sow farm | Shanxian Xinhao | Sow farm | Kangping Xintai | Sow farm |
| Dongying Xinhao | Sow farm | Shibing Xinliu | Sow farm | Santai Nongmu | Sow farm |
| Shibing Xinliu | Sow farm | Zhangjiakou Xinwang | Sow farm | Liaocheng Xinhao | Sow farm |
| Longzhou Xinhao | Sow farm | Zhenjiang Xinhai | Sow farm | Yantai Xinhao | Sow farm |
| Pucheng Xinliu | Sow farm | Shanxian Xinhao | Sow farm | Gansu Xin Hao | Sow farm |
| Chenzhou Xinhao | Sow farm | Dongying Xinhao | Sow farm | Zhangjiakou Xintai | Sow farm |
| Dongying Xinhao | Sow farm | Guigang Xinliu | Sow farm | Longzhou Xinhao | Sow farm |
| Wuhe Xinhai | Sow farm | Chenzhou Xinhao | Sow farm | Guangyuan Xinhao | Sow farm |
| Tianjin Agriculture and Animal Husbandry | Sow farm | Longzhou Xinhao | Sow farm | Qinghua Nongmu | Sow farm |
| Shanxian Xinhao | Sow farm | Yan'an Benyuan | Sow farm | Laizhou Xinhai | Sow farm |
| Shanxian Xinhao | Sow farm | Shanxian Xinhao | Sow farm | Yantai Xinhao | Sow farm |
| Jiangxi Xinliu | Sow farm | Shanxian Xinhao | Sow farm | Hezhou Xinhao | Sow farm |
| Shanxian Xinhao | Sow farm | Laizhou Xinhai | Sow farm | Dongying Xinhao | Sow farm |
| Guangyuan Xinhao | Sow farm | Yantai Xinhao | Sow farm | Laibin Breeding | Sow farm |
| Nanning Xinliu | Sow farm | Qinghua Nongmu | Sow farm | Pucheng Xinliu | Sow farm |
| Jiaxiang Xinliu | Sow farm | Hezhou Xinhao | Sow farm | Nanning Xinliu | Sow farm |
| Liangshan Xinliu | Sow farm | Santai Nongmu | Sow farm | Tongliao Xinhao | Sow farm |
| Zhenjiang Xinhai | Sow farm | Pucheng Xinliu | Sow farm | Guigang Xinliu | Sow farm |
| Longzhou Xinhao | Sow farm | Guangdong Xinhao | Sow farm | Longzhou Xinhao | Sow farm |
| Gansu Xinhao | Sow farm | Guangyuan Xinhao | Sow farm | Pingfu Nongmu | Sow farm |
| Yantai Xinhao | Sow farm | Kangping Xinwang | Sow farm | Yantai Xinhao | Sow farm |
| Yantai Xinhao | Sow farm | Gansu Xinhao | Sow farm | Longzhou Xinhao | Sow farm |
| Pingfu Farming and Animal Husbandry | Sow farm | Yantai Xinhao | Sow farm | Nanning Xinhao | Sow farm |
| Longzhou Xinhao | Sow farm | Ruyuan Xinhao | Sow farm | Longzhou Xinhao | Sow farm |
| Longzhou Xinhao | Sow farm | Wuhe Xinhai | Sow farm | Longzhou Xinhao | Sow farm |
| Longzhou Xinhao | Sow farm | Jiaxiang Xinliu | Sow farm | Chenzhou Xinhao | Sow farm |
| Shanxian Xinhao | Sow farm | Longzhou Xinhao | Sow farm | Caoxian Xin Hao | Sow farm |
| Yantai Xinhao | Sow farm | Dongying Xinhao | Sow farm | Yantai Xinhao | Sow farm |
| Longzhou Xinhao | Sow farm | Jiangxi Xinliu | Sow farm | Jiangxi Xinliu | Sow farm |
| Longzhou Xinhao | Sow farm | Pingfu Nongmu | Sow farm | Jiaxiang Xinliu | Sow farm |
| Yantai Xinhao | Sow farm | Nanning Xinliu | Sow farm | Yantai Xinhao | Sow farm |
| Nanning Xinhao | Sow farm | Longzhou Xinhao | Sow farm | Dongying Xinhao | Sow farm |
| Wuhe Xinhai | Sow farm | Yantai Xinhao | Sow farm | Longzhou Xinhao | Sow farm |
| Yantai Xinhao | Sow farm | Longzhou Xinhao | Sow farm | Longzhou Xinhao | Sow farm |
| Santai Agriculture and Animal Husbandry | Sow farm | Yantai Xinhao | Sow farm | Yantai Xinhao | Sow farm |
| Shuozhou Xinhao | Sow farm | Longzhou Xinhao | Sow farm | Wuhe Xinhai | Sow farm |
| Caoxian Xinhao | Sow farm | Longzhou Xinhao | Sow farm | Dong'e Xinliu | Sow farm |
| Liaocheng Xinhao | Sow farm | Dongying Xinhao | Sow farm | Xinjin Xinhao | Sow farm |
| Gaotang Xinhao | Sow farm | Liaocheng Xinhao | Sow farm | Dongying Xinhao | Sow farm |
| Zhongshan Agriculture and Animal Husbandry | Sow farm | Yantai Xinhao | Sow farm | Wuhe Xinhai | Sow farm |
| Tianjin Agriculture and Animal Husbandry | Sow farm | Nanning Xinhao | Sow farm | Dongying Xinhao | Sow farm |
| Zhangjiakou Xinwang | Sow farm | Yantai Xinhao | Sow farm | Liangshan Xinliu | Sow farm |
| Fuxin Xinwang | Sow farm | Caoxian Xinhao | Sow farm | Ruyuan Xin Hao | Sow farm |
| Jiangyou Pig Farm | Sow farm | Longzhou Xinhao | Sow farm | Xingren Xinliu | Sow farm |
| Laibin Xinhao | Sow farm | Wuhe Xinhai | Sow farm | Xingren Xinliu | Sow farm |
| Shuozhou Xinhao | Sow farm | Liangshan Xinliu | Sow farm | Zhongshan Nongmu | Sow farm |
| Shuozhou Xinhao | Sow farm | Chenzhou Xinhao | Sow farm | Weinan Xinliu | Sow farm |
| Xiajin Agriculture and Animal Husbandry | Sow farm | Gaotang Xinhao | Sow farm | Weinan Xinliu | Sow farm |
| Fuxin Xinwang | Sow farm | Tianjin Nongmu | Sow farm | Gaotang Xin Hao | Sow farm |
| Liaoning Xinwang | Sow farm | Zhongshan Agriculture and Animal Husbandry | Sow farm | Jiangyou Pig Farm | Sow farm |
| Lezhi Xinhai | Sow farm | Chenzhou Xinhao | Sow farm | Weinan Xin Liu | Sow farm |
| Jingxian Xinhao | Sow farm | Jiangyou Pig Farm | Sow farm | Changyi Xin Hao | Sow farm |
| Changyi Xinhao | Sow farm | Tianjin Agriculture and Animal Husbandry | Sow farm | Chenzhou Xin Hao | Sow farm |
| Lezhi Xinhai | Sow farm | Tianjin Agriculture and Animal Husbandry | Sow farm | Liuzhou Xin Liu | Sow farm |
| Heishan Gaojia | Sow farm | Raoyang Xinhao | Sow farm | Tianjin Nongmu | Sow farm |
| Laibin Xinhao | Sow farm | Liuzhou Xinliu | Sow farm | Hubei Xin Hao | Sow farm |
| Weinan Xinliu | Sow farm | Weinan Xinliu | Sow farm | Jingxian Xin Hao | Sow farm |
| Laixi Xin Liu | Sow farm | Weinan Xinliu | Sow farm | Tianjin Nongmu | Sow farm |
| Raoyang Xin Hao | Sow farm | Laibin Xinhao | Sow farm | Laibin Xin Hao | Sow farm |
| Zhangxin Wangchang | Sow farm | Zhangjiakou New Hope | Sow farm | Tianjin Nongmu | Sow farm |
| Changyi Xin Hao | Sow farm | Tianjin Agriculture and Animal Husbandry | Sow farm | Chenzhou Xin Hao | Sow farm |
| Xiajin Nongmu | Sow farm | Jingxian Xinhao | Sow farm | Tianjin Nongmu | Sow farm |
| Liaocheng Xin Hao | Sow farm | Raoyang Xinhao | Sow farm | Liaocheng Xin Hao | Sow farm |
| Fuxin Xinwang | Sow farm | Fuxin New Hope | Sow farm | Zhangxin Wangchang | Sow farm |
| Linhai Xin Liu | Sow farm | Liaocheng Xinhao | Sow farm | Taian Xin Liu | Sow farm |
| Anhui Xin Liu | Sow farm | Zhangxin New Field | Sow farm | Xiajin Nongmu | Sow farm |
| Liaocheng Xin Hao | Sow farm | Xiajin Agriculture and Animal Husbandry | Sow farm | Zhangjiakou Xinwang | Sow farm |
| Zhangxin Wangchang | Sow farm | Linhai Xinliu | Sow farm | Anhui Xin Liu | Sow farm |
| Raoyang Xin Hao | Sow farm | Changyi Xinhao | Sow farm | Weinan Xin Liu | Sow farm |
| Heishan Gaojia | Sow farm | Liaoning New Taowang | Sow farm | Yingcheng Xin Hao | Sow farm |
| Liaoning Xintao Wang | Sow farm | Liaocheng Xinhao | Sow farm | Zhangxin Wangchang | Sow farm |
| Wulian Xin Hao | Sow farm | Laibin Xinhao | Sow farm | Liaoning Xintao Wang | Sow farm |
| Xiajin Nongmu | Sow farm | Liaoning New Hope | Sow farm | Liaocheng Xin Hao | Sow farm |
| Xiajin Nongmu | Sow farm | Anhui Xinliu | Sow farm | Linhai Xin Liu | Sow farm |
| Wulian Xin Hao | Sow farm | Liaoning New Hope | Sow farm | Heishan Gaojia | Sow farm |
| Xiajin Nongmu | Sow farm | Heishan Gaojia | Sow farm | Xiajin Nongmu | Sow farm |
| Tongliao Xin Hao | Sow farm | Zhangxin New Field | Sow farm | Liaoning Xinwang | Sow farm |
| Tongliao Xin Hao | Sow farm | Heishan Gaojia | Sow farm | Liaocheng Xin Hao | Sow farm |
| Taian Xin Liu | Sow farm | Fuxin New Hope | Sow farm | Heishan Gaojia | Sow farm |
| Heishan Gaojia | Sow farm | Changyi Xinhao | Sow farm | Wulian Xin Hao | Sow farm |
| Liaoning Xinwang | Sow farm | Fuxin New Hope | Sow farm | Changyi Xin Hao | Sow farm |
| Taian Xin Liu | Sow farm | Tongliao Xinhao | Sow farm | Gansu Xin Hao | Sow farm |
| Gansu Xin Hao | Sow farm | Tongliao Xinhao | Sow farm | Xiajin Nongmu | Sow farm |
| Liaoning Xinwang | Sow farm | Tongliao Xinhao | Sow farm | Gansu Xin Hao | Sow farm |
| Laibin Xin Hao | Sow farm | Xiajin Agriculture and Animal Husbandry | Sow farm | Liaoning Xintao Wang | Sow farm |
| Tongliao Xin Hao | Sow farm | Zhejiang Xinhai | Sow farm | Gansu Xin Hao | Sow farm |
| Tongliao Xin Hao | Sow farm | Gansu Xinhao | Sow farm | Hubei Xin Hao | Sow farm |
| Santai Nongmu | Sow farm | Liaoning New Taowang | Sow farm | Hubei Xin Hao | Sow farm |
| Wuhe Xinhai | Sow farm | Taian Xinliu | Sow farm | Santai Nongmu | Sow farm |
| Hubei Xin Hao | Sow farm | Hubei Xinhao | Sow farm | Tongliao Xin Hao | Sow farm |
| Zhejiang Xinhai | Sow farm | Liaocheng Xinhao | Sow farm | Guanling Xinhai | Sow farm |
| Liaoning Xinwang | Sow farm | Guanling Xinhai | Sow farm | Gansu Xin Hao | Sow farm |
| Hubei Xin Hao | Sow farm | Santai Agriculture and Animal Husbandry | Sow farm | Taian Xin Liu | Sow farm |
| Chenzhou Xin Hao | Sow farm | Liaoning New Hope | Sow farm | Zhejiang Xinhai | Sow farm |
| Wuhe Xinhai | Sow farm | Taian Xinliu | Sow farm | Xiajin Nongmu | Sow farm |
| Liaoning Xinwang | Sow farm | Xiajin Agriculture and Animal Husbandry | Sow farm | Hubei Xin Hao | Sow farm |
| Yingcheng Xin Hao | Sow farm | Wulian Xinhao | Sow farm | Guanling Xinhai | Sow farm |
| Guangling Xinhai | Sow farm | Gansu Xinhao | Sow farm | Gansu Xin Hao | Sow farm |
| Hengnan Muyun | Sow farm | Dong'e Xinliu | Sow farm | Liaoning Xinwang | Sow farm |
| Taian Xin Liu | Sow farm | Wulian Xinhao | Sow farm | Fuxin Xinwang | Sow farm |
| Taian Xin Liu | Sow farm | Tongliao Xinhao | Sow farm | Hengnan Muyun | Sow farm |
| Laixi Xin Liu | Sow farm | Gansu Xinhao | Sow farm | Hubei Xin Hao | Sow farm |
| Xiajin Nongmu | Sow farm | Hengnan Muyun | Sow farm | Taian Xin Liu | Sow farm |
| Gansu Xin Hao | Sow farm | Wuhuai Xinhai | Sow farm | Tongliao Xin Hao | Sow farm |
| Liaoning Xintao Wang | Sow farm | Hubei Xinhao | Sow farm | Tongliao Xin Hao | Sow farm |
| Hezhou Xin Hao | Sow farm | Taian Xinliu | Sow farm | Chenzhou Xin Hao | Sow farm |
| Heishan Gaojia | Sow farm | Hubei Xinhao | Sow farm | Wulian Xin Hao | Sow farm |
| Kangping Xinwang | Sow farm | Hubei Xinhao | Sow farm | Hezhou Xin Hao | Sow farm |
| Gansu Xin Hao | Sow farm | Hubei Xinhao | Sow farm | Dong'e Xin Liu | Sow farm |
| Zhejiang Xinhai | Sow farm | Guanling Xinhai | Sow farm | Liaoning Xinwang | Sow farm |
| Hubei Xin Hao | Sow farm | Hubei Xinhao | Sow farm | Xiajin Nongmu | Sow farm |
| Wuhe Xinhai | Sow farm | Laiyang Xinhao | Sow farm | Fuxin Xinwang | Sow farm |
| Laibin Xin Hao | Sow farm | Chenzhou Xinhao | Sow farm | Gansu Xin Hao | Sow farm |
| Laibin Xin Hao | Sow farm | Gansu Xinhao | Sow farm | Taian Xin Liu | Sow farm |
| Gaotang Xin Hao | Sow farm | Taian Xinliu | Sow farm | Hubei Xin Hao | Sow farm |
| Gansu Xin Hao | Sow farm | Hubei Xinhao | Sow farm | Xiajin Nongmu | Sow farm |
| Hubei Xin Hao | Sow farm | Wuhuai Xinhai | Sow farm | Hubei Xin Hao | Sow farm |
| Gansu Xin Hao | Sow farm | Yingcheng Xinhao | Sow farm | Laiyang Xin Hao | Sow farm |
| Guangling Xinhai | Sow farm | Xiajin Agriculture and Animal Husbandry | Sow farm | Wuhe Xinhai | Sow farm |
| Hubei Xin Hao | Sow farm | Gaotang Xinhao | Sow farm | Liaoning Xinwang | Sow farm |
| Hubei Xin Hao | Sow farm | Hubei Xinhao | Sow farm | Yingcheng Xin Hao | Sow farm |
| Xiajin Nongmu | Sow farm | Hezhou Xinhao | Sow farm | Dong'e Xin Liu | Sow farm |
| Hezhou Xin Hao | Sow farm | Liaoning New Hope | Sow farm | Hubei Xin Hao | Sow farm |
| Yingcheng Xin Hao | Sow farm | Gansu Xinhao | Sow farm | Fuxin Xinwang | Sow farm |
| Gansu Xin Hao | Sow farm | Hezhou Xinhao | Sow farm | Zhangxin Wangchang | Sow farm |
| Xiajin Nongmu | Sow farm | Weinan Xinliu | Sow farm | Guangdong Xin Hao | Sow farm |
| Wuhe Xinhai | Sow farm | Hubei Xinhao | Sow farm | Liaoning Xintao Wang | Sow farm |
| Hezhou Xin Hao | Sow farm | Guangdong Xinhao | Sow farm | Hengnan Muyun | Sow farm |
| Hezhou Xin Hao | Sow farm | Gansu Xinhao | Sow farm | Gaotang Xin Hao | Sow farm |
| Yingcheng Xin Hao | Sow farm | Hezhou Xinhao | Sow farm | Gansu Xin Hao | Sow farm |
| Liaocheng Xin Hao | Sow farm | Xiajin Agriculture and Animal Husbandry | Sow farm | Hubei Xin Hao | Sow farm |
| Wuhe Xinhai | Sow farm | Liaoning New Taowang | Sow farm | Gansu Xin Hao | Sow farm |
| Hubei Xin Hao | Sow farm | Kangping New Hope | Sow farm | Chenzhou Xin Hao | Sow farm |
| Laibin Xin Hao | Sow farm | Yingcheng Xinhao | Sow farm | Hubei Xin Hao | Sow farm |
| Wuqi Xin Liu | Sow farm | Yanting Xinhao | Sow farm | Kangping Xinwang | Sow farm |
| Pingfu Nongmu | Sow farm | Chenzhou Xinhao | Sow farm | Laibin Xin Hao | Sow farm |
| Liaoning Xintao Wang | Sow farm | Hezhou Xinhao | Sow farm | Ningbo Xinhai | Sow farm |
| Weinan Xin Liu | Sow farm | Gansu Xinhao | Sow farm | Wuhe Xinhai | Sow farm |
| Weinan Xin Liu | Sow farm | Dong'e Xinliu | Sow farm | Shibing Xin Liu | Sow farm |
| Chenzhou Xin Hao | Sow farm | Chenzhou Xinhao | Sow farm | Heishan Gaojia | Sow farm |
| Laiyang Xin Hao | Sow farm | Hubei Xinhao | Sow farm | Hubei Xin Hao | Sow farm |
| Laibin Xin Hao | Sow farm | Ningming Xinhao | Sow farm | Hezhou Xin Hao | Sow farm |
| Hubei Xin Hao | Sow farm | Hengnan Muyun | Sow farm | Changyi Xin Hao | Sow farm |
| Dong'e Xin Liu | Sow farm | Weinan Xinliu | Sow farm | Tongliao Xin Hao | Sow farm |
| Wuhe Xinhai | Sow farm | Xiajin Agriculture and Animal Husbandry | Sow farm | Chenzhou Xin Hao | Sow farm |
| Langzhong Xin Liu | Sow farm | Letian Xinhai | Sow farm | Yingcheng Xin Hao | Sow farm |
| Chenzhou Xin Hao | Sow farm | Linhai Xinliu | Sow farm | Yanting Xin Hao | Sow farm |
| Guangdong Xin Hao | Sow farm | Hubei Xinhao | Sow farm | Laibin Xin Hao | Sow farm |
| Hubei Xin Hao | Sow farm | Wuqi Xinliu | Sow farm | Hezhou Xin Hao | Sow farm |
| Yingcheng Xin Hao | Sow farm | Shibing Xinliu | Sow farm | Yingcheng Xin Hao | Sow farm |
| Gansu Xin Hao | Sow farm | Laibin Xinhao | Sow farm | Laiyang Xin Hao | Sow farm |
| Zhangjiakou Xinwang | Sow farm | Heishan Gaojia | Sow farm | Langzhong Xin Liu | Sow farm |
| Laibin Xin Hao | Sow farm | Guangyuan Xinhao | Sow farm | Laibin Xin Hao | Sow farm |
| Jiangxi Xin Liu | Sow farm | Shibing Xinliu | Sow farm | Hezhou Xin Hao | Sow farm |
| Guangan Xin Hao | Sow farm | Laibin Xinhao | Sow farm | Liaoning Xintao Wang | Sow farm |
| Hubei Xin Hao | Sow farm | Yingcheng Xinhao | Sow farm | Shibing Xin Liu | Sow farm |
| Leshan Nongmu | Sow farm | Gansu Xinhao | Sow farm | Ningming Xin Hao | Sow farm |
| Langzhong Xin Liu | Sow farm | Gansu Xinhao | Sow farm | Laibin Xin Hao | Sow farm |
| Kangping Xinwang | Sow farm | Leshan Agriculture and Animal Husbandry | Sow farm | Linhai Xin Liu | Sow farm |
| Jiangxi Xin Liu | Sow farm | Laibin Xinhao | Sow farm | Leshan Nongmu | Sow farm |
| Guangyuan Xin Hao | Sow farm | Leshan Agriculture and Animal Husbandry | Sow farm | Leshan Nongmu | Sow farm |
| Ningming Xin Hao | Sow farm | Hezhou Xinhao | Sow farm | Ningming Xin Hao | Sow farm |
| Hubei Xin Hao | Sow farm | Kangping New Hope | Sow farm | Hengnan Muyun | Sow farm |
| Shibing Xin Liu | Sow farm | Weinan Xinliu | Sow farm | Hezhou Xin Hao | Sow farm |
| Hengnan Muyun | Sow farm | Yingcheng Xinhao | Sow farm | Liaoning Xintao Wang | Sow farm |
| Leshan Nongmu | Sow farm | Ningbo Xinhai | Sow farm | Zhangxin Wangchang | Sow farm |
| Shibing Xin Liu | Sow farm | Yanting Xinhao | Sow farm | Gansu Xin Hao | Sow farm |
| Ningming Xin Hao | Sow farm | Wuhuai Xinhai | Sow farm | Kangping Xinwang | Sow farm |
| Pingfu Nongmu | Sow farm | Xiangzhou Xinhao | Sow farm | Shibing Xin Liu | Sow farm |
| Wuqi Xin Liu | Sow farm | Wuhuai Xinhai | Sow farm | Guigang Xin Liu | Sow farm |
| Ningming Xin Hao | Sow farm | Ningming Xinhao | Sow farm | Guang'an Xin Hao | Sow farm |
| Yingcheng Xin Hao | Sow farm | Shibing Xinliu | Sow farm | Laibin Xin Hao | Sow farm |
| Lingbao Xin Liu | Sow farm | Laibin Xinhao | Sow farm | Zhangjiakou Xinwang | Sow farm |
| Zhangjiakou Xinwang | Sow farm | Bijie Xinliu | Sow farm | Ningbo Xinhai | Sow farm |
| Hezhou Xin Hao | Sow farm | Liaoning New Hope | Sow farm | Weinan Xin Liu | Sow farm |
| Yanting Xinhao | Sow farm | Lanxin Xinliu | Sow farm | Yingcheng Xinhao | Sow farm |
| Zhangxin Wangchang | Sow farm | Lingbao Xinliu | Sow farm | Yanting Xinhao | Sow farm |
| Gansu Xinhao | Sow farm | Guangan Xinhao | Sow farm | Lingbao Xinliu | Sow farm |
| Gansu Xinhao | Sow farm | Zhejiang Xinhai | Sow farm | Weinan Xinliu | Sow farm |
| Lingbao Xinliu | Sow farm | Lanxin Xinliu | Sow farm | Yijun Xinliu | Sow farm |
| Bijie Xinliu | Sow farm | Qinghua Agriculture and Animal Husbandry | Sow farm | Liuzhou Xinliu | Sow farm |
| Bijie Xinliu | Sow farm | Liaoning New Taowang | Sow farm | Pingfu Agriculture and Animal Husbandry | Sow farm |
| Xiajin Agriculture and Animal Husbandry | Sow farm | Zhangxiang Wangchang | Sow farm | Xiajin Agriculture and Animal Husbandry | Sow farm |
| Dong'e Xinliu | Sow farm | Pingfu Agriculture and Animal Husbandry | Sow farm | Qinghua Agriculture and Animal Husbandry | Sow farm |
| Weinan Xinliu | Sow farm | Ningming Xinhao | Sow farm | Liaoning New Hope | Sow farm |
| Yijun Xinliu | Sow farm | Yijun Xinliu | Sow farm | Heishan Gaojia | Sow farm |
| Kangping Newwang | Sow farm | Zhangjiakou Xinwang | Sow farm | Wuqi Xinliu | Sow farm |
| Lingxin Xinliu | Sow farm | Jiangxi Xinliu | Sow farm | Yingcheng Xinhao | Sow farm |
| Wuhe Xinhai | Sow farm | Xiajin Agriculture and Animal Husbandry | Sow farm | Xiangzhou Xinhao | Sow farm |
| Qinghua Agriculture and Animal Husbandry | Sow farm | Wuhe Xinhai | Sow farm | Xiajin Agriculture and Animal Husbandry | Sow farm |
| Liaoning Xintao Wang | Sow farm | Bijie Xinliu | Sow farm | Yichun New Hope | Sow farm |
| Ningming Xinhao | Sow farm | Weinan Xinliu | Sow farm | Nanning Xinhao | Sow farm |
| Xiangzhou Xinhao | Sow farm | Liaoning New Taowang | Sow farm | Weinan Xinliu | Sow farm |
| Guigang Xinliu | Sow farm | Wuqi Xinliu | Sow farm | Wuqi Xinliu | Sow farm |
| Shibing Xinliu | Sow farm | Yingcheng Xinhao | Sow farm | Pingfu Agriculture and Animal Husbandry | Sow farm |
| Yanting Xinhao | Sow farm | Hengnan Muyun | Sow farm | Lingbao Xinliu | Sow farm |
| Zhangjiakou Xinwang | Sow farm | Wuhe Xinhai | Sow farm | Guangan Xinhao | Sow farm |
| Dong'e Xinliu | Sow farm | Pingfu Agriculture and Animal Husbandry | Sow farm | Weinan Xinliu | Sow farm |
| Yijun Xinliu | Sow farm | Zhangxiang Wangchang | Sow farm | Hainan Agriculture and Forestry Reclamation | Sow farm |
| Shibing Xinliu | Sow farm | Nanning Xinhao | Sow farm | Laibin Xinhao | Sow farm |
| Yingcheng Xinhao | Sow farm | Guigang Xinliu | Sow farm | Wuhe Xinhai | Sow farm |
| Ningming Xinhao | Sow farm | Xiajin Agriculture and Animal Husbandry | Sow farm | Xiangzhou Xinhao | Sow farm |
| Nanning Xinhao | Sow farm | Jiangxi Xinliu | Sow farm | Bijie Xinliu | Sow farm |
| Qinghua Agriculture and Animal Husbandry | Sow farm | Lingbao Xinliu | Sow farm | Gansu Xinliu | Sow farm |
| Yijun Xinliu | Sow farm | Pucheng Xinliu | Sow farm | Dong'e Xinliu | Sow farm |
| Yan'an Benyuan | Sow farm | Zhangjiakou Xinwang | Sow farm | Pingfu Agriculture and Animal Husbandry | Sow farm |
| Hengnan Muyun | Sow farm | Dong'a Xinliu | Sow farm | Zhangjiakou New Hope | Sow farm |
| Pingfu Agriculture and Animal Husbandry | Sow farm | Qinghua Agriculture and Animal Husbandry | Sow farm | Pingfu Agriculture and Animal Husbandry | Sow farm |
| Laiyang Xinhao | Sow farm | Heishan Gaojia | Sow farm | Pucheng Xinliu | Sow farm |
| Jiangxi Xinliu | Sow farm | Laiyang Xinhao | Sow farm | Shibing Xinliu | Sow farm |
| Xiangzhou Xinhao | Sow farm | Hainan State Farms | Sow farm | Yingcheng Xinhao | Sow farm |
| Yingcheng Xinhao | Sow farm | Xiajin Agriculture and Animal Husbandry | Sow farm | Bijie Xinliu | Sow farm |
| Hainan Agriculture and Reclamation | Sow farm | Hainan State Farms | Sow farm | Dong'e Xinliu | Sow farm |
| Wuhe Xinhai | Sow farm | Dong'a Xinliu | Sow farm | Laibin Xinhao | Sow farm |
| Pucheng Xinliu | Sow farm | Yijun Xinliu | Sow farm | Pingfu Agriculture and Animal Husbandry | Sow farm |
| Dong'e Xinliu | Sow farm | Gansu Xinliu | Sow farm | Xiajin Agriculture and Animal Husbandry | Sow farm |
| Laibin Xinhao | Sow farm | Ningming Xinhao | Sow farm | Gaotang Xinhao | Sow farm |
| Liaoning Xintao Wang | Sow farm | Shibing Xinliu | Sow farm | Guigang Xinliu | Sow farm |
| Zhangxin Wangchang | Sow farm | Guangan Xinhao | Sow farm | Yan'an Benyuan | Sow farm |
| Pucheng Xinliu | Sow farm | Yingcheng Xinhao | Sow farm | Qinghua Agriculture and Animal Husbandry | Sow farm |
| Kangping Newwang | Sow farm | Yingcheng Xinhao | Sow farm | Dong'e Xinliu | Sow farm |
| Dong'e Xinliu | Sow farm | Pingfu Agriculture and Animal Husbandry | Sow farm | Caoxian Xinhao | Sow farm |
| Hainan Agriculture and Reclamation | Sow farm | Xiangzhou Xinhao | Sow farm | Guangyuan Xinhao | Sow farm |
| Caoxian Xinhao | Sow farm | Ningming Xinhao | Sow farm | Jiangxi Xinliu | Sow farm |
| Pucheng Xinliu | Sow farm | Yan'an Benyuan | Sow farm | Langzhong Xinliu | Sow farm |
| Ningming Xinhao | Sow farm | Zhangjiakou Xinwang | Sow farm | Heyang Xinliu | Sow farm |
| Nanning Xinhao | Sow farm | Pucheng Xinliu | Sow farm | Wuhe Xinhai | Sow farm |
| Jiangxi Xinliu | Sow farm | Laibin Xinhao | Sow farm | Yijun Xinliu | Sow farm |
| Weinan Xinliu | Sow farm | Wuhe Xinhai | Sow farm | Yichun New Hope | Sow farm |
| Guigang Xinliu | Sow farm | Shibing Xinliu | Sow farm | Guangdong Xinhao | Sow farm |
| Shibing Xinliu | Sow farm | Nanning Xinhao | Sow farm | Shibing Xinliu | Sow farm |
| Nanning Xinhao | Sow farm | Yijun Xinliu | Sow farm | Jiangxi Xinliu | Sow farm |
| Guigang Xinliu | Sow farm | Jiangxi Xinliu | Sow farm | Pucheng Xinliu | Sow farm |
| Ningbo Xinhai | Sow farm | Pingfu Agriculture and Animal Husbandry | Sow farm | Yan'an Benyuan | Sow farm |
| Pingfu Agriculture and Animal Husbandry | Sow farm | Ningming Xinhao | Sow farm | Wuhe Xinhai | Sow farm |
| Guangan Xinhao | Sow farm | Caoxian Xinhao | Sow farm | Yijun Xinliu | Sow farm |
| Guangyuan Xinhao | Sow farm | Pingfu Agriculture and Animal Husbandry | Sow farm | Jiangxi Xinliu | Sow farm |
| Yan'an Benyuan | Sow farm | Ningbo Xinhai | Sow farm | Hainan Agriculture and Forestry Reclamation | Sow farm |
| Guangdong Xinhao | Sow farm | Laibin Xinhao | Sow farm | Wuhe Xinhai | Sow farm |
| Yan'an Benyuan | Sow farm | Pingfu Agriculture and Animal Husbandry | Sow farm | Nanning Xinliu | Sow farm |
| Guangdong Xinhao | Sow farm | Dong'a Xinliu | Sow farm | Letong Xinhai | Sow farm |
| Yucheng Xinliu | Sow farm | Guigang Xinliu | Sow farm | Ningming Xinhao | Sow farm |
| Gansu Xinliu | Sow farm | Yichun Xinwang | Sow farm | Gansu Xinhao | Sow farm |
| Gaotang Xinhao | Sow farm | Guangyuan Xinhao | Sow farm | Pingfu Agriculture and Animal Husbandry | Sow farm |
| Pingfu Agriculture and Animal Husbandry | Sow farm | Gansu Xinliu | Sow farm | Wuhe Xinhai | Sow farm |
| Ningming Xinhao | Sow farm | Kangping Xinwang | Sow farm | Nanning Xinhao | Sow farm |
| Nanning Xinhao | Sow farm | Dong'a Xinliu | Sow farm | Jiaxiang Xinliu | Sow farm |
| Dong'e Xinliu | Sow farm | Guangdong Xinhao | Sow farm | Kangping New Hope | Sow farm |
| Guangdong Xinhao | Sow farm | Jiangxi Xinliu | Sow farm | Xiangzhou Xinhao | Sow farm |
| Gansu Xinhao | Sow farm | Wannian Xinhai | Sow farm | Zhangjiakou New Hope | Sow farm |
| Nanning Xinliu | Sow farm | Kangping Xinwang | Sow farm | Caoxian Xinhao | Sow farm |
| Xiangzhou Xinhao | Sow farm | Yan'an Benyuan | Sow farm | Guangyuan Xinhao | Sow farm |
| Guigang Xinliu | Sow farm | Ningming Xinhao | Sow farm | Ningming Xinhao | Sow farm |
| Yichun Xinwang | Sow farm | Yucheng Xinliu | Sow farm | Zhejiang Xinhai | Sow farm |
| Nanning Xinliu | Sow farm | Wuhe Xinhai | Sow farm | Guangdong Xinhao | Sow farm |
| Pingfu Agriculture and Animal Husbandry | Sow farm | Nanning Xinhao | Sow farm | Gansu Xinliu | Sow farm |
| Caoxian Xinhao | Sow farm | Lezhi Xinhai | Sow farm | Ningming Xinhao | Sow farm |
| Guangyuan Xinhao | Sow farm | Yan'an Benyuan | Sow farm | Nanning Xinhao | Sow farm |
| Gansu Xinhao | Sow farm | Laibin Xinhao | Sow farm | Guigang Xinliu | Sow farm |
| Pucheng Xinliu | Sow farm | Guangdong Xinhao | Sow farm | Wannian Xinhai | Sow farm |
| Ningbo Xinhai | Sow farm | Guigang Xinliu | Sow farm | Caoxian Xinhao | Sow farm |
| Yucheng Xinliu | Sow farm | Nanning Xinliu | Sow farm | Guangdong Xinhao | Sow farm |
| Jiaxiang Xinliu | Sow farm | Xiangzhou Xinhao | Sow farm | Ningming Xinhao | Sow farm |
| Yanting Xinhao | Sow farm | Pucheng Xinliu | Sow farm | Guigang Xinliu | Sow farm |
| Rugao Xinhao | Sow farm | Nanning Xinliu | Sow farm | Kangping New Hope | Sow farm |
| Yichun Xinwang | Sow farm | Guangdong Xinhao | Sow farm | Pucheng Xinliu | Sow farm |
| Heyang Xinliu | Sow farm | Nanning Xinhao | Sow farm | Gansu Xinhao | Sow farm |
| Caoxian Xinhao | Sow farm | Yichun Xinwang | Sow farm | Yucheng Xinliu | Sow farm |
| Wannian Xinhai | Sow farm | Gansu Xinhao | Sow farm | Yanting Xinhao | Sow farm |
| Gansu Xinliu | Sow farm | Yanting Xinhao | Sow farm | Caoxian Xinhao | Sow farm |
| Xiangzhou Xinhao | Sow farm | Yucheng Xinliu | Sow farm | Xiangzhou Xinhao | Sow farm |
| Yanting Xinhao | Sow farm | Caoxian Xinhao | Sow farm | Yanting Xinhao | Sow farm |
| Yingtan Xinliu | Sow farm | Guangyuan Xinhao | Sow farm | Xiangzhou Xinhao | Sow farm |
| Yingtan Xinliu | Sow farm | Jiaxiang Xinliu | Sow farm | Guangyuan Xinhao | Sow farm |
| Hainan Xinliu | Sow farm | Heyang Xinliu | Sow farm | Yucheng Xinliu | Sow farm |
| Caoxian Xinhao | Sow farm | Gansu Xinhao | Sow farm | Yan'an Benyuan | Sow farm |
| Jiaxiang Xinliu | Sow farm | Yanting Xinhao | Sow farm | Laibin Xinhao | Sow farm |
| Hainan Xinliu | Sow farm | Yingtan Xinliu | Sow farm | Guangdong Xinhao | Sow farm |
| Xiangzhou Xinhao | Sow farm | Xiangzhou Xinhao | Sow farm | Dong'e Xinliu | Sow farm |
| Guangdong Xinhao | Sow farm | Gaotang Xinhao | Sow farm | Wuhe Xinhai | Sow farm |
| Wannian Xinhai | Sow farm | Guangdong Xinhao | Sow farm | Jiaxiang Xinliu | Sow farm |
| Guigang Xinliu | Sow farm | Caoxian Xinhao | Sow farm | Heyang Xinliu | Sow farm |
| Zhaoqing Xinhao | Sow farm | Xiangzhou Xinhao | Sow farm | Nanning Xinhao | Sow farm |
| Zhaoqing Xinhao | Sow farm | Yingtan Xinliu | Sow farm | Ningming Xinhao | Sow farm |
| Heyang Xinliu | Sow farm | Wannian Xinhai | Sow farm | Yingtan Xinliu | Sow farm |
| Ruyuan Xinhao | Sow farm | Guigang Xinliu | Sow farm | Guigang Xinliu | Sow farm |
| Zhaoqing Xinhao | Sow farm | Pucheng Xinliu | Sow farm | Jiangxi Xinliu | Sow farm |
| Zhaoqing Xinhao | Sow farm | Jiaxiang Xinliu | Sow farm | Wannian Xinhai | Sow farm |
| Ruyuan Xinhao | Sow farm | Hainan Xinliu | Sow farm | Ruyuan Xinhao | Sow farm |
| Xiajiang Xinliu | Sow farm | Caoxian Xinhao | Sow farm | Yingtan Xinliu | Sow farm |
| Liaoning Xintao Wang | Sow farm | Guigang Xinliu | Sow farm | Nanning Xinliu | Sow farm |
| Linyi Breeding Pig | Sow farm | Heyang Xinliu | Sow farm | Pucheng Xinliu | Sow farm |
| Laiyang Xinmu | Sow farm | Ruyuan Xinhao | Sow farm | Ruyuan Xinhao | Sow farm |
| Benxiang Liji | Sow farm | Hainan Xinliu | Sow farm | Letong Xinhai | Sow farm |
| Xianyang Yongxiang | Sow farm | Zhaoqing Xinhao | Sow farm | Xiajiang Xinliu | Sow farm |
| Yan'an Benyuan | Sow farm | Zhaoqing Xinhao | Sow farm | Zhaoqing Xinhao | Sow farm |
| Laiyang Xinmu | Sow farm | Zhaoqing Xinhao | Sow farm | Zhaoqing Xinhao | Sow farm |
| Laiyang Xinmu | Sow farm | Zhaoqing Xinhao | Sow farm | Zhaoqing Xinhao | Sow farm |
| Anyue Xingxin Xin | Fattening farm | Xiajiang Xinliu | Sow farm | Hainan Xinliu | Sow farm |
|  |  | Ruyuan Xinhao | Sow farm | Zhaoqing Xinhao | Sow farm |
|  |  | Liaoning New Taowang | Sow farm | Hainan Xinliu | Sow farm |
|  |  | Rugao Xinhao | Sow farm | Liaoning New Tao Wang | Sow farm |
|  |  | Laiyang Xinmu | Sow farm | Laiyang New Livestock | Sow farm |
|  |  | Benxiang Li Family | Sow farm | Xianyang Yongxiang | Sow farm |
|  |  | Linyi Breeding Pig | Sow farm | Benxiang Li Jia | Sow farm |
|  |  | Xianyang Yongxiang | Sow farm | Linyi Breeding Pig | Sow farm |
|  |  | Yan'an Benyuan | Sow farm | Yan'an Benyuan | Sow farm |
|  |  | Laiyang Xinmu | Sow farm | Rugao Xinhao | Sow farm |
|  |  | Laiyang Xinmu | Sow farm | Laiyang New Livestock | Sow farm |
|  |  | Huanghua Xinhao | Sow farm | Laiyang New Livestock | Sow farm |
|  |  | Huairou Agriculture and Animal Husbandry | Sow farm | Huanghua Xinhao | Sow farm |
|  |  | Laiyang Xinhao | Sow farm | Huairou Agriculture and Animal Husbandry | Sow farm |
|  |  | Laibin Xinhao | Sow farm | Laiyang Xinhao | Sow farm |
|  |  | Yan'an Benyuan | Sow farm | Laibin Xinhao | Sow farm |
|  |  | Laibin Xinhao | Sow farm | Yan'an Benyuan | Sow farm |
|  |  | Laiyang Xinhao | Sow farm | Laibin Xinhao | Sow farm |
|  |  |  |  | Laiyang Xinhao | Sow farm |
|  |  |  |  | Hebei Xinhao | Sow farm |
|  |  |  |  | Hubei Xinhao | Sow farm |
|  |  |  |  | Dong'e Xinliu | Sow farm |
|  |  |  |  | Laibin Xinhao | Sow farm |
|  |  |  |  | Caoxian Xinhao | Sow farm |
|  |  |  |  | Huaiyin New Chi | Sow farm |
|  |  |  |  | Xiajin Agriculture and Animal Husbandry | Sow farm |
